# Experimental Study Redefines the Mechanism of Heptamethine Cyanine Phototruncation

**DOI:** 10.1021/jacs.5c18903

**Published:** 2026-03-10

**Authors:** Nasrulla Majid Khan, Gabriel Glotz, Jana Okoročenkova, Jakub Dostál, Miroslav Kloz, Max T. G. M. Derks, Aleksandr Y. Pereverzev, Dmytro Neshchadin, Jana Roithová, Petr Klán

**Affiliations:** † Department of Chemistry, Faculty of Science, Masaryk University, 62500 Brno, Czech Republic; ‡ RECETOX, Faculty of Science, Masaryk University, 62500 Brno, Czech Republic; § The Extreme Light Infrastructure Facility ERIC, Za Radnicí 835, 25241 Dolní Břežany, Czech Republic; ∥ Institute for Molecules and Materials, Faculty of Science, 6029 Radboud University, Heyendaalseweg 135, Nijmegen 6525 AJ, The Netherlands; ⊥ Institute of Physical and Theoretical Chemistry, Graz University of Technology, Stremayrgasse 9, 8010 Graz, Austria

## Abstract

Cyanine dyes are popular chromophores used in contemporary biomedical fields due to their tunable near-infrared absorption and fluorescence properties. One such application of cyanines involves the photochemical shortening of the polymethine chain by a two-carbon fragment. This process is referred to as phototruncation or photoblueing because the resulting cyanine’s absorption band shifts hypsochromically. A recent quantum-chemical study has proposed a mechanism for this process; however, it cannot explain the substantial enhancement of the reaction under specific conditions. Here, we present the results of an extensive investigation of phototruncation of the prototypical heptamethine cyanine dye (**Cy7**). To elucidate the underlying mechanism, a comprehensive analytical approach was employed, encompassing kinetic studies, isotopic labeling, transient spectroscopy, femtosecond stimulated Raman spectroscopy, collision-induced dissociation, and infrared photodissociation spectroscopy. Our findings demonstrate that phototruncation occurs efficiently in aqueous solutions of a specific organic buffer composition. It is sensitive to reactant concentrations and pH, and its efficiency increases with the addition of electron acceptors. The reaction involves ultrafast electron transfer from a singlet-excited cyanine dye to oxygen, forming a radical dication intermediate. This intermediate reacts with another oxygen molecule and subsequently with a buffer constituent featuring an ethanolamine scaffold. The reaction continues with oxidation, cyclization, and elimination steps to form pentamethine cyanine (**Cy5**) in yields up to 33%. We also demonstrate that **Cy5** undergoes phototruncation via the same mechanism but with lower efficiency. The triplet-excited **Cy7** also undergoes phototruncation. The findings of this study lay the foundation for the further exploitation of this unique process.

## Graphical abstract

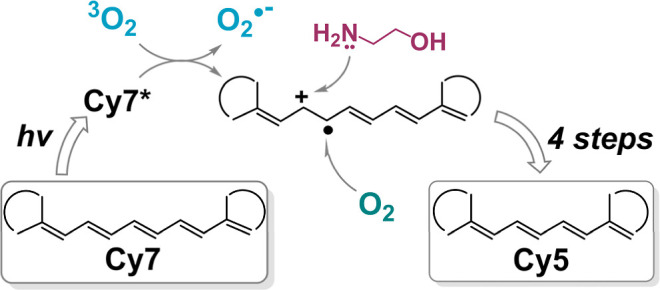

## Introduction

Heptamethine cyanine dyes represent a well-known class of organic near-infrared (NIR) fluorophores that play an indispensable role in modern chemistry and biology.^1^ These molecules are characterized by a polymethine chain with two heterocyclic end groups and unique physicochemical properties,^2^ including high molar absorption coefficients and narrow absorption and emission bands.^3,4^ Cyanine dyes are traditionally classified by the number of carbon atoms in their polymethine chains. The most commonly used dyes are the pentamethine (**Cy5**) and heptamethine (**Cy7**) cyanines, which have five and seven carbon atoms in their chains, respectively. Their distinctive conjugated systems are responsible for their unique photophysical properties and chemical reactivity, as reflected in their absorption spectra. The absorption maximum of **Cy5** (∼635 nm) shifts bathochromically to ∼735 nm in **Cy7**.^5^ Furthermore, their high biocompatibility and low toxicity make these dyes ideal candidates for in vivo imaging and sensing.^6,7^ Accordingly, heptamethine cyanine dyes have been used for super-resolution imaging,^8–10^ optical cancer imaging,^11^ photodynamic therapy,^12,13^ phototriggered drug delivery,^14,15^ pH,^16,17^ and reactive oxygen species sensing,^18–20^ prompting intensive research into their photodegradation pathways. The relaxation pathways of photoexcited heptamethine cyanine dyes include thermal deactivation^21^ coupled with photoisomerization^22,23^ and inefficient fluorescence.^23,24^ The intersystem crossing rate that affords the first excited triplet state is usually very low (<1%). The decreased photostability of these dyes in aerated solutions is attributed to their reaction with reactive oxygen species, such as singlet oxygen (^1^O_2_), which is produced by the photosensitization of molecular oxygen with the triplet state of the dye.^25^ This oxidative cleavage process is called photobleaching.^26–28^

An unanticipated photodegradation pathway was reported for heptamethine^29^ and pentamethine^30^ cyanines. This reaction involves the shortening of the polymethine chains by two-carbon fragments upon irradiation, a process referred to as phototruncation or photoblueing,^29^ to form products with a hypsochromically shifted absorption maxima. In addition, cyanine truncation also occurs in the dark at higher temperatures and in the presence of a base.^31,32^

The photoconversion of cyanine dyes, leading to their truncation, was utilized for cell-migration monitoring already more than a decade ago.^33,34^ Schnermann, Sauer, Greer, and co-workers investigated the phototruncation mechanism of heptamethine (Scheme 1) and pentamethine cyanine dyes.^29,35^ Using a combination of experimental results and quantum-chemical calculations, they proposed a mechanism that proceeds via an intramolecular rearrangement involving singlet oxygen, leading to the expulsion of 2-hydroperoxyethen-1-ol (Scheme 2a). In addition, they showed the substantial impact of the buffer on the reaction outcome. The irradiation of **Cy7** derivatives in phosphate-buffered saline (PBS) buffer led to very low yields of phototruncation (<1–5%).^36^ However, using 3-cyclohexylamino-2-hydroxy-1-propanesulfonic acid (CAPSO), a biologically relevant buffer, resulted in the highest yield (17%).^29^

**Scheme 1:**
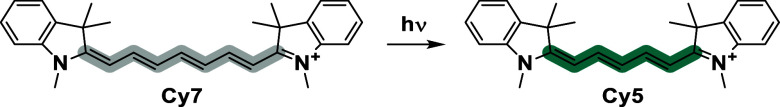
Phototruncation of Cy7 to Cy5

**Scheme 2:**
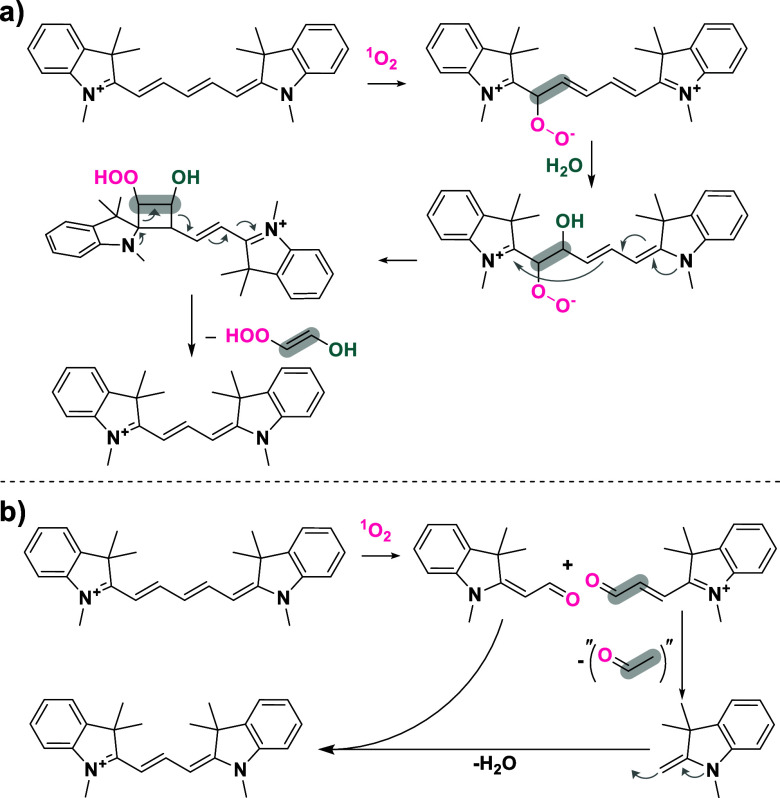
A Brief Outline of the Proposed Phototruncation Mechanism by the Groups of (a) Schnermann and Co-workers (Reproduced from Ref 29; Copyright 2021 American Chemical Society) and (b) Lee and Co-workers (Reproduced from Ref 30; Copyright 2021 American Chemical Society)

Lee and co-workers reported on the phototruncation mechanism of pentamethine cyanine.^30^ In contrast to the conclusions reported by Schnermann, they demonstrated that phototruncation occurred through the cleavage of the polymethine chain, a process facilitated by singlet oxygen and resulting in the production of two carbonyl fragments (Scheme 2b). After decarbonylation, one of the initial electrophilic fragments undergoes nucleophilic attack by another fragment, forming a nucleophile that is two carbons shorter (Fischer’s base). Despite the mechanistic inconsistencies between the proposed phototruncation mechanisms and the overall low yields, the phototruncation process was used to develop a phototruncation-assisted cell tracking (PACT) strategy for in vivo cell-migration monitoring.^36^ Truncation was also applied for the single-molecule localization microscopy (SMLM).^29^

Here, we present a thorough study of the phototruncation mechanism of prototypical heptamethine cyanine (**Cy7**), leading to pentamethine cyanine (**Cy5**; Scheme 1), motivated by the following questions: Why is the phototruncation yield higher in CAPSO buffer than in PBS and Britton-Robinson (BRB) buffers? Is the phototruncation mechanism a general phenomenon or specific to unique buffer components or reaction conditions? Can we experimentally prove the mechanism recently proposed^29^ by quantum-chemical calculations? Can the phototruncation yield be significantly increased?

During our research, we found that the intrinsic complexity of the phototruncation mechanism, the inability to spectroscopically or chemically trap all short-lived reaction intermediates, and the unwanted side-reaction pathways prevented us from conducting a heuristic analysis based on Occam’s razor. Initially, many of the results were fundamentally contradictory, and many additional experiments designed to confirm the developed hypotheses failed. Our article presents substantial experimental data, each of which played a pivotal role in the formulation of the final mechanism.

Although some of the original conclusions^29^ have been confirmed, this work proposes a mechanism that differs significantly from the original hypothesis. It shows that the conditions for efficient phototruncation to occur are specific and that the reaction is not a general phenomenon. A wide range of experimental evidence was based on various methods, including kinetic and kinetic isotope effect studies, isotopic labeling, transient spectroscopy, femtosecond stimulated Raman spectroscopy, collision-induced dissociation mass spectrometry, and infrared photodissociation spectroscopy.

## Results and Discussion

### Effects of Buffer Composition and Reaction Optimization

Schnermann and his colleagues reported that the yields of **Cy7** phototruncation to **Cy5** in PBS and BRB buffers were low, whereas the reaction in CAPSO was much more efficient.^29^ It was further reported that the reaction is strongly pH-dependent and that truncation does not occur in nonaqueous environments. This unexpected result suggests that the medium’s quality is directly related to the reaction mechanism. Therefore, we first focused on optimizing the reaction conditions. All the following phototruncation experiments were performed with LEDs at λ = 735 nm under aerated conditions.

We confirmed the previous finding^29^ that the reaction results in small **Cy5** yields of ≤1% in PBS (pH = 7.4) and BRB (pH = 7.4, 9.0, and 10.0), while the irradiation of **Cy7** in a CAPSO buffer (pH = 10) resulted in a yield of 12%, which is less than the reported value of 17% (Table 1).

**Table 1:**
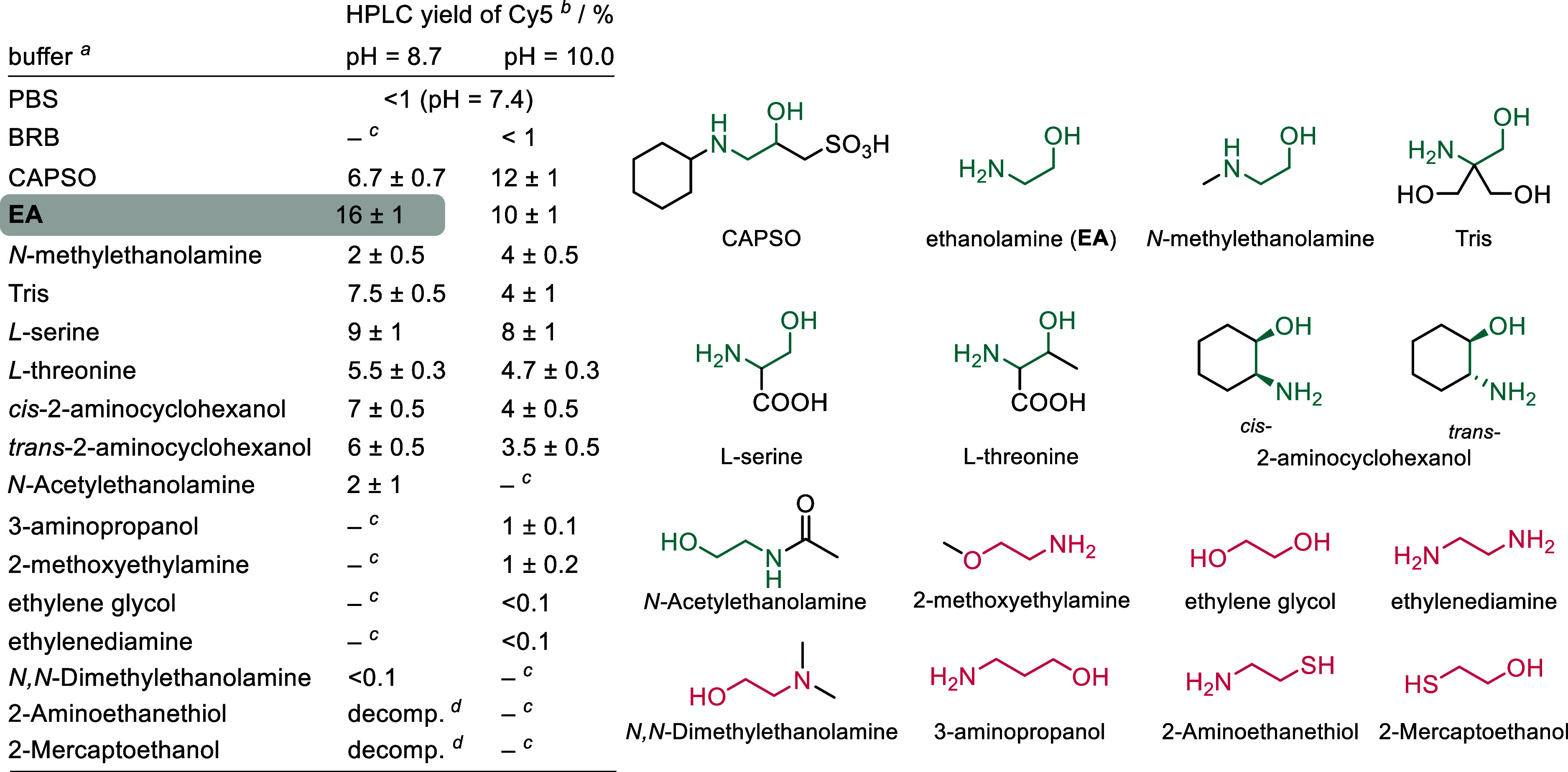
Screening of Various Buffers^e^

a Buffer concentration = 500 mM in H_2_O; pH adjusted using acetic acid or NaOH.

b The yield was determined by HPLC (calibrated with analytically pure **Cy7** and **Cy5** samples) and corrected for 100% conversion of **Cy7**.

c Not determined.

d **Cy7** rapidly decomposes.

e Reaction condition for all experiments: **Cy7** = 10 μM in buffer solution with 1% DMSO as a cosolvent; irradiated in a cuvette under aerated conditions at 22 ± 1 °C with LEDs (λ = 735 nm).

The main difference between inorganic PBS and BRB buffers, on the one hand, and CAPSO, on the other, was the presence of an organic substance, 3-(cyclohexylamino)-1-propanesulfonic acid, in CAPSO, possessing a basic 2-aminoethan-1-ol motif (Table 1). Therefore, we used 2-aminoethanol (ethanolamine, **EA**) and its structural derivatives and analogs as buffer constituents to determine the reason for the observed reaction selectivity. At pH = 10, **EA** was nearly as efficient a truncation agent as CAPSO itself (10%). Subsequent optimization of the reaction conditions (pH and **EA** concentrations) revealed that the highest yield (16%) was obtained at a pH of 8.7 with a 500 mM concentration of **EA** (Table 1, Figure 1a,b). Further study revealed that increasing the temperature and solution viscosity significantly decreased phototruncation yields (Figure 1c,d). The quantum yield for **Cy5** formation at the optimum pH (8.7) was Φ_**Cy5**form_ = 1.2 × 10^–5^, demonstrating that the process is quite inefficient. The degradation quantum yield (Φ_**Cy7**dec_) versus **EA** concentration shows a linear decrease from 1.6 × 10^–4^ at 20 mM to 8.3 × 10^–5^ at 1000 mM **EA** (pH = 8.7). On the other hand, Φ_**Cy5**form_ showed a volcano-like dependence with a maximum at 500 mM (1.2 × 10^–5^; Figure S33). The quantum yield ratio (Φ_**Cy5**form_/Φ_**Cy7**dec_) provides information on the phototruncation’s overall efficiency with a maximum of 0.11 at 500 mM **EA** at pH 8.7. We also experimentally confirmed that the pH remains constant throughout irradiation (Figure S25).

**Figure 1:**
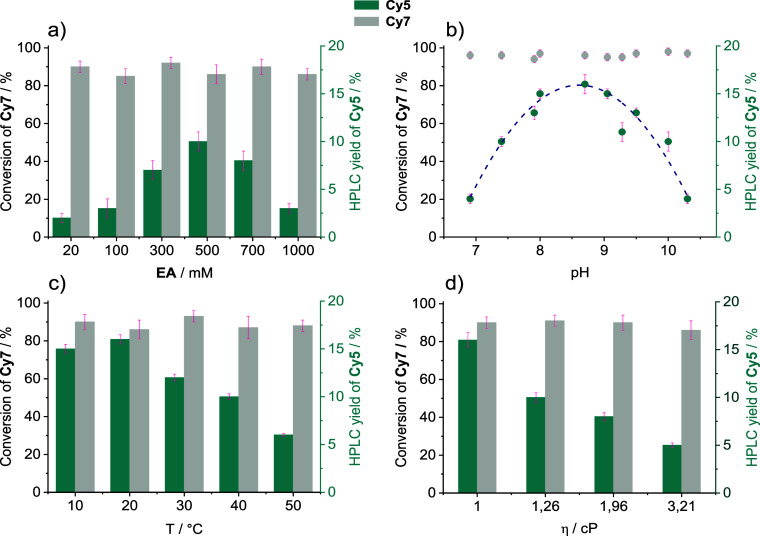
Optimization of the phototruncation conditions: (a) **EA** concentration vs **Cy5** yields (pH = 10, measured at 22 ± 1 °C); (b) pH vs **Cy5** yields (**EA** = 500 mM; measured at 22 ± 1 °C); (c) **Cy5** yields at different temperatures (**EA** = 500 mM, pH = 8.7); and (d) viscosity vs **Cy5** yields (**EA** = 500 mM, pH = 8.7, measured at 22 ± 1 °C; viscosity adjusted by addition of sucrose). Conditions for all measurements: **Cy7** = 10 μM, buffer solutions with 1% DMSO as a cosolvent, irradiated with LEDs (735 nm) under aerated conditions. The reaction was monitored by UV–vis spectroscopy, and the yields were determined by HPLC and are corrected for 100% **Cy7** conversion.

The introduction of various substituents on one or both carbons of the **EA** scaffold in derivatives tris­(hydroxymethyl)­aminomethane (Tris), l-serine, or l-threonine resulted in lower, yet still significant, truncation yields at both pH levels (Table 1; we did not search for an optimum pH for each compound). Configurationally different *cis*- and *trans*-2-aminocyclohexanols produced nearly identical phototruncation yields. However, structural deviation from the basic **EA** skeleton always led to a significant decrease in **Cy5** yield. These modifications included chain extension (3-aminopropanol), blocking of the hydroxyl group (2-methoxyethylamine), blocking of the amino group (*N*,*N*-dimethylethanolamine), replacing the amino group with a hydroxyl group (ethylene glycol), and replacing the hydroxyl group with an amino group (ethylenediamine). The replacement of the amino group with the acetamide group (*N*-acetylethanolamine) resulted in the formation of **Cy5** with a very low yield (2%). Blocking the nitrogen atom in the **EA** fragment (CAPSO or *N*-methylethanolamine) led to still relatively efficient truncation compared to substitution on the oxygen atom (2-methoxyethylamine), which nearly stopped the process. Structurally similar compounds, such as ethylene glycol or ethylene diamine, did not promote phototruncation. The presence of 2-aminoethanolethiol and 2-mercaptoethanol caused the nonproductive decomposition of **Cy7**. In conclusion, the simple **EA** structure appeared to be the best agent and buffer component for this process.

We also evaluated the extent of phototruncation in chain-3′-substituted cyanines (methoxy-, fluoro-, and bromo-substituents) that were recently utilized in tracking immune cells into tumor-draining lymphatics.^36^ However, under our optimized conditions, the chemical yields of the corresponding **Cy5** derivatives were found to be under 3%, which makes the parent unsubstituted heptamethine cyanine a significantly better phototruncation moiety than the chain-substituted derivatives.

Phototruncation was reported to proceed only in aqueous buffer media.^29^ Initially, we used 1% DMSO as a cosolvent for solubility reasons. However, the phototruncation yield was affected by the concentration of DMSO as a cosolvent. Increasing the DMSO concentration (0.5–15%) resulted in a decrease in **Cy5** yield (17–10%, respectively; Figure S27). Because changing to a 1% methanol cosolvent had no noticeable effect on phototruncation, we tested the maximum amount of methanol that would still allow the reaction to proceed. We observed a linear decrease in the rate of **Cy7** consumption and **Cy5** yield as the methanol concentration increased. The yield of **Cy5** decreased to 2 ± 1% at 33% methanol in **EA** buffer; subsequent to this, no further phototruncation was detected (Figure S28).

### Molecularity and Regioselectivity of Chain Truncation

Schnermann and co-workers provided evidence for phototruncation of **Cy5** via an intramolecular rearrangement through a crossover experiment. On the contrary, the findings of the group of Lee suggested that the **Cy5** phototruncation to trimethine cyanine (**Cy3**) is intermolecular.^30^ In this study, we aimed to verify the molecularity of phototruncation under optimized conditions and to determine the regioselectivity of 2-carbon fragment cleavage.

In the crossover experiment, an equimolar mixture of **Cy7** and **Cy7-** *d*_**6**_ (dimethyl-*d*_6_) was irradiated with 735 nm LEDs, and the reaction was analyzed by high-resolution mass spectrometry (HRMS). Only **Cy5** and **Cy5-** *d*_**6**_ were observed, while the **Cy5-** *d*_**3**_ crossover product was not detected (Scheme 3, Figure S86). We concluded that phototruncation occurs intramolecularly and supported the previous observation.^29^ To identify the specific fragment that is cleaved, we designed different chain-deuterated cyanines, dideuterated **Cy7–3′,5′-** *d*_**2**_, **Cy7–2′,6′-** *d*_**2**_ and pentadeuterated **Cy7–2′,3′,4′,5′,6′-** *d*_**5**_ (Scheme 4, Figures S74–S80). Following the irradiation of all three deuterated cyanines and HRMS analysis, we found that the C2′–C3′ fragment, rather than the C1′–C2′ fragment,^29^ is the only common species to eliminate.

**Scheme 3:**
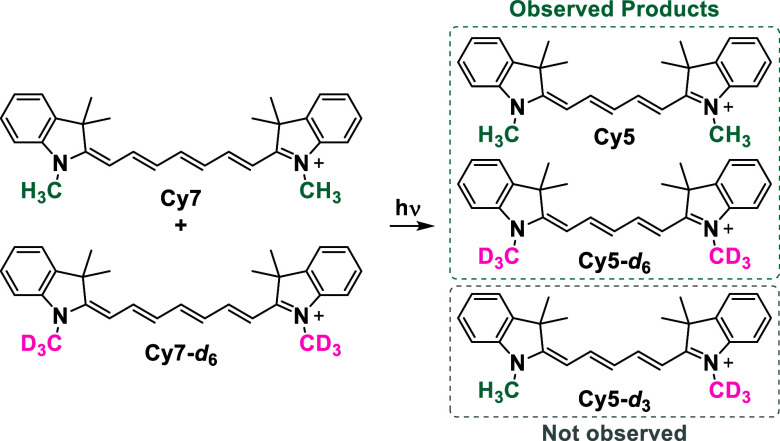
Phototruncated Products in a Crossover Experiment^a^

a Reaction conditions: an equimolar mixture of the starting material in **EA** buffer, 500 mM, pH = 8.7; irradiated at 735 nm.

**Scheme 4:**
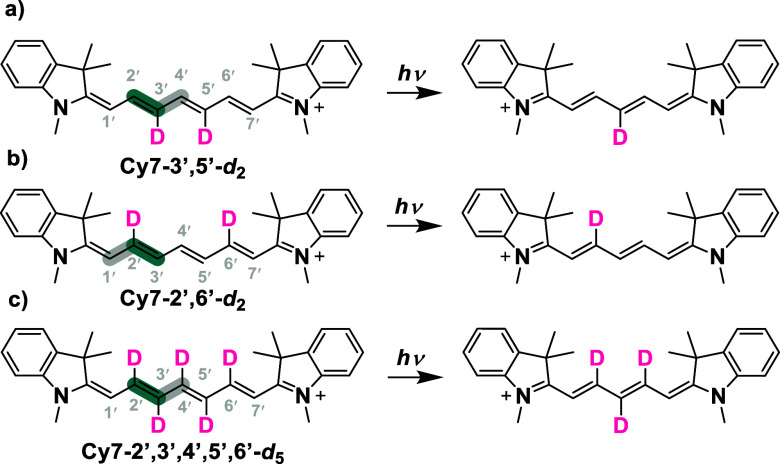
Regioselectivity of Phototruncation^a^

a Reaction conditions: (a) **Cy7–3′,5′-** *d*_**2**_, (b) **Cy7–2′,6′-** *d*_**2**_ and (c) **Cy7–2′,3′,4′,5′,6′-** *d*_**5**_ (50 μM of **Cy7**, **EA** buffer, 500 mM, pH = 8.7, with 1% MeOH; 22 °C; irradiated at 735 nm), the photoproducts monitored by HRMS (their structural assignment is based on the analysis of all three experiments mentioned). Color coding indicates possible elimination of two-carbon fragments; only the bond highlighted in dark green is common to all experiments.

### Molecular Oxygen and Reactive Oxygen Species

The irradiation of **Cy7** in the **EA** buffer under degassed (freeze–thaw–pump) conditions did not result in phototruncation. At the same time, the photodegradation of the starting material was almost completely halted. The results thus indicated that some form of molecular oxygen is essential for the process. In addition to the proposed singlet oxygen (^1^O_2_),^29^ other forms of reactive oxygen species (ROS) or triplet (ground-state) oxygen were considered. We first focused on singlet oxygen.

Photoexcitation of **Cy7** results in the singlet excited state, which undergoes intersystem crossing with a low efficiency (Φ_ISC_ = 8.9 × 10^–3^).^5^ This excited state can sensitize molecular oxygen, resulting in the formation of ^1^O_2_. Therefore, a series of ^1^O_2_ quenchers and traps were examined, revealing no substantial influence of ^1^O_2_ on the **Cy5** yield (<0.5%; Table 2). This finding was accentuated by the observation that thermally produced ^1^O_2_ by 1-methylnaphthalene-4-propionate endoperoxide^29^ (**EN**) did not lead to a notable **Cy5** yield under the study conditions in the absence of light (Figure S97). Contrary to the initial hypothesis,^29^ ^1^O_2_ did not appear to significantly impact the overall truncation mechanism.

**Table 2:** Effects of ^1^O_2_ Quenchers, Traps, and Thermally Generated ^1^O_2_ on Cy5 Yields^h^

additive^a^	HPLC yield of **Cy5** ^b^/%
–^c^	16 ± 1
Sodium azide^d^	15 ± 1
DABCO^e^	13 ± 1
Furfuryl alcohol^f^	11 ± 2
**EN** (^1^O_2_)^g^	<0.5 ± 0.05

a The concentration of an additive = 10 mM.

b Reaction was monitored by UV–vis spectroscopy, the yield was determined by HPLC.

c No additives.

d ^1^O_2_ quencher.^37^

e ^1^O_2_ quencher.^38^

f ^1^O_2_ trap.^39^

g Thermally generated ^1^O_2_ from 1-methylnaphthalene-4-propionate endoperoxide (**EN**) at 30 ± 2 °C in the absence of light.

h Condition for all experiments; **Cy7** = 10 μM, **EA** buffer (500 mM in H_2_O, pH = 8.70, adjusted using acetic acid with 1% DMSO as a cosolvent; irradiated at 22 ± 1 °C at 735 nm).

After determining that ^1^O_2_ is not responsible for producing **Cy5**, we tested hydroxyl radical (HO^•^) traps, such as 5,5-dimethyl-1-pyrroline *N*-oxide (DMPO)^40^ and *t*-butyl alcohol.^41^ They did not substantially change the **Cy5** yield (Table 3, Figures S4 and S5). However, the use of *N*-acetylcysteine, glutathione, and ascorbic acid as powerful radical scavengers^42–44^ led to the complete inhibition of the phototruncation reaction, resulting in **Cy5** yields falling below the detection limit. This observation suggests the potential involvement of radical species. Furthermore, the presence of **Cy7**-*N*-acetylcysteine and **Cy7**-*N*-acetylcysteine-O_2_ adducts was detected by HRMS. The structural elucidation of these adducts was facilitated using collision-induced dissociation^45,46^ (CID, Figures S90–S95) and infrared photodissociation spectroscopy^47,48^ (IRPD, Figure S114). The identified intermediates are discussed in more detail later in the text. This finding is consistent with the report by Cosa and co-workers on the photoreactivity of **Cy5** in the presence of thiols,^49^ in which they argue that the C2′-thiol adduct of **Cy5** forms through recombination of the geminate radical pair. Finally, the enzyme superoxide dismutase (SOD), as a highly specific trap of the superoxide radical anion (O_2_^•–^),^50^ was utilized. The addition of SOD (≥300 units/mL) had no noticeable effect on the phototruncation yield; thus, we concluded that O_2_^•–^ is not responsible for phototruncation.

**Table 3:** Effects of Various ROS Traps and Radical Scavengers on Cy5 Yields^k^

Additive^a^	HPLC Yield of Cy5^b^/%
-^c^	16 ± 1
**DMPO** ^d^	15 ± 1
*t*-Butyl alcohol^e^	16 ± 1
*N*-Acetylcysteine^f^	<0.1
Glutathione^g^	<0.1
Ascorbic acid^h^	<0.1
**SOD** ^i^	16 ± 0.5
**NBT** ^j^	30 ± 0.5

a Additive concentrations = 2 mM.

b Reaction was monitored by UV–vis spectroscopy, the yield was determined by HPLC.

c No additive.

d HO^•^ trap.^40^

e HO^•^ trap.^41^

f Radical scavenger.^42^

g Radical scavenger.^43^

h Radical scavenger and electron donor.^44^

i (≥300 units/mL), O_2_^•–^ scavenger.^50^

j O_2_^•–^ trap and electron acceptor.^51^

k Condition for all experiments: **Cy7** = 10 μM, **EA** buffer (500 mM in H_2_O, pH = 8.7, with 1% DMSO as a cosolvent), irradiated at 735 nm in a cuvette under aerated conditions (at 22 ± 1 °C).

### Multiplicity of the Productive State and Electron Transfer (ET) Process

The subsequent phase of the investigation focused on identifying the reactive cyanine state responsible for phototruncation. In our recent study, we conducted an in-depth analysis of **Cy7** photoinduced processes using femtosecond stimulated Raman (FSR) spectroscopy.^52–55^ This analysis revealed an ultrafast (<75 fs, instrument response function) formation of the cyanine radical dication (**Cy7** ^**•+**^; the second positive charge is implied in the abbreviation **Cy7**) and O_2_^•–^ (at 1147 cm^–1^) pair, formed via photoinduced electron transfer (PET). This result implies the existence of a **Cy7**–O_2_ ground-state complex prior to excitation, thereby indicating the exited singlet state (S_1_) as the productive state. Furthermore, femtosecond broadband transient absorption spectroscopy (fs-TA) was performed.^56–58^ The experimental details of the fs-TA setup, data processing, and lifetimes of the evolution-associated difference spectra are provided in the Supporting Information. Due to the exceedingly low quantum yields of the **Cy7** decomposition (see above) in the **EA** buffer, no clear correlations could be established (Figures S38–S49).

Subsequently, our focus was directed toward the triplet excited state, which has been identified as the key intermediate for phototruncation.^29,30^ As previously discussed, the formation of ^1^O_2_ via sensitization by the **Cy7** triplet is the cause of the dye bleaching but not truncation.^26^ However, the photobleaching process is inefficient due to the low **Cy7** ISC quantum yield (see above). An attempt was made to increase the triplet population by the addition of potassium iodide and triplet sensitization. Both the addition of potassium iodide (heavy-atom effect)^59^ and the triplet sensitization of **Cy7** (*E*_T_ = 27.6 kcal mol^–1^)^63^ by triplet-excited anthracene (*E*_T_ = 42.7 kcal mol^–1^)^60^ or anthracene-9,10-propanoic acid disodium salt (**2**; *E*_T_ not reported, but assumed to be similar to that of 9,10-dimethylanthracene *E*_T_ = 38.5 kcal mol^–1^ value obtained by quantum chemical calculations^61^) resulted in the increased **Cy5** yields. Trapping ^1^O_2_ produced by sensitization of either triplet species had a minimal effect on the **Cy5** yield (Table 4, Figures S7 and S8). To further elucidate whether the T_1_ state of **Cy7** contributes to phototruncation, we tested several triplet-state quenchers. The addition of cycloocta-1,3,5,7-tetraene-1-carboxylic acid (**COT-COOH**, 2 mM; the T_1_ state of **Cy7** is sufficiently long-lived: τ_T_ = 21.8 ± 2.7 μs ^64^), a well-established **Cy7** triplet quencher,^63^ had no effect on the phototruncation (Figures S9 and S10). Although triplet sensitization of **Cy7** also led to efficient truncation, quenching experiments using **COT-COOH** revealed that the singlet excited state, populated upon direct irradiation, is primarily responsible for the phototruncation process.

**Table 4:** Effects of the ISC Agents, Triplet Sensitizers, and Triplet Quenchers on Cy5 Yields^m^

Additive	HPLC Yield of Cy5^a^/%
-^b^ ^,^ ^c^	16 ± 1
Potassium iodide^c^ ^,^ ^d^	24 ± 1
Anthracene^e^ ^,^ ^f^	19 ± 2
Anthracene^e^ ^,^ ^f^ ^,^ ^g^	16 ± 1
**2** ^f^ ^,^ ^h^	20 ± 1
**2** ^f^ ^,^ ^g^ ^,^ ^h^	17 ± 0.5
**NBA** ^c^ ^,^ ^i^	28 ± 1
**CQ** ^j^ ^,^ ^k^	20 ± 1
**CQ** ^g^ ^,^ ^j^ ^,^ ^k^	32 ± 1
**CQ** ^j^ ^,^ ^k^ ^,^ ^l^	<0.3 ± 0.5

a Reaction was monitored by UV–vis spectroscopy, the yield was determined by HPLC (calibrated with analytically pure **Cy7** and **Cy5** samples).

b No additive.

c Irradiated using 735 nm LEDs.

d *c* = 5 mM; an ISC promoter.^59^

e *c* = 0.05 mM; a triplet sensitizer.^60^

f Irradiated with 365 nm LEDs.

g The addition of sodium azide (5 mM).

h *c* = 0.2 mM as a disodium salt; a triplet sensitizer.^61^

i *c* = 2.0 mM, a triplet quencher.^62^

j *c* = 2 mM.

k Irradiated with 450 nm LEDs.

l *c* = 2 mM; the solutions were degassed with a freeze–pump–thaw method.

m Condition for all experiments: **Cy7** = 10 μM in **EA** buffer (500 mM in H_2_O, pH = 8.7) with 2% MeOH as a cosolvent. Irradiated in a quartz cuvette at 22 ± 1 °C under aerated conditions.

Surprisingly, the addition of 4-nitrobenzyl alcohol (**NBA**; 2 mM in **EA** buffer, 500 mM, pH = 8.7), a water-soluble **Cy7** triplet quencher,^62^ led to a substantial increase in **Cy5** yield (28%; Table 4, Figure 2). The quantum yields also increased to Φ_**Cy5**form_ = 1.4 × 10^–4^ and Φ_**Cy7**dec_ = 4.5 × 10^–4^ under the same conditions. The ratio Φ_**Cy5**dec_/Φ_**Cy7**form_ of 0.32 reflected the measured chemical yields (Figure S35, Table S5). These results have led us to reevaluate the function of the **NBA**, prompting us to consider it not only as a physical triplet quencher but also as an additional electron acceptor, alongside O_2_.^64–66^ Therefore, we assumed that photooxidation of **Cy7** is the initial step leading to truncation in both cases. To initiate electron transfer, we designed an alternative experiment. Here, the photooxidation of **Cy7** was carried out using camphorquinone (**CQ**; Φ_ISC_ ≈ 1,^67^ *E*_1/2_ of T_1_ = 1.49 V vs NHE;^68^ Table 4). Irradiation of **CQ** with **Cy7** in **EA** buffer at 450 nm, the wavelength at which **Cy7** exhibits minimal absorbance, provided a 20% yield of **Cy5**. When ^1^O_2_ was quenched by NaN_3_, the **Cy5** yield even increased (32%, Figure S12). Again, no significant **Cy5** yield was observed in a degassed solution, despite the complete consumption of **Cy7**.

**Figure 2:**
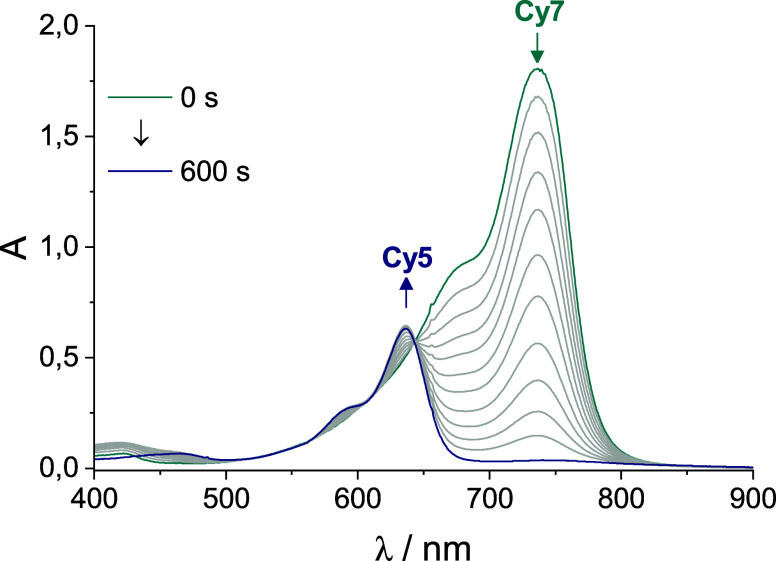
UV–vis spectra obtained upon the irradiation of **Cy7** (10 μM) by LEDs (735 nm) in the presence of **NBA** (2 mM) in **EA** buffer (500 mM, pH = 8.7) at 22 ± 1 °C.

The significant increase in **Cy5** production upon addition of **NBA** prompted us to explore other electron acceptors, such as nitroblue tetrazolium (**NBT**),^51^ methyl viologen dichloride (**MV** ^**2+**^),^70^ and sodium nitrate.^71^ The addition of each electron acceptor (2 mM) to the **EA** buffer (500 mM, pH 8.7) significantly increased the **Cy5** yield (Table 5, Figure S11), while the Φ_**Cy5**dec_/Φ_**Cy7**form_ ratio remained approximately the same (0.31; Figure S35, Table S5). Furthermore, combining **NBA** with other buffer components possessing a 2-aminoethan-1-ol motif also enhanced phototruncation. In the absence of **EA** (in the BRB buffer), the **Cy5** yields dramatically decreased (<1%).

**Table 5:** Effects of Various Electron Acceptors on Phototruncation

Buffer^a^	Additive	HPLC Yield of Cy5^b^/%
**EA** ^c^	-^d^	16 ± 1
**EA** ^c^	**NBT** ^e^	30 ± 0.5
**EA** ^c^	**NBA** ^e^	28 ± 1
**EA** ^c^	**MV** ^**2+**^ ^e^	28 ± 0.5
**EA** ^c^	sodium nitrate^e^	24 ± 2
BRB^f^	**NBA** ^e^	<1
*cis*-2-Aminocyclohexanol^c^	**NBA** ^e^	14 ± 1
*trans*-2-Aminocyclohexanol^c^	**NBA** ^e^	12 ± 1
l-serine^c^	**NBA** ^e^	18 ± 1
l-threonine^c^	**NBA** ^e^	10 ± 1

a **Cy7** = 10 μM in a buffer solution (with 2% DMSO as a cosolvent), irradiated at 22 ± 1 °C under aerated conditions with LEDs (735 nm).

b Reaction was monitored by UV–vis spectroscopy; the yield was determined by HPLC.

c 500 mM in H_2_O, pH = 8.70, pH was adjusted using acetic acid or NaOH, depending on the buffer type.

d No additive.

e Additive concentration = 2 mM.

f Standard Britton-Robinson buffer composed of 40 mM H_3_BO_3_, 40 mM H_3_PO_4_, and 40 mM AcOH;^69^ pH adjusted to 8.7.

To gain further insight into the redox processes, cyclic voltammetry was performed on **Cy7** (Figure S60). Considering the redox potentials and the **Cy7** excited state energies of the S_1_ and T_1_ states, as well as the estimated oxidation peak potential of **EA** (Figure S61), it can be concluded that ET between **EA** and the S_1_ or T_1_ states of **Cy7** is not feasible. Conversely, ET from **Cy7** to molecular oxygen is exergonic from both the S_1_ state (−6.9 kcal mol^–1^) and the T_1_ state (−0.5 kcal mol^–1^). This indicates that both the S_1_ and T_1_ states can contribute to ET, yielding the **Cy7** ^**•+**^ and O_2_^•–^ pair. These findings are important because they offer a unifying perspective on the initially contradictory experimental results from FSR spectroscopy (S_1_ state) and sensitization experiments (T_1_ state). The analysis also allowed us to estimate the feasibility of ET. The Δ*G*_PET_ was calculated to be −3.5 to −4.2 kcal mol^–1^ for the S_1_ state, and the 3 to 2.3 kcal mol^–1^ for the T_1_ state for **NBA** and **MV** ^**2+**^, respectively (see the Supporting Information for details).

### Intermediates and Photoproducts

A custom-designed flow photoreactor, in conjunction with UV–vis and HRMS detection,^72,73^ enabled the identification of the species formed during the photoreaction and their temporal evolution (see the Supporting Information for details). Additionally, helium-tagging photodissociation spectroscopy was employed to characterize short-lived reaction intermediates.^74^ In descending order of their abundance, the species observed after irradiating **Cy7** (100 μM) in **EA** buffer (500 mM, pH = 8.7) were as follows: *m*/*z* 383.24 assigned to **Cy5** and *m*/*z* 441.25 identified as an adduct of **Cy7** and molecular oxygen (Figures 3a and S69). The structure of the latter species, the 1′-hydroperoxy **Cy7** derivative **3**, was assigned through a series of HRMS experiments with **Cy7**, **Cy7–2′,6′-** *d*_**2**_, and **Cy7–2′,3′,4′,5′,6′-** *d*_**5**_ (in H_2_O and D_2_O), as well as CID analysis to identify the cleavage site based on the exact masses of the resulting carbonyl fragments. Furthermore, we observed the OH stretching vibration bands at 3504 cm^–1^ in the IRPD spectrum of *m*/*z* 441.25 unambiguously showing the presence of a hydroperoxide rather than an isomeric endoperoxide (Figure S111). Using the same methods, we identified other intermediates with *m*/*z* values of 502.30 and 484.29, which were assigned to an adduct of **Cy7**, **EA**, and O_2_ as the 2′-hydroperoxy-3′-(2-hydroxyethyl)­amino) **Cy7** derivative **4** and the related ketocyanine **5**. The data in Figure 3b, as obtained by HRMS, demonstrate the sequential kinetic profile of the process. Initially, the concentration of **Cy7** (*m*/*z* 409.26) decreased, followed by the formation of **5**, with a very low abundance as expected for such a highly reactive intermediate, which was consumed, and subsequently **Cy5** (*m*/*z* 383.24) was formed. In parallel, the side product **3** was observed and subsequently disappeared, along with the formation of the *m*/*z* 470.28 product. We obtained HRMS, CID, IRPD, and visible-light photodissociation (VisPD)^75–77^ spectra of this ion (Figures S67, S108, and S113). Based on the experimental data, we can conclude that the *m*/*z* 470.28 compound contains two *N*-methyl groups (CID) as well as a carbonyl group and a secondary amine N–H bond (IRPD). Lastly, the moiety is conjugated (VisPD) and rigid, with only one conformer, as demonstrated by the mobilogram in Figure S105. Despite this, we could not determine its structure. The presence of **NBA** in the reaction mixture did not lead to the formation of any new intermediates.

**Figure 3:**
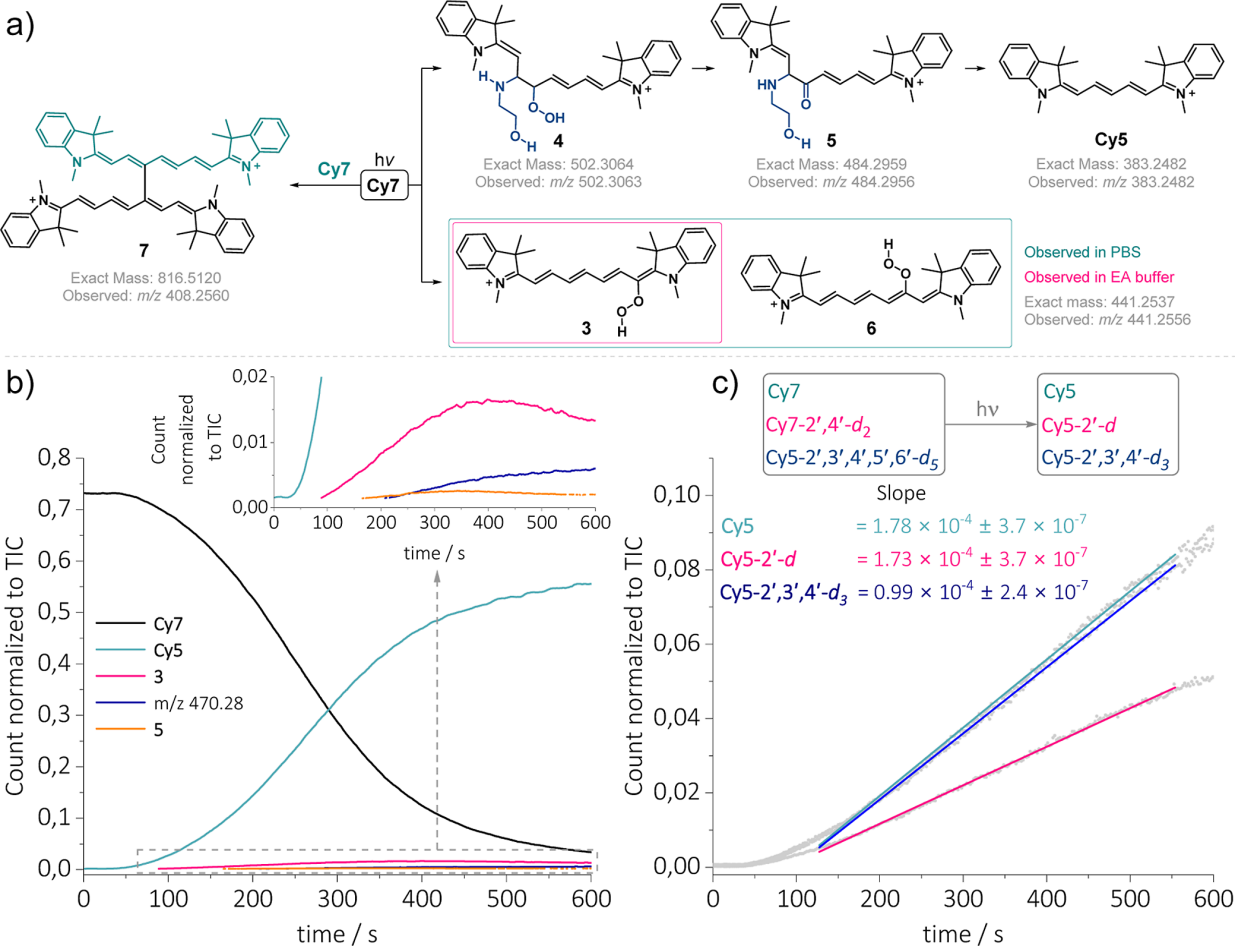
Reaction intermediates, their time evolution, and kinetic isotope effect study. (a) Structures of intermediates and photoproducts confirmed by HRMS, CID, and IRPD. (b) Time traces showing the temporal evolution of 409.26 *m*/*z* (**Cy7**), 383.24 *m*/*z* (**Cy5**), 442.25 *m*/*z* (**3**), 470.28 *m*/*z*, and 486.30 *m*/*z* (**5**) ions, obtained during the irradiation of **Cy7** in an **EA** buffer (500 mM EA, pH = 8.7). The insert depicts a zoomed view of lower-abundance species. (c) The HRMS-KIE experiment involving an equimolar mixture of **Cy7**, **Cy7–2′,6′-** *d*_**2**_, and **Cy7–2′3′4′5′6′-** *d*_**5**_ (33 μM each) in an **EA** buffer (500 mM, pH = 8.7), with the ion count normalized to the total ion current (TIC) vs time traces of **Cy5**, **Cy5–2′-** *d* and **Cy5–2′,3′,4′-** *d*_**3**_ products.

Irradiation of **Cy7** in PBS, a solution that provides only minimal phototruncation, resulted in the formation of a species with a mass of *m*/*z* 441.25 as the primary product. The CID analysis revealed that the ions are at least two hydroperoxy regioisomers: intermediate **3**, previously identified, and its 2′-hydroperoxy analog **6** (Figures 3a, and S96). This compound was identified by HRMS analysis of **Cy7** reacting with thermally generated ^1^O_2_ from **EN** in the dark (Figure S97). Furthermore, a substantial amount of a **Cy7** dimer (**7**, *m*/*z* 408.25) was detected under these conditions. The dimer concentration decreased with increasing **EA** concentration (Figure S73). Previous studies have reported that the **Cy7** dimer forms via reaction of **Cy7** with **Cy7** ^•**+**^, which is produced by **Cy7** photooxidation.^78^

Furthermore, HRMS allowed determination of the kinetics of the formation of **Cy5** isotopomers formed during an experiment with an equimolar mixture of **Cy7**, **Cy7–2′,6′-** *d*_**2**_, and **Cy7–2′,3′,4′,5′,6′-** *d*_**5**_ in an **EA** buffer solution (500 mM at pH 8.7; Figure 3c). The ion counts were normalized to the total ion current (TIC) and corrected for the isotopic distribution (see the Supporting Information). Analysis of the time traces revealed an equal slope for the formation of **Cy5** and **Cy5–2′,6′-** *d*_**2**_. However, a significantly smaller slope, indicating slower formation, was observed for **Cy5–2′,3′,4′-** *d*_**3**_. The slope ratio of 1.7 indicates a primary kinetic isotope effect (PKIE) of the C3′ hydrogen, suggesting that a hydrogen atom at the C3′ position is involved in the rate-determining step (Figures S87–S89).

Next, we assessed the impact of using different wavelengths and excitation intensities. At 750–700 nm, **Cy5** was formed as the primary product. The quantum yield ratio, Φ_**Cy5**form_/Φ_**Cy7**dec_, remained constant within this wavelength range, as well as when irradiation was performed at a lower light intensity (Figures S13–S20, Tables S7 and S8).

At 680–590 nm, where both **Cy5** and **Cy7** absorb, a significant amount of **Cy3** was also produced. Therefore, we examined the mechanism and efficiency of **Cy5** phototruncation under the conditions of this study. As mentioned above, Lee and co-workers showed that the **Cy5** → **Cy3** photoconversion occurs via an intermolecular cross-coupling process.^30^ The irradiation of **Cy5** in an **EA** buffer (**EA** = 500 mM, pH = 8.7, 2 mM **NBA**; 625 nm LEDs) resulted in the formation of **Cy3**, both without and with a singlet oxygen trap (NaN_3_; 2 mM). The maximum yields of **Cy3** were determined to be 4% and 5%, respectively, as calculated from the absorption spectra (Figures S22 and S23). These results were validated by HPLC measurements. Analogous to the reaction of **Cy7**, it was concluded that the trapping of singlet oxygen led to a slight decrease in the unproductive **Cy5** decomposition pathway. To study the phototruncation mechanism of **Cy5**, a similar crossover experiment to that with **Cy7**, shown in Scheme 3, was performed with an equimolar mixture of **Cy5** and **Cy5-** *d*_**6**_ (dimethyl-*d*_6_). Only **Cy3** and **Cy3-** *d*_**6**_ products were formed, indicating that the primary **Cy5** phototruncation pathway is also intramolecular (Figure S99). The **Cy5** → **Cy3** truncation reaction by thermally produced ^1^O_2_ by 1-methylnaphthalene-4-propionate endoperoxide^29^ in the absence of light was inefficient (∼1%) under the same study conditions used for the **Cy7** → **Cy5** conversion (Table 2).

### Mechanism

Here, we present a summary of the key experimental findings that have contributed to our understanding of the mechanism of **Cy7** phototruncation:

- Both the singlet and triplet excited states are the productive states. Triplet quenching does not halt the reaction, but triplet sensitization results in phototruncation.
- The 2-aminoethan-1-ol motif of the additive/buffer plays an essential role in the process.
- The reaction requires an optimal pH level and a nonviscous water-based medium.
- Molecular oxygen is essential for the reaction and serves as a photooxidant.
- The elimination of the C2′–C3′ chain fragment was identified.
- The identified reaction intermediates are adducts of oxygen and/or 2-aminoethanol.
- No reactive oxygen species (^1^O_2_, HO^**•**^, O_2_^**•**–^) are responsible for producing **Cy5**; O_2_^**•**–^ is produced in the initial oxidation step.
- Electron acceptors significantly increase the yield of **Cy5.**
- Electron transfer occurs in the primary photochemical step, most probably via the singlet excited state.
- Electron abstraction from the ground-state **Cy7** also results in truncation.
- Chain 3′-hydrogen is cleaved in the rate-determining step.

A substantial amount of data and experimental findings allowed us to propose a mechanism for the phototruncation of **Cy7** in the presence of a specific reagent, **EA** (Scheme 5a). In the absence of direct involvement of the common reactive oxygen species (^1^O_2_, O_2_^**•**–^, HO^**•**^) in the truncation process, and given the observation of the formation of O_2_^**•**–^ by FSR spectroscopy at ultrafast time scales (<75 fs),^64^ it is proposed that an initial photochemical step involves electron transfer from the singlet excited state to the ground-state oxygen (Scheme 5), step (i). ET faster than diffusion indicates the formation of a ground-state complex. To test this hypothesis, we measured the UV–vis spectra of **Cy7** in the presence and absence of O_2_ (Figure S30). The appearance of a low-intensity, broad band in the presence of O_2_ was consistent with previous studies of ground-state complexes of molecular oxygen and organic molecules.^79,80^ Upon excitation of the **Cy7**–O_2_ ground-state complex, electron transfer leads to the formation of a radical ion pair. The PET processes in cyanine dyes have been well-documented.^81–84^ This is further substantiated by reports on the EPR characterization of the O_2_^**•**–^ spin trap adducts observed upon the irradiation of cyanines.^65,66,85^ The unique properties of water molecules^86,87^ enable the solvation and rapid separation of the primary radical–ion pair,^88^ thus preventing back ET,^89^ and facilitate rapid proton transfer through the hydrogen bonding network.^88,90^ This may explain why phototruncation occurs only in aqueous media or is less efficient in mixtures of water and polar protic solvents, such as methanol (see above; Figure S28). Furthermore, it is well established that cyanine dyes tend to self-aggregate in aqueous media.^91,92^ Upon the irradiation, these dyes undergo interaggregate ET, forming radical dication and radical anion pair.^93,94^ However, our spectroscopic experiments at various concentrations and optical path lengths showed that the extent of aggregation is minimal (Figure S29). Therefore, we concluded that, under our conditions, the excitation of the aggregated **Cy7** can, at best, play only a minor role in phototruncation.

**Scheme 5:**
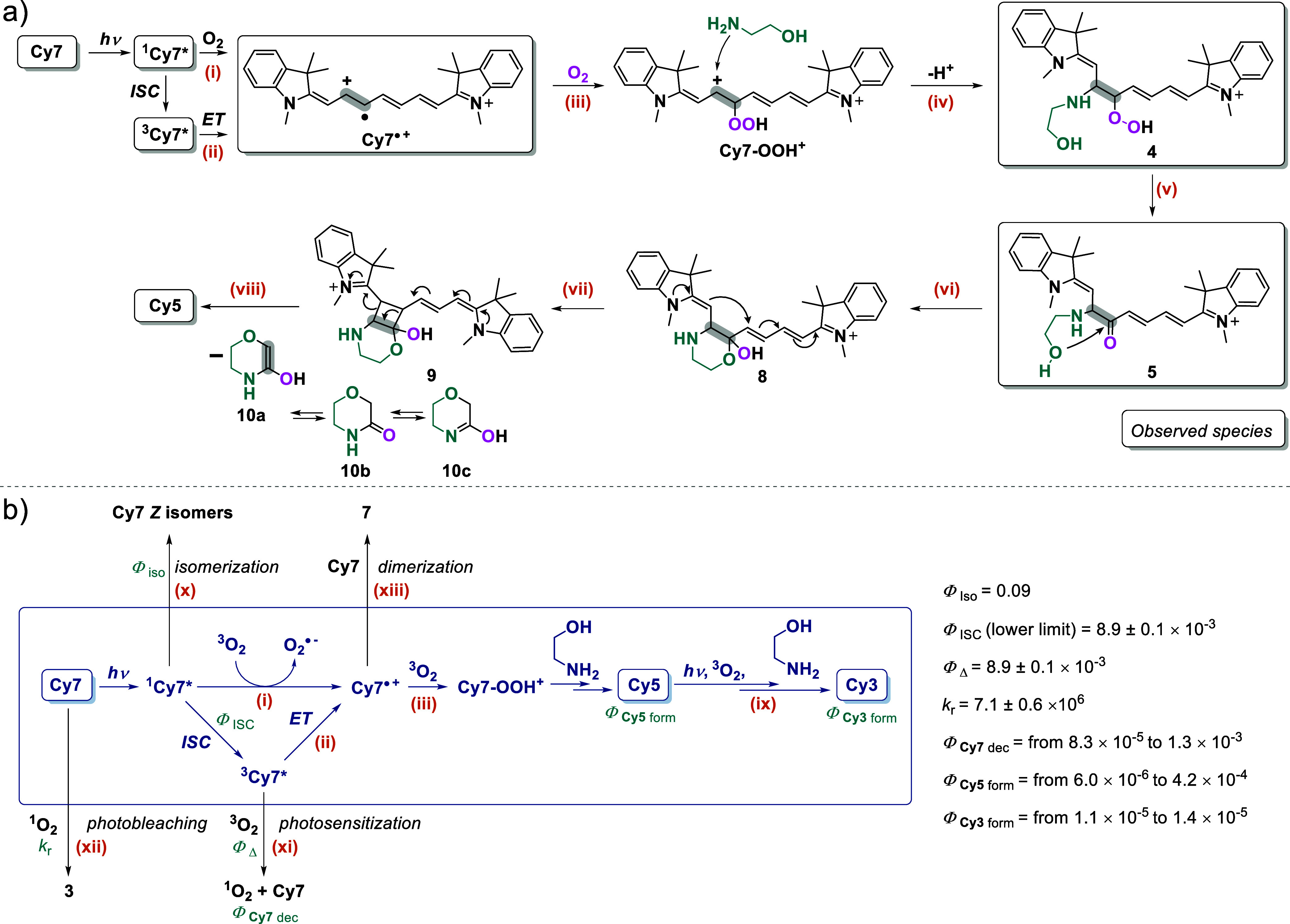
Proposed Phototruncation Mechanism of Cy7^a^

a The mechanism of **Cy7** phototruncation depicting the observed and assumed intermediates and side-products. (b) Productive and nonproductive pathways after **Cy7** irradiation: The quantum yield of ^1^O_2_ production (Φ_Δ_), the rate constant for the reaction of **Cy7** with ^1^O_2_ (*k*_r_),^5^ the ISC quantum yield (Φ_ISC_; the lower limit of which was determined by the singlet oxygen production),^5^ and the photoisomerization quantum yield (Φ_iso_)^105^ were obtained from the literature. The limiting values of the quantum yields of **Cy7** decomposition (Φ_**Cy7**dec_) and **Cy5** (Φ_**Cy5**form_) formation are discussed in the Supporting Information. Please note that the values provided are for illustrative purposes only and represent measurements taken under different experimental conditions. For a comprehensive overview of the experiments conducted for this study and the conclusions derived from them, please refer to Table S15.

The **Cy7** ^**•+**^ species formed in the step (i) has a short lifetime (τ = 276 ± 50 ps)^64^ despite the anticipated persistence resulting from resonance stabilization of the extended polymethine chain.^95,96^ The radical center of cyanine radical dications is presumably localized on the odd carbon atoms.^49^ The formation of **Cy7** ^**•+**^ directly connected to the truncation was confirmed through the analysis of the **CQ** photooxidation experiment (Table 4). The significant increase in the absolute values of Φ_**Cy7**dec_ and Φ_**Cy5**form_, as well as the increase in the Φ_**Cy5**form_/Φ_**Cy7**dec_ ratio, together with the calculated exergonicity of the electron transfer, suggested that the T_1_ state also undergoes ET to electron acceptors, including O_2_ (Scheme 5), step (ii). Moreover, the participation of the cyanine T_1_ state in ET has already been observed for other cyanine derivatives.^97^ This means that both S_1_ and T_1_ pathways share the same intermediate (**Cy7** ^**•+**^).

It is well established that radical cations react with molecular oxygen at the radical site.^98,99^ Here, we propose that the initial reaction be the reaction of **Cy7** ^**•+**^ with O_2_, which results in the formation of 3-hydroperoxycation (**Cy7** ^**+**^ **-OOH**), step (iii). Indeed, the HRMS experiments using O^18^-labeled water supported this presumption as the attack by O_2_ was more efficient than the nucleophilic attack by water (Figure S62). Furthermore, the proposed **Cy7** ^**+**^ **-OOH** structure most effectively accommodates two positive charges and a conjugation break. In the next step, **Cy7** ^**+**^ **-OOH** reacts with **EA** as a nucleophile, which is the first intermediate identified by HRMS/CID (*m*/*z* 502.30); step (iv). The reported diffusion-controlled nucleophilic attack of a primary amine on radical cations is related to the higher nucleophilicity of the amino group versus the hydroxy group of **EA**.^100,101^ Conversely, hydroxy nucleophiles are considered to be poorer nucleophiles.^102^ Under our conditions, the amino group of **EA** attacks **Cy7** ^**+**^ **-OOH** to form **4** first, as evidenced by HRMS and CID analyses (Figures S70 and S71). The temperature and viscosity (Figure 1) would surely affect this bimolecular step. The nucleophilic attack on the C2′ carbon is underscored by analogous, thiol-**Cy7** adducts, proposed by Zhuang and co-workers, in which a sulfur–carbon bond was demonstrated to form with the C2′ carbon of the **Cy7**.^103^ The rate-determining step, as indicated by the KIE (Figure S89), is the abstraction of a hydrogen atom from the C3′ atom. This occurs during the oxidative transformation of the peroxy group to the carbonyl group, for which the intermediate **5** (*m*/*z* 484.29), step (v) was identified. The existence of **5** is substantiated by the literature reports of several ketocyanine derivatives.^104^

Due to the lack of direct evidence of the existence of the intermediate(s) prior to the formation of **Cy5**, we propose the final steps, inspired by the work of Schnermann et al.,^29^ who anticipated the formation of a small-ring (cyclobutane) intermediate prior to the elimination step (Scheme 2a). Such highly reactive species must be short-lived, so it was not surprising that we could not detect them using any of the analytical methods. Here, we hypothesize that the hydroxyl group attacks the newly formed carbonyl group via an intramolecular nucleophilic process in **5**, leading to the formation of the substituted morpholine intermediate (**8**, step vi). This is followed by a rearrangement to afford intermediate **9** (step vii), and finally, retro 2 + 2 cycloaddition with elimination of 3,4-dihydro-2*H*-1,4-oxazin-5-ol (**10a**; step viii) provides the final **Cy5** derivative. This compound is an enol form of morpholin-3-one (**10b**), which at higher pH should be in equilibrium with a different enol form **10c**. Despite strenuous efforts, the detection of **10a** in irradiated reaction mixtures remained elusive. The Supporting Information describes the methods used to confirm the proposed final steps of the mechanism (Chapter 10.9). This part of the mechanism is still conjectural and may be resolved in a future study.

In addition to **Cy7** → **Cy5** phototruncation, Scheme 5b illustrates **Cy5** → **Cy3** phototruncation (step ix) and the pathways leading to the nonproductive photodegradation of **Cy7** that have been studied to date and partially discussed in this paper. The scheme also depicts some kinetic and other photochemical data.

Following excitation to the excited singlet state, the primary process is configurational photoisomerization, which results in *Z*-isomers. In our recent study, two of the four possible photoisomers formed from the S_1_ (step x) and T_1_ states were identified.^64^ The photoisomerization quantum yields were found to be considerably higher (Φ_iso_ = 0.09)^105^ than those of phototruncation. Therefore, this channel is identified as one of the dominant deexcitation pathways. The T_1_ state of **Cy7** is also responsible for the formation of ^1^O_2_ via photosensitization (step xi) and consequently for the formation of the 1′-hydroperoxy radical (**3**, step xii; Figure 3) and thus lower the phototruncation chemical yield.

The **Cy7** ^**•+**^, a typical π-type radical, readily undergoes coupling with another **Cy7** molecule, leading to the formation of a dimer.^78,106^ Indeed, this process was observed, but it was less efficient in the presence of **EA** (step xiii; Figure S73). Spectroelectrochemical investigations demonstrated the enhanced persistence of cyanine radical dications for cyanines with bulky substituents on the chain.^95^ Some of the reported radical cations exhibit sufficient lifetimes to allow UV–vis and EPR characterization, and undergo dimerization.^107^

We do not exclude the possibility that phototruncation of **Cy7**, a process that occurs in low yields (<1%) in aqueous buffers that do not contain the **EA** scaffold, occurs via the mechanism suggested by Schnermann, Sauer, Greer, and co-workers,^29,35^ although the radical cation pathway should still remain a viable option.

## Conclusions

Through extensive experimental investigations, we uncovered the phototruncation mechanism of prototypical heptamethine cyanine, which is converted to pentamethine cyanine. We demonstrate that this reaction is highly selective and sensitive to reaction conditions, such as the presence of water as a solvent, pH level, concentration, and buffer constituents. The first reaction step is an electron transfer from the singlet- or triplet-excited **Cy7** to oxygen. This step leads to a superoxide radical anion and a cyanine radical dication intermediate. Subsequent steps involve the latter species reacting with another oxygen molecule and a specific buffer component featuring an ethanolamine scaffold. Subsequent oxidation, cyclization, and elimination steps lead to the formation of phototruncated **Cy5** and a side-product containing the 2′ and 3′ chain carbons of the initial **Cy7** molecule. A series of quenching, trapping, and state-of-the-art spectroscopic and mass spectrometric experiments helped us to determine the roles of reaction intermediates, reactive oxygen species, and solution components in productive and unproductive pathways. Additionally, understanding the detailed mechanism enabled us to significantly improve reaction yields by employing water-soluble electron acceptor additives. The cyanine radical cation, which is formed when electrons are transferred from the excited **Cy7** to oxygen, is a key intermediate that can also explain the photooxidative dealkylation^108^ mechanism of cationic dyes.

Our experimental findings provide a fresh perspective on the phototruncation mechanism and pave the way for more effective cyanine-based in vivo imaging and sensing strategies. Furthermore, we emphasize in this work that thorough experimentation remains essential when investigating complex photochemical reaction mechanisms.

## Supplementary Material



## Notes

Materials and methods, synthesis, quantum yield measurements, UV–vis, NMR, fs-TA, FSR, HRMS, CID, IRPD and VisPD spectra, and additional experimental details (PDF)
