# Supplementary material for: Experimental Study Redefines the Mechanism of Heptamethine Cyanine Phototruncation
Source: J Am Chem Soc. 2026 Mar 10;148(11):11595–608. doi: 10.1021/jacs.5c18903 (PMC13022889; doi:10.1021/jacs.5c18903)
Supplement: Supplementary file 1 [file ja5c18903_si_001.pdf]

Supporting Information for

Experimental Study Redefines the Mechanism of Heptamethine Cyanine Phototruncation

Nasrulla Majid Khan,[1,2,§] Gabriel Glotz,[2,§] Jana Okoročenkova,[1,2] Jakub Dostál,[3] Miroslav Kloz,[3] Max T. G. M. Derks,[4] Aleksandr Y. Pereverzev,[4] Dmytro Neshchadin,[5] Jana Roithová,[4]* and Petr Klán[1,2]*

[1] Department of Chemistry, Faculty of Science, Masaryk University, 62500 Brno, Czech Republic
[2] RECETOX, Faculty of Science, Masaryk University, 62500 Brno, Czech Republic
[3] The Extreme Light Infrastructure Facility ERIC, Za Radnicí 835, 25241 Dolní Břežany, Czech Republic
[4] Institute for Molecules and Materials, Faculty of Science, Radboud University, Heyendaalseweg 135, Nijmegen 6525 AJ, Netherlands
[5] Institute of Physical and Theoretical Chemistry, Graz University of Technology, Stremayrgasse 9, 8010 Graz, Austria

[§] These authors contributed equally to this work.

Contents

1. General information S3
   1.1. Materials and sample preparation S3
   1.2. Methods S3
   1.3. HPLC analysis S3
2. Irradiation experiments S5
   2.1. Experimental setup S5
   2.2. Data from irradiation experiments S5
      2.2.1. Quenching or traping of reactive oxygen species and radical species S6
      2.2.2. Triplet sensitization and quenching experiments S9
      2.2.3. Photooxidation by camphorquinone (**CQ**) S14
      2.2.4. Impact of varying light intensity and irradiation wavelength S15
      2.2.5. Irradiation of **Cy5** S19
      2.2.6. Other irradiation experiments S21
3. UVvis measurements S25
   3.1. UVvis of **Cy7** in **EA** buffer at different concentrations S25
   3.2. UVVis spectra of **Cy7**-O_2 ground state complex in **EA** buffer S26
4. Quantum yield measurements S27
   4.1. Experimental setup S27
   4.2. Theory S27
   4.3. Quantum yields data S30
      4.3.1. Quantum yields at varying light intensity and irradiation wavelengths S33
      4.3.2. Quantum yields for irradiation of **Cy5** S35
5. Femtosecond transient absorption spectroscopy (fs-TA) S36
   5.1. fs-TA data analysis S36
   5.2. fs-TA-data S36
6. Femtosecond stimulated Raman spectroscopy (FSR spectroscopy) S41
   6.1. Target analysis S41
   6.2. FSR spectroscopy data S42
   6.3. Superoxide radical anion S46
7. Cyclic voltammetry S48
   7.1. Redox potentials analysis S49
8. Overview of the experimental results S50
9. A flow photoreactor S51
10. High resolution mass spectrometry - trapped ion mobility spectrometry S53
   10.1. HRMS of **Cy7** and derivatives in **EA** buffer S54
   10.2. Crossover experiment S67
   10.3. The kinetic isotope effect S68
   10.4. HRMS of **Cy7** + N-acetylcysteine S70
   10.5. HRMS of **Cy7** irradiated in PBS buffer S73
   10.6. HRMS of **Cy7** and derivatives with endoperoxide S74
   10.7. Crossover experiment with **Cy5** S76
   10.8. Mobilograms of selected ions S77
   10.9. HRMS Detection of **10a** S79
11. Helium tagging photodissociation spectroscopy S80
   11.1. Visible photodissociation spectroscopy (VisPD) S80
   11.2. Infrared photodissociation Spectroscopy (IRPD) S81
12. Synthesis of cyanine derivatives S85
   12.1. Synthesis of **Cy7-3',5'-d$_2$** S85
   12.2. Synthesis of **Cy7-*d*$_6$** (dimethyl-*d*$_6$) S86
   12.3. NMR spectra S87
13. References S95

1. General information

1.1. Materials and sample preparation

Solvents and reagents of the highest purity available were used as purchased. Ethanolamine (**EA**) was distilled under reduced pressure and stored under argon in a vial, protected from light. Deionized water used for the preparation of buffers was obtained using a Milli-Q® system.

Heptamethine cyanine derivatives: All cyanine derivatives were synthesized and purified according to our previously published work from the corresponding pyridine derivatives via the Zincke reaction.[1] The pentadeuteriated (**Cy7-2′,3′,4′,5′,6′-d_2**), **Cy7** di-(**Cy7-2′,6′-d_2**), and di-(**Cy7-3′,5′-d_2**) derivatives were prepared from commercially available pyridine-d_5 or from pyridine-2,6-d_2 or pyridine-3,5-d_2. The latter two were prepared from commercially available 2,6-dibromopyridine and its 3,5-dibromo derivative, respectively, according to our previously published work[2-3] (details in the synthesis part on page S85). The analytical data of the deuterated cyanine derivatives were in line with the literature values.[4] The hexadeuteriated (**Cy7-d_6** dimethyl-d_6) was prepared from 2,3,3-trimethyl-1-(methyl-d_3)-3H-indol-1-ium iodide via the Zincke reaction (details in the synthesis part, page S85).

Sample purification: Flash column chromatography was performed using silica gel (230−400 mesh).

Preparation of buffers:

EA buffer: A 1.52 mL of **EA** was dissolved in 35.0 mL of Milli-Q water, followed by adjusting the pH of the solution with Acetic acid (8 M). The final volume of the mixture was adjusted to 50 mL using Milli-Q water; thus, the obtained 0.5 M solution was then used for experiments. Similarly, 0.02, 0.1, 0.3, 0.7, and 1.0 M **EA** buffer solutions were prepared by dissolving 0.06, 0.30, 0.92, 2.13, and 3.05 mL of **EA** in 35 mL of Milli-Q water; pH and volume were adjusted accordingly.

PBS buffer: One tablet of phosphate buffered saline (PBS; P4417, purchased from Sigma) was dissolved in 200 mL of deionized water to yield 0.01 M phosphate buffer, 0.0027 M potassium chloride, and 0.137 M sodium chloride, pH 7.4, at 25 °C.

Britton-Robinson buffer: A mixture of 0.04 M boric acid, 0.04 M phosphoric acid, and 0.04 M acetic acid was titrated to the desired pH with 1 M sodium hydroxide.

Sample preparation: A 50 μM and 10 μM of **Cy7** in the buffer of interest was obtained from 5 mM or 1 mM dye stock solutions in DMSO, respectively.

1.2. Methods

The UV-Vis spectroscopy: UV-Vis spectra were acquired either using a Specord S600 from Analytikjena or a Perkin Elmer Lambda 5 spectrometer.

The NMR spectroscopy: ^{1}H NMR spectra were recorded on a 500 MHz spectrometer; ^{13}C NMR spectra were obtained on a 125 MHz instrument in DMSO-d_6 or CD_3OD. ^{1}H chemical shifts are reported in ppm relative to tetramethylsilane (δ = 0.00 ppm) using the residual solvent signal as an internal reference. ^{13}C chemical shifts are reported in ppm with DMSO-d_6 (δ = 39.52 ppm) and CD_3OD-d_4 (δ = 49.00 ppm) as internal references. The deuterated solvents were kept under a nitrogen atmosphere.

Mass spectrometry: High resolution mass spectra (HRMS) were acquired on an Agilent 6224 Accurate-Mass TOF LC-MS with a dual electrospray/chemical ionization mode or a Bruker TIMS-TOF Classic instrument with an ESI source.

1.3. HPLC analysis

HPLC analyses were performed on an Agilent 1260 Infinity II HPLC system with a Zorbax SB-Aq C18 column (3.5 µm, 4.6 × 150 mm) and a photodiode array detector (200–900 nm) at 30 °C with a flow rate of 1 mL min^{-1}. Two mobile phases consisted of 0.1% trifluoroacetic acid in UHPLC-grade water (A) and HPLC-grade acetonitrile (B), with the following gradient: 10% to 90% of B over 6 min, 90% to 95% of B over 0.5 min, 95% B for 0.5 min, and re-equilibrated with 10% solution B for 3 min.

Figure S1 shows a typical chromatogram obtained during irradiation experiments (see below). The quantities of **Cy7** and **Cy5** were determined from the peak area using the calibration obtained with analytical pure **Cy7** (Figure S1a) and **Cy5** (Figure S1c) samples.

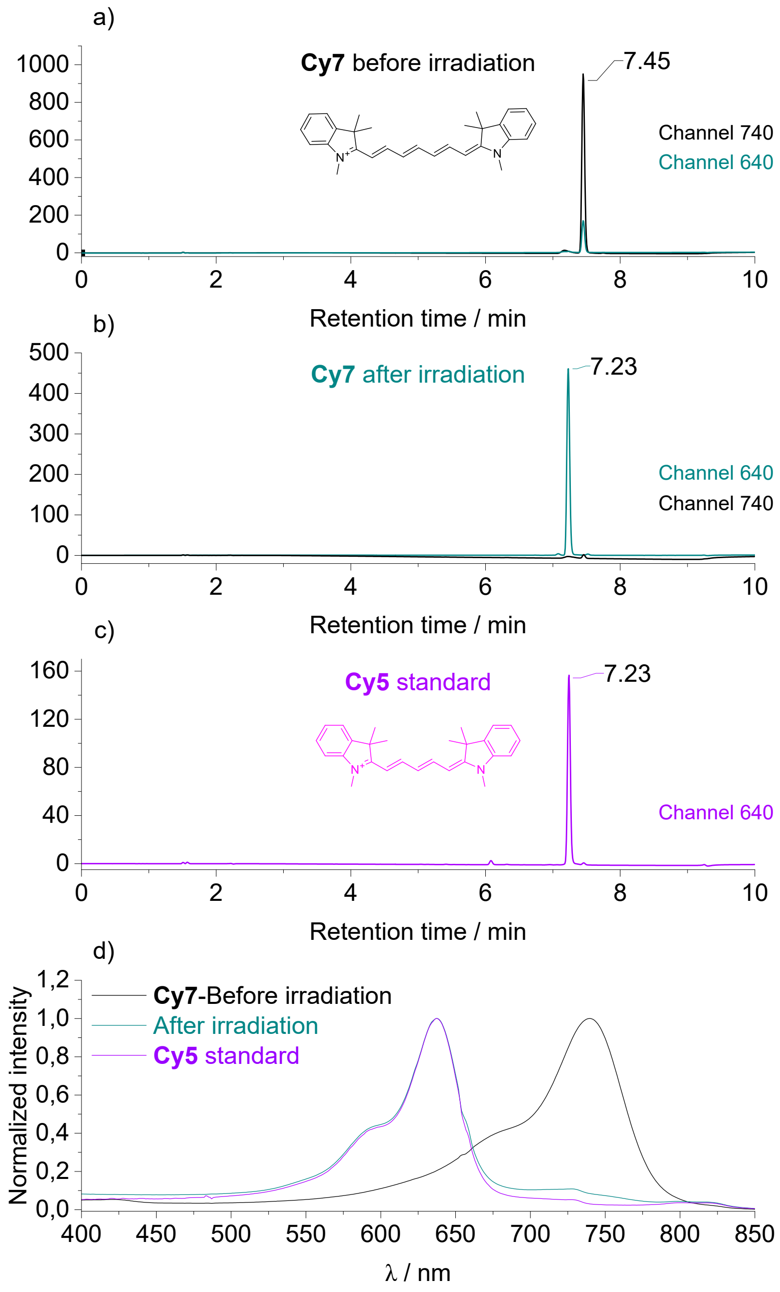

Figure S1. HPLC Chromatograms: a) **Cy7** before irradiation, channel: 740 nm, retention time of 7.45 min, b) **Cy7** after irradiation, Channel 640 nm, retention time of 7.23 min corresponds to the analytical standard of **Cy5**, c) **Cy5** analytical standard, retention time of 7.23, channel: 640 nm. d) Absorption spectra of an HPLC peak at 7.45 min correspond to (**Cy7** before irradiation), 7.23 min correspond to after irradiation of **Cy7**, and 7.23 min correspond to **Cy5** analytical standard.

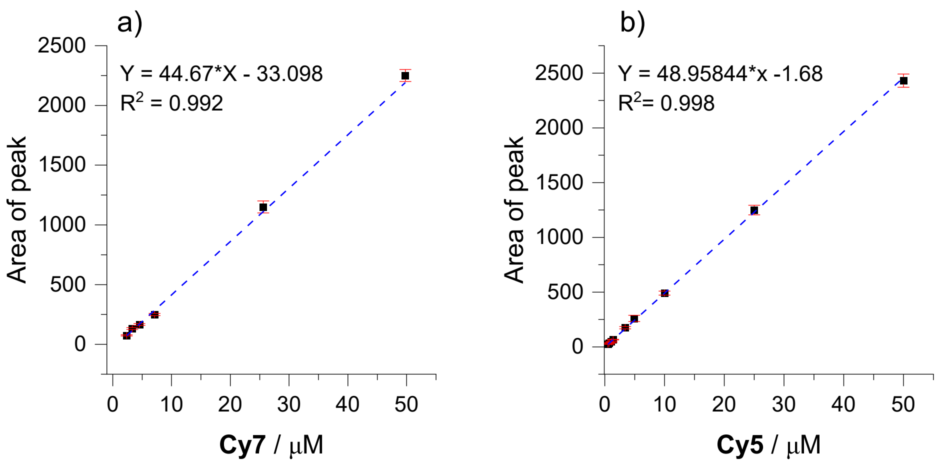

Figure S2. The HPLC calibration curves for a) **Cy7** and b) **Cy5**.

2. Irradiation Experiments

2.1. Experimental setup

Irradiation experiments – UV-Vis monitoring: The irradiation experiments were performed in a custom-built setup, using a homemade photoreactor mounted in a Perkin Elmer Lambda 5 spectrometer's beam path. This custom built photoreactor features two rows of light-emitting diodes (LEDs) perpendicular to the spectrometer beam path, driven by the constant current source, integrated cooling fan and a stirring pad with adjustable speed (Figure S3). All irradiation experiments were performed at 22 ± 1˚C in a cuvette under aerated conditions with LEDs with emission maxima centered at 735 nm, unless stated otherwise.

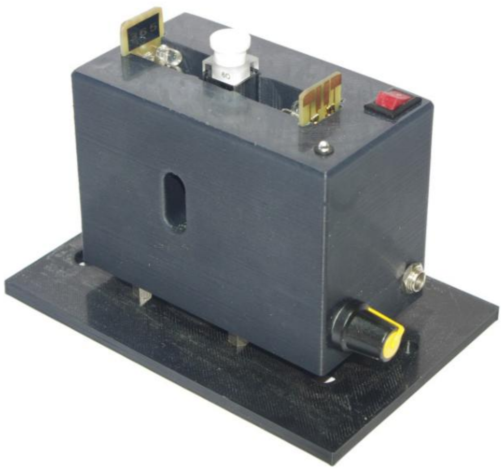

Figure S3. A custom-made 3D printed LED reactor (with two plug-in LED modules, 14 low-power LEDs per module, an integrated cooling fan, and a stirring pad with adjustable speed) used for simultaneous irradiation and UV-Vis measurements of the samples in 1.0 cm cuvettes.

2.2. Data from Irradiation experiments

The data from the irradiation experiments are divided into the following sections:

- Quenching or traping of reactive oxygen species and radical species
- Triplet sensitization and quenching experiments
- Photooxidation by camphorquinone
- Other irradiation experiments

2.2.1. Quenching or traping of reactive oxygen species and radical species

Several quenchers and traps have been employed depending on the nature of the reactive oxygen species under consideration. For singlet oxygen, sodium azide, DABCO, and furfuryl alcohol were used (Figure S4). For hydroxy radicals, superoxide dismutase, *t*-butyl alcohol, and DMPO were used (Figure S5), while ascorbic acid, *N*-acetylcysteine, and glutathione were tested as radical scavengers (Figure S6).

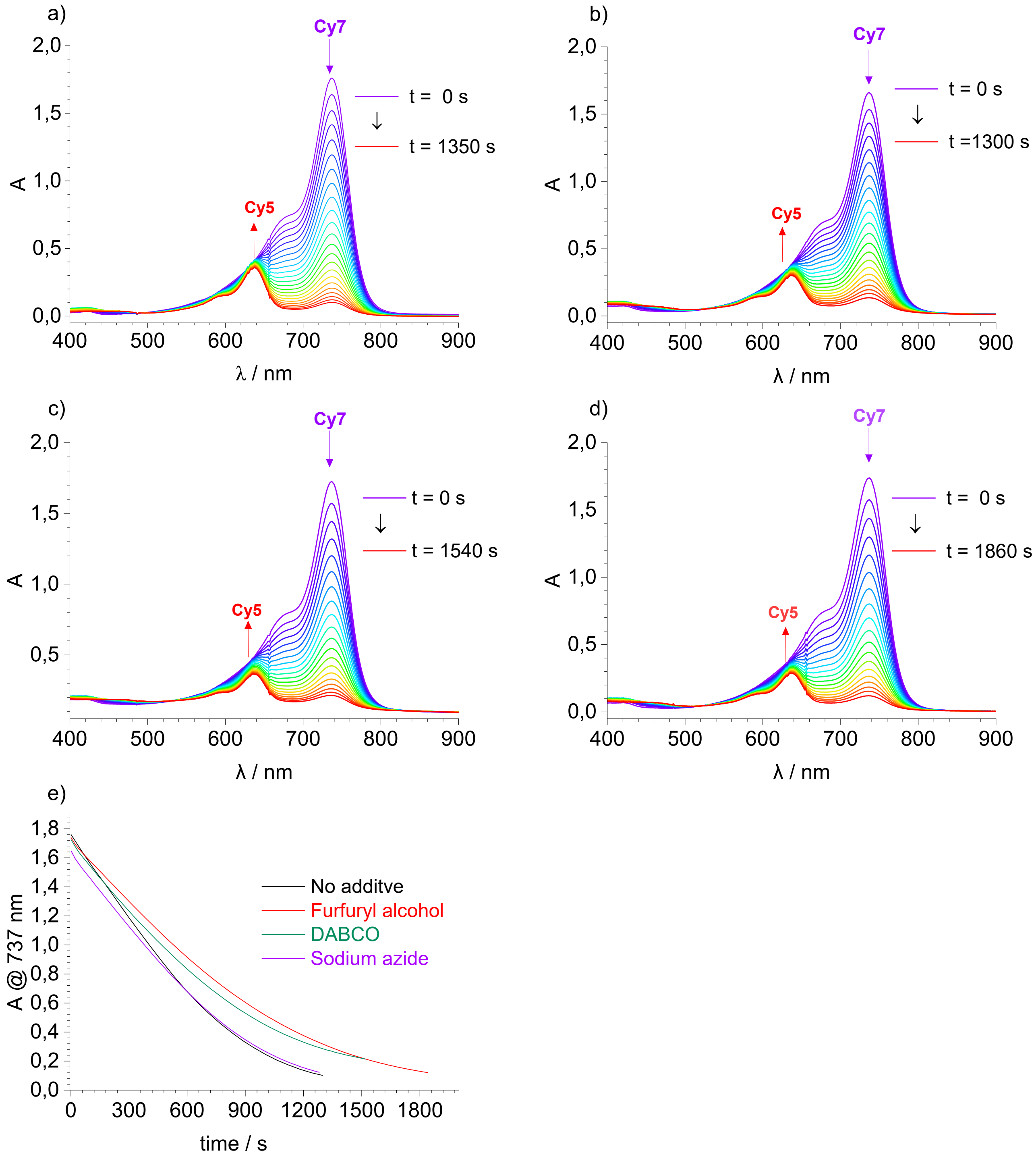

Figure S4. Experimental UV-Vis spectra vs. time for the irradiation of **Cy7** (10 μM) in **EA** buffer (500 mM, pH = 8.7) with a) no additives, b) sodium azide (10 mM), c) DABCO (10 mM), and d) furfuryl alcohol (10 mM), and e) absorbance at 737 nm (λ_{max} of **Cy7**) vs. time for a) to d) experiments.

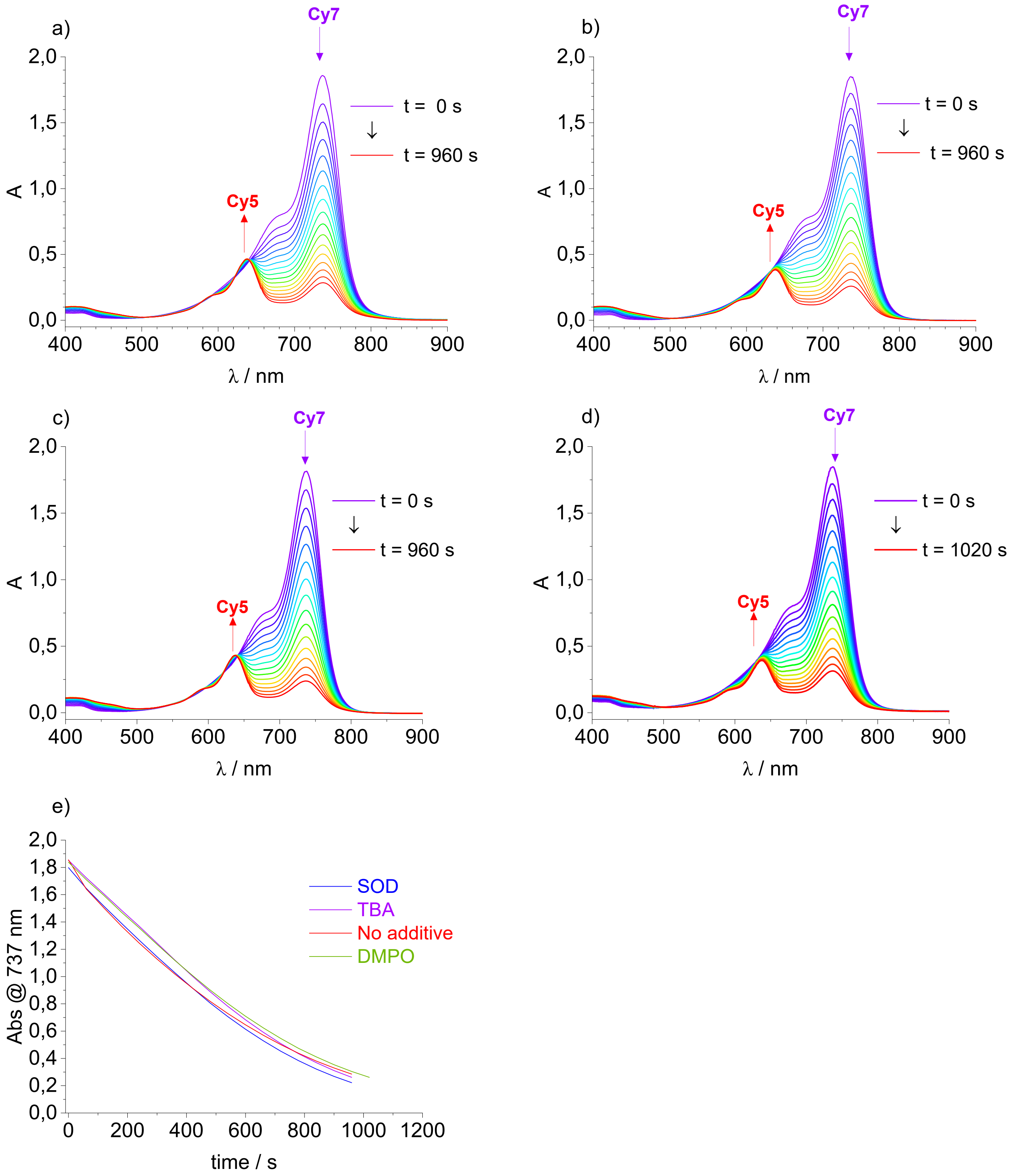

Figure S5. Experimental UV-Vis spectra vs. time for the irradiation of **Cy7** (10 μM) in **EA** buffer (500 mM, pH = 8.7) with a) no additives, b) superoxide dismutase (300 U/mL), c) *t*-butyl alcohol (5 mM), and d) 5,5-dimethyl-1-pyrroline-*N*-oxide (DMPO; 5 mM), and e) absorbance at 737 nm (λ_{max} of **Cy7**) vs. time for a) to d) experiments.

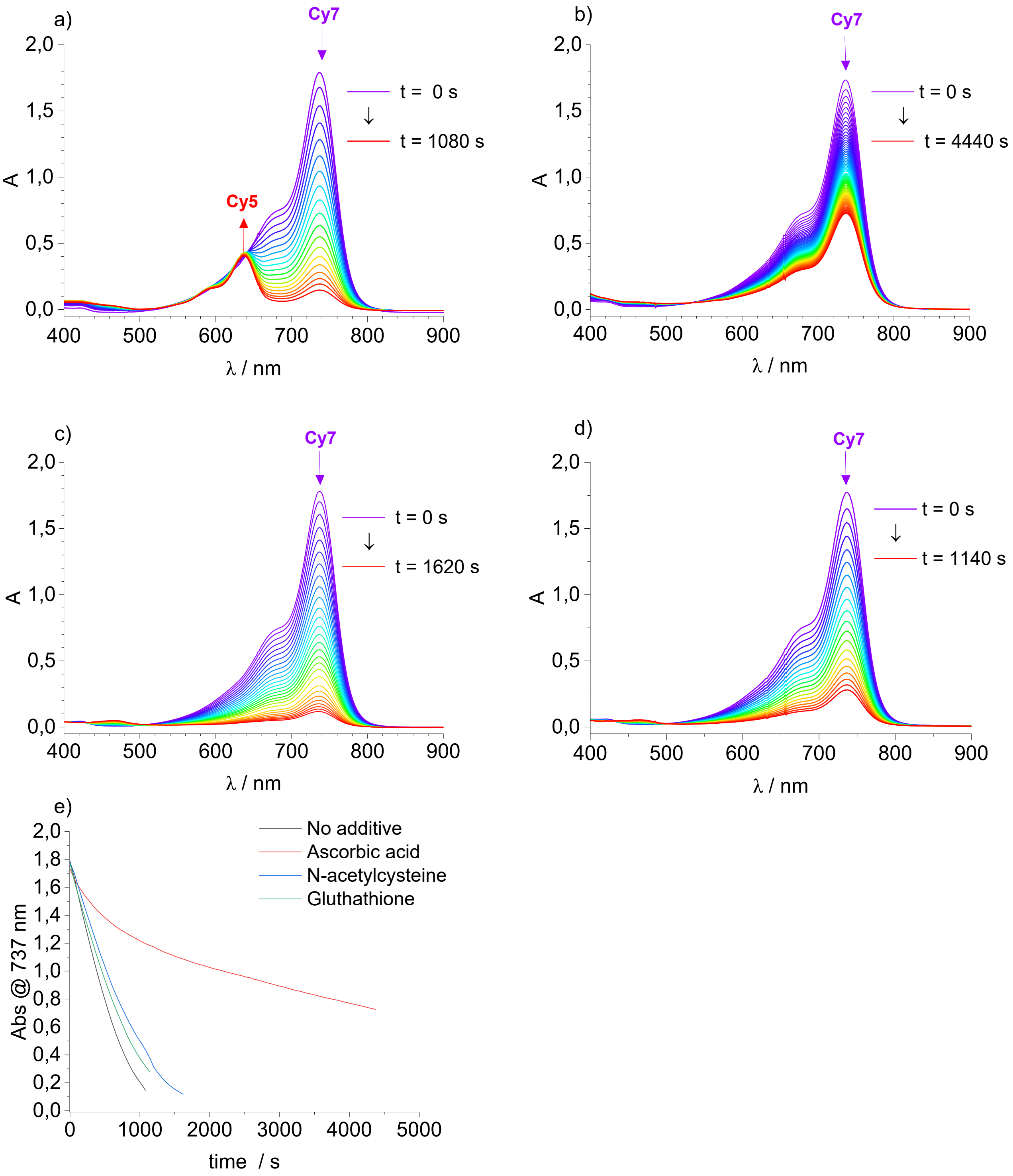

Figure S6. Experimental UV-Vis spectra vs. time for the irradiation of **Cy7** (10 μM) in **EA** buffer (500 mM, pH = 8.7) with a) no additive, b) ascorbic acid (2 mM), c) *N*-acetylcysteine (2 mM), and d) glutathione (2 mM), and e) absorbance at 737 nm (λ_{max} of **Cy7**) vs. time for a) to d) experiments.

2.2.2. *Triplet sensitization and quenching experiments*

Several approaches were used to investigate the role of the triplet state in phototruncation. Initially, we increased the population of triplet state by sensitization using KI as an external heavy atom effect agent (Figure S7) or triplet sensitizers, such as anthracene or anthracene-9,10-dipropanoic acid disodium salt (Figure S8). The second approach was to use triplet quenchers. However, a common **Cy7** triplet quencher, cycloocta-1,3,5,7-tetraene (**COT**), is not soluble under our conditions. Therefore, we synthesized a water-soluble version – cycloocta-1,3,5,7-tetraene-1-carboxylic acid (**COT-COOH**)[5] – and performed irradiation experiments. We observed no decrease in phototruncation (Figure S9). To demonstrate that **COT-COOH** indeed quenches T_1, we performed irradiation experiments in methanol. Irradiation of **Cy7** in MeOH resulted in photobleaching of **Cy7** due to the reaction with singlet oxygen, produced via sensitization of molecular oxygen with the **Cy7** triplet state. Therefore, if **COT-COOH** quenched the T_1 state, the rate of **Cy7** decomposition in methanol would decrease upon its presence, as it was observed (Figure S10). To ensure that **COT-COOH** does not simply scavenge singlet oxygen, we added sodium azide, and observed the same **Cy7** photobleaching rate as without sodium azide (Figure S10c), conclusively showing that **COT-COOH** quenched the triplet. Furthermore, we tested other water soluble T_1 quenchers, such as 4-nitrobenzyl alcohol, methyl viologen, nitro blue tetrazolium, and sodium nitrate (Figure S11), which resulted in increased phototruncation (see main text).

Triplet sensitization

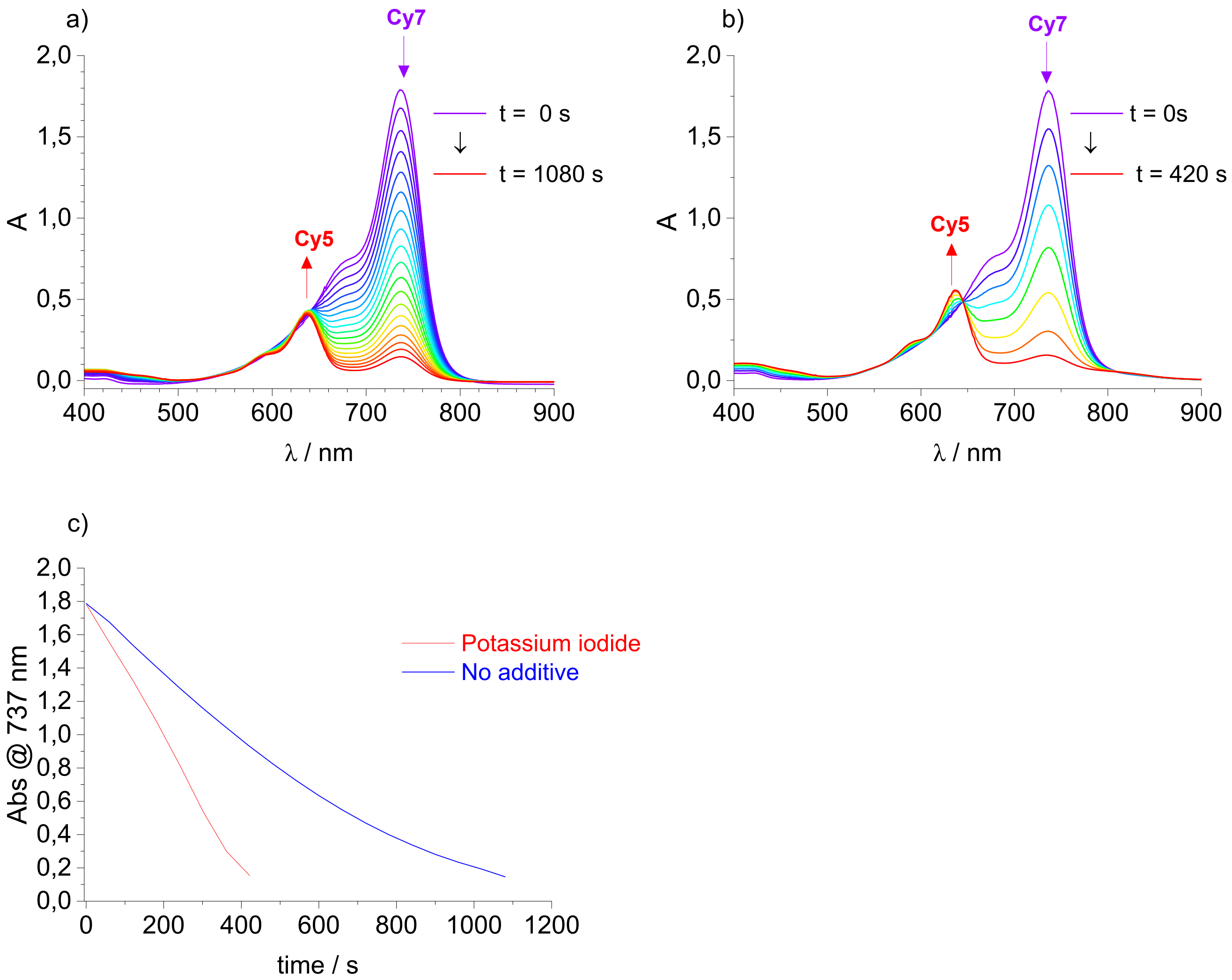

Figure S7. Experimental UV-Vis spectra vs. time for the irradiation of **Cy7** (10 μM) in **EA** buffer (500 mM, pH = 8.7) with a) no additive and b) addition of KI (5 mM), and c) absorbance at 737 nm (λ_{max} of **Cy7**) vs. time for a) and b) experiments.

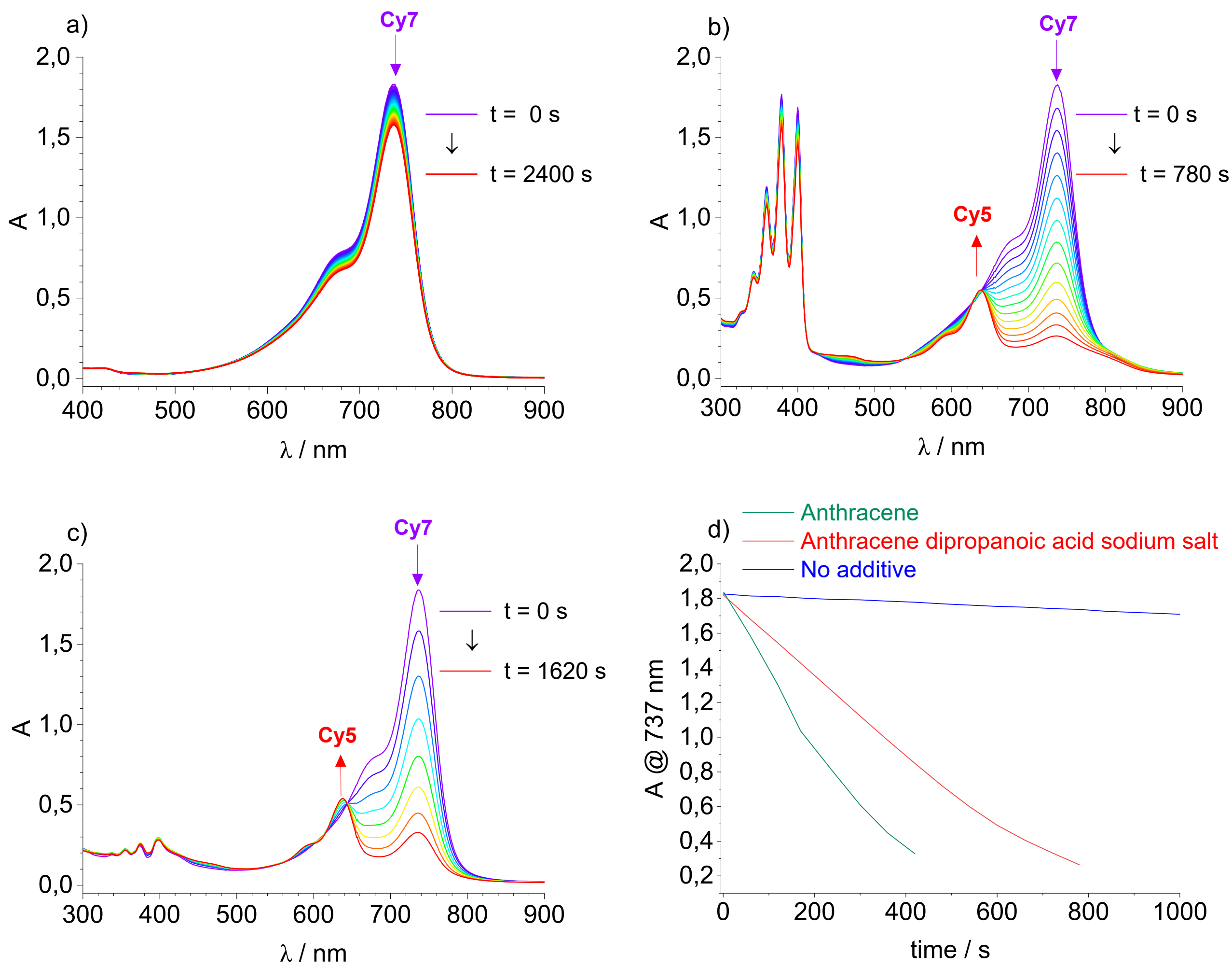

Figure S8. Experimental UV-Vis spectra vs. time for the irradiation of **Cy7** (10 μM) in **EA** buffer (500 mM, pH = 8.7) with a) no additive, b) anthracene-9,10-dipropanoic acid disodium salt (0.2 mM), and c) anthracene (0.05 mM), and d) absorbance at 737 nm (λ_{max} of **Cy7**) vs. time for a) to c) experiments. The samples were irradiated using LEDs with the emission maxima centered at 365 nm.

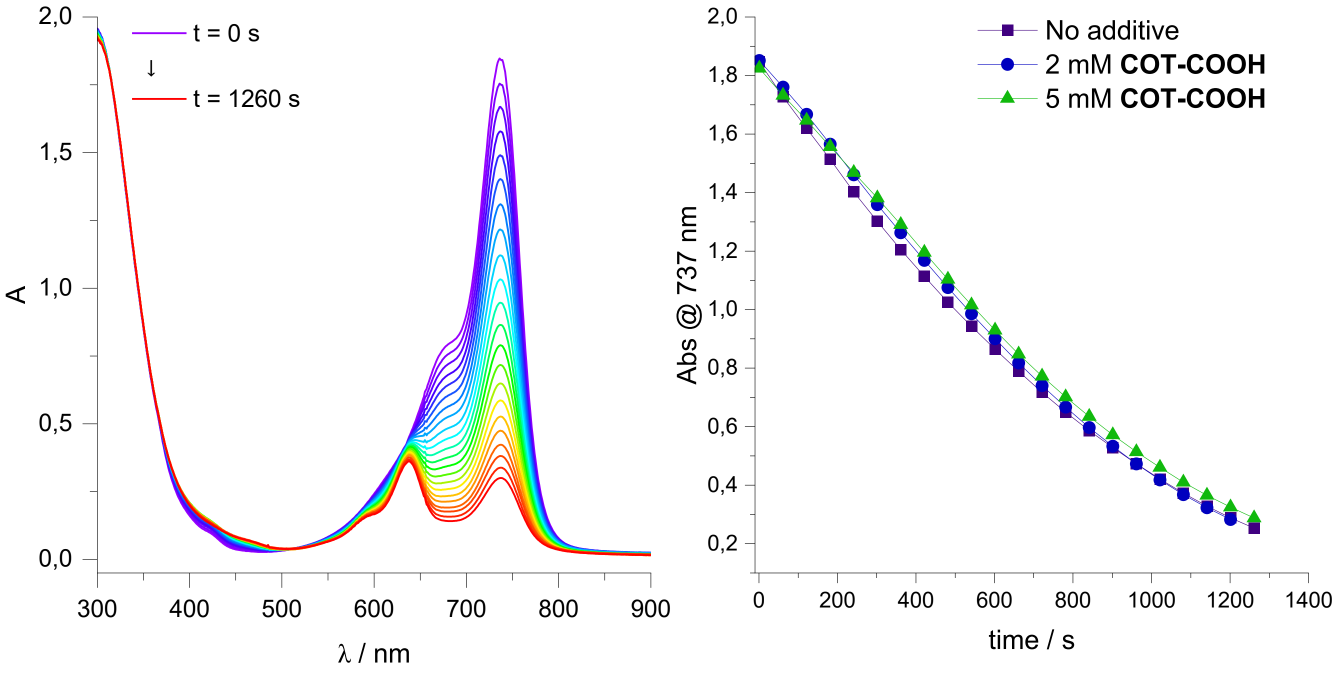

Figure S9. a) Experimental UV-Vis spectra vs. time for the irradiation of **Cy7** (10 μM) in **EA** buffer (500 mM, pH = 8.7) with **COT-COOH** (2 mM). b) Absorbances at 737 nm (λ_{max} of **Cy7**) vs. time for the experiment with no additive, or 2mM and 5 mM **COT-COOH**.

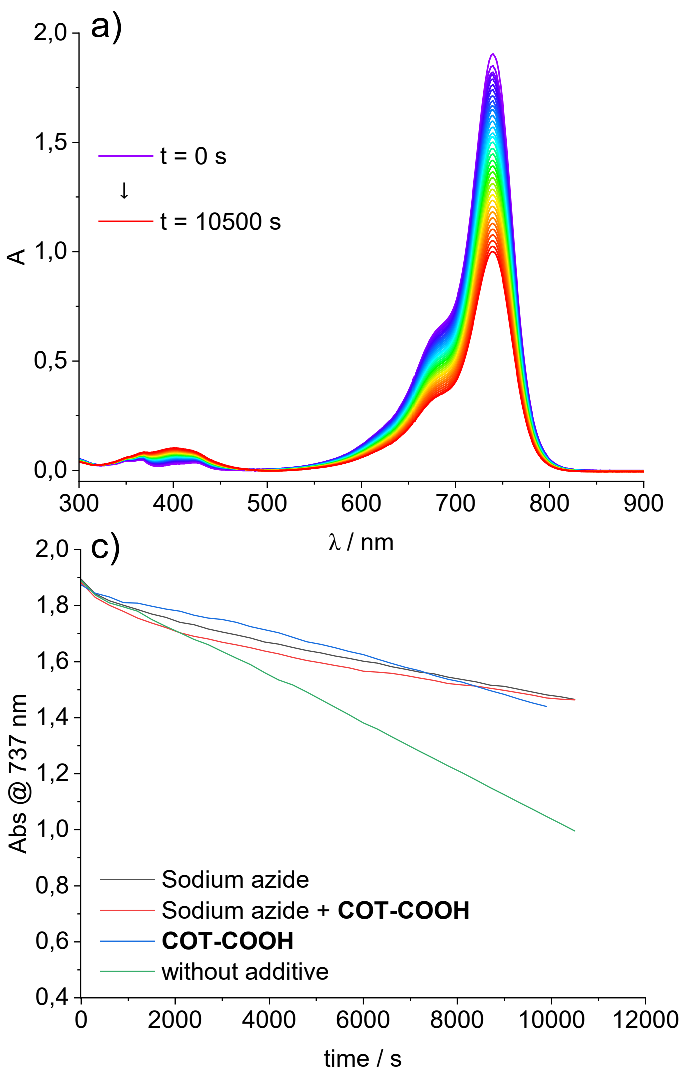

Figure S10. Experimental UV-Vis spectra vs. time for the irradiation of **Cy7** (10 μM) in methanol; a) without additive, b) with **COT-COOH** (2 mM), and c) absorbance at 737 nm (λ_{max} of **Cy7**) vs. time for irradiation of **Cy7** in methanol (without any additive, with 2 mM **COT-COOH**, and with 2 mM sodium azide as a singlet oxygen quencher).

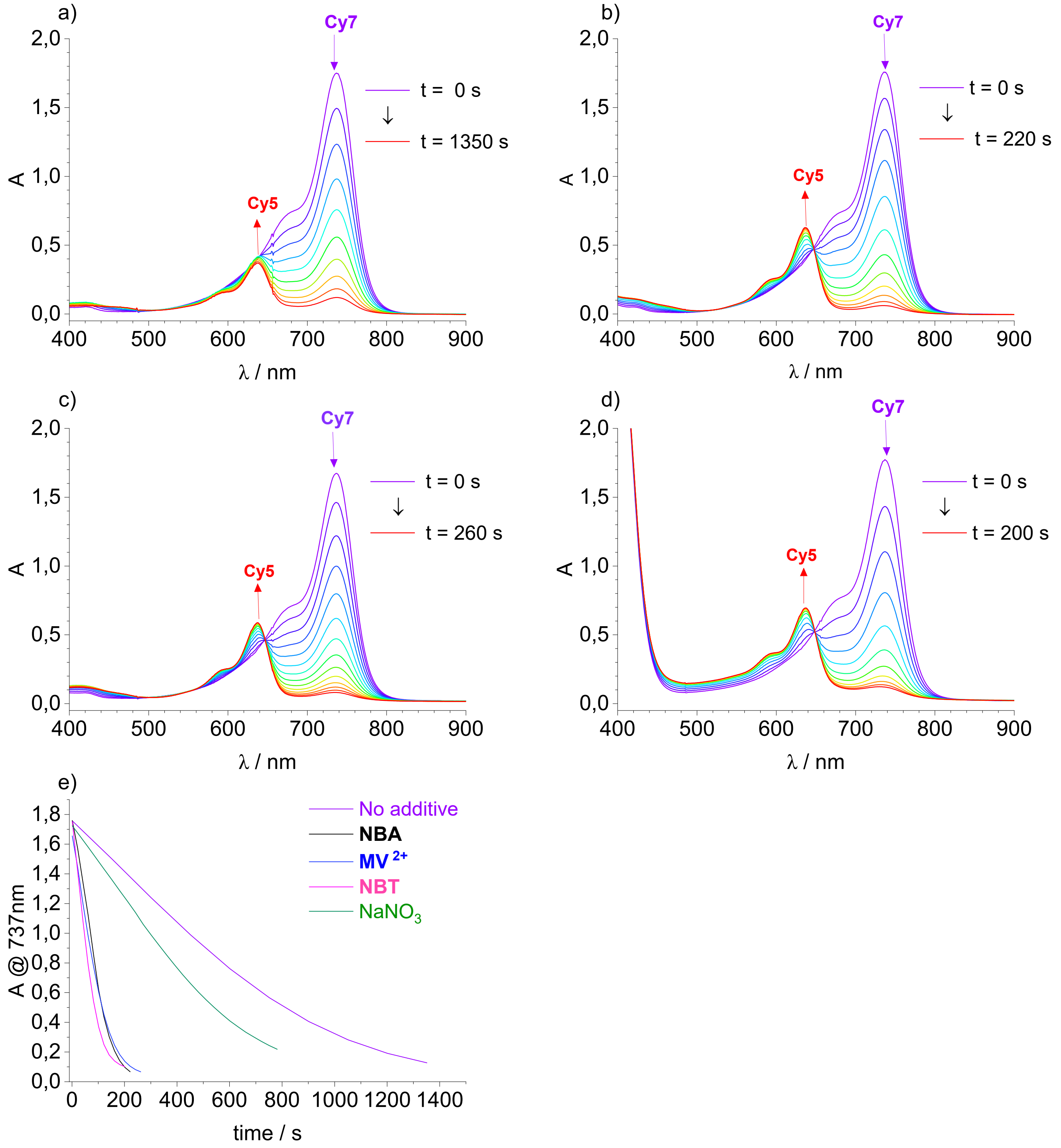

Figure S11. Experimental UV-Vis spectra vs. time for the irradiation of **Cy7** (10 μM) in **EA** buffer (500 mM, pH = 8.7) with a) no additive, b) 4-nitrobenzyl alcohol (**NBA**; 2 mM), c) methyl viologen (**MV**$^{2+}$; 2 mM), and d) nitro blue tetrazolium (**NBT**; 2 mM), and d) absorbance at 737 nm (λ_{max} of **Cy7**) vs. time for a) to d) experiments.

2.2.3. Photooxidation by camphorquinone

We used camphorquinone (**CQ**) - a well established type II photoinitiator as a photooxidant because of its high ISC efficiency ($\Phi_{ISC} \approx 1$), a high T_1 reduction potential ($E_{1/2}$ = 1.49 V vs. NHE), and a significant absorbance at 450 nm, the wavelength where **Cy7** features a minimal absorbance. The irradiation of **CQ** (2 mM) in **EA** buffer (500 mM, pH = 8.5) with **Cy7** (10 µM) at 450 nm, is shown on Figure S12. The irradiation of **CQ** in the presence of **EA** at 450 nm for an extended time period showed no changes in the concentration of either of the compounds, demonstrating the absence of PET between CQ* (T_1) and **EA**. To exclude the influence of $^1O_2$, which can be formed via sensitization by **CQ**, we used sodium azide as a quencher (Figure S12c).

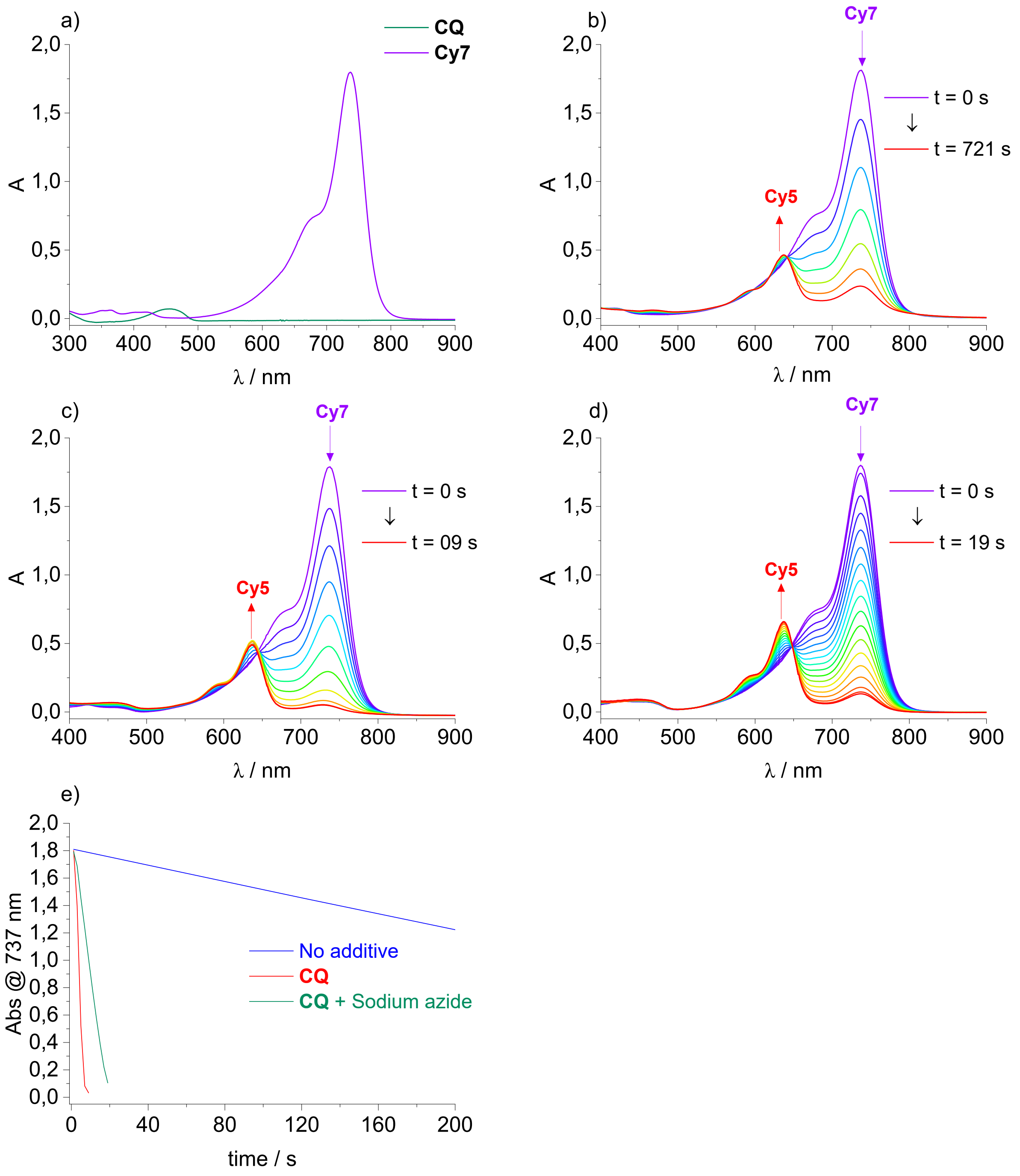

Figure S12. a) UV-Vis spectra of **Cy7** (10 µM) and camphorquinone (**CQ**; 2 mM) in **EA** buffer (500 mM, pH = 8.7). b) Experimental UV-Vis spectra vs. time for the irradiation of **Cy7** (10 µM) in **EA** buffer (500 mM, pH = 8.7) irradiated with LED with an emission maximum centered at 450 nm. c) The same conditions as in b) with 2 mM **CQ**. d) The same conditions as in b) with 2 mM **CQ** and sodium azide (5 mM). e) Absorbance at 737 nm (λ_{max} of **Cy7**) vs. time for a) to d) experiments

2.2.4. Impact of varying light intensity and wavelength of irradiation

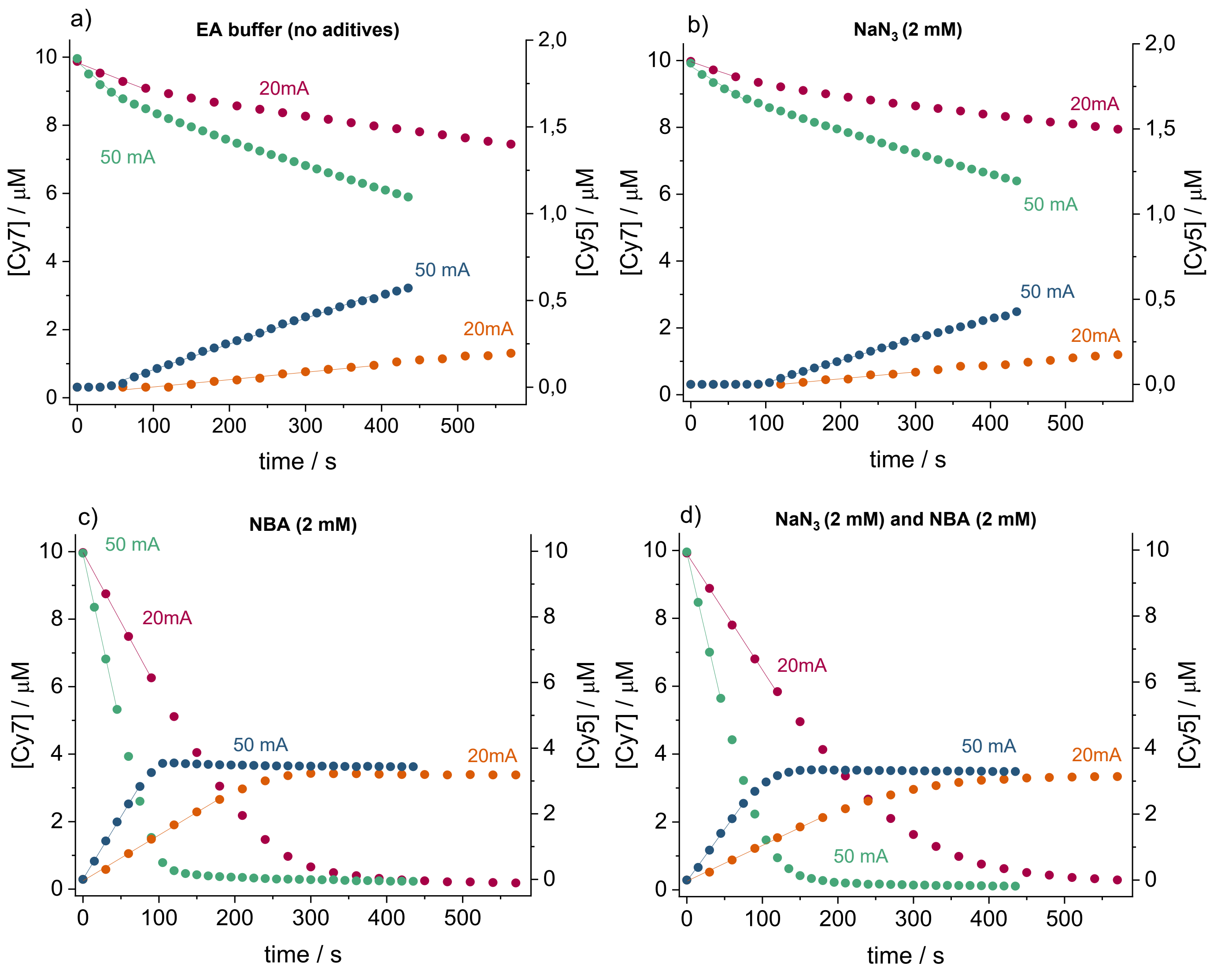

Figure S13. The influence of light intensity on phototruncation. The concentration vs. time profile for **Cy7** and **Cy5** during the irradiation in: a) a standard **EA** buffer (500 mM **EA,** 8.7 pH), b) **EA** buffer with 2 mM sodium azide, c) **EA** buffer with 2 mM **NBA**, and d) **EA** buffer with 2 mM **NBA** and 2 mM sodium azide.

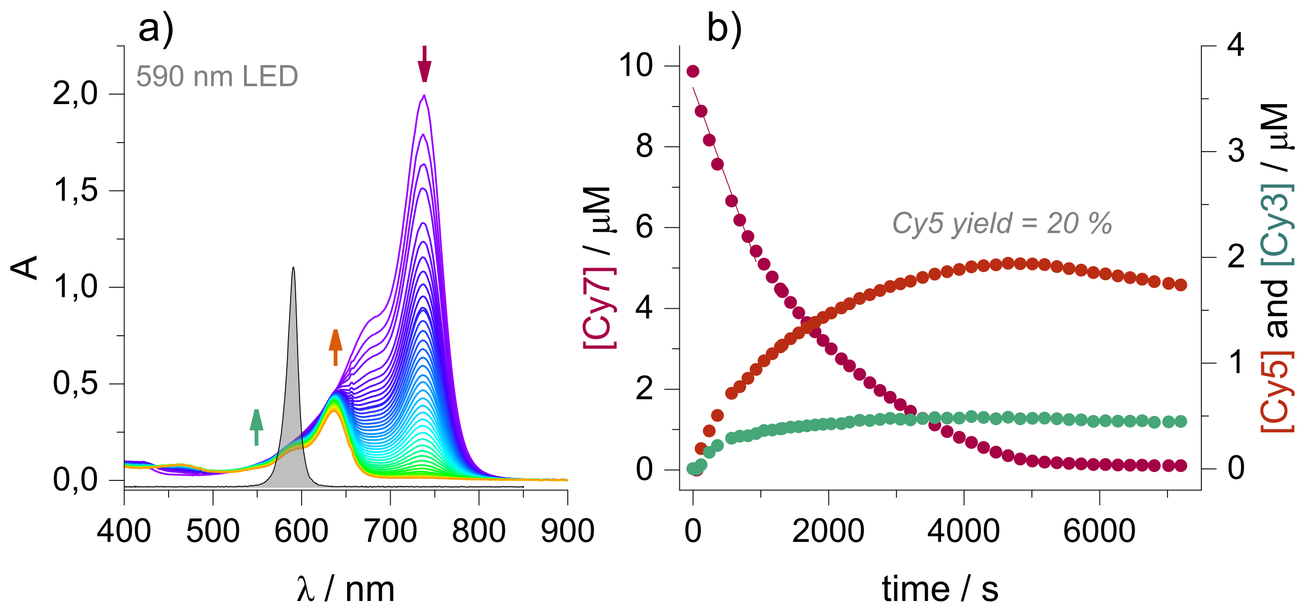

Figure S14. a) Experimental UV-Vis spectra vs. time for the irradiation of **Cy7** (10 μM) in **EA** buffer (500 mM, pH = 8.7, 2mM **NBA**) with LEDs at 590 nm (the LED emission spectrum shown in gray), b) concentration vs. time profile for **Cy7**, **Cy5**, and **Cy3**.

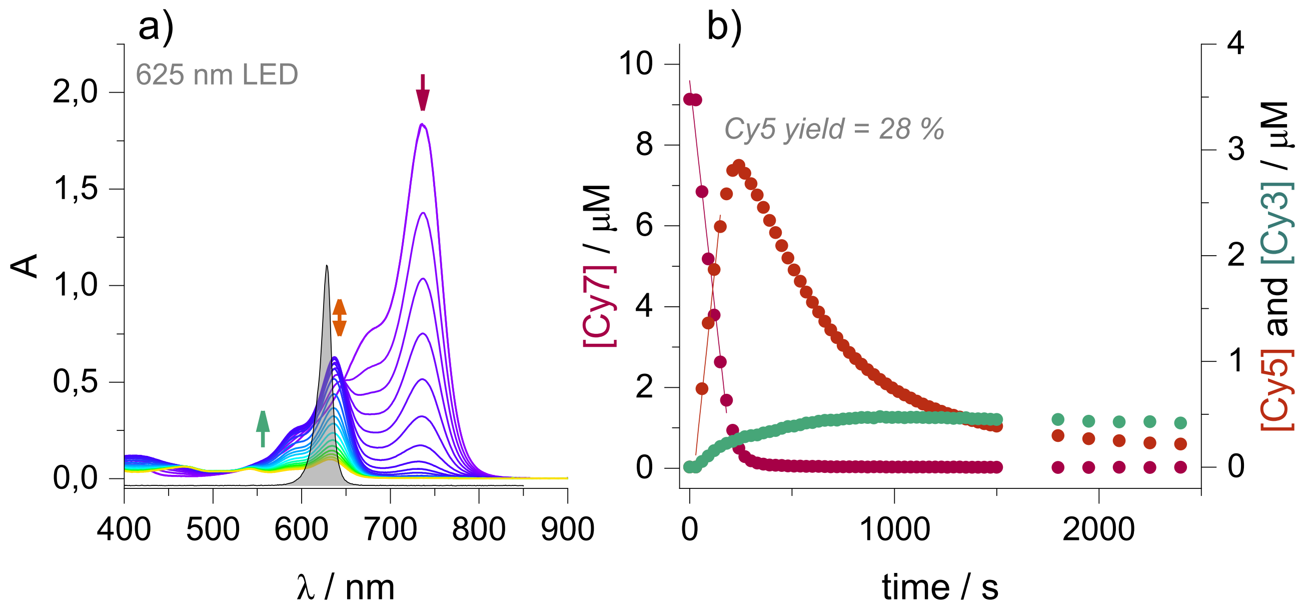

Figure S15. a) Experimental UV-Vis spectra vs. time for the irradiation of **Cy7** (10 μM) in **EA** buffer (500 mM, pH = 8.7, 2mM **NBA**) with LEDs at 625 nm (the LED emission spectrum shown in gray), b) concentration vs. time profile for **Cy7**, **Cy5**, and **Cy3**.

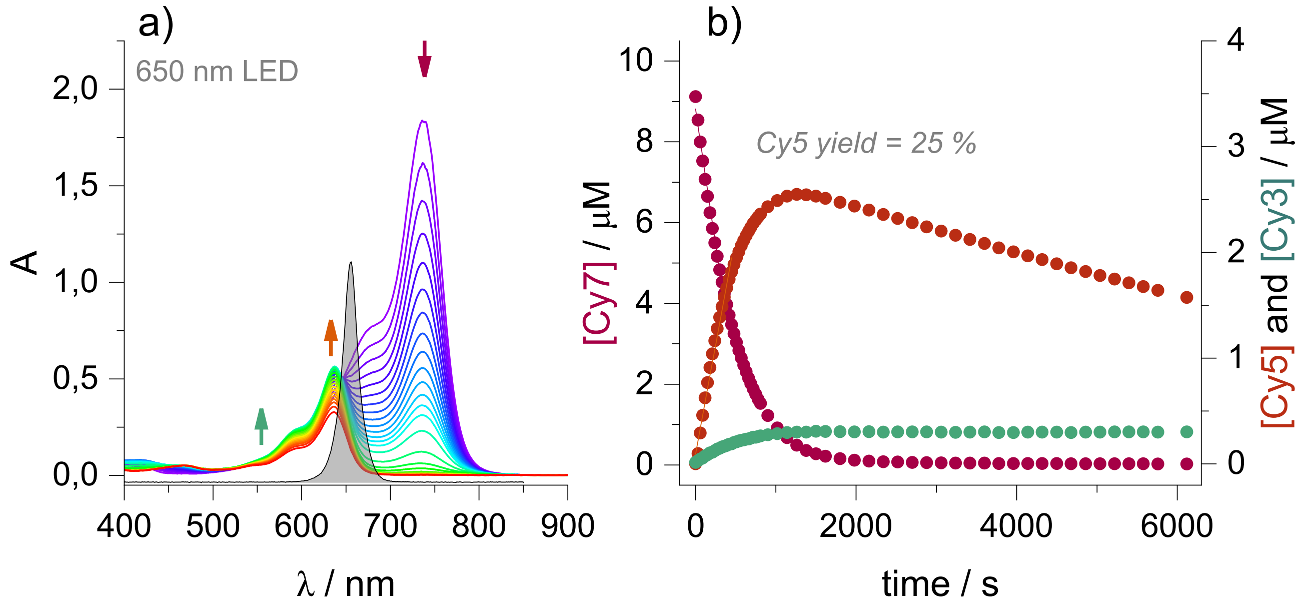

Figure S16. a) Experimental UV-Vis spectra vs. time for the irradiation of **Cy7** (10 µM) in **EA** buffer (500 mM, pH = 8.7, 2mM **NBA**) with LEDs at 650 nm (the LED emission spectrum shown in gray), b) concentration vs. time profile for **Cy7**, **Cy5**, and **Cy3**.

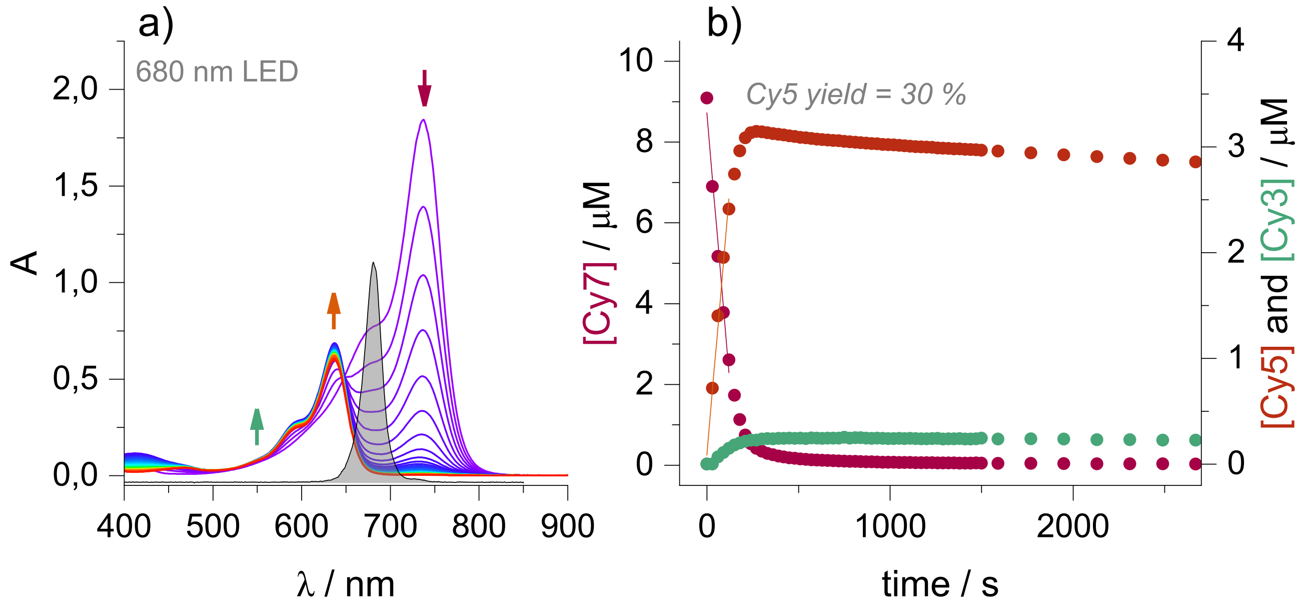

Figure S17. a) Experimental UV-Vis spectra vs. time for the irradiation of **Cy7** (10 µM) in **EA** buffer (500 mM, pH = 8.7, 2mM **NBA**) with LEDs at 680 nm (the LED emission spectrum shown in gray), b) concentration vs. time profile for **Cy7**, **Cy5**, and **Cy3**.

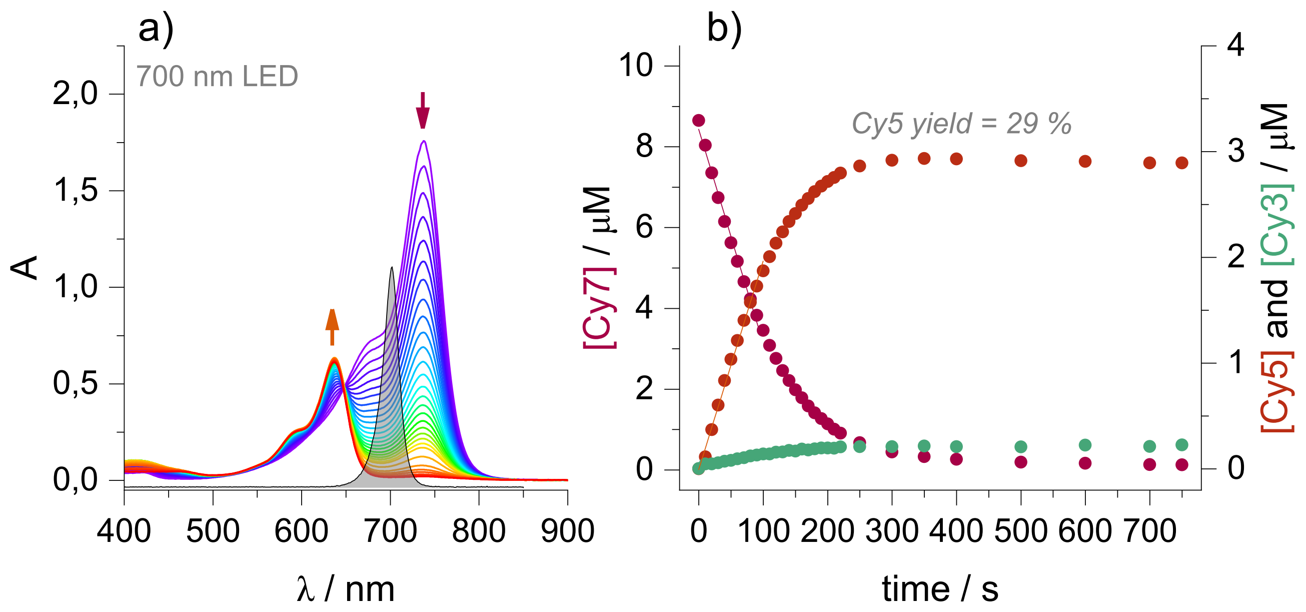

Figure S18. a) Experimental UV-Vis spectra vs. time for the irradiation of **Cy7** (10 µM) in **EA** buffer (500 mM, pH = 8.7, 2mM **NBA**) with LEDs at 700 nm (the LED emission spectrum shown in gray), b) concentration vs. time profile for **Cy7**, **Cy5**, and **Cy3**.

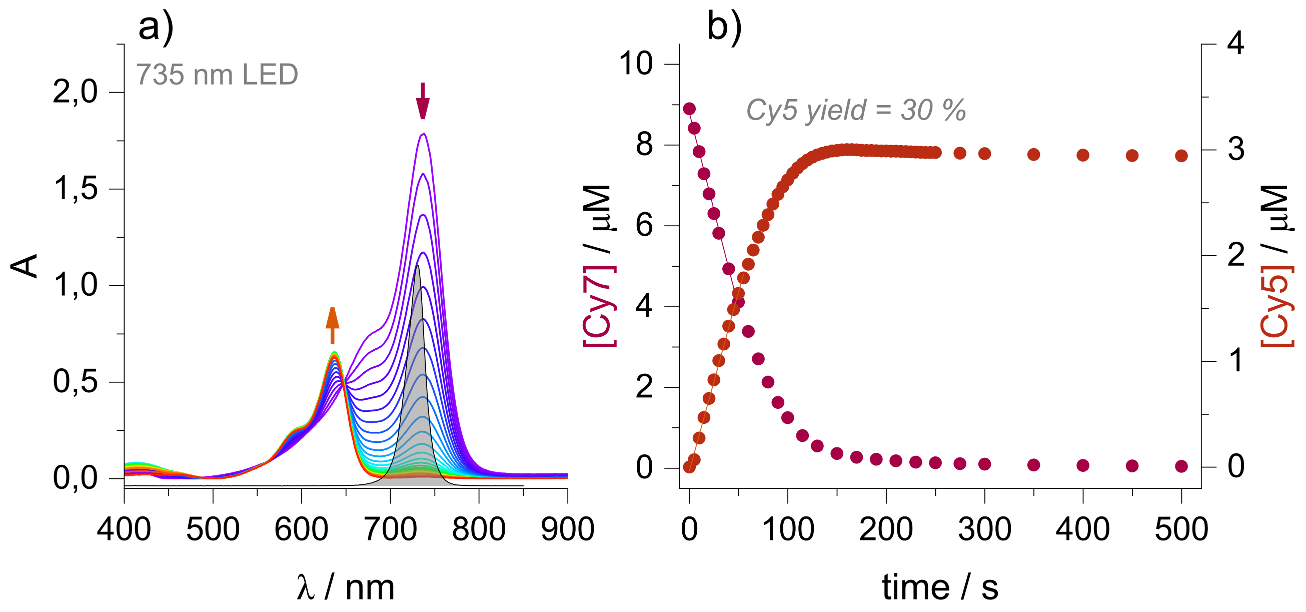

Figure S19. a) Experimental UV-Vis spectra vs. time for the irradiation of **Cy7** (10 µM) in **EA** buffer (500 mM, pH = 8.7, 2mM **NBA**) with LEDs at 735 nm (the LED emission spectrum shown in gray), b) concentration vs. time profile for **Cy7** and **Cy5**.

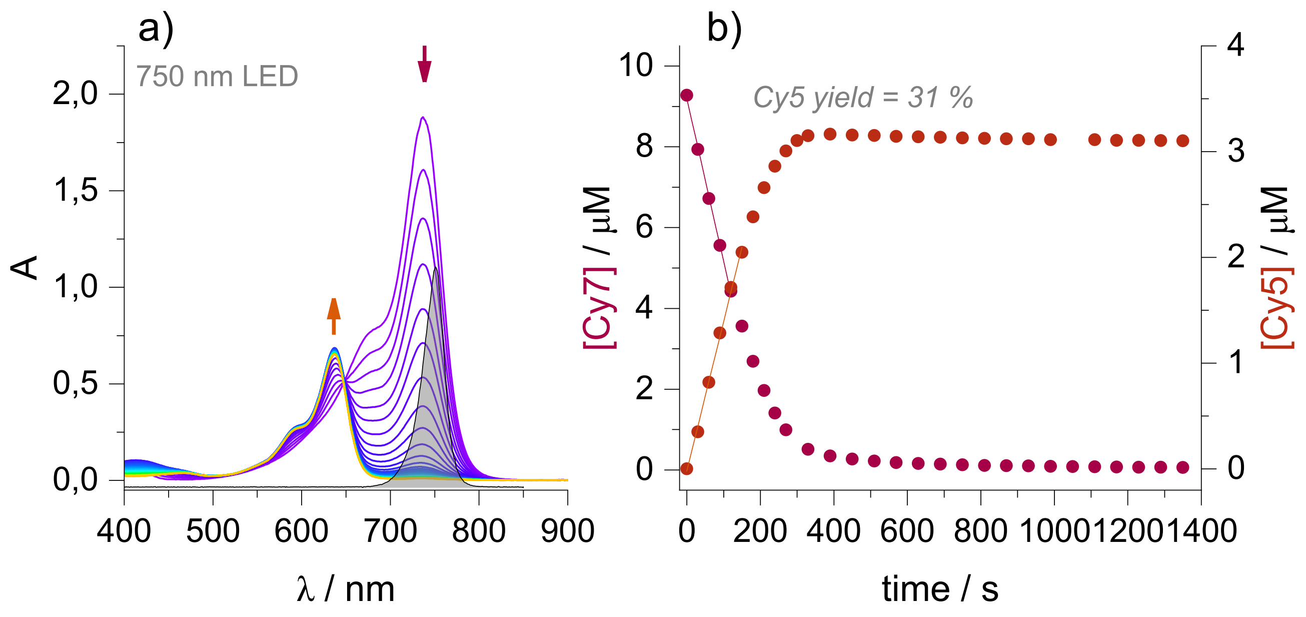

Figure S20. a) Experimental UV-Vis spectra vs. time for the irradiation of **Cy7** (10 µM) in **EA** buffer (500 mM, pH = 8.7, 2mM **NBA**) with LEDs at 735 nm (the LED emission spectrum shown in gray), b) concentration vs. time profile for **Cy7** and **Cy5**.

2.2.5. Irradiation of Cy5

For the irradiation of **Cy5**, LEDs with emission maxima centered at 625 nm were used (the emission spectrum shown in Figure S15).

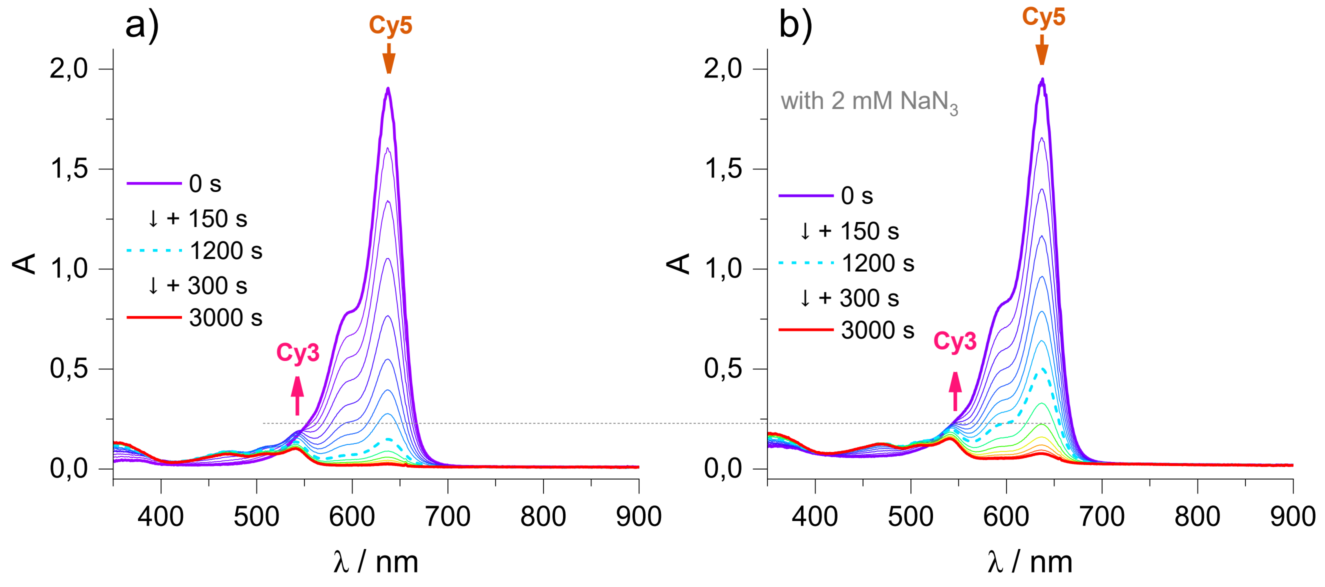

Figure S21. Experimental UV-Vis spectra for the irradiation of **Cy5** (10 µM) in **EA** buffer (500 mM, pH = 8.7, 2 mM **NBA**) irradiated using 625 nm LEDs, a) without sodium azide and b) with sodium azide (2 mM).

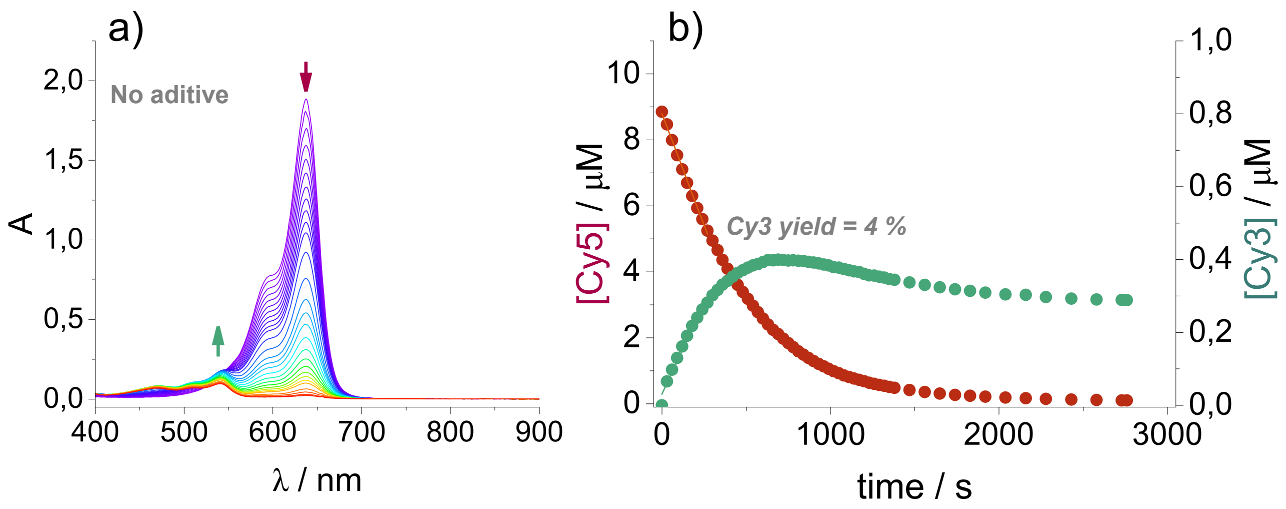

Figure S22. Irradiation of **Cy5** (9 µM) in **EA** buffer (500 mM, pH = 8.7, 2 mM **NBA**, irradiated using 625 nm LEDs); a) experimental UV-Vis spectra, b) concentration vs. time profile for **Cy5** and **Cy3**. The uncertainty in determining the **Cy3** yield is ±1%, as determined by a series of separate measurements.

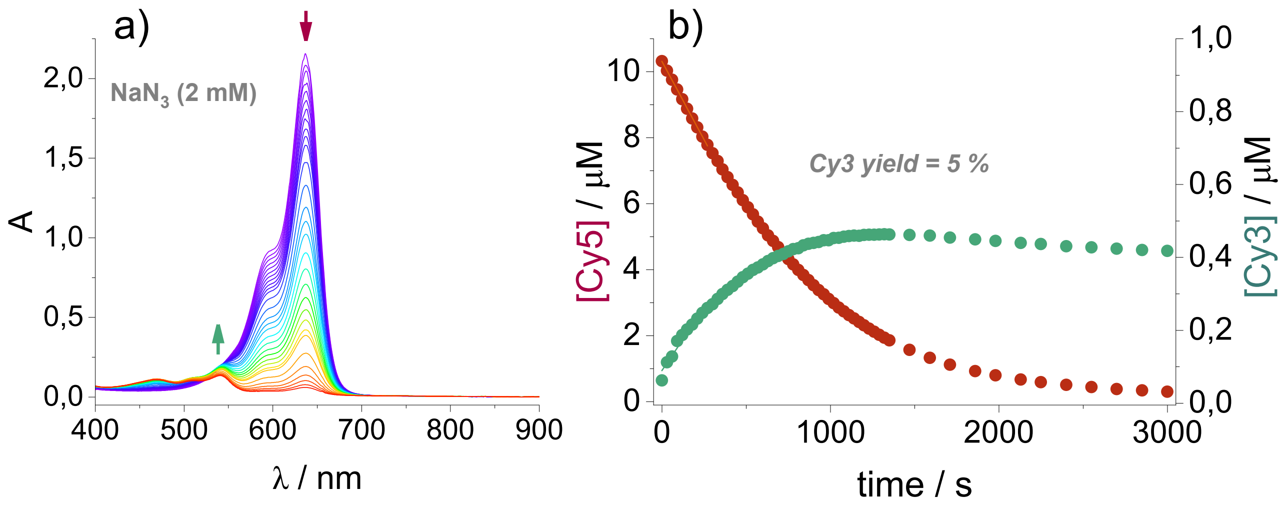

Figure S23. Irradiation of **Cy5** (9 µM) in **EA** buffer (500 mM, pH = 8.7, 2 mM **NBA**, and 2 mM sodium azide, irradiated using 625 nm LEDs); a) Experimental UV-Vis spectra, b) concentration vs. time profile for **Cy5** and **Cy3**. The uncertainty in determining the **Cy3** yield is ±1%, as determined by a series of separate measurements.

2.2.6. Other UVvis experiments

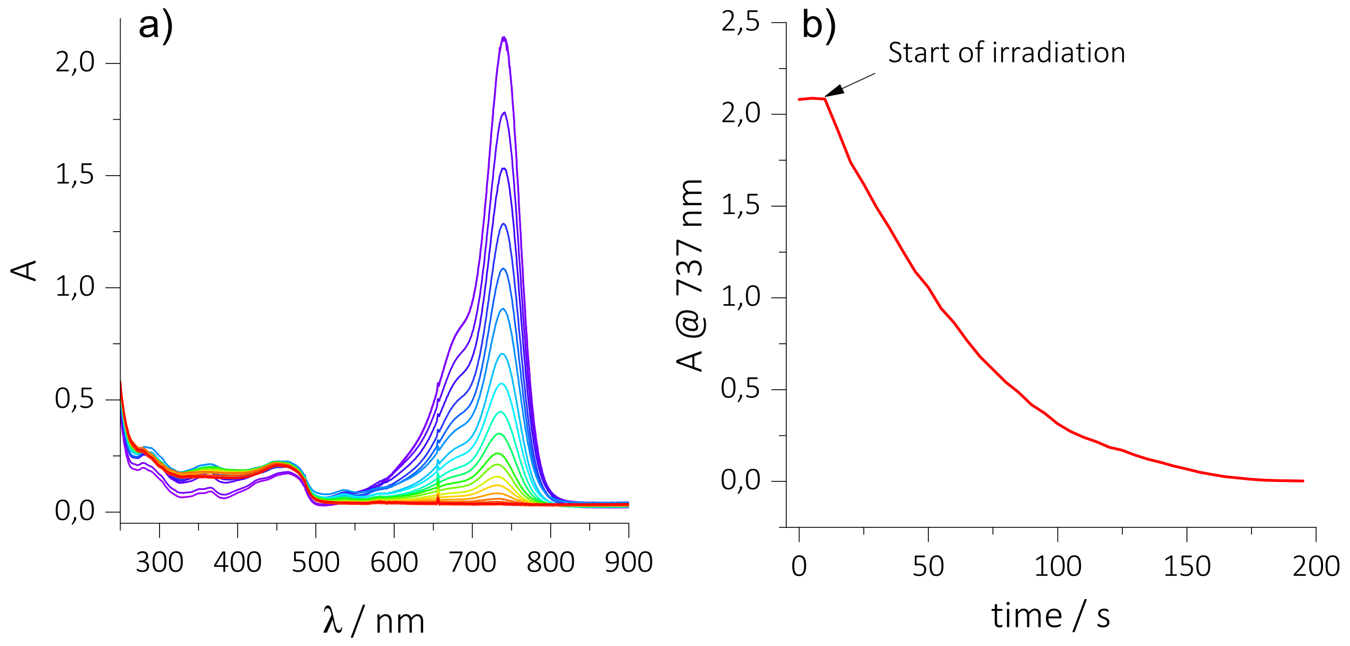

Figure S24. a) UV-Vis spectra of **Cy7** (10 µM) and camphorquinone (**CQ**; 2 mM) in CH_3CN/H_2O (2:1): a) Experimental UV-Vis spectra vs. time for the irradiation of **CQ**, irradiated with LEDs at 465 nm; b) absorbance at 737 nm (λ_{max} of **Cy7**) vs. time from the experiment in a).

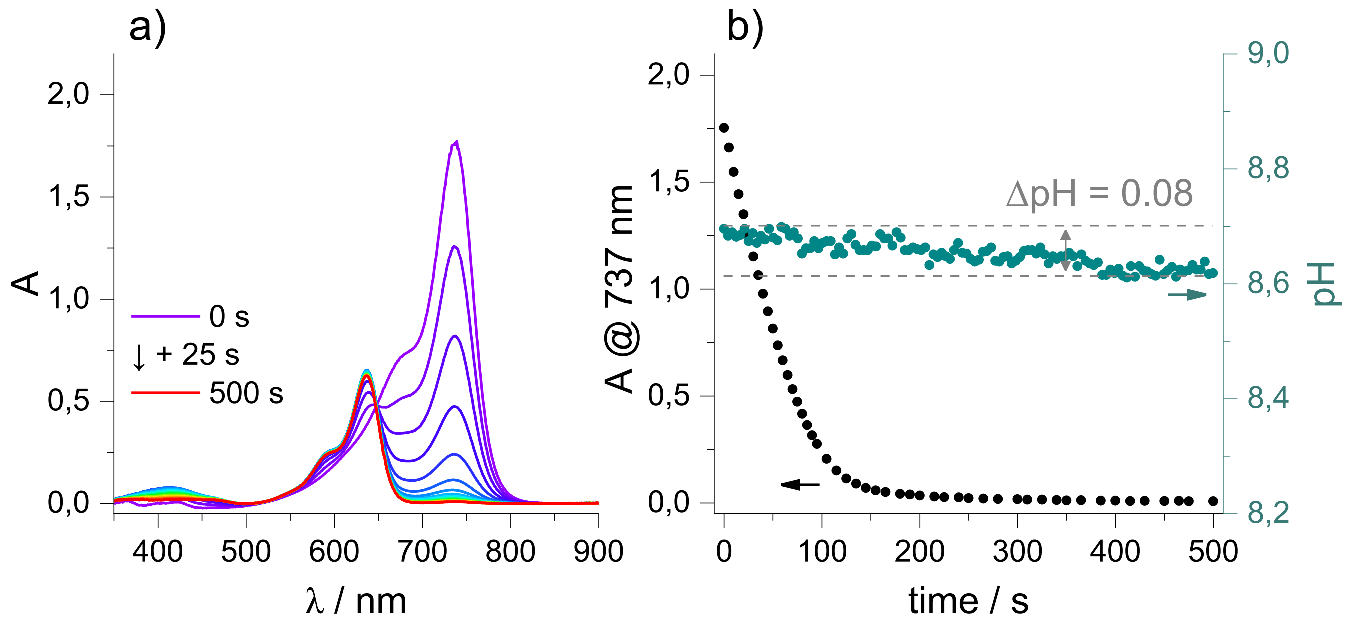

Figure S25. Experimental UV-Vis spectra for the irradiation of **Cy7** (10 µM) in **EA** buffer (500 mM, pH = 8.7, 2 mM **NBA**), irradiated using 735 nm LEDs; b) absorbance at 737 nm (λ_{max} of **Cy7**) and pH vs. time during the irradiation experiment.

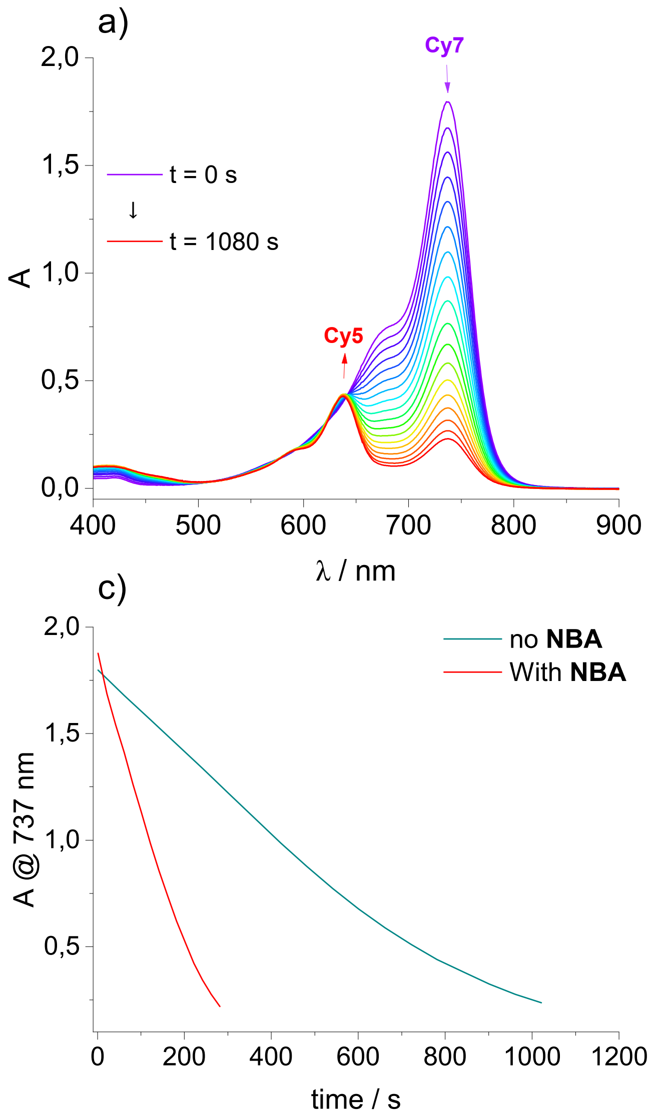

Figure S26. Experimental UV-Vis spectra vs. time for the irradiation of **Cy7** having PF_6 as the counterion (10 μM) in **EA** buffer (500 mM, pH = 8.7) with a) no additive and b) addition of **NBA** (2 mM), and c) absorbance at 737 nm (λ_{max} of **Cy7**) vs. time for a) and b) experiments.

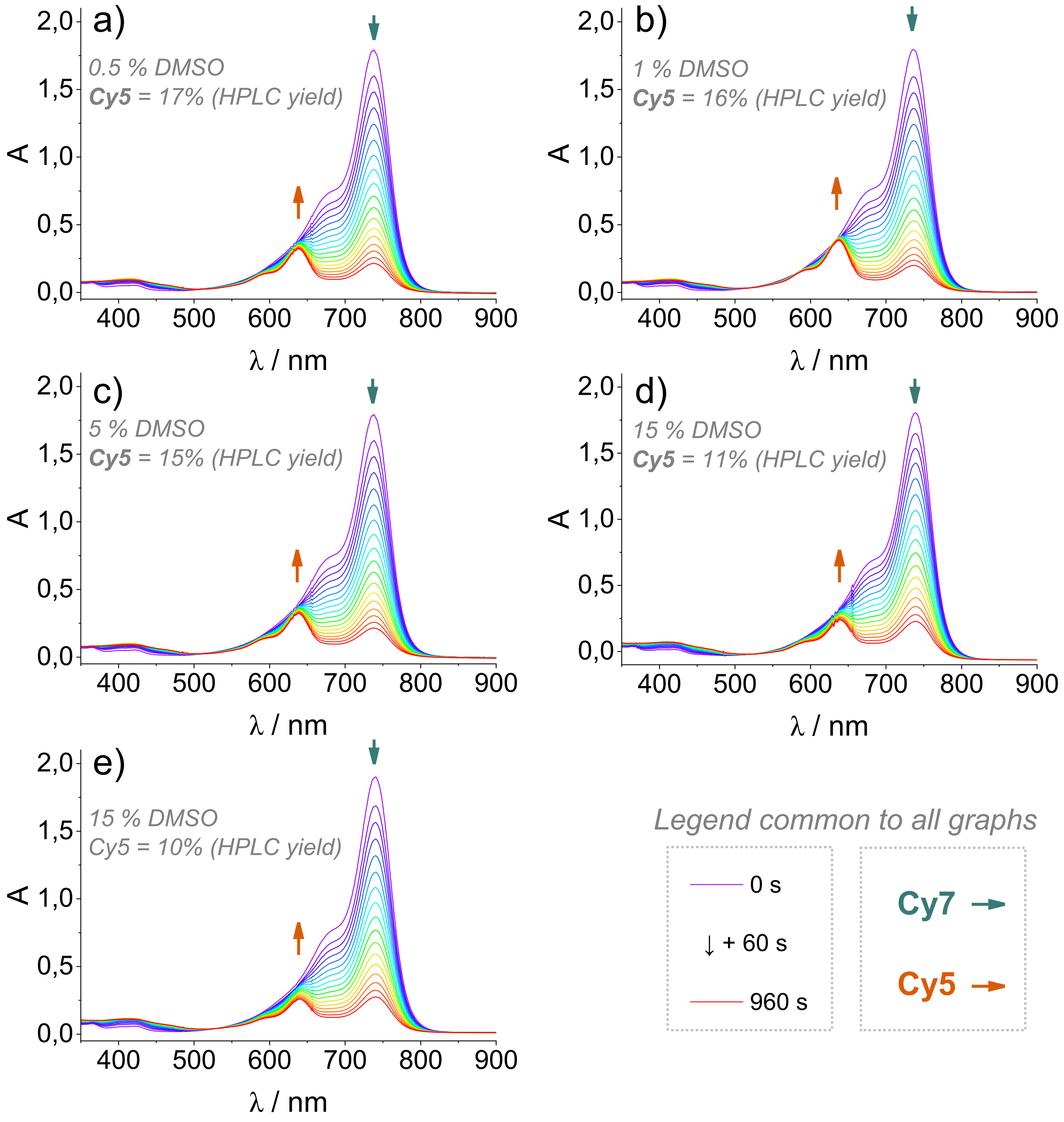

Figure S27. Experimental UV-Vis spectra for the irradiation of **Cy7** (10 µM) in **EA** buffer (500 mM, pH = 8.7) with an increasing amount of DMSO as a cosolvent from 0.5% (a) to 15% (e). The HPLC yield of **Cy5** is indicated on the respective graphs.

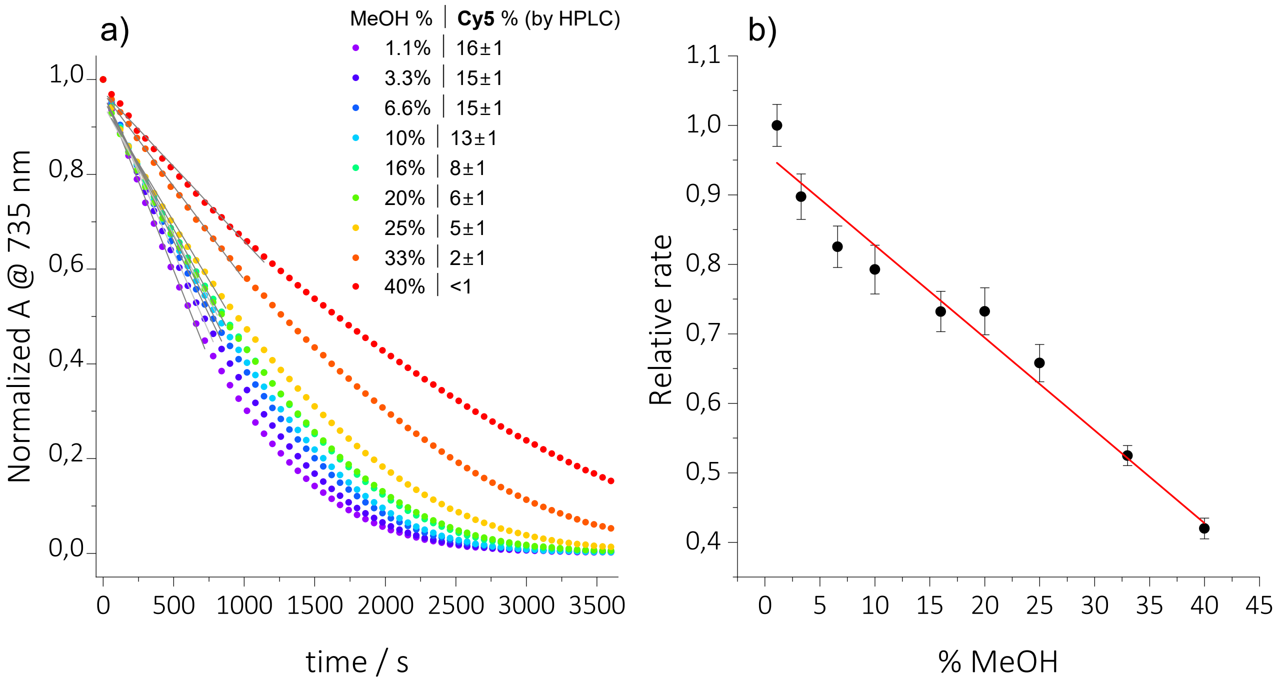

Figure S28. Irradiation of **Cy7** in **EA** buffer (500 mM, pH = 8.7) containing different amounts of methanol (in %; corrected for the volume changes). a) Absorbance at 737 nm (λ_{max} of **Cy7**) vs. time for the experiments with varying methanol amounts (from 1.1 to 40%), showing HPLC yields of **Cy5** in the legend. b) The calculated relative rate of **Cy7** decomposition (relative to 1.1% MeOH in buffer) for the experiments with varying methanol amount shown in the graph (a).

3. UVVis measurements

3.1. UVvis of Cy7 in EA buffer at different concentrations

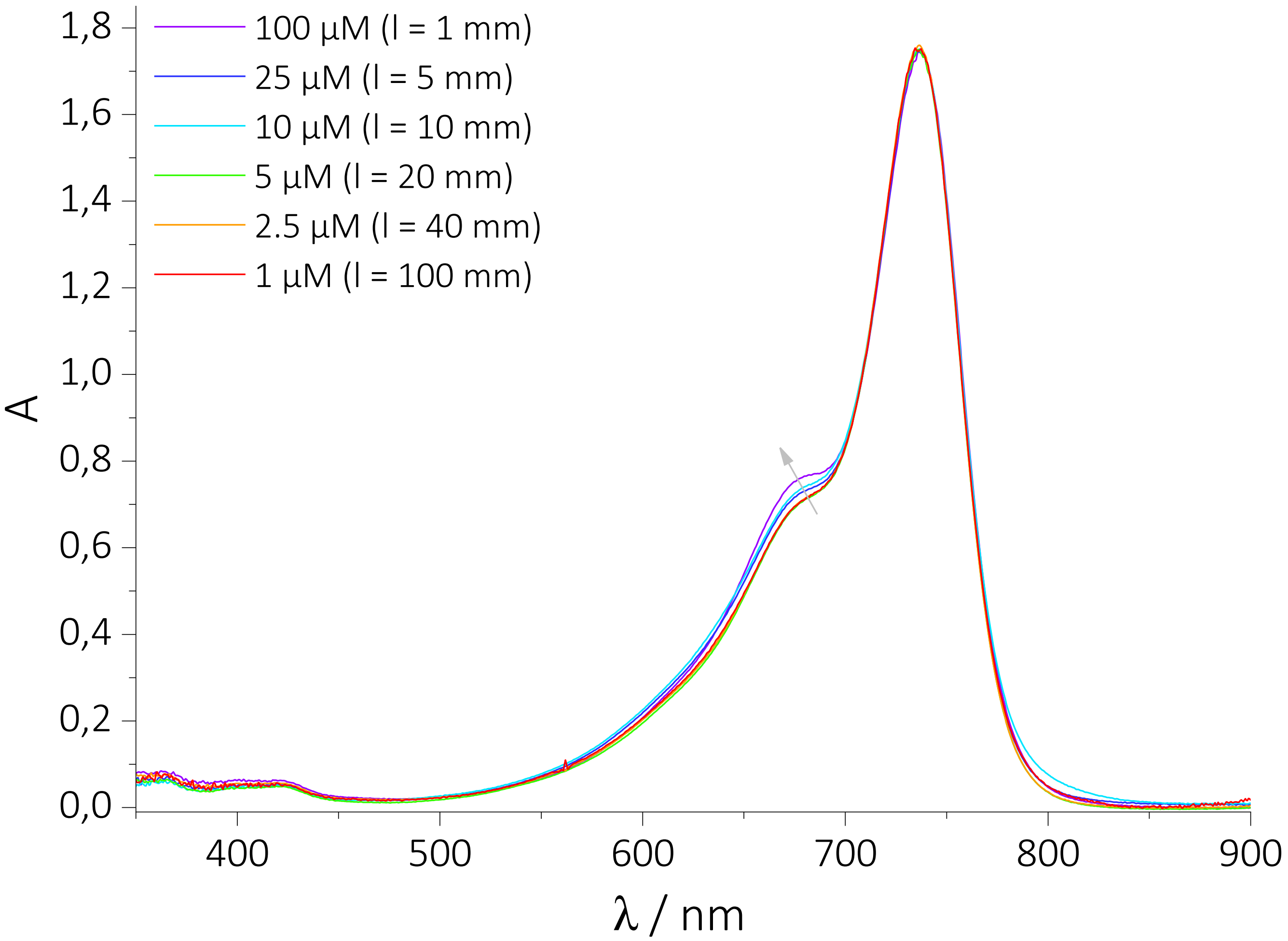

Figure S29. UV-vis spectra of **Cy7** at several concentrations in **EA** buffer (500 mM EA, pH = 8.7). The spectra were recorded in cuvettes of adequate optical path lengths (shown in the legend) to keep the absorbance withing the optimum operating range of the spectrometer.

3.2. UVVis spectra of Cy7-O_2 ground state complex in EA buffer

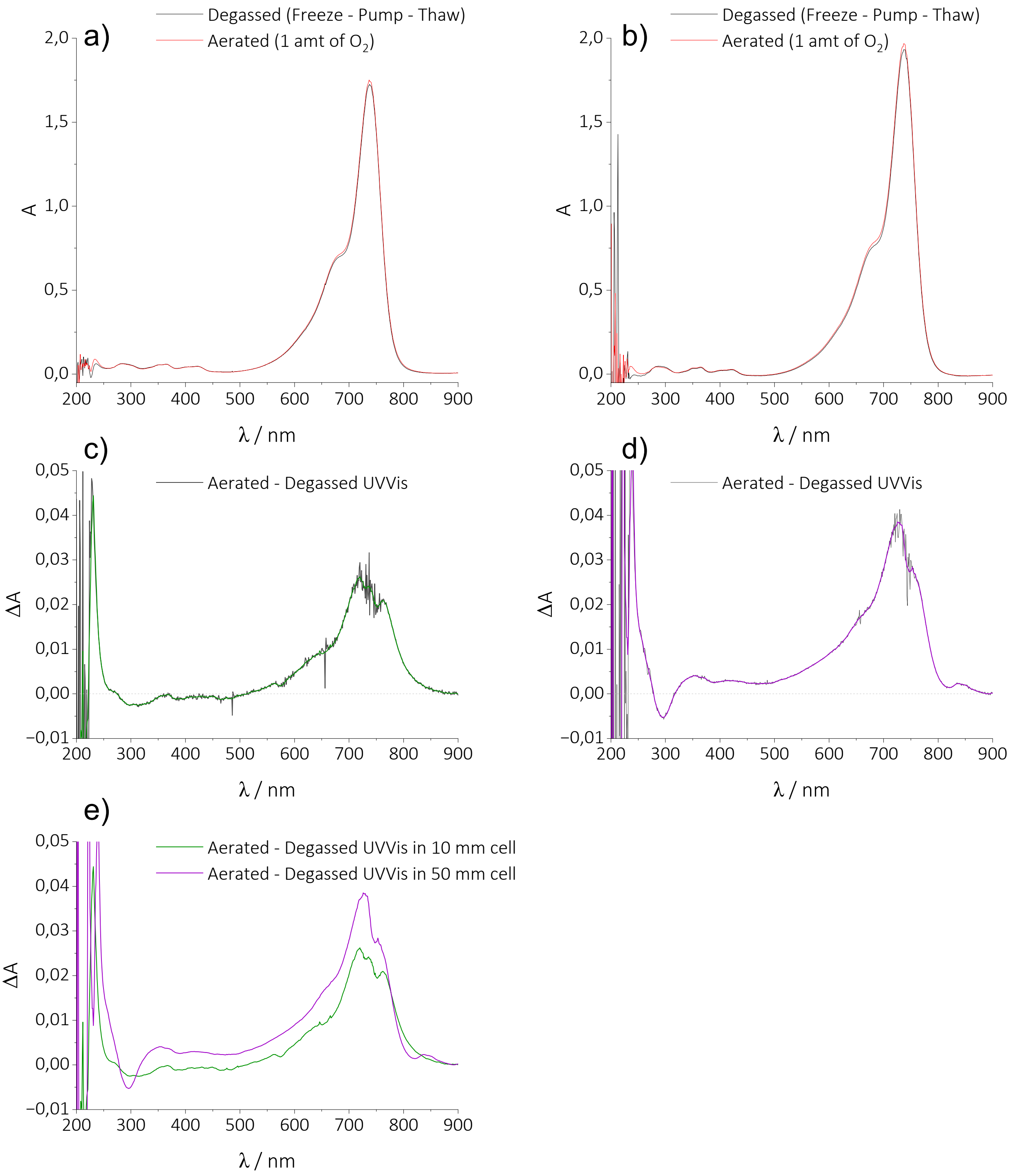

Figure S30. Absorption spectra of **Cy7** in aerated (1 atm of O_2, red line) and degassed (>4 cycles of freeze–pump–thaw, black line) **EA** buffer (500 mM **EA**, pH = 8.7). The figures a) and b) show the UV-Vis spectra of **Cy7** in the presence and absence of O_2, in 10 mm cell (10 µM - **Cy7**) and 50 mm cell (2 µM - **Cy7**) respectively, while figures c) and d) are the difference spectra of aerated vs.. degassed samples from the same experiments. The gray line depicts the raw difference spectra while colored line are smoothed spectra – using the Savitzky-Golay smoothing (points of window: 15, polynomial order: 2). Figure e) shows the comparison of the two difference spectra from c) and d). The measurements were taken using an instrument that was powered on for at least two hours in a temperature-controlled room to stabilize the light source. The cuvette was placed in the same position each time. Due to the **Cy7** adsorption on the walls of the degassing cell, the degassed sample was measured first, followed by opening the degassing cell and connecting it to a balloon containing pure O_2, which saturated the solution with O_2 (aerated).

4. Quantum yield measurements

4.1. Experimental setup

The quantum yields were determined according to our previously published method.[6-7] The quantum yield measurements of **Cy7** decomposition and **Cy5** formation were performed in a custom-made setup shown in Figure S31. Briefly, light from the high-power LED (LED735-66-60, Roithner Laser Technik Gmbh, λ_{max} = 735 nm/1 W, mounted on aluminum heat sink equipped with 12V DC fan to keep it at a constant operating temperature, was supplied by 12V/0.7A using Rigol DP821 power supply) and collimated by the combination of aspheric condenser lens and concave mirrors, entering the sample in a cuvette (3 mL with 1 cm optical path length) at the right angle. The photon flux from LEDs going through the cuvette was measured using a photodiode (Thorlabs S120VC, 200-1100 nm, 50 mW) connected to the power meter (Thorlabs PM100D). The content of cuvettes was stirred during the irradiation by a magnetic stirrer (Hellma cuv-o-stir, model 333). The Specord S600 from Analytikjena was used for the UV-Vis measurements. All of the measurements were performed at 20 ± 1 °C, and sample preparations were conducted under semi-dark conditions.

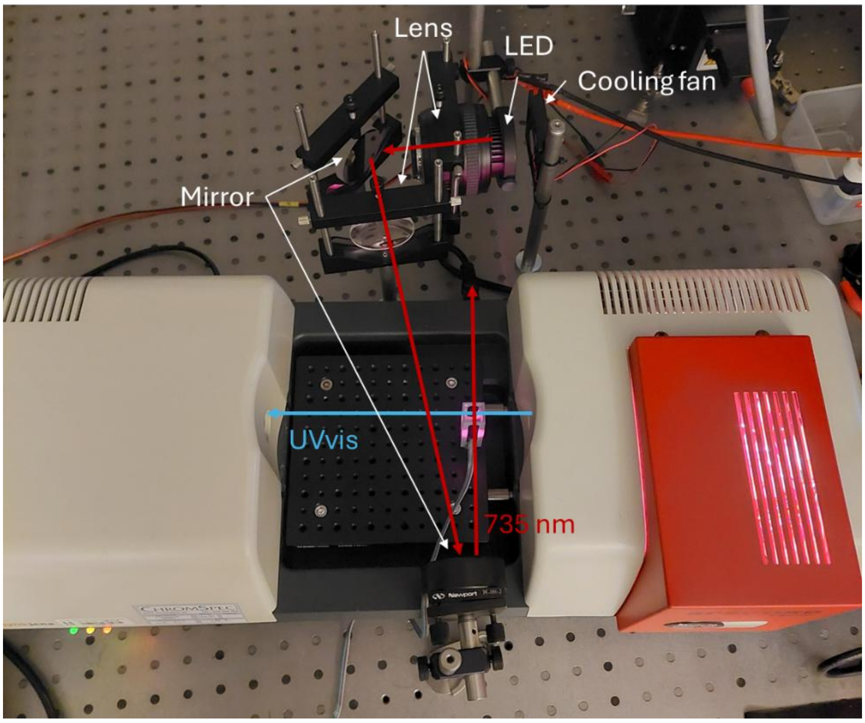

Figure S31. The experimental setup for quantum yield measurements.

4.2. Theory

Quantum yield of the ***Cy7*** *decomposition:* The time-dependent decay of **Cy7** during the photoreaction can be described using the following equation:

$$\frac{dc_{Cy7}(t)}{dt} = -\Phi_r J_A(t) \quad \text{(S1)}$$

where c_{Cy7} is the time-dependent concentration of **Cy7**, J_A is the time-dependent absorbed photon flux density (mol s^{-1} L^{-1}) and Φ_r is the quantum yield of the photoreaction. Since decay of **Cy7** depends only on the amount of light absorbed and Φ_r and, moreover, only **Cy7** absorbs at the irradiation wavelengths (Figure S32a), the kinetic equation can be integrated, providing that the absorbance of **Cy7** at the irradiation wavelength is:

$$A(\lambda_{irr}, t) = l c_{Cy7} \varepsilon_{Cy7}(\lambda_{irr}) \quad \text{(S2)}$$

where l is the optical path length (1 cm), and ε_{Cy7} is the molar absorption coefficient at the irradiation wavelength of **Cy7**, (L mol^{-1} cm^{-1}). The absorbed photon flux density can be calculated from absorbance and the incident photon flux density (J_0)

$$J_A(t) = J_0(1 - 10^{-A(\lambda_{irr},t)}), \text{ where } J_0 = \frac{q_{n,p}^0}{V} \quad \text{(S3)}$$

where $q_{n,p}^0$ is the incident photon flux (mol s^{-1}) and V is the volume of the solution (L), the $1 - 10^{-A(\lambda_{irr},t)}$ term is an absorption factor and denotes the fraction of the absorbed light. The differential and integrated forms of equation **3** are presented below:

$$\frac{dc_{Cy7}(t)}{dt} = -\Phi_r J_0(1 - 10^{-(lC_{Cy7}(t)\varepsilon_{Cy7}(\lambda_{irr})}) \quad \text{(S4)}$$

$$A(\lambda_{irr}, t) = log\big[1 = \big(10^{A(\lambda_{irr},t)} - 1\big)\, 10^{-J_0\Phi_r l\varepsilon_{Cy7}(\lambda_{irr})t}\big] \quad \text{(S5)}$$

The term $(\lambda_{irr}, 0) = \ lC_{Cy7}(0)\varepsilon_{Cy7}(\lambda_{irr})$ is an initial absorbance at t = 0 (without irradiation). The integrated form can be linearized:

$$\log\big(10^{A(\lambda_{irr},0)} - 1\big) - \log\big(10^{A(\lambda_{irr},t)} - 1\big) = \ J_0\Phi_r l\varepsilon_{Cy7}(\lambda_{irr})t \quad \text{(S6)}$$

in the limiting case where absorbance at λ_{irr} is sufficiently high (A ≈ ; almost all of the light is absorbed) and the exponential term in equation S4 can be omitted, resulting in the zeroth order kinetics (Equation S7, see Figure S32c).

$$-\frac{dc_{ACy7}(t)}{dt} = -\Phi_r J_0 \quad \text{(S7)}$$

Using the equation S6, plot of y ($\log\big(10^{A(\lambda_{irr},0)} - 1\big) - \log\big(10^{A(\lambda_{irr},t)} - 1\big)$) *vs.* t ($J_0\Phi_r l\varepsilon_{Cy7}(\lambda_{irr})$) is created, and the Φ_r is determined from the slope according to the Equation S8:

$$\Phi_r = \frac{a\ (slope)}{J_0 l\varepsilon_{Cy7}(\lambda_{irr}) \times (1-R)} \quad \text{(S8)}$$

Where: $l = 1\ cm$, ε_{Cy7} is known for the **Cy7** at the irradiation wavelength (ε = 178 000 L mol^{-1} cm^{-1} at 735 nm in H_2O),[8] R is the factor accounting for the reflections at the surface of the cuvette (≈ 0.036) and J_0 is determined according to:

$$J_0 = \frac{q_0}{V} \quad \text{(S9)}$$

Where V is the volume of the solution (0.003 L) and $q_0 = \ \int P\ \times I_{pd}$ where P is the integral of LED emission spectrum multiplied by Q_{rel} (Equation S10) and I_{pd} (A) is the current produced on the photodiode as a result of light passing through cuvette filled with pure solvent.

$$Q_{rel} = \frac{\lambda\ (in\ m)}{N_A hcR(\lambda)} \quad \text{(S10)}$$

Where N_A is Avogadro`s constant (6.02×10$^{23}$ mol$^{-1}$), $h$ is Planck`s constant (6.63×10^{-34} J s), c is the speed of light in vacuum (299 792 458 m s^{-1}), and $R(\lambda)$ is the responsivity of the photodiode (provided by the manufacturer).

Quantum yields of the ***Cy5*** *formation:* The first step in determining the quantum yield of **Cy5** formation (Φ_{Cy5}) is to extract the UV-Vis spectra of **Cy5** from the recorded time-dependent absorption spectra (Figure S32b). To separate the spectra of **Cy7** and **Cy5**, a custom function: Y = A**Cy7**×C1 + A**Cy5**×C2 was created in OriginPro2023 (64-bit), 10.0.0.154 (Academic), OriginLab Corporation software. The Y represents an experimental UV-Vis spectrum at the given time (Figure S32b), A**Cy7** and A**Cy5** are the normalized UV-Vis spectra of pure **Cy7** and **Cy5**, respectively (Figure S32a). The C1 and C2 are the associated coefficients (output of the fit), *i.e.*, the values by which the UV-Vis spectra of pure **Cy7** and **Cy5** (normalized to unit absorbance) need to be multiplied for their sum to be equal to the experimental UV-Vis spectrum. The C1 and C2 coefficients are proportional to the concentration, and the proportionality constants are provided by the molar absorption coefficients of **Cy7** and **Cy5**. The Levenberg-Marquardt iteration algorithm was used with a maximum of 500 iterations, and a 1× 10^{-12} tolerance was set as a default. For details on how to create a custom function, see page 135 of the Origin tutorial pdf document.[9] The extracted UV-Vis spectra of **Cy5** (Figure S32d) allowed us to calculate the **Cy5** concentration *vs.* time (see Figure S32d insert) from which Φ_{Cy5} was obtained. To that end, the [**Cy5**] *vs.* time plot was first fitted with a linear function, and the slope was used to calculate Φ_{Cy5} with Equation S11.

$$\Phi_{Cy5} = \frac{a\ (slope)}{J_0} \quad \text{(S11)}$$

where J_0 is the same as determined for Φ_r of **Cy7** (Equation S9).

The quantum yields for the decomposition of **Cy7** and the formation of **Cy5** were measured depending on the concentration of **EA** (Table S1 - S2, and Figure S33), pH (Table S3, and Figure S34), additives such as **NBA** and **MV**$^{2+}$ (Table S4 – S5, and Figure S35), and in the presence of molecular oxygen (Table S3, and Figure S36).

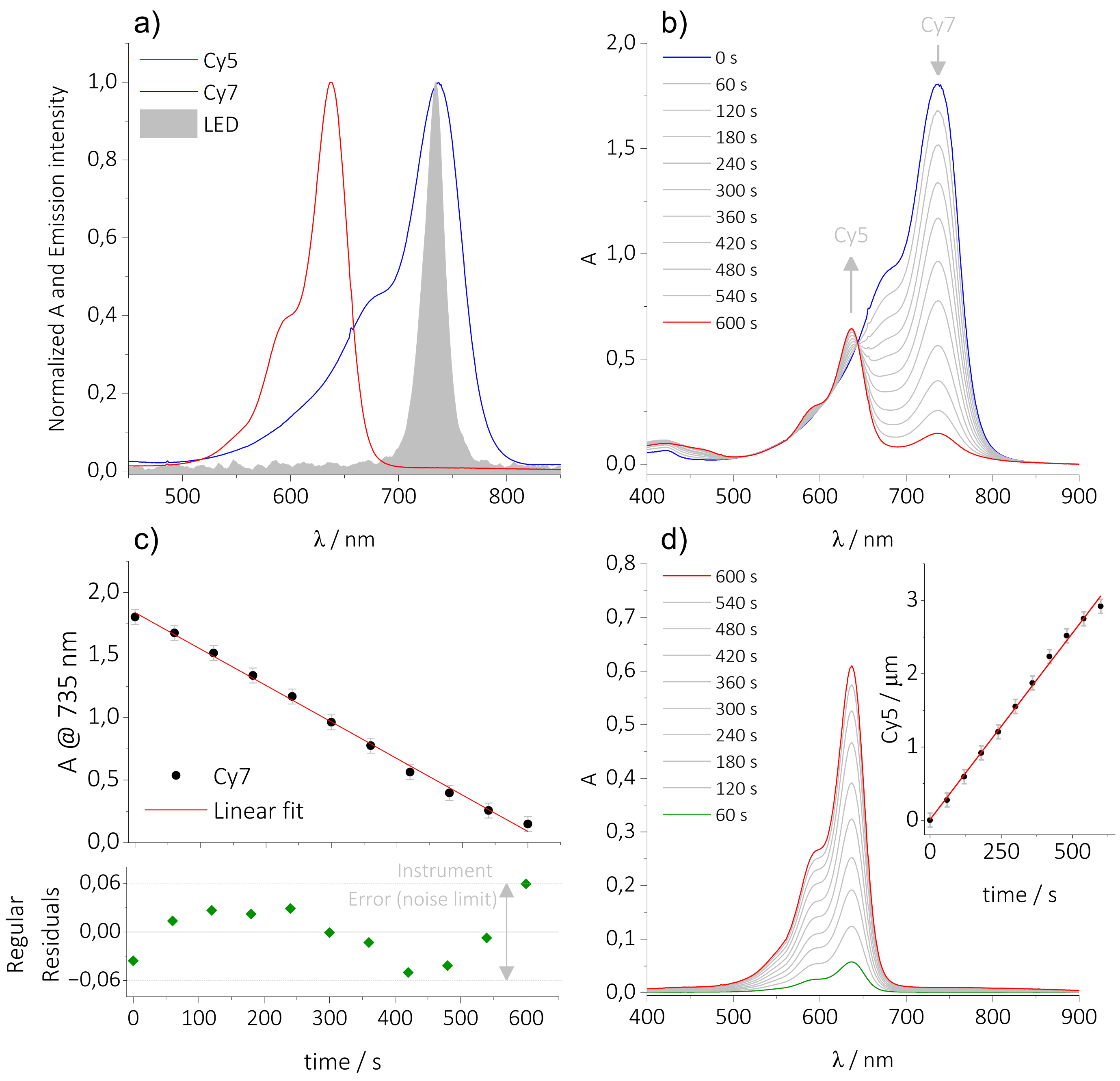

Figure S32. The quantum yields: a) normalized experimental UV-Vis spectra of pure **Cy7** and **Cy5**, showing the overlap between the two, together with the normalized emission spectrum of high-power light-emitting diode (LED) used during the measurements (emission maximum is centered at 735 ± 1 nm). Importantly, the emission profile of the LED overlaps perfectly with the absorption of **Cy7** but does not overlap with **Cy5**. b) An example of experimental UV-Vis spectra recorded at increasing time delays after the start of the irradiation; this example was taken to illustrate the data processing further. C) The absorbance at 735 nm (that of **Cy7**) extracted from the experimental UV-Vis spectra (graph b), the absorbance decreases linearly in time, as indicated by the linear fit of data points. Furthermore, fitting residuals are within the experimentally determined noise of the instrument, further supporting the suitability of the linear fit. d) An example of the extracted **Cy5** UV-Vis spectra from experimental data (graph b); the insert shows **Cy5** concentration *vs.* time calculated from the extracted UV-Vis spectra.

4.3. Quantum yields data

Table S1. The quantum yields *Φ* for the **Cy7** decomposition and **Cy5** formation *vs.* the concentration of **EA**

**EA** (mM)	*Φ* of **Cy7** decomposition	Standard Deviation	*Φ* of **Cy5** Formation	Standard Deviation	*Φ* (**Cy7**) / ϕ (**Cy5**)
20	1.63×10^{-4}	1.91×10^{-6}	6.70×10^{-6}	1.02×10^{-7}	24.3
100	1.15×10^{-4}	7.14×10^{-7}	7.81×10^{-6}	3.89×10^{-6}	14.7
300	1.04×10^{-4}	4.80×10^{-7}	9.09×10^{-6}	4.54×10^{-7}	11.5
500	1.18×10^{-5}	3.90×10^{-7}	1.25×10^{-5}	1.03×10^{-7}	9.4
700	9.76×10^{-5}	1.56×10^{-7}	6.51×10^{-6}	9.23×10^{-8}	14.9
1000	8.31×10^{-5}	3.40×10^{-8}	6.02×10^{-6}	3.89×10^{-6}	13.8

Conditions: 0.01 mM **Cy7**, 8.7 pH, 2% DMSO as a cosolvent.

Table S2. The quantum yields *Φ* for the **Cy7** decomposition and **Cy5** formation *vs.* concentration of **EA** in the presence of **NBA** (2 mM)

**EA** (mM)	*Φ* of **Cy7** decomposition	Standard Deviation	*Φ* of **Cy5** Formation	Standard Deviation	*Φ* (**Cy7**) / *Φ* (**Cy5**)
20	5.81×10^{-4}	3.05×10^{-6}	4.66×10^{-5}	9.03×10^{-5}	12.5
100	5.29×10^{-4}	2.23×10^{-5}	1.07×10^{-4}	9.30×10^{-5}	4.9
300	4.56×10^{-4}	2.77×10^{-5}	1.21×10^{-4}	8.43×10^{-5}	3.8
500	4.48×10^{-4}	2.34×10^{-5}	1.37×10^{-4}	5.34×10^{-5}	3.3
700	3.15×10^{-4}	2.77×10^{-5}	1.08×10^{-5}	8.37×10^{-5}	2.9
1000	2.52×10^{-4}	9.45×10^{-6}	8.34×10^{-5}	5.42×10^{-5}	3.0

Conditions: 0.01 mM **Cy7**, 8.7 pH, 2 mM **NBA**, 2% DMSO as a cosolvent.

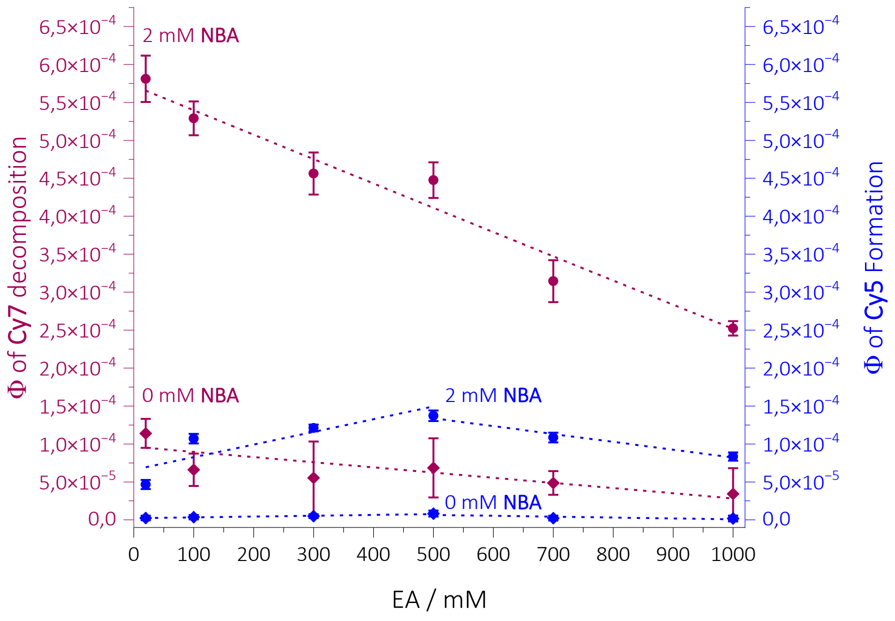

Figure S33. The quantum yields *Φ* for the decomposition of **Cy7** and the formation of **Cy5** *vs.* concentration of **EA** in the absence and presence of 2 mM **NBA** (pH was kept constant at 8.7 with 2% DMSO as cosolvent).

Table S3. The quantum yields *Φ* for the **Cy7** decomposition and **Cy5** formation *vs.* pH in the presence of 2 mM **NBA**

pH	*Φ* of **Cy7** decomposition	Standard Deviation	*Φ* of **Cy5** Formation	Standard Deviation	*Φ* (**Cy7**) / *Φ* (**Cy5**)
7.0	2.62×10^{-4}	9.87×10^{-6}	2.54×10^{-5}	2.93×10^{-5}	10.3
8.0	4.37×10^{-4}	1.34×10^{-5}	8.88×10^{-5}	4.62×10^{-5}	4.9
8.7	4.48×10^{-4}	2.34×10^{-5}	1.37×10^{-4}	5.34×10^{-5}	3.3
9.0	4.65×10^{-4}	2.05×10^{-5}	1.30×10^{-5}	6.79×10^{-5}	3.6
10.0	4.42×10^{-4}	1.68×10^{-5}	8.61×10^{-5}	6.94×10^{-5}	5.1

Conditions: 0.01 mM **Cy7**, 500 mM **EA**, pH was adjusted using AcOH, 2 mM **NBA**, 2% DMSO as a cosolvent.

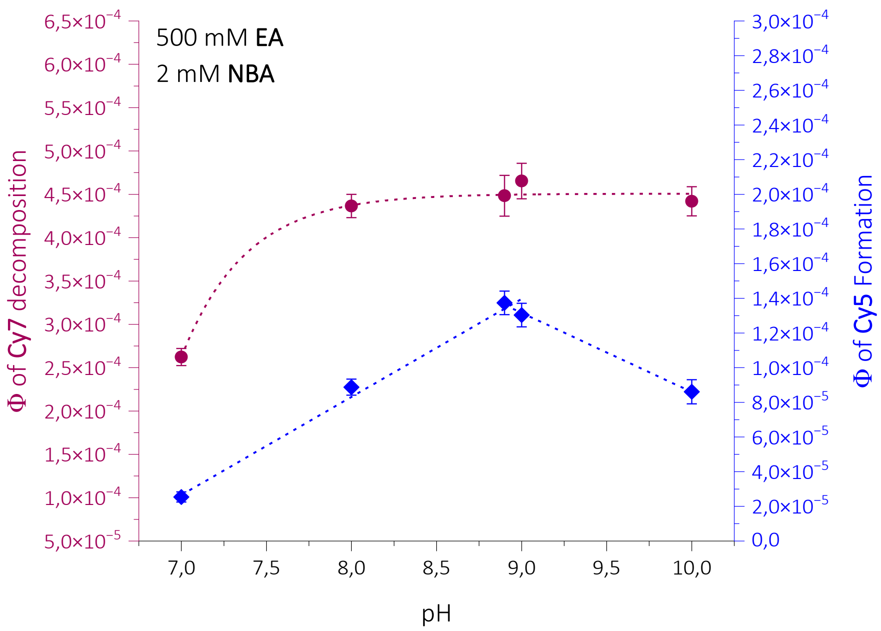

Figure S34. The quantum yields *Φ* for the decomposition of **Cy7** and the formation of **Cy5** *vs.* pH in the presence of 2 mM **NBA**. All solutions contain 500 mM **EA**, and the pH was adjusted with AcOH. All solutions contain 2% DMSO as a cosolvent.

Table S4. The quantum yields *Φ* for the **Cy7** decomposition and **Cy5** formation *vs.* **NBA** concentration

NBA (mM)	*Φ* of **Cy7** decomposition	Standard Deviation	*Φ* of **Cy5** Formation	Standard Deviation	*Φ* (**Cy7**) / *Φ* (**Cy5**)
0 [a]	1.18×10^{-5}	3.90×10^{-7}	1.25×10^{-5}	1.03×10^{-7}	9.4
0.01	8.45×10^{-5}	2.01×10^{-5}	6.96×10^{-6}	2.37×10^{-5}	12.1
0.5	2.00×10^{-4}	3.79×10^{-5}	5.62×10^{-5}	7.37×10^{-5}	3.5
2.0	4.48×10^{-4}	2.34×10^{-5}	1.37×10^{-4}	5.34×10^{-5}	3.3
5.0	6.56×10^{-4}	3.65×10^{-5}	2.07×10^{-4}	5.22×10^{-5}	2.2

Conditions: 0.01 mM **Cy7**, 500 mM **EA**, 8.7 pH, 2% DMSO as a cosolvent. [a] Value taken from Table S1, entry 4.

Table S5. The quantum yields *Φ* for the **Cy7** decomposition and **Cy5** formation with 5 mM of electron acceptor additives.

Additive	*Φ* of **Cy7** decomposition	Standard Deviation	*Φ* of **Cy5** Formation	Standard Deviation	*Φ* (**Cy7**) / *Φ* (**Cy5**)
MV^{2+}	1.04×10^{-3}	2.08×10^{-5}	3.19×10^{-4}	6.53×10^{-5}	3.3
**NBA** [a]	6.56×10^{-4}	3.65×10^{-5}	2.07×10^{-4}	5.22×10^{-5}	3.2

Conditions: 0.01 mM **Cy7**, 500 mM **EA**, 8.7 pH, 2% DMSO as a cosolvent. [a] Value taken from Table S4, entry 5.

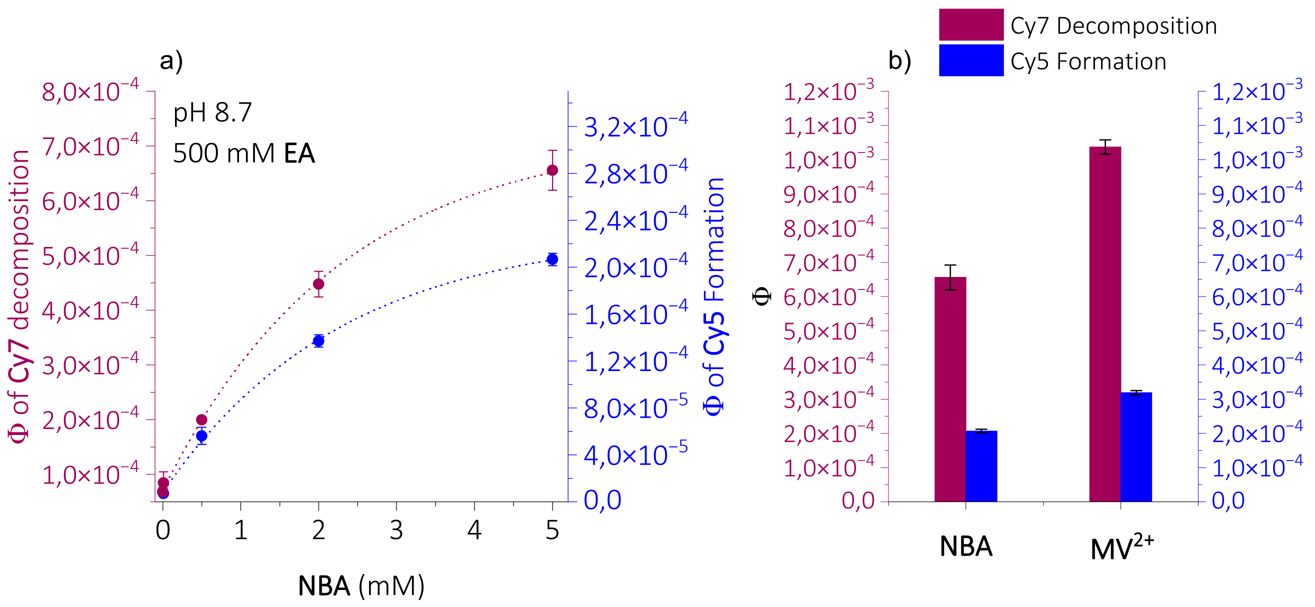

Figure S35. The influence of **NBA** and MV^{2+} on the quantum yields *Φ*; a) The quantum yields for the decomposition of **Cy7** and the formation of **Cy5** *vs.* concentration of **NBA**, and b) quantum yields with 2 mM of **NBA** or MV^{2+}. All measurements in 500 mM **EA**, pH 8.7, with 2% DMSO as a cosolvent.

Table S6. The quantum yields *Φ* for the **Cy7** decomposition and **Cy5** formation *vs.* the amount of molecular oxygen.

Conditions	*Φ* of **Cy7** decomposition	Standard Deviation	*Φ* of **Cy5** Formation	Standard Deviation	*Φ* (**Cy7**) / *Φ* (**Cy5**)
N_2 Saturated [a]	3.93×10^{-5}	3.41×10^{-6}	7.55×10^{-6}	3.16×10^{-6}	5.2
O_2:N_2 (1:1) [b]	1.06×10^{-3}	2.20×10^{-5}	3.11×10^{-4}	4.35×10^{-5}	3.4
O_2 Saturated [c]	$1,32 \times 10^{-3}$	7.07×10^{-6}	4.19×10^{-4}	4.27×10^{-5}	3.1
Standard [d,e]	4.48×10^{-4}	2.34×10^{-5}	1.37×10^{-4}	5.34×10^{-5}	3.3

Conditions: 0.01 mM **Cy7**, 8.7 pH, 500 mM **EA**, 2 mM **NBA**, 2% DMSO cosolvent. [a] Purged with N_2 for 5 min. [b] Purged with O_2/N_2 (1:1, v/v) mixture for 5 min. [c] Purged with O_2 for 5 min; [d] no additional O_2 introduced other than already dissolved in solvent; [e] the value taken from Table S2, entry 4.

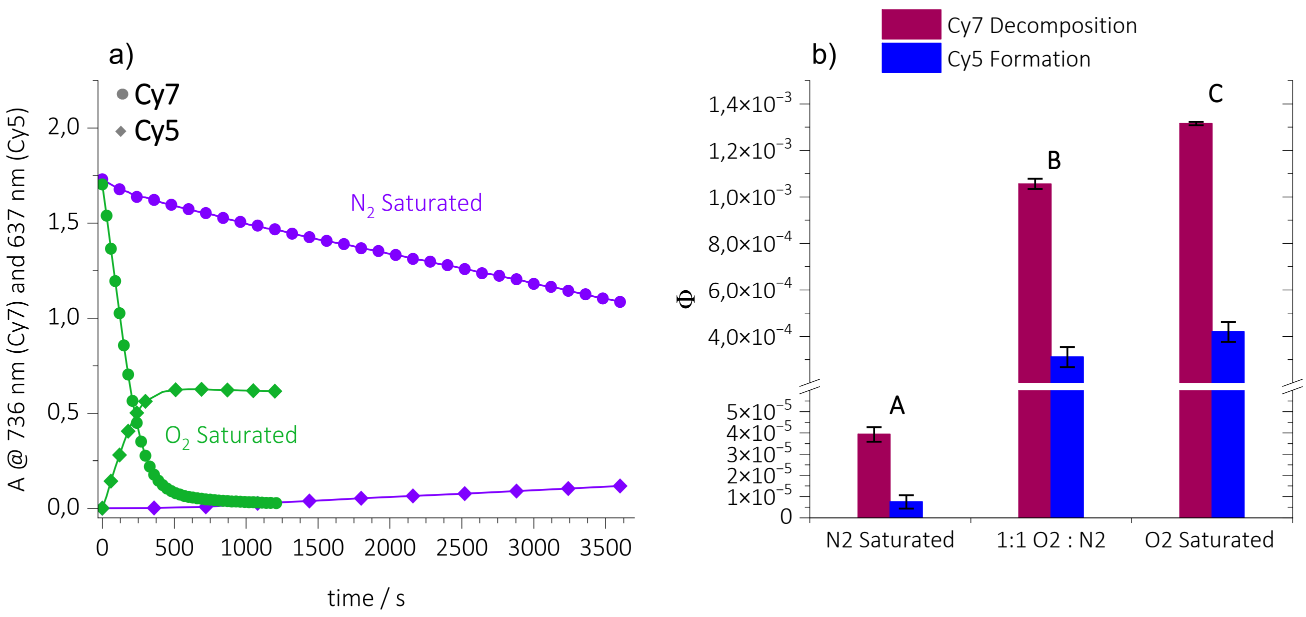

Figure S36. The influence of molecular oxygen on the quantum yields *Φ*; a) **Cy7** decomposition and **Cy5** formation rate depending on the O_2 amount, b) quantum yields for the decomposition of **Cy7** and the formation of **Cy5** vs. amount of molecular oxygen; A - solution purged with N_2 for 5 min. B – solution purged with O_2/N_2 (1:1, v:v) mixture for 5 min, C – solution purged with O_2 for 5 min. All measurements in 500 mM **EA** buffer, pH 8.7, with 2 mM **NBA** and 2% DMSO as a cosolvent.

4.3.1. Quantum yields at varying light intensity and wavelengths of irradiation

The quantum yields for the **Cy7** decomposition and **Cy5** formation as a function of light intensity were calculated from experiments shown in Figure S33.

Table S7. The quantum yields *Φ* for the **Cy7** decomposition and **Cy5** formation *vs.* the light intensity under various conditions

	LED I = 20 mA					*LED I = 50 mA*				
Conditions [a]	*Φ* of **Cy7** dec.	St. dev.	*Φ* of **Cy5** Form.	St. dev.	*Φ* (Cy7) / *Φ* (Cy5)	*Φ* of **Cy7** dec.	St. dev.	*Φ* of **Cy5** Form.	St. dev.	*Φ* (Cy7) / *Φ* (Cy5)
**EA** Buffer [b]	1.29×10^{-5}	4.05×10^{-7}	1.91×10^{-6}	6.89×10^{-8}	0.11	1.24×10^{-5}	1.78×10^{-7}	1.24×10^{-6}	4.81×10^{-7}	0.10
NaN_3 [b,c]	1.16×10^{-5}	6.18×10^{-7}	7.14×10^{-6}	1.11×10^{-7}	0.15	1.20×10^{-5}	2.29×10^{-7}	2.21×10^{-6}	6.36×10^{-7}	0.18
NBA [b,d]	4.18×10^{-4}	5.21×10^{-6}	4.80×10^{-4}	8.86×10^{-8}	0.30	3.90×10^{-4}	8.59×10^{-6}	1.15×10^{-4}	1.20×10^{-5}	0.29
NaN_3 + NBA [b,c,d]	4.02×10^{-4}	3.98×10^{-6}	3.90×10^{-4}	1.03×10^{-7}	0.40	3.83×10^{-4}	8.27×10^{-6}	1.41×10^{-4}	1.01×10^{-5}	0.37

[a] 0.01 mM **Cy7**, irradiated using 745 nm LEDs at two different driving currents (indicated next to the LED, J_0 determined using calibrated photodiode), 2% MeOH as a cosolvent, [b] 500 mM **EA** 8.7 pH, [c] NaN_3 (2 mM), [d] NBA (2 mM).

The quantum yields for the **Cy7** decomposition and **Cy5** formation at different wavelengths were calculated from experiments shown in Figures S14 to S20. Figure S37 shows the data in a graphical form while Table S8 shows the numeric values of the quantum yields.

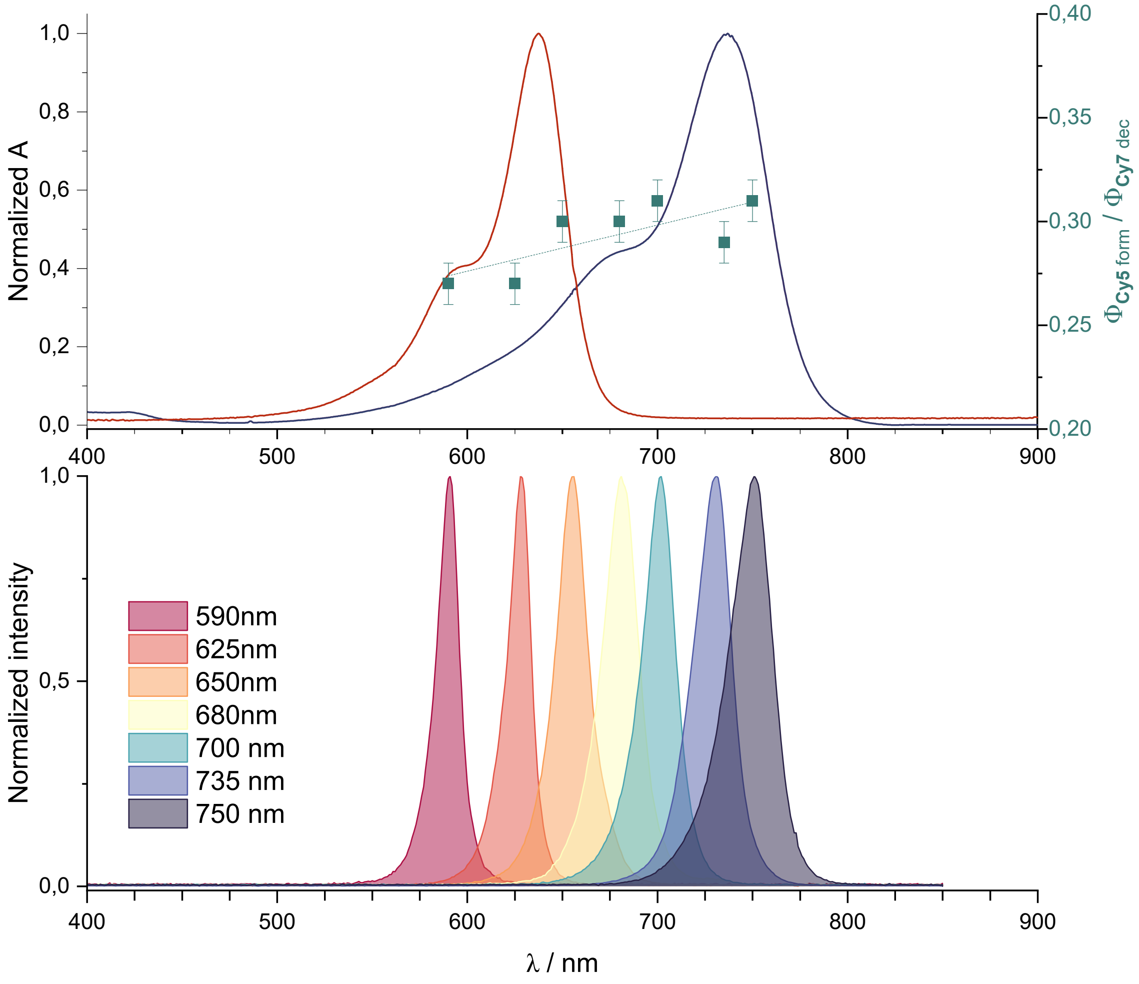

Figure S37. Overview of varying wavelengths of irradiation on phototruncation. The normalized absorption spectra of **Cy7** and **Cy5** overlapped with the quantum yield ratio (*Φ* (**Cy7**) / *Φ* (**Cy5**) and normalized emission spectra of used LEDs.

Table S8. The quantum yields *Φ* for the **Cy7** decomposition and **Cy5** formation *vs.* the irradiation wavelength

LED [a] / conditions [b]	*Φ* of **Cy7** decomposition	*Standard deviation*	*Φ* of **Cy5** Formation	*Standard deviation*	*Φ* (**Cy7**) / *Φ* (**Cy5**)
750 nm LED	4.33×10^{-4}	1.13×10^{-6}	1.36×10^{-4}	1.18×10^{-6}	0.31
735 nm LED	4.34×10^{-4}	1.83×10^{-6}	1.27×10^{-4}	1.24×10^{-6}	0.29
700 nm LED	4.23×10^{-4}	1.37×10^{-6}	1.29×10^{-4}	1.12×10^{-6}	0.31
680 nm LED	4.02×10^{-4}	1.36×10^{-6}	1.21×10^{-4}	1.14×10^{-6}	0.30
650 nm LED	4.19×10^{-4}	1.75×10^{-6}	1.25×10^{-4}	1.31×10^{-6}	0.30
625 nm LED	4.13×10^{-4}	1.75×10^{-6}	1.13×10^{-4}	1.75×10^{-6}	0.27
590 nm LED	4.03×10^{-4}	1.25×10^{-6}	1.07×10^{-4}	1.78×10^{-6}	0.27

[a] Figure S37 shows the emission spectra of the used LEDs. All LEDs were supplied by a constant current source (50 mA), and J_0 was determined using a calibrated photodiode, [b] 0.01 mM **Cy7**, **EA** buffer (500 mM **EA,** 8.7 pH, with 2 mM **NBA**) 2% MeOH as a cosolvent

4.3.2. Quantum yields for irradiation of Cy5

The quantum yields for the **Cy5** decomposition and **Cy3** formation under different reaction conditions were calculated from experiments shown in Figures S22 and S23.

Table S9. The quantum yields *Φ* for the **Cy5** decomposition and **Cy3** formation

Conditions [a]	*Φ* of **Cy7** decomposition	Standard deviation	*Φ* of **Cy5** Formation	Standard deviation	*Φ* (**Cy7**) / *Φ* (**Cy5**)
**EA** Buffer [b]	3.36×10^{-4}	5.34×10^{-5}	1.44×10^{-5}	8.64×10^{-7}	0.043
NaN_3 [b,c]	2.35×10^{-4}	3.36×10^{-5}	1.09×10^{-5}	6.86×10^{-7}	0.046

[a] 0.01 mM **Cy5**, 2% MeOH as a cosolvent, irradiated using 625 nm LEDs, [b] 500 mM **EA** 8.7 pH with **NBA** (2 mM), [c] NaN_3 (2 mM).

5. Femtosecond transient absorption spectroscopy (fs-TA)

fs-TA measurements were conducted on homebuilt 1-kHz transient absorption and femtosecond-stimulated Raman spectroscopy setups constructed around femtosecond Ti:sapphire amplifiers Femtopower (Spectra Physics) and Solstice amplifier (Spectra Physics), sharing a common oscillator. The amplifiers were synchronized by electronic triggering and an optical delay of the seed prior to amplification, allowing setting the delay between their pulses up to <1 ms with fs precision. Two laser beams, a pump and a probe, were used in the TA experiment. White light supercontinuum generated in an argon-filled hollow core fiber (Ultrafast Innovations, Savannah, USA) driven by the Femtopower amplifier served as the probe. The pump beam (centered on 735 nm, 18 nJ per pulse) was generated by an optical parametric amplifier (OPA; TOPAS, Light Conversion, Vilnius, Lithuania) driven by a Solstice amplifier. The pump and probe beams were overlapped and focused on the same spot in the sample. Each beam was interrupted on a shot-to-shot basis by an optomechanical chopper to acquire all four possible pulse combinations (pumped, not-pumped, dark background, pump-only). The spectrum of the probe beam transmitted through the sample was acquired by a home-built prism spectrometer utilizing a 1 kHz CCD camera (Entwicklungsbuero Stresing, Berlin, Germany). To reduce noise caused by white light fluctuations, we used a second identical detector to acquire a reference spectrum of the probe replica without the sample and performed a correction as described in the literature.[10] All the experiments were taken under the magic angle (54.7°) condition to remove the influence of orientation relaxation. The 361 exponentially spaced time delays from 10 fs to 0.6 ms were implemented. The sample solutions for fs-TA measurements (A = 1 ± 0.05 at 735 nm / 1 mm optical path length) were prepared by dilution of **Cy7** stock solutions (10 mM in DMSO) in the appropriate buffer mixtures. The degassed samples were prepared by the freeze-pump-thaw method; the cycles were repeated until no significant pressure change was observed (at least 4 cycles).

5.1. fs-TA Data analysis.

Our setup was designed to use 4 pulses out of each 100 and 96 to measure fs-TA and FSR spectroscopies, respectively. This setup is motivated by the much weaker signals observed in a typical FSR spectroscopy experiment compared to that of a fs-TA experiment running in the background. A dwell time of 2 min was used for each time delay between the pump and the probe, meaning that the fs-TA spectra are the result of an average of ≈4 ms (100 laser shots - 25 fs-TA measurements and 25 auxiliary measurements), and the FSR spectra are the result of an average of ≈115 s (115 000 shots). However, both the FSR spectroscopy and fs-TA methods run effectively in parallel within a single experiment. To reduce the raw data production, an on-fly algorithm was used to reject 15% of the most outstanding shots and to apply a mathematical mean to the remaining ones. The processed data from the fs-TA methods was globally fitted by the previously described homemade software[6, 11] written in Python, using a partitioned variable projection algorithm. [12-13] Coherent artifacts (cross-phase modulation, stimulated Raman scattering) resulting from the overlap of the pump and probe pulses in time and space were modeled as a Gaussian function and its derivatives.[14-15] The presented data are corrected for group velocity dispersion (chirp) by linear interpolation, but the fit was performed on the raw data.

5.2. fs-TA data

Table S10. The lifetimes τ as obtained from the global analysis fit of fs-TA data for **Cy7** in various solvents

Solvent	**EADES1** τ (ps)	**EADES2** τ (ps)	**EADES3** τ (ps)	**EADES4** τ (μs)
MeOH	1.01 ± 0.0045	3.7 ± 0.95	790 ± 1.3	22.6 ± 1.8
MeOH [a]	2.33 ± 0.0014	102 ± 2.3	861 ± 1.4	451 ± 2.1
H_2O + 2% DMSO	0.95 ± 0.0032	5.1 ± 0.34	353 ± 2.3	12.8 ± 0.9

[a] Solution degassed by freeze–pump–thaw method.

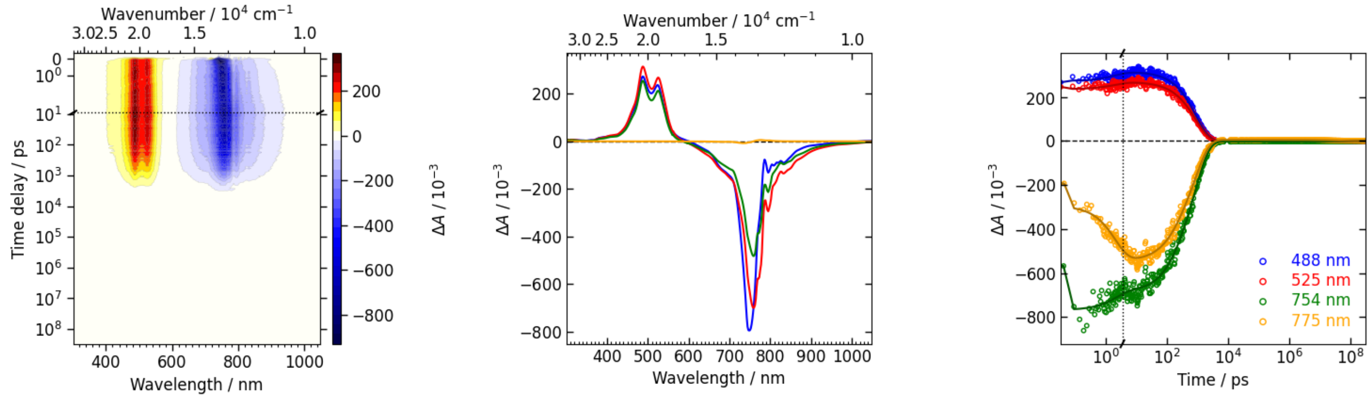

Figure S38. The fitted fs-TA data of **Cy7** in aerated MeOH.

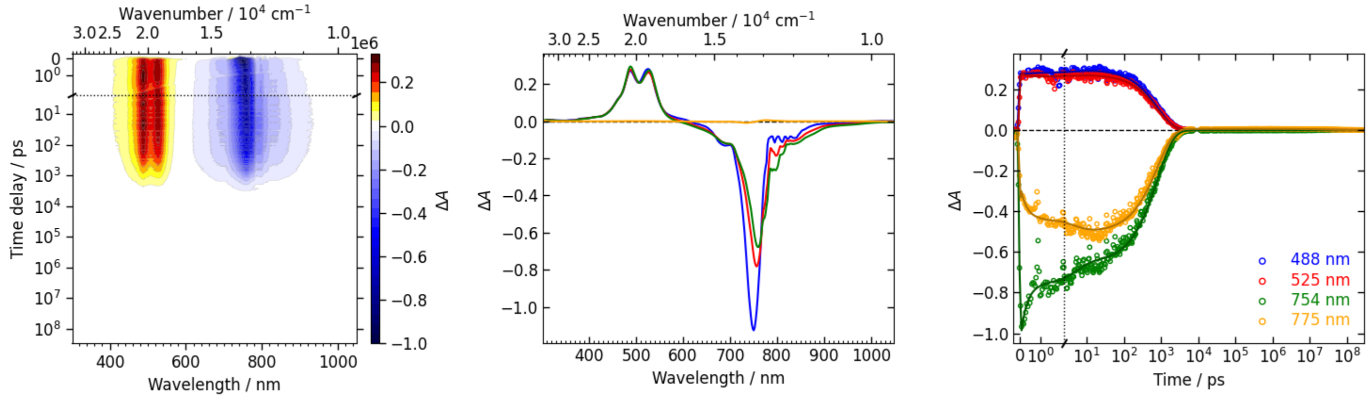

Figure S39. The fitted fs-TA data of **Cy7** in degassed (freeze – pump - thaw) MeOH.

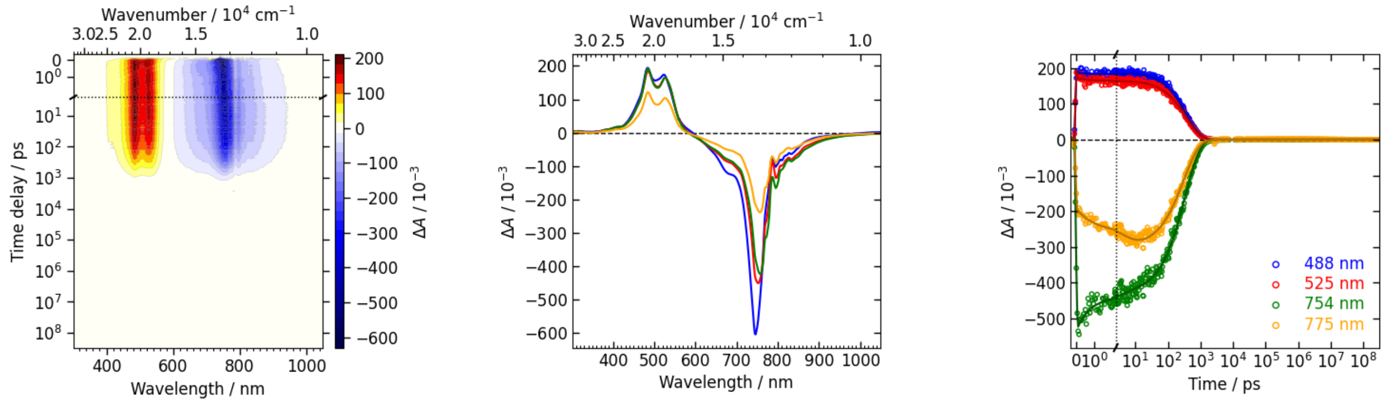

Figure S40. The fitted fs-TA data of **Cy7** in aerated water (with 2% DMSO for solubility).

Table S11. The lifetimes τ as obtained from the global analysis fit of fs-TA data for **Cy7** in **EA** buffers of various concentrations (pH maintained at 8.7 with AcOH, 2% DMSO as a cosolvent)

**EA** (mM)	EADES1 τ (ps)	EADES2 τ (ps)	EADES3 τ (ps)	EADES4 τ (µs)
0 ^{a}	0.95 ± 0.0032	5.1 ± 0.34	353 ± 2.3	12.8 ± 0.9
50	2.6 ± 0.0013	35.3 ± 1.01	364 ± 1.3	18.5 ± 1.8
100	1.6 ± 0.0045	6.37 ± 0.97	350 ± 0.9	9.28 ± 1.0
200	0.7 ± 0.0027	4.57 ± 0.65	345 ± 1.1	10.4 ± 1.9
500	1.04 ± 0.0098	6.2 ± 0.87	357 ± 1.8	7.98 ± 0.8

^{a} From Table S10, last entry.

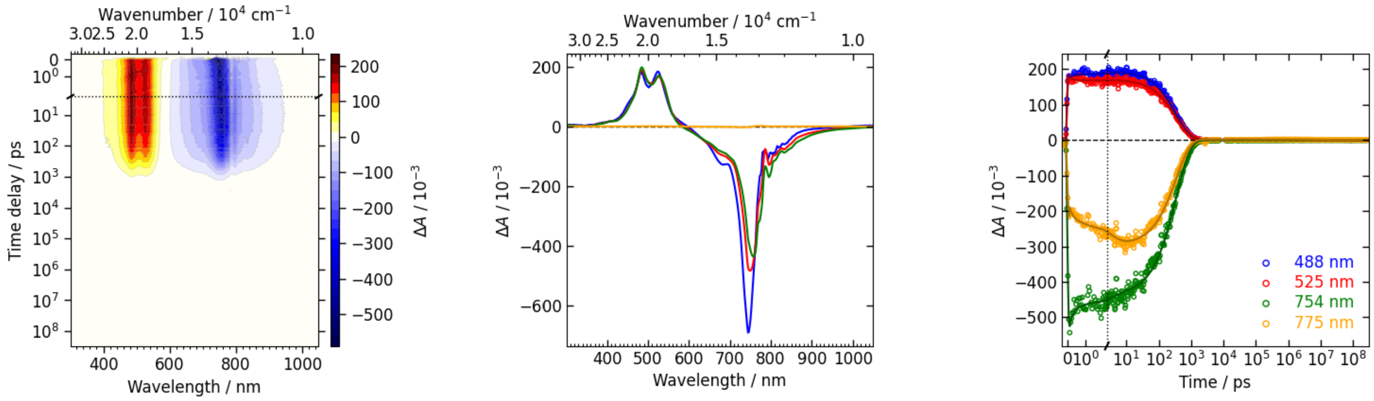

Figure S41. The fitted fs-TA data of **Cy7** in aerated 50 mM **EA** buffer (8.7 pH, with 2% DMSO for solubility)

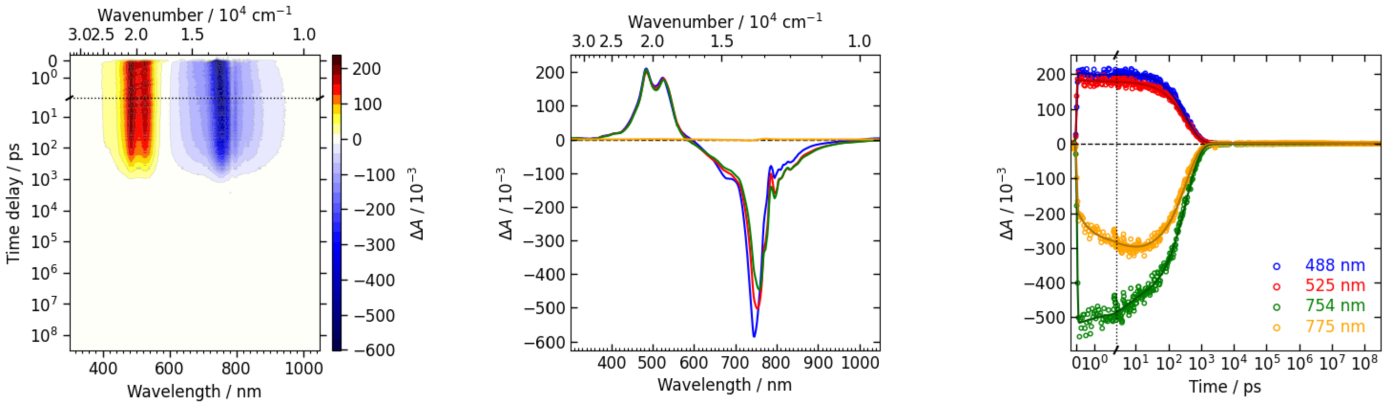

Figure S42. The fitted fs-TA data of **Cy7** in aerated 100 mM **EA** buffer (8.7 pH, with 2% DMSO for solubility)

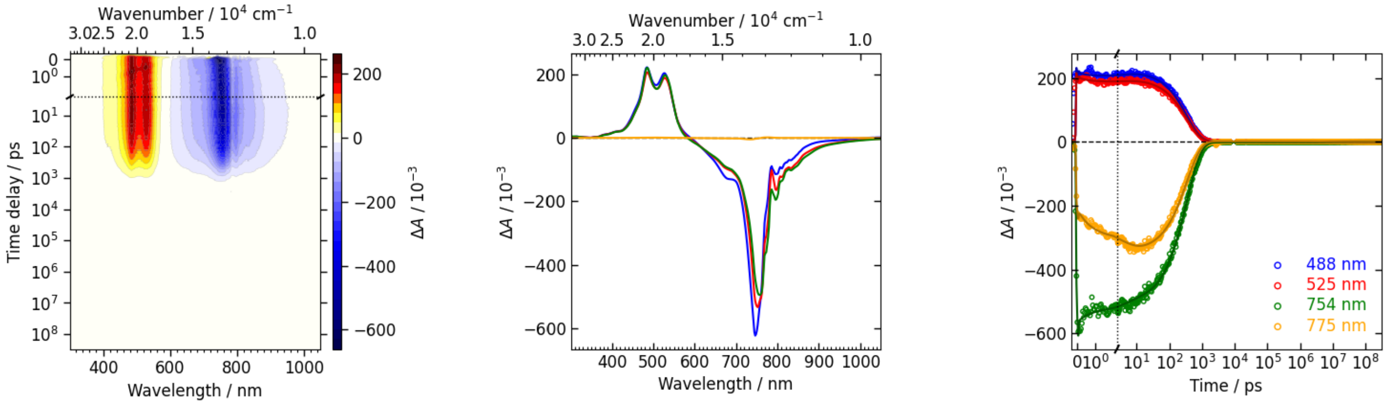

Figure S43. The fitted fs-TA data of **Cy7** in aerated 200 mM **EA** buffer (8.7 pH, with 2% DMSO for solubility)

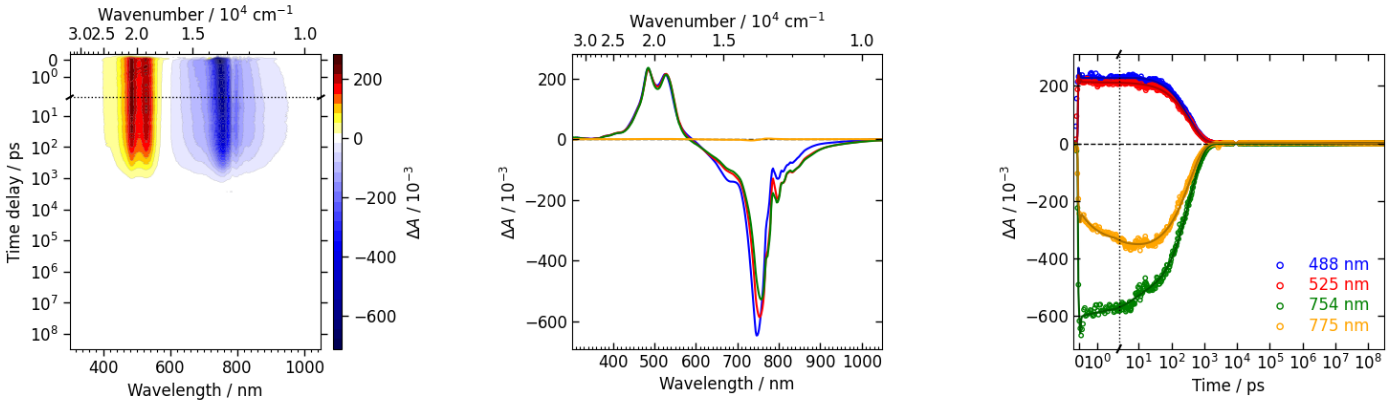

Figure S44. The fitted fs-TA data of **Cy7** in aerated 500 mM **EA** buffer (8.7 pH, with 2% DMSO for solubility)

Table S12. The lifetimes τ as obtained from the global analysis fit of fs-TA data for **Cy7** in **EA** buffers of various pH (pH adjusted with AcOH, 2% DMSO as cosolvent)

pH	EADES1 τ (ps)	EADES2 τ (ps)	EADES3 τ (ps)	EADES4 τ (μs)
7.0	0.95 ± 0.0074	5.1 ± 0.48	353 ± 1.8	12.8 ± 0.85
8.7 [a]	1.04 ± 0.0098	6.2 ± 0.87	357 ± 1.8	7.98 ± 0.8
10.0	1.02 ± 0.0087	7.7 ± 1.01	359 ± 1.9	11.2 ± 1.9

[a] From Table S11, last entry.

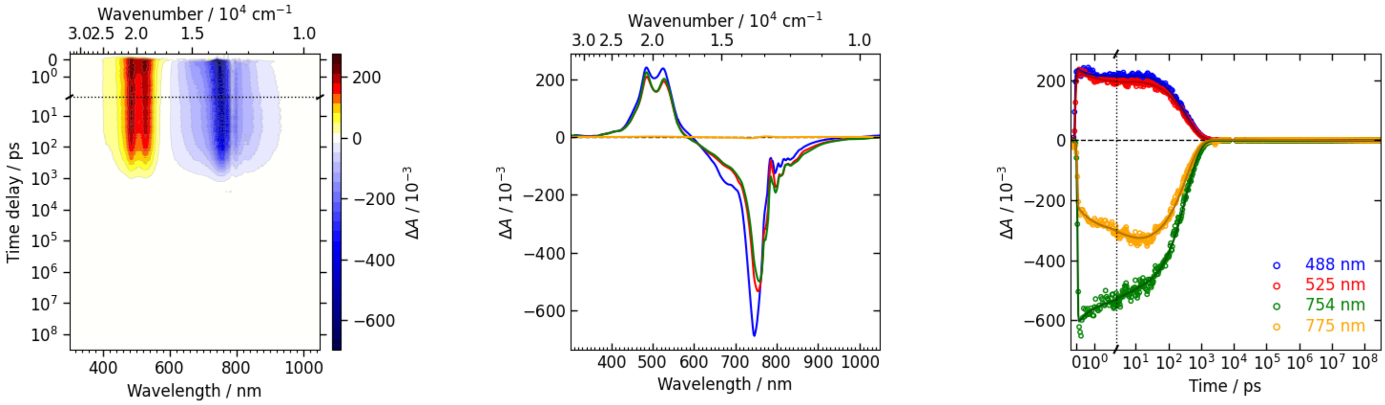

Figure S45. The fitted fs-TA data of **Cy7** in aerated 500 mM **EA** buffer with 7.0 pH (2% DMSO for solubility)

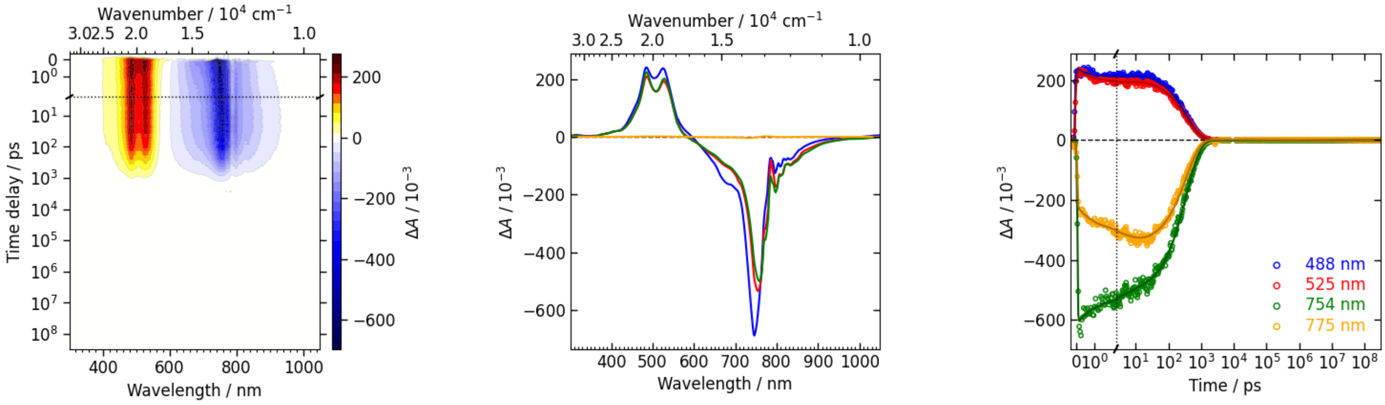

Figure S46. The fitted fs-TA data of **Cy7** in aerated 500 mM **EA** buffer with 10.0 pH (2% DMSO for solubility)

Table S13. The lifetimes τ as obtained from the global analysis fit of fs-TA data for **Cy7** in **EA** buffer with the addition of **NBA** (500 mM **EA** buffer, pH 8.7, 2% DMSO as a cosolvent)

**NBA** (mM)	EADES1 τ (ps)	EADES2 τ (ps)	EADES3 τ (ps)	EADES4 τ (μs)
0 [a]	1.04 ± 0.0098	6.2 ± 0.87	357 ± 1.8	7.98 ± 0.8
0.5	0.9 ± 0.0049	4.8 ± 0.76	344 ± 1.1	7.18 ± 0.9
2	0.54 ± 0.0084	6.5 ± 0.68	380 ± 1.3	29.1 ± 2.1
2 [b]	0.52 ±0.0075	4.7 ± 0.65	355 ± 1.9	8.47 ± 0.9

[a] From Table S11, last entry. [b] Solution degassed by freeze–pump–thaw method.

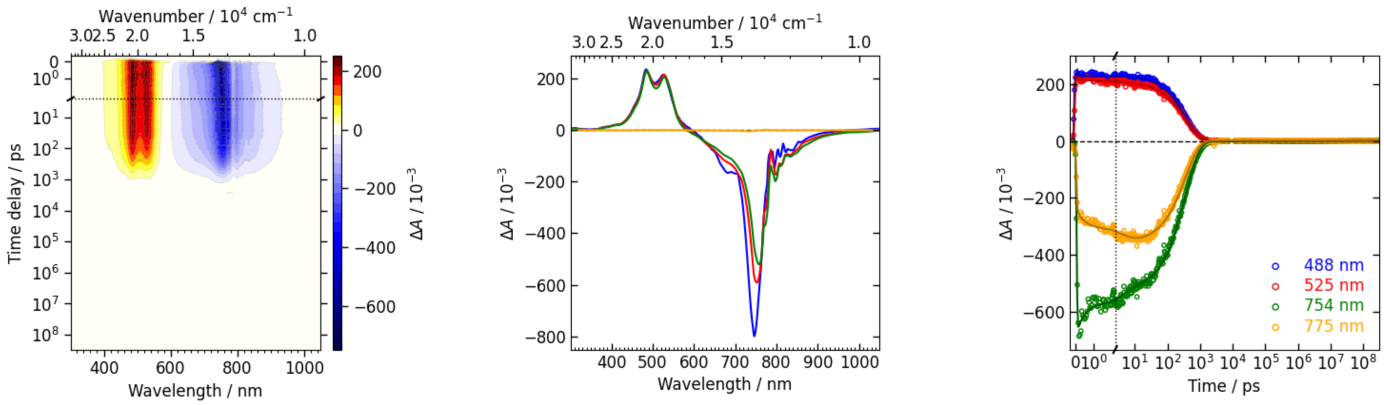

Figure S47. The fitted fs-TA data of **Cy7** in aerated 500 mM **EA** buffer with 0.5 mM **NBA** (8.7 pH, 2% DMSO for solubility)

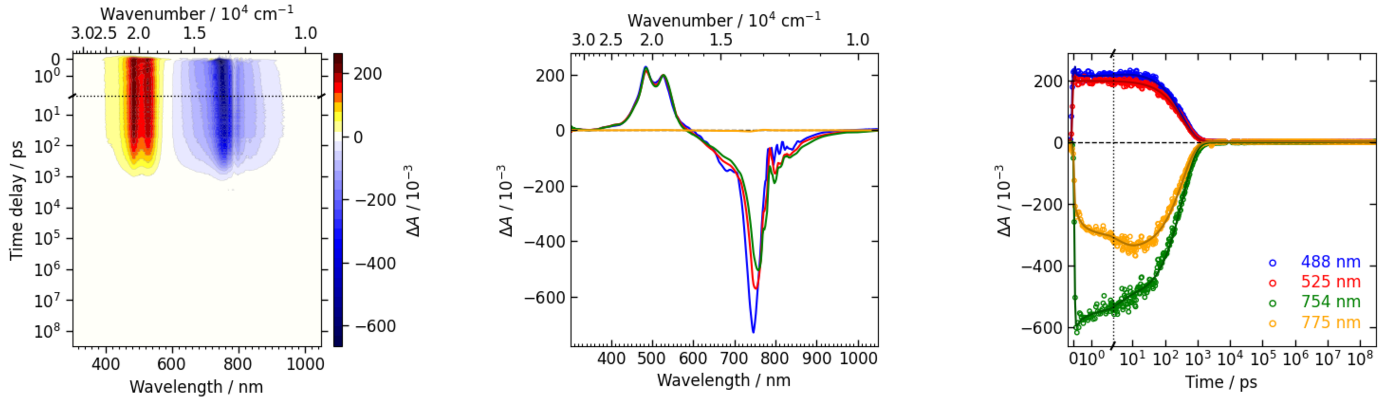

Figure S48. The fitted fs-TA data of **Cy7** in aerated 500 mM **EA** buffer with 2 mM **NBA** (8.7 pH, 2% DMSO for solubility)

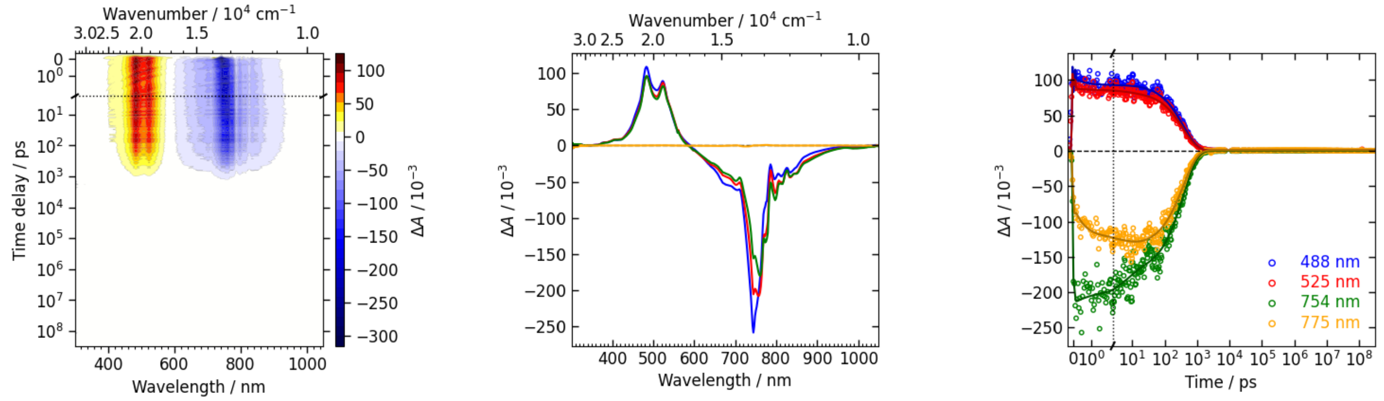

Figure S49. The fitted fs-TA data of **Cy7** in aerated 500 mM **EA** buffer with 2 mM **NBA**, solution degassed by freeze–pump–thaw method (8.7 pH, 2% DMSO for solubility).

6. Femtosecond stimulated Raman spectroscopy (FSR spectroscopy)

A femtosecond-stimulated Raman spectroscopy setup is an upgraded version[16] of the one described in our previous work.[17] In brief, in the current design, we seeded two independent 1 kHz chirped pulse amplifiers (CPAs) with fs pulses from one shared Ti:sapphire oscillator. The seed pulses were delayed electronically and optically to trace processes beyond six ns. The 735 nm pulses (150 nJ) from OPA driven by a Solstice amplifier, focused into a 100 µm spot, were used as the actinic pump (phototrigger) with ≈50 fs (full-width half maximum) pulse duration. Meanwhile, by focusing a 1450 nm signal beam from a second OPA system on a moving CaF_2 plate, we generated a white-light supercontinuum as a probe, and the probe was focused on the sample at a spot of approximately 50 µm. In the detection apparatus, the probe was split into two beams. One part was sent to a grating-based high-resolution imaging spectrograph (Acton, Princeton Instruments) for Raman analysis in the 750–950 nm region. The other part was directed to a prism spectrograph to obtain transient absorption spectra in the 370–1200 nm range. In both spectrographs, a 58 × 1024 pixels CCD camera (Entwicklungsbuero Stresing) was used as a linear image sensor via operation in a full vertical binning mode. The cameras were triggered from the lasers at 1 kHz and provided shot-to-shot detection. The 800 nm fs pulses from the second amplifier passed through a home-built pulse shaper to create a series of frequency-locked ps pulses as the Raman pump, with a total of 96 wavelength-shifted Raman pumps. The energy of the Raman pump was 2 µJ. We implemented 98 exponentially spaced time delays from 10 fs to 51.2 µs to sample the photoinduced dynamics. All the experiments were taken under the magic-angle (54.7°) condition to remove the influence of orientation relaxation. To reduce the impact of photodamage, we moved the sample in the beam at a speed of approximately 10 cm s^{-1} in a sample scanner in the case of degassed samples. For aerated samples, a peristaltic pump and flow cuvette were used to continuously flow the sample through the beam path to reduce photoinduced damage. The sample solutions for FSR measurements were prepared in an identical manner to those in fs-TA measurements. The path length was 1 mm, and the sample absorbance at the excitation wavelength was ≈1 ± 0.05 (with a 1-mm optical path length). All steady-state stimulated Raman spectra were taken with the actinic pulse off.

6.1. Target analysis

FSR spectroscopy data analysis in Origin – Target analysis: The target analysis of FSR spectra was performed according to the previously published work[3] briefly, a custom function: Y = A×C1 + B×C2 + C×C3 + D×C4 + E×C5 + F×C6 was created in OriginPro2023 (64-bit), 10.0.0.154 (Academic), OriginLab Corporation software (in the same way as described in Quantum Yield Measurements). The Y represents an experimental FSR spectrum at the given time. A, B, C, D, E, and F are the extracted (constructed) transient Raman spectra of ***Z*3-Cy7**, ***Z*4-Cy7**, S_1, T_1, hot S_1, and **Cy7$^{\bullet+}$** species, respectively (as described in [3]). The C1–C6 are the associated coefficients (output of the fit), *i.e.*, the values by which the transient Raman spectra (A to F) are multiplied to make the final sum equal to the experimental FSR spectrum. The Levenberg-Marquardt iteration algorithm was used with a maximum of 500 iterations and a 1×10^{-12} tolerance set as a default. If necessary, the Savitzky-Golay smoothing (points of window: 15, polynomial order: 2) was performed on the raw FSR spectra. The photophysical/photochemical model for the temporal evolution of species was created using the Bounds option in the function dialogue by setting the range in which the C1–C6 coefficients have positive values *via* the Higher bounds values set to >0. The lower Bounds were fixed to 0 for C1–C6. The model presented in Figure S50 and as described in [3] was applied to the experimental data. The output coefficients C1–C6 were fitted with an exponential fit (ExpGrowDec function) to obtain the approximate lifetimes of the species (Figures S51 – S56).

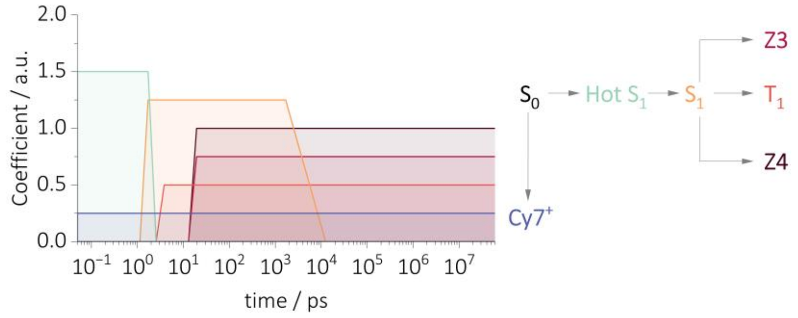

Figure S50. The target analysis model used to fit the experimental FSR data.

6.2. FSR Spectroscopy data

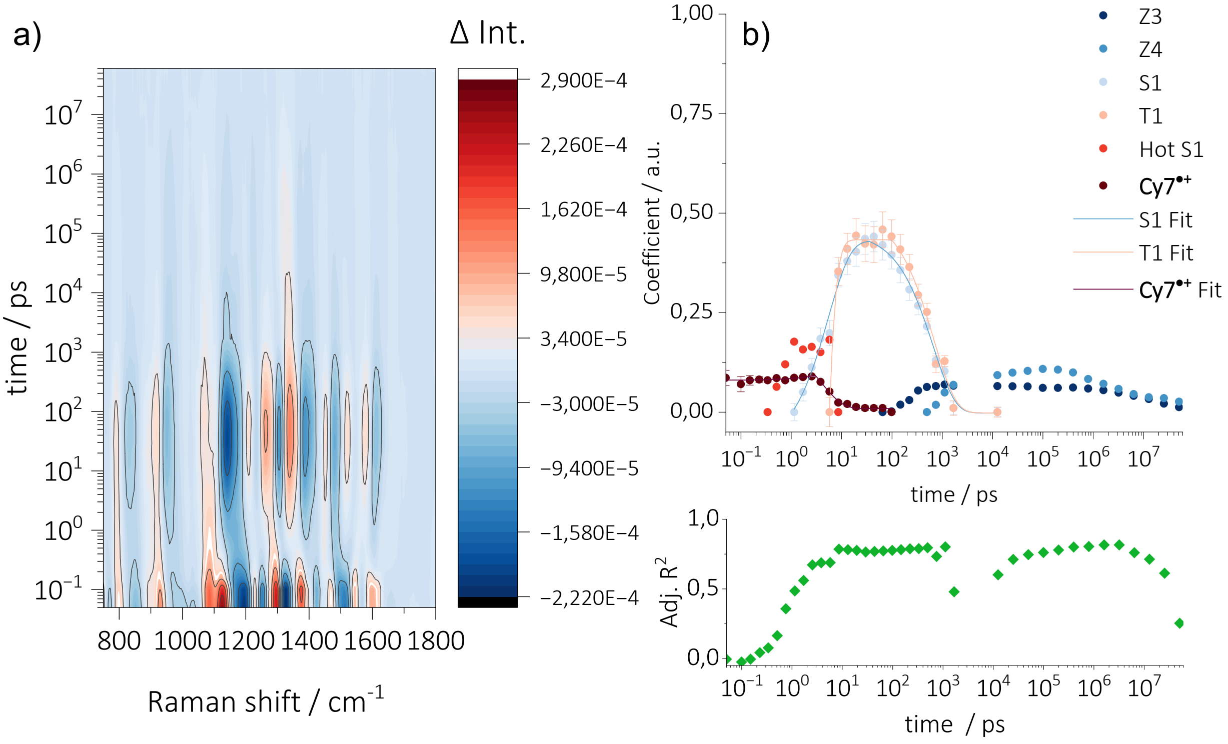

Figure S50. a) The experimental FSR spectra of **Cy7** in aerated solution (50 mM **EA**, 8.7 pH, 2% MeOH as a cosolvent) presented as the contour graph. b) The output of the target analysis model (Figure S50) upon fitting the experimental FSR data with R^2 shown at the bottom of the figure. The solid lines show the fit of model output coefficients with *ExpGrowDec* function to extract lifetimes of **Cy7**$^{•+}$, S_1, and T_1.

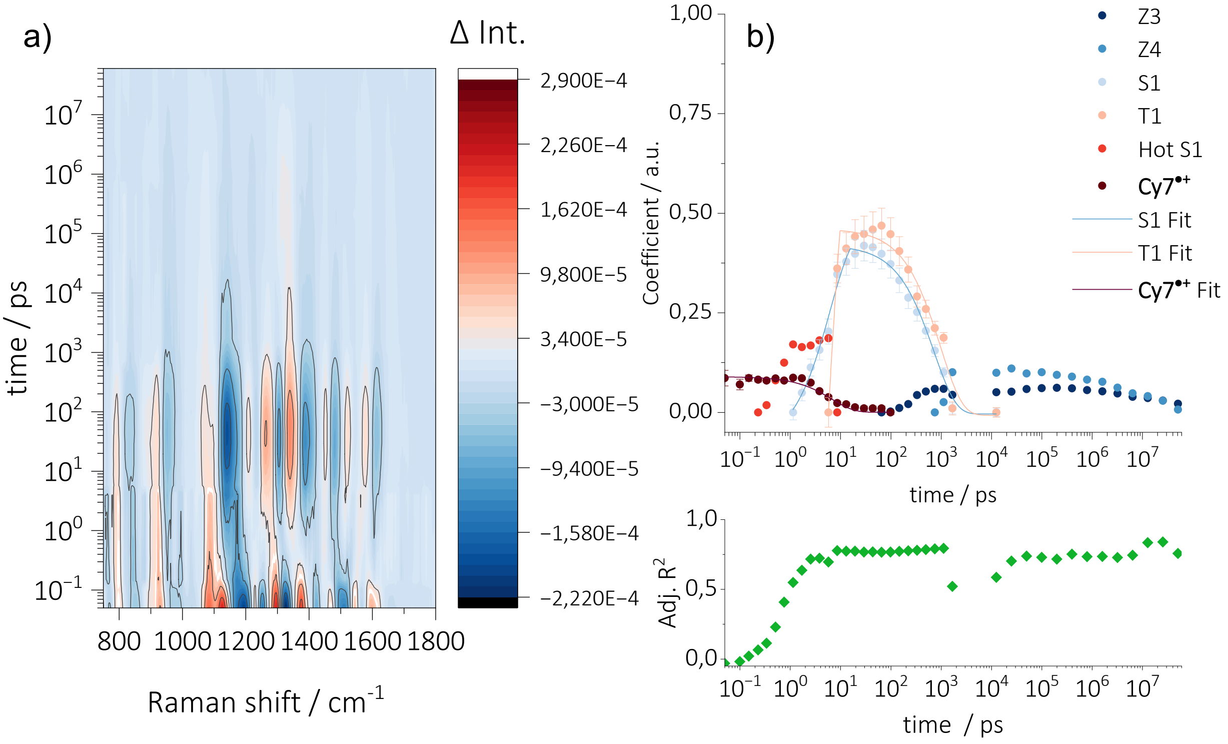

Figure S51. a) The experimental FSR spectra of **Cy7** in aerated solution (100 mM **EA**, 8.7 pH, 2% MeOH as a cosolvent) presented as the contour graph. b) The output of the target analysis model (Figure S50) upon fitting the experimental FSR data with R^2 shown at the bottom of the figure. The solid lines show the fit of the model output coefficients with *ExpGrowDec* function to extract lifetimes of **Cy7**$^{•+}$, S_1, and T_1.

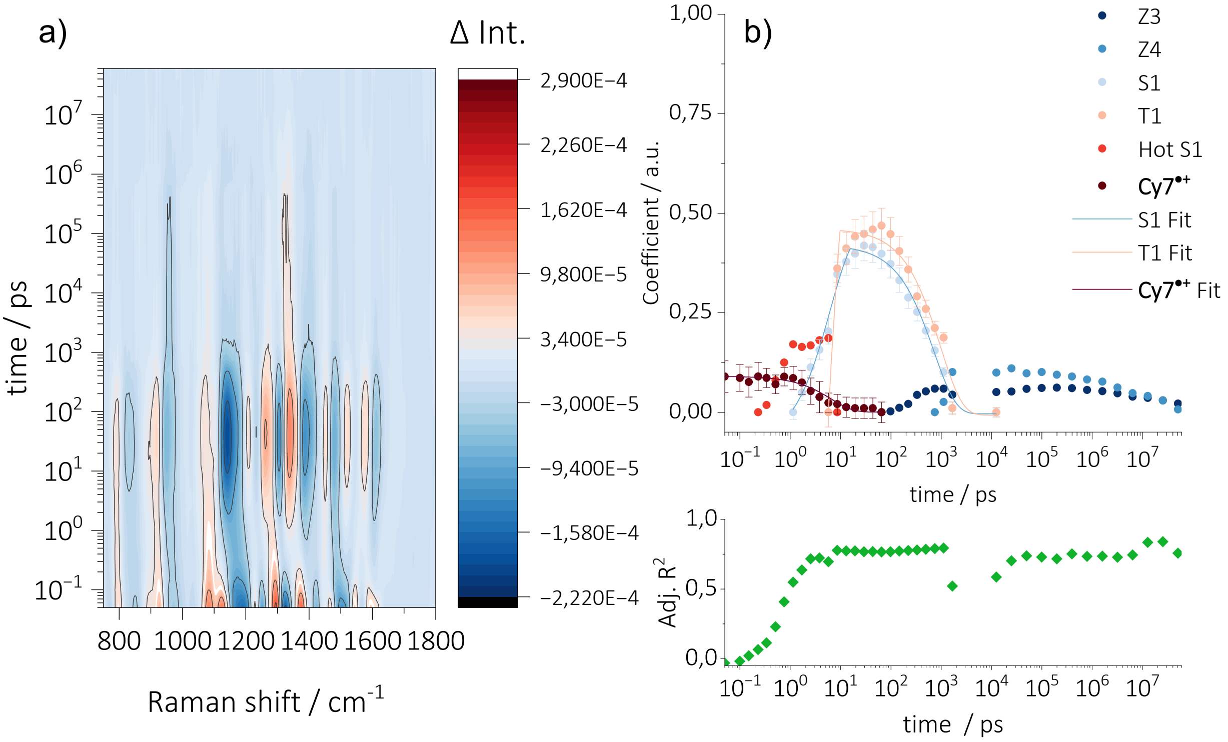

Figure S52. a) The experimental FSR spectra of **Cy7** in aerated solution (200 mM **EA**, 8.7 pH, 2% MeOH as a cosolvent) presented as the contour graph. b) The output of the target analysis model (Figure S50) upon fitting the experimental FSR data with R^2 shown at the bottom of the figure. The solid lines show the fit of the model output coefficients with *ExpGrowDec* function to extract lifetimes of **Cy7$^{•+}$**, S_1, and T_1.

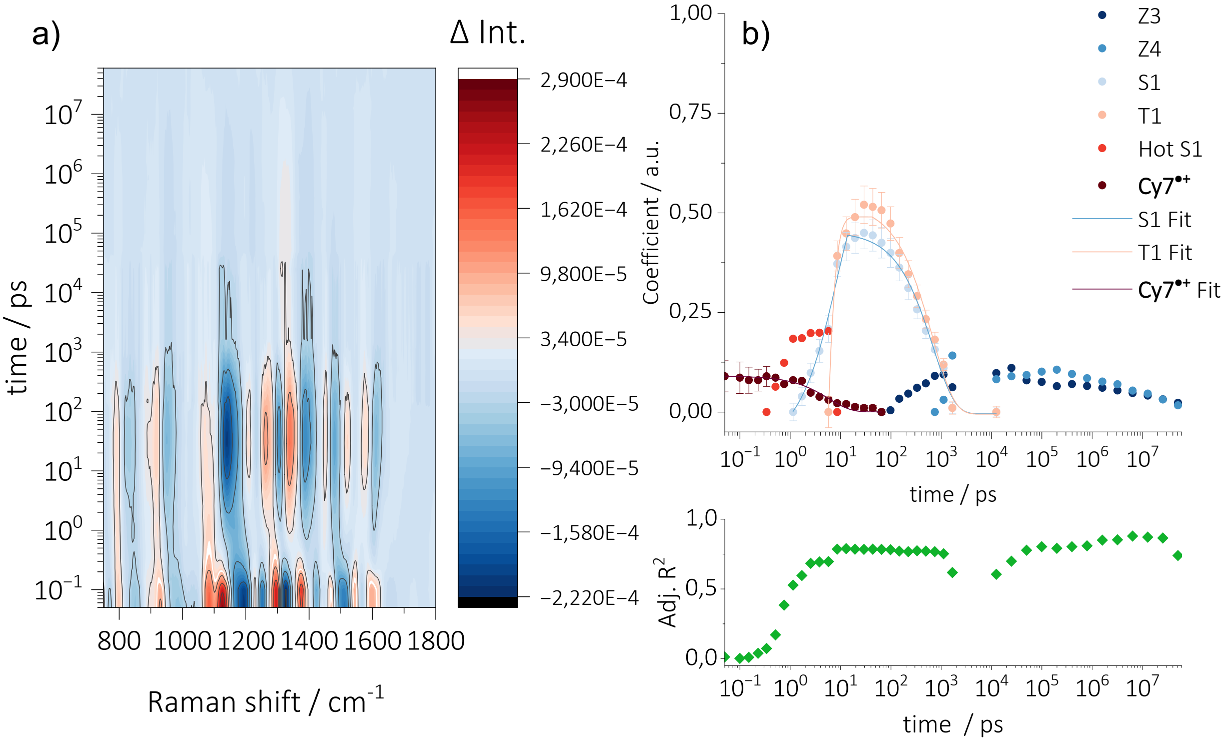

Figure S53. a) The experimental FSR spectra of **Cy7** in aerated solution (500 mM **EA**, 8.7 pH, 2% MeOH as a cosolvent) presented as the contour graph. b) The output of the target analysis model (Figure S50) upon fitting the experimental FSR data with R^2 shown at the bottom of the figure. The solid lines show the fit of the model output coefficients with *ExpGrowDec* function to extract lifetimes of **Cy7$^{•+}$**, S_1, and T_1.

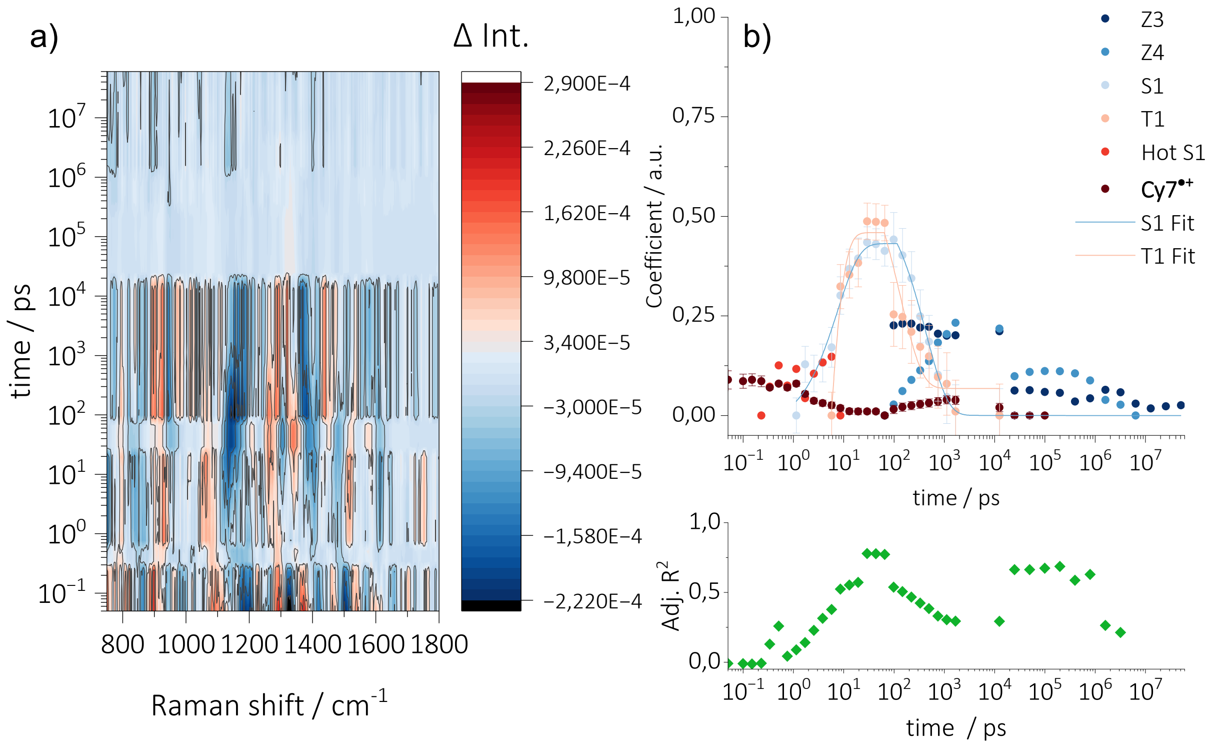

Figure S54. a) The experimental FSR spectra of **Cy7** in aerated solution (500 mM **EA**, 8.7 pH, 2 mM **NBA**, 2% MeOH as a cosolvent) presented as the contour graph. b) The output of the target analysis model (Figure S50) upon fitting the experimental FSR data (the R^2 is significantly lower than in other fits, due to a much higher noise in the data set). The solid lines show the fit of the model output coefficients with *ExpGrowDec* function to extract lifetimes of **Cy7$^{•+}$**, S_1, and T_1.

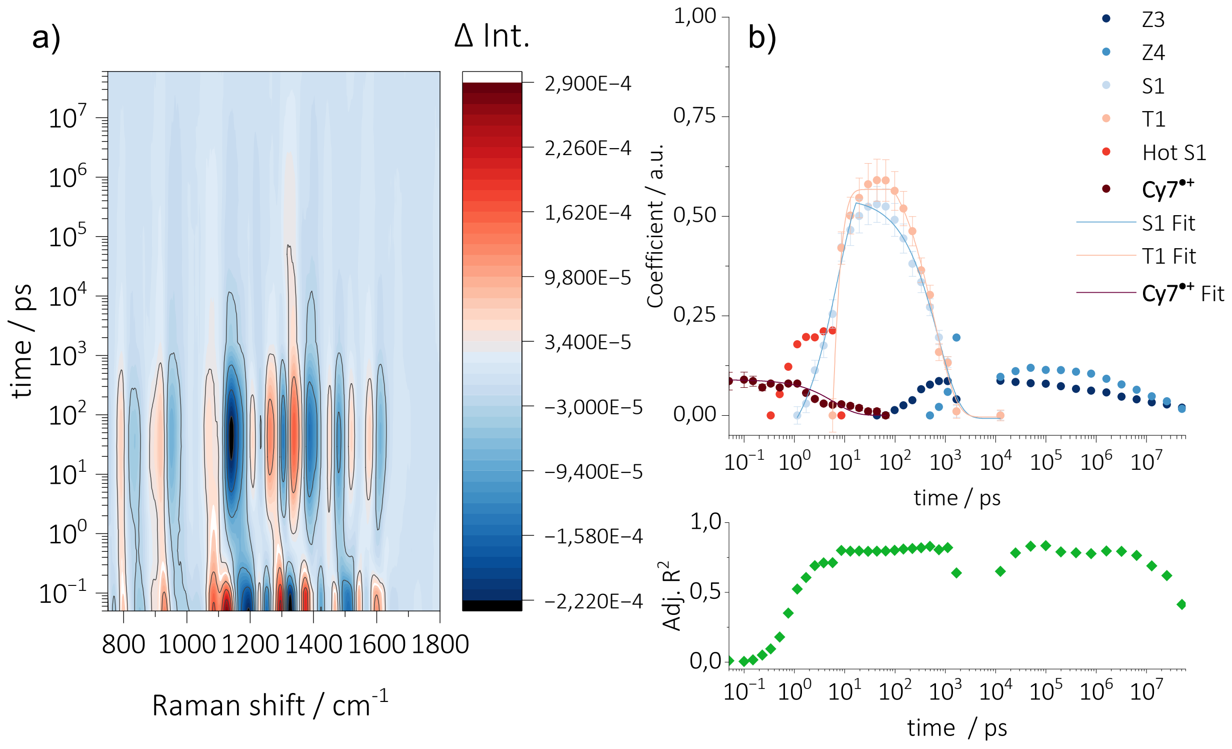

Figure S55. a) The experimental FSR spectra of **Cy7** in aerated solution (500 mM **EA**, 7.0 pH, 2% MeOH as a cosolvent) presented as the contour graph. b) The output of the target analysis model (Figure S50) upon fitting the experimental FSR data with R^2 shown at the bottom of the figure. The solid lines show the fit of the model output coefficients with *ExpGrowDec* function to extract lifetimes of **Cy7$^{•+}$**, S_1, and T_1.

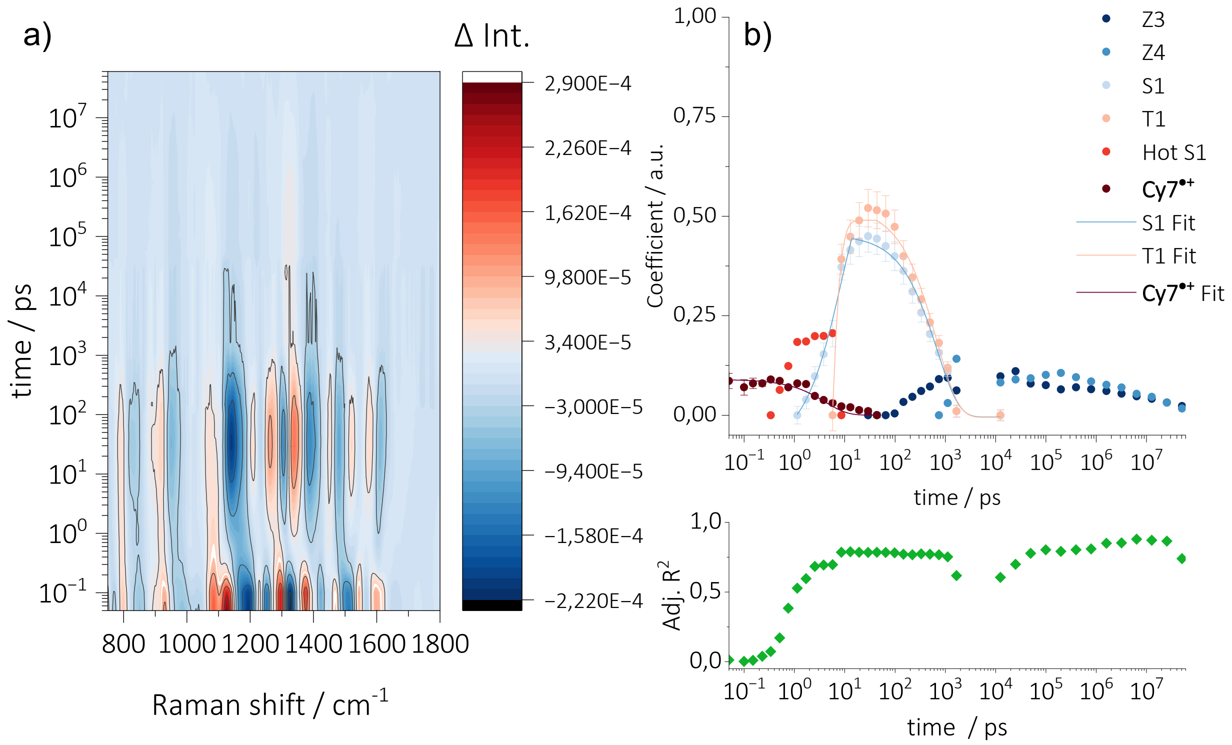

Figure S56. a) The experimental FSR spectra of **Cy7** in aerated solution (500 mM **EA**, 10.0 pH, 2% MeOH as a cosolvent) presented as the contour graph. b) The output of the target analysis model (Figure S50) upon fitting the experimental FSR data with R^2 shown at the bottom of the figure. The solid lines show the fit of the model output coefficients with *ExpGrowDec* function to extract lifetimes of **Cy7$^{\bullet+}$**, S_1, and T_1.

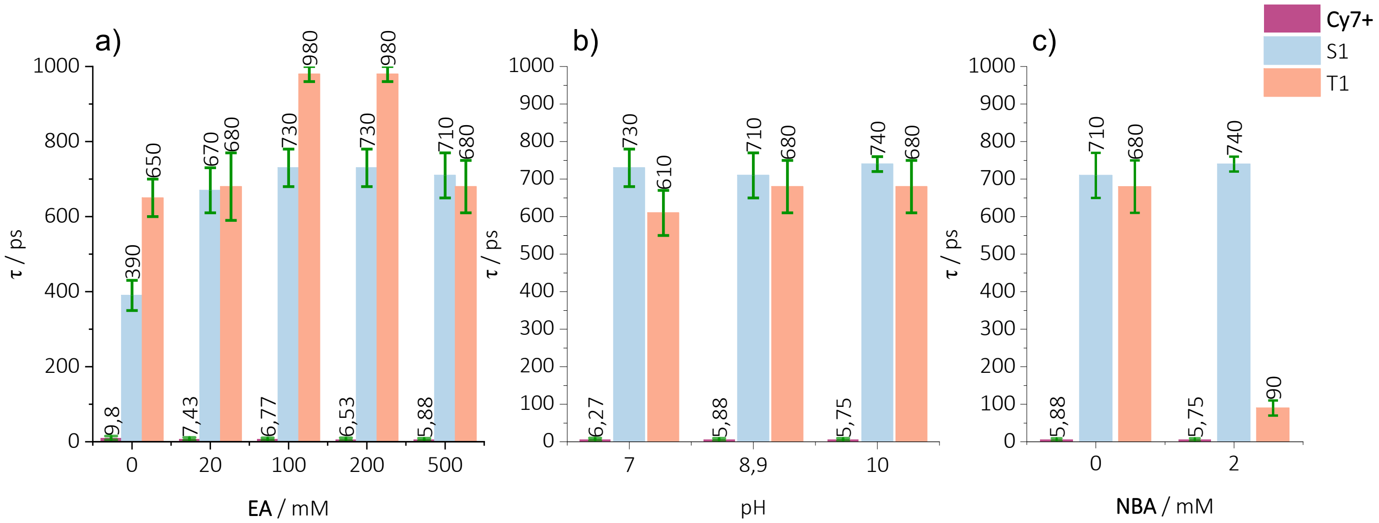

Figure S57. The extracted lifetimes of **Cy7$^{\bullet+}$**, S_1 and T_1 from FSR data, *vs.* a) **EA** concentration (the pH of all solution was adjusted to 8.7, the first point, **EA** = 0 mM was taken from[3], b) pH (all solutions contain 500 mM **EA**, the pH was adjusted with AcOH), and c) addition of **NBA** (all solutions contain 500 mM **EA**, pH of 8.7).

6.3. Superoxide radical anion

The 1050–1160 cm^{-1} region of the processed experimental FSR spectra recorded under aerated conditions showing the bands attributed to the solvated hot S_1 state of **Cy7**, solvated radical dication (**Cy7$^{•+}$**), and the superoxide radical anion ($O_2^{•-}$; at 1147 cm^{-1}, filled red band). The band assignment was done according to our previously published procedure.[3]

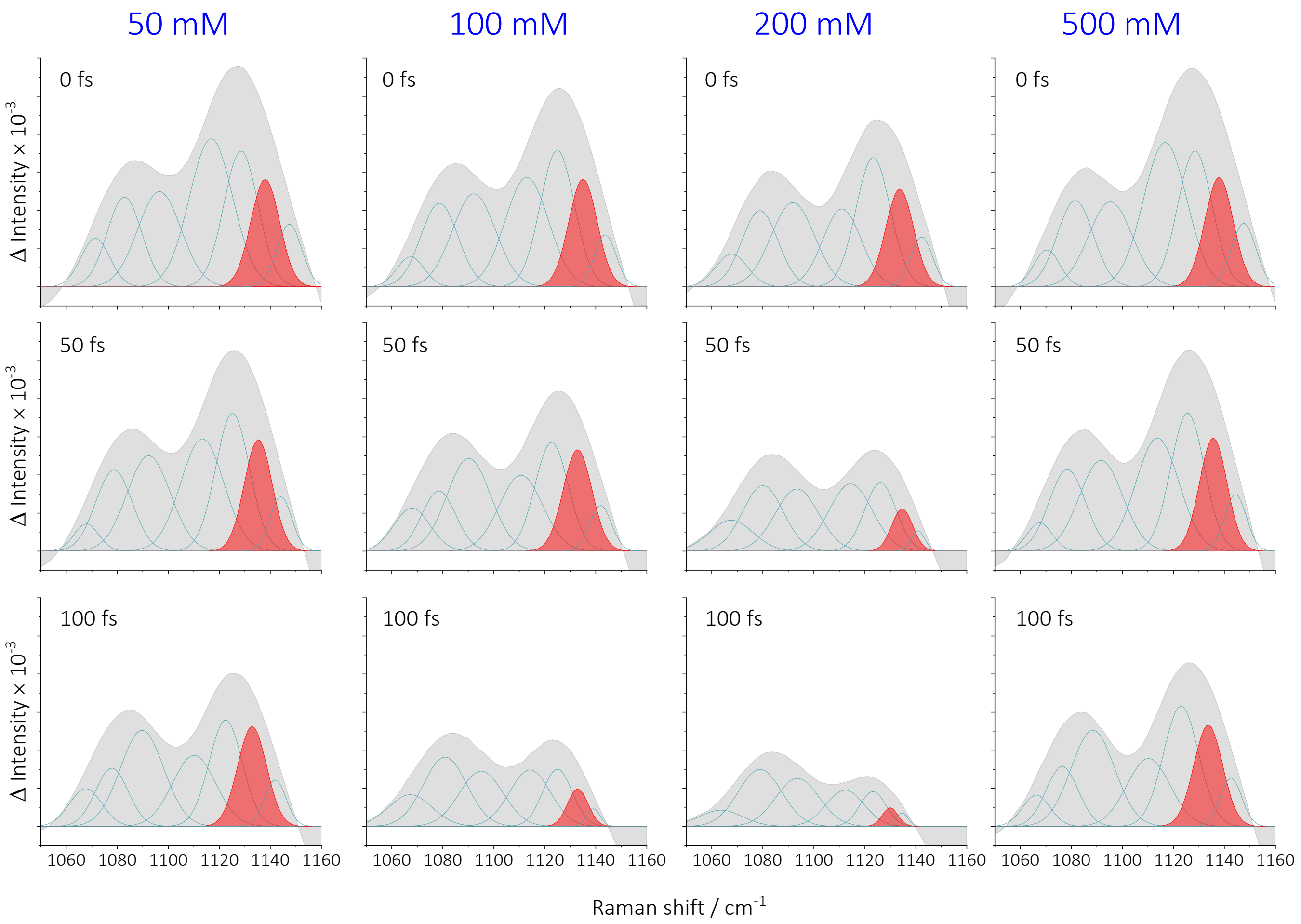

Figure S58. The superoxide radical anion band from the FSR spectra vs. concentration of **EA** (**EA** buffer, pH = 8.7, with 2% MeOH for solubility). Experimental (gray filled line) FSR spectrum at the time delays noted above each spectrum, fitted with seven Gaussian peaks, assigned to superoxide radical anion (red filled line), solvated hot-S_1 **Cy7** state, and solvated **Cy7$^{•+}$** (cyan lines).

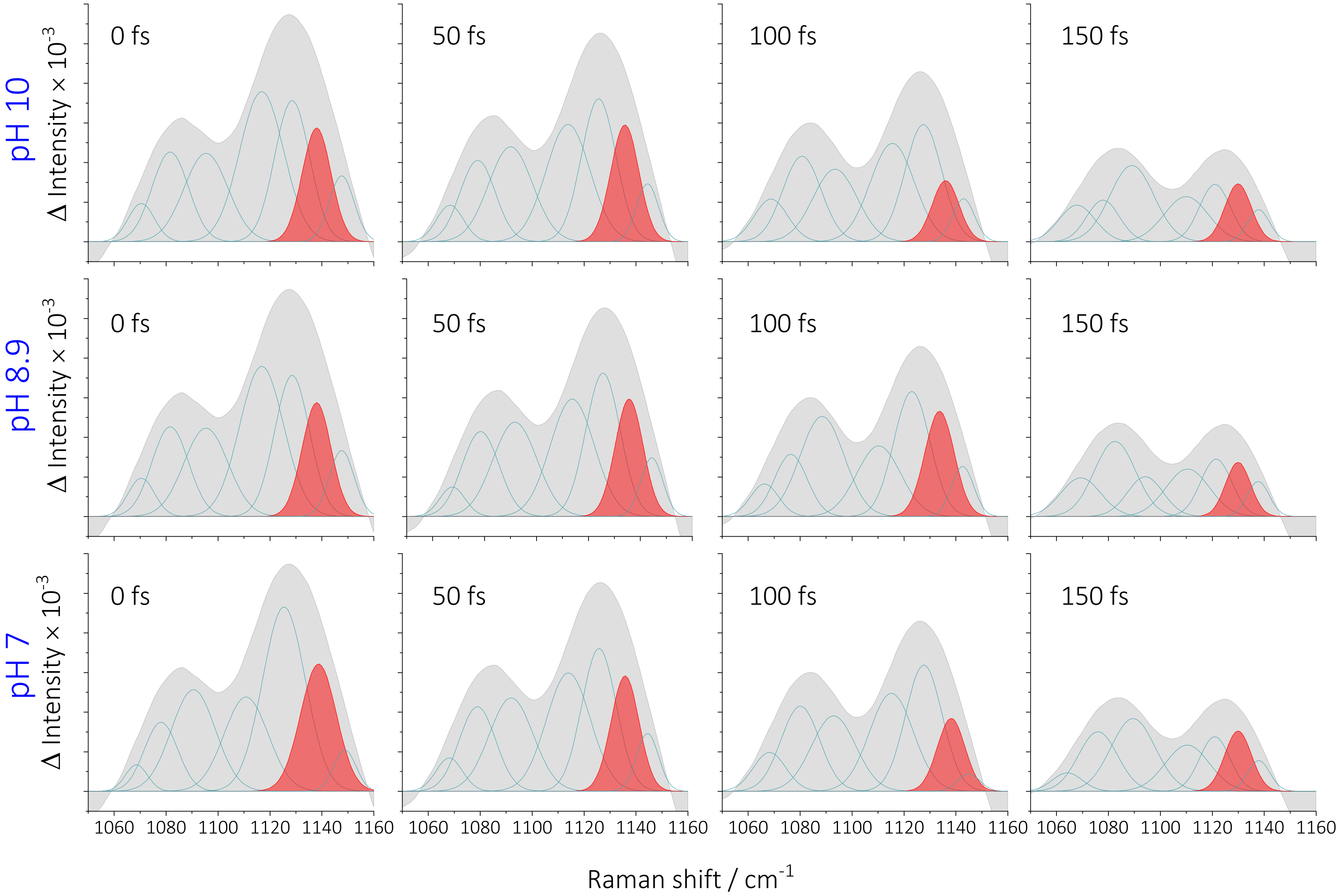

Figure S59. The superoxide radical anion band vs. pH of **EA** buffer (500 mM, with 2% MeOH for solubility). Experimental (Gray filled line) FSR spectrum at the time delays noted above each spectrum, fitted with seven Gaussian peaks, assigned to superoxide radical anion (red filled line), solvated hot-S_1 **Cy7** state, and solvated **Cy7$^{\bullet+}$** (cyan lines).

7. Cyclic voltammetry – Redox potential analysis

Cyclovoltammetry measurements were performed on an Autolab PGSTAT12 potentiostat (Eco Chemie BV, The Netherlands) connected to a PC running the software package Nova 2.1.4 (Metrohm, The Netherlands) in degassed DMF as a solvent containing 0.1 mol L^{-1} tetrabutylammonium perchlorate ($TBAClO_4$) as electrolyte employing Ag wire as a pseudo reference electrode, Pt wire as the counter electrode and a working electrode. Ferrocene was added to the solution after recording the CV of the compound, and the measurement was repeated to reference the Ag electrode. The $E_{1/2}$ potential of ferrocene-ferrocenium (Fc/Fc^+) couple was in good agreement with the literature value in all cases.[18]

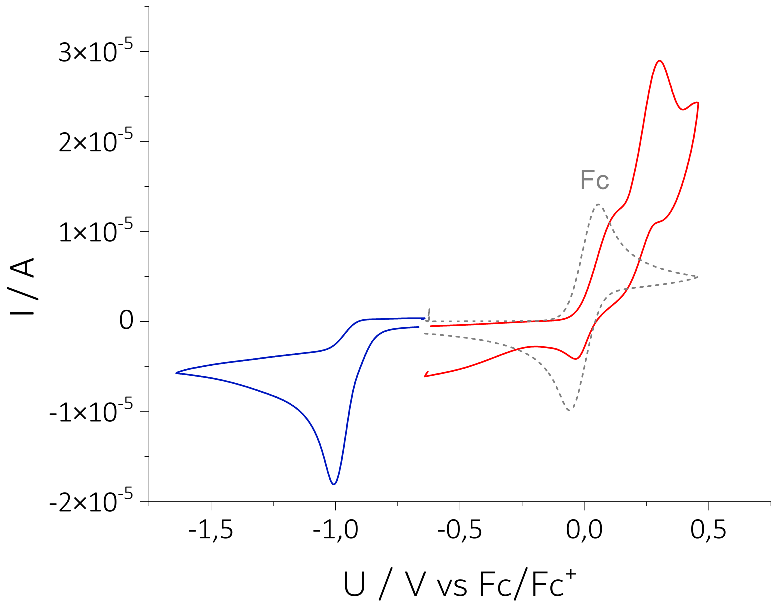

Figure S60. The cyclic voltammogram of **Cy7** (scan rate, 1000 mV s^{-1}, solvent: degassed DMF; supporting electrolyte: 0.1 mol L^{-1} $TBAClO_4$; a pseudo reference electrode: Ag wire; counter electrode and working electrode: Pt, and the potentials referenced to Fc/Fc^+).

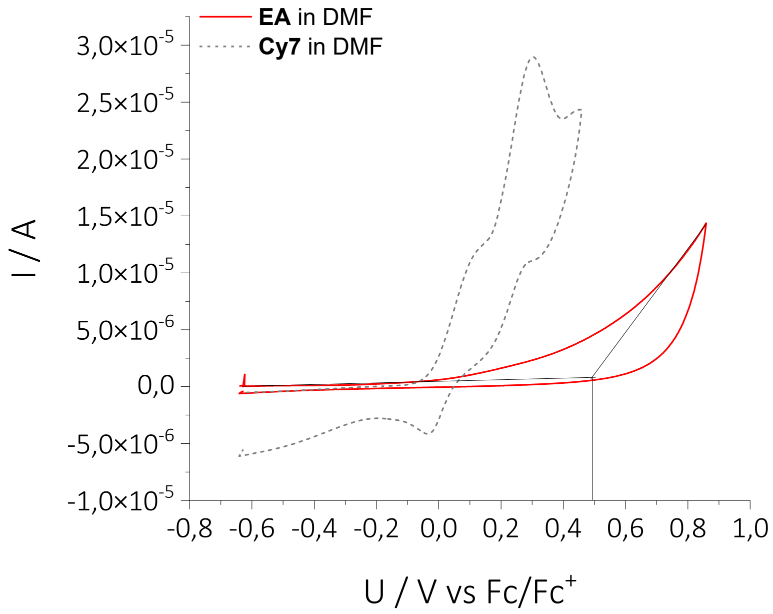

Figure S61. The cyclic voltammograms of **EA** and **Cy7** (scan rate, 1000 mV s^{-1}, solvent: degassed DMF; supporting electrolyte: 0.1 mol L^{-1} $TBAClO_4$; a pseudo reference electrode: Ag wire; counter electrode and working electrode: Pt, potentials referenced to Fc/Fc^+).

7.1. Redox potentials analysis

Cyclic voltammetry of **Cy7** (Figure S60) showed one-electron reduction ([Red]) to be fully irreversible with peak potential E_{pc} of -0.99 V vs. NHE. The one-electron oxidation potential ([Ox] $E_{1/2}$) of **Cy7** was observed at 0.85 V vs. NHE and found to be quasi-reversible with peak separation of 156 mV, indicative of an electrochemical ECE mechanism. Overall, our data are in good agreement with the values reported in DMF.[19] Regrettably, we were not able to obtain CV of **Cy7** in water due to its poor solubility, exacerbated in the presence of a supporting electrolyte. The measured potentials together with the excited state energy of S_1 (1.48 eV)[19] and T_1 (1.20 eV)[5] allowed us to calculate the excited state oxidation potentials ([Ox] $E^*_{1/2}$) of **Cy7** to be 0.46 and 0.18 V vs. NHE for S_1 and T_1 states, respectively. The irreversible nature of **Cy7** reduction prevents calculating the excited state reduction potential of **Cy7**, accordingly, we calculated the excited state reduction peak potential ([Red] E^*_{pc}) to be -0.53 and -0.25 V vs. NHE for S_1 and T_1 states, respectively (see Table S14). Furthermore, we estimated the oxidation peak potential ([Ox] E_{pa}) of **EA** under our conditions to be ≈1.60 V vs. NHE (Figure S61). This estimate is in line with the expected slightly lower oxidation potential of **EA**, as compared to other aliphatic primary amines (1.6–1.7 V vs. NHE).[20] With this in hand, we can see how the photoinduced electron transfer (PET) from **EA** to **Cy7** is unfavorable in both S_1 and T_1 states. On the other hand, PET from **Cy7** to molecular oxygen ([Red] $E_{1/2}$ = -0.33 V vs. NHE, in H_2O at pH 7)[21] is exergonic from both S_1 and T_1 states (-6.92 and -0.46 kcal mol^{-1}, respectively). ΔG_{PET} from excited states of **Cy7** to **NBA** and **MV^{2+}** was calculated to be –3.5 and –4.2 kcal mol^{-1} for the S_1 state, and +3 and +2.3 kcal mol^{-1} for the T_1 state, respectively. However, ΔG_{PET} for the **Cy7–MV^{2+}** couple changes to -10.4 and -3.9 kcal mol^{-1} for the S_1 and T_1 states of **Cy7**, respectively, when [Ox] $E_{1/2}$ of the **MV^{2+}** in DMF is used.

Table S14. The excited state energies *E* and redox potentials of compounds relevant to this work.

			Excited state *E* / eV		Excited state V vs. NHE			
					E^*_{red}		E^*_{ox}	
Compound (solvent)	1st Red V *vs.* NHE	1st Ox V *vs.* NHE	$E_{0,0}^{S_1}$	$E_{0,0}^{T_1}$	S_1	T_1	S_1	T_1
**Cy7** (DMF)	-0.99 [a]	0.85 [a]	1.48 [b]	1.20 [c]	0.49 [d]	0.21 [d]	-0.63 [d]	-0.35 [d]
**EA** (DMF)	- [n.d.]	1.60 [a]	-	-	-	-	-	-
Propylamine	-	1.7 [e]	-	-	-	-	-	-
Butylamine	-	1.7 [e]	-	-	-	-	-	-
**CQ** (H_2O, pH 9)	-0.55 [f]	-	-	2.02 [g]	-	1.47 [d]	-	-
O_2 (Au, H_2O)	-0.33 [h]	-	-	-	-	-	-	-
O_2 (Hg, DMSO)	-0.50 [h]	-	-	-	-	-	-	-
O_2 (Pt, Aprotic solvents)	-0.76 [i]	-	-	-	-	-	-	-
**NBA** (H_2O, pH 7)	-0.48 [j]	-	-	-	-	-	-	-
**MV^{2+}** (H_2O)	-0.45 [k]	-	-	-	-	-	-	-
**MV^{2+}** (DMF)	-0.18 [l]	-	-	-	-	-	-	-

[a] This work, [b] the value of 1.48 eV was obtained from the literature.[19] [c] the value of 1.20 eV was obtained by recalculating the literature value of 115.5 kJmol^{-1}.[5] [d] Calculated from the redox potentials and excited state energy with excited state redox potentials formula: ($E^*_{red}\left(\frac{cat^*}{cat^{\bullet-}}\right) = E_{red}\left(\frac{cat}{cat^{\bullet-}}\right) + E_{0,0}$) or ($E^*_{ox}\left(\frac{cat^{\bullet+}}{cat^*}\right) = E_{ox}\left(\frac{cat^{\bullet+}}{cat}\right) - E_{0,0}$).[22] [e] The value was obtained from the literature.[20] [f] The value of −0.55 V vs. NHE was obtained by recalculating the literature value of -0.80 V vs. SCE (H_2O, pH 9)[23] [g] The value of 2.02 eV was obtained from the literature.[24] [h] The value was obtained from the literature.[21] [i] The value of −0.76 V vs. NHE was obtained by recalculating the literature value of -1.00 V vs. SCE.[25] [j] The value was obtained from the literature.[26] [k] The value was obtained from the literature.[27-28] [l] The value of -0.18 V vs. NHE was obtained by recalculating the literature value of -0.424 V vs. SCE from.[28] n.d. not determined.

8. Overview of the experimental results

Table S15. Overview of experiments, conclusions drawn and reference to the experimental data

Target	Additive [a] (comment)	Outcome			Description and conclusion	Experimental data
		**Cy5** / %	$\Phi_{\textbf{Cy7}\ dec}$	$\Phi_{\textbf{Cy5}\ form}$		
$^3O_2$	Aerated (no **NBA**)	16%	- n.d.	- n.d.	O_2 is crucial for the formation of **Cy5**.	
	Aerated (with **NBA**)	31%	1,32 × 10^{-3}	4.19 × 10^{-4}		Table S6
	Degassing (N_2 saturated, with **NBA**)	- n.d.	3.93 × 10^{-5}	7.55 × 10^{-6}		Figure S36
	Degassed (freeze – pump -thaw)	<0.1%	- n.d.	- n.d.		
$^1O_2$	NaN_3 (Quencher)	15%	- n.d.	- n.d.	Insignificant decrease in the **Cy5** yield. Moreover, thermally	Figure S4
	DABCO (Quencher)	13%	- n.d.	- n.d.	generated $^1O_2$ does not lead to any significant amount of **Cy5**.	
	Furfuryl alcohol ($^1O_2$ Trap)	11%	- n.d.	- n.d.	Overall: $^1O_2$ is not responsible for production of **Cy5**, however, it is	
	**EN** (thermally generated $^1O_2$)	<0.5%	- n.d.	- n.d.	included in the decomposition of **Cy7**	Figure S97
$OH^•$	**DMPO**	15%	- n.d.	- n.d.	Insignificant decrease in **Cy5** yield. $OH^•$ is not responsible for	Figure S5
	t-Butyl alcohol	16%	- n.d.	- n.d.	production of **Cy5**	
Radical scavengers	*N*-Acetylcisteine, Glutathione, and Ascorbic acid (also e^- donor)	<0.1%	- n.d.	- n.d.	Formation of **Cy5** completely halted, i.e., radical species involved in the formation of **Cy5**	Figure S6<br>Figures S90 to S95
$O_2^{•-}$	**SOD** ($O_2^{•-}$ scavanger)	16%	- n.d.	- n.d.	$O_2^{•-}$ is not responsible for the production of **Cy5**.	Figure S5
	**NBT** ($O_2^{•-}$ trap and e^- acceptor)	30%	- n.d.	- n.d.	Increase in the **Cy5** formation, however, **NBT** acts as an electron acceptor	Figure S11
**Cy7** (T_1)	KI	24%	- n.d.	- n.d.	Sensitization of **Cy7** to its T_1 state leads to increased **Cy5** yield.	Figure S7
Φ_{ISC} =	Anthracene	19%	- n.d.	- n.d.	Quenching of $^1O_2$ decreases the **Cy7** decomposition.	Figure S8
8.9 × 10^{-3}	**2**	20%	- n.d.	- n.d.	Overall: increasing the population of **Cy7** (T_1) state increases **Cy5**	
	**2** + NaN_3	17%	- n.d.	- n.d.	yield.	
	**COT-COOH** (in **EA** buffer)	16%	- n.d.	- n.d.	Physical quenching of the **Cy7** (T_1) state does not decrease **Cy5** yield;	Figure S9
	**COT-COOH** + NaN_3 (in **EA** buffer)	16%	- n.d.	- n.d.	the extent of **Cy7** (T_1) involvement in phototruncation is negligible.	
	**COT-COOH** (in MeOH)	- n.d	- n.d.	- n.d.	Experiments with **COT-COOH** in MeOH demonstrate that NaN_3	Figure S10
	**COT-COOH** + NaN_3 (in MeOH)	- n.d	- n.d.	- n.d.	indeed quenches T_1 of **Cy7**.	
E_{ox} (T_1) =	**NBA** ($E_{1/2}$ (red) = -0.48 V vs. NHE) [b]	28%	6.56 × 10^{-4}	2.07 × 10^{-4}	Chemical quenching of the **Cy7** (T_1) state significantly increases **Cy5**	Figures S11 and S26
-0.35 V	**NBA** + NaN_3	33%	- n.d.	- n.d.	yield.	Figure S13
vs. NHE [b]	$\textbf{MV}^{2+}$ ($E_{1/2}$ (red) = -0.45 V vs. NHE) [b]	28%	1.04 × 10^{-3}	3.19 × 10^{-4}	However, all of the used quenchers operate via electron transfer,	Tables S3 to S5,
	**NBT**	30%	- n.d.	- n.d.	resulting in the formation of $\textbf{Cy7}^{•+}$	Figures S34 and S35
$\textbf{Cy7}^{•+}$	**CQ** (in ACN/H_2O)	<0.1%	- n.d.	- n.d.	**CQ** effectively photooxidizes **Cy7**.	Figure S24
E_{ox} (**Cy7**) =	**CQ** ($E_{1/2}$ (T_1) = 1.49 V vs. NHE) [b]	20%	- n.d.	- n.d.	Oxidation of **Cy7** leads to the **Cy5** formation	Figure S12
0.85 V	**CQ** + NaN_3	33%	- n.d.	- n.d.	Quenching of $^1O_2$ (sensitized by ^{3}**CQ***) increases the **Cy5** formation	
vs. NHE [b]	**CQ** (no O_2)	<0.3%	- n.d.	- n.d.	O_2 is involved in a step after **Cy7** oxidation	

[a] Specific conditions are described together with the experimental data (see the correspoding place), [b] For detailed analysis of the redox potentials and feasibility of electron transfer processes, see Table S14, n.d. = not determined

9. A flow photoreactor

Flow photoreactor design and construction

The 3D model of the photoreactor was designed with an AutoCAD 2024 software (academic license); the file is sliced using an Anycubic Photon Workshop 3.6.2 software (free version) and 3D printed using Anycubic Tough Resin 2.0, on an Anycubic Photon Mono M5s Pro DLP printer equipped with 405-nm high power light emitting diode (LED) as an irradiation source. The Photoreactor (Figure 62) consists of several parts; the main body (Figure S62a) serves to hold 4 LED strips and as a reflector for focusing the scattered light on the PFA tubing wind around central cylinder (1.4 mL internal volume. Two clamps (Figure S62a) are used for securing the reactor in place, and the light shielding cylinder (Figure S62b) is used for limiting the light exposure of the PFA tubing, allowing for changing the residence time without the need to change the flow rate. Each of the LED strips contains 12, 20 mW LEDs connected in series, driven by the constant current source based on TL431 supplied by 29 V/20 mA using a Rigol DP821 power supply (Figure 63).

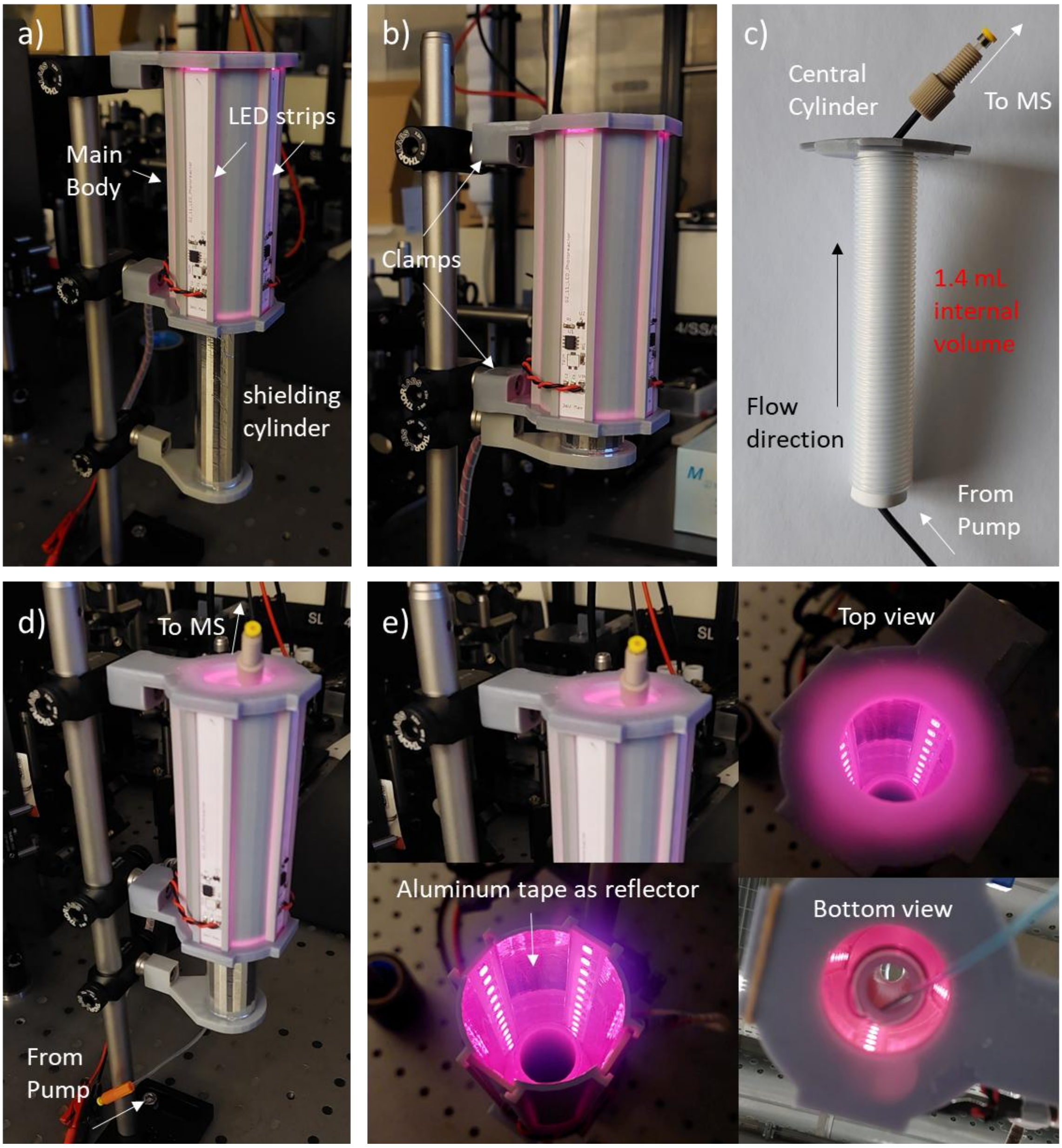

Figure S62. Highlighted various parts of a flow photoreactor; a) a shielding cylinder completely out (full 1.4 mL of the reaction mixture is irradiated), b) a shielding cylinder fully inserted (no reaction mixture irradiated), c) a view at the central cylinder, composed of reflective Teflon tape with a PFA tube wound around it, having 1.4 mL internal volume, d) an assembled photoreactor, e) different views of the LED strips.

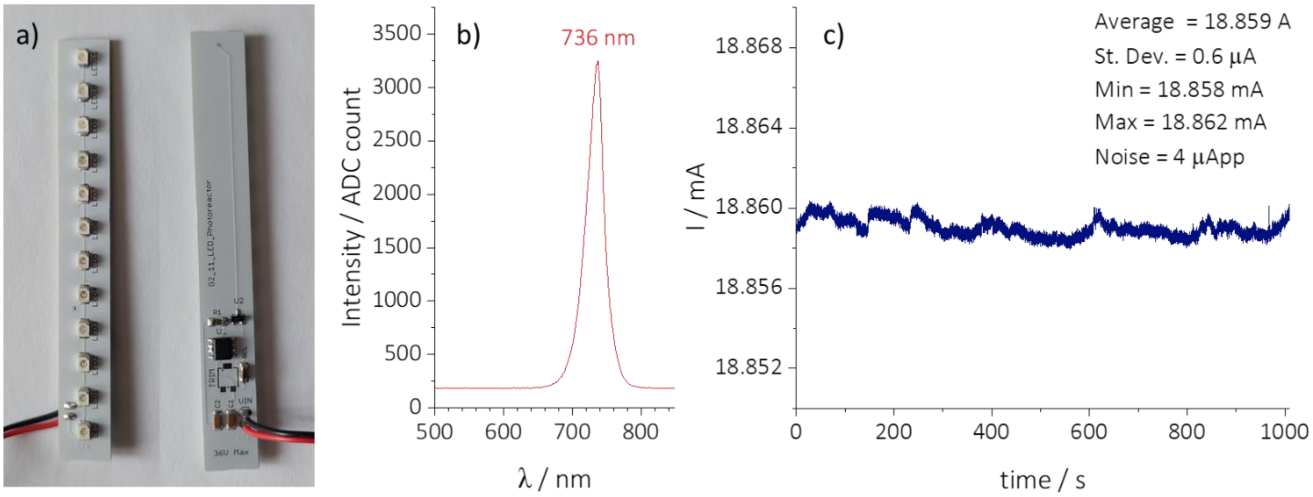

Figure S63. The characterization of LED strips; a) the printed circuit boards with LEDs on the front (left) and the driving circuit on the back (right), b) an emission spectrum of LEDs, c) long-term stability and noise measurements of the LED driver. The current measurement was performed by a calibrated Keithley DMM6500, 6.5-digit multimeter, with 0.05 number of the power line cycles sampling time (NPLC = 0.024 s for 60 Hz power grid). All 4 panels are driven by identical low noise constant current drivers, providing 19 ± 0.2 mA of current through each LED (using 1%, 50 ppm resistors). The 4 µApp current noise results in ± 0.022 % photon flux noise.

Coupling of a flow photoreactor to the mass spectrometer

A flow photoreactor was coupled to the mass spectrometers: a Bruker TIMS-TOF Classic instrument with an ESI source or an Infrared Spectroscopy of Reaction Intermediates (ISORI) instrument, equipped with an electrospray ionization (ESI source[29]) by quartz capillary and appropriate fluidic connectors (Figures S64–S65). The inline UV-Vis cell was placed at the exit port of the flow reactor allowing for simultaneous monitoring of the reaction by UV-Vis (Avantes, AvaSpec-ULS2048x64-EVO). The reaction mixture was pumped through the photoreactor using a syringe pump (Labm8 M8003 syringe pump) with a flow rate of 140 µL/min, providing a 10 min residence time. The content of the syringe was protected from the ambient light by aluminum foil (Figure S66c).

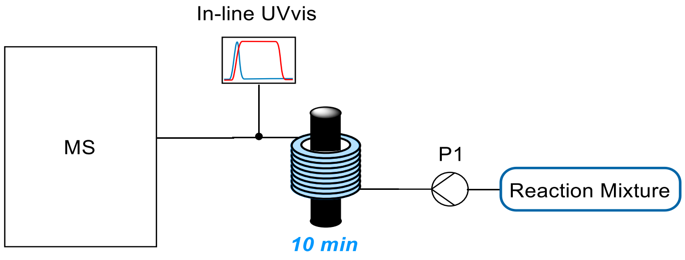

Figure S64. A flow photoreactor coupled to MS with in-line UV-Vis measurement, a schematics of the setup

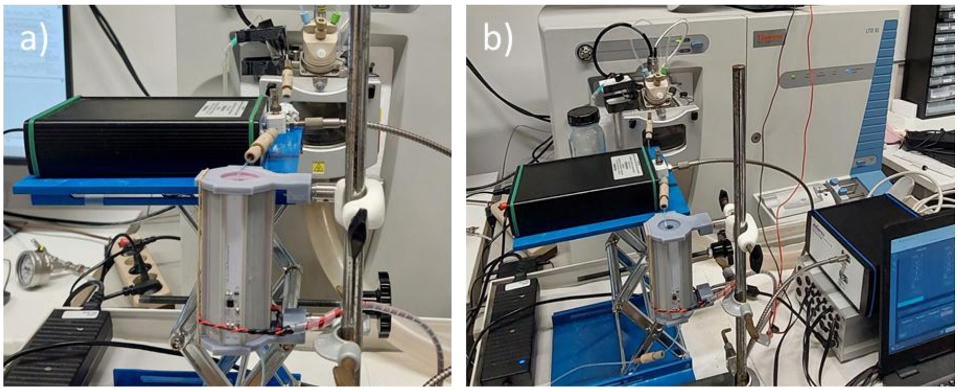

Figure S65. A flow photoreactor coupled to MS; a) and b) different views of the photoreactor coupled to the low-resolution MS (Thermo Scientific LTQ XL) with in-line UV-Vis measurement (Avantes).

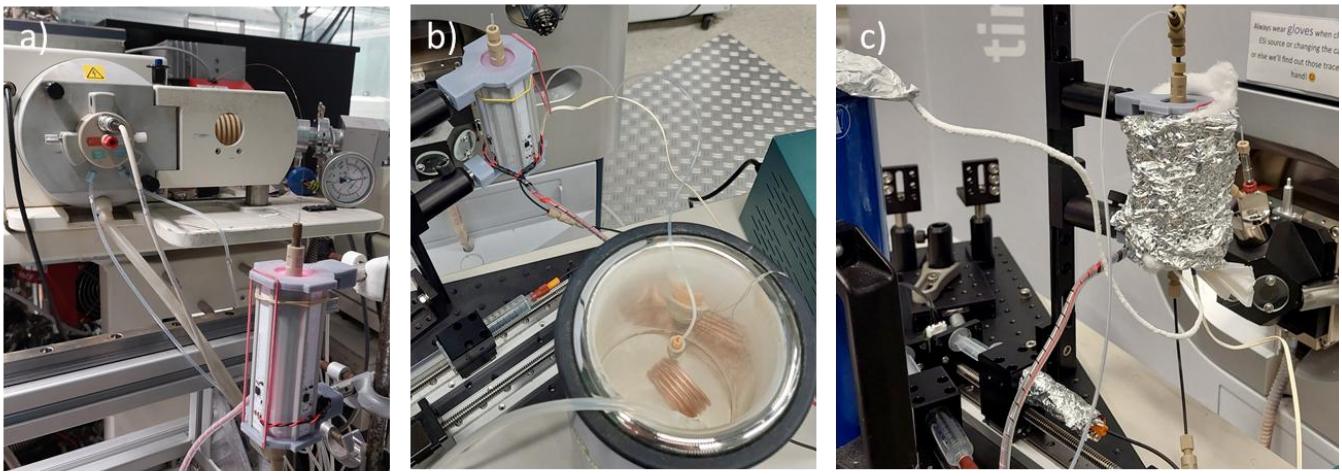

Figure S66. A flow photoreactor coupled to; a) an Infrared Spectroscopy of Reaction Intermediates (ISORI) instrument equipped with an electrospray ionization (ESI) source,[29] b) and c) the photoreactor coupled to a Bruker TIMS-TOF Classic instrument with an ESI source, modified for the ability to cool the reaction mixture by stream of cold nitrogen gas.

10. High-resolution mass spectrometry - trapped ion mobility spectrometry

The HRMS data below were obtained with a Bruker TIMS-TOF Classic instrument with an ESI source spectrometer with a coupled flow photoreactor (as described above). The conditions for experiments, unless stated otherwise, were 50 µM cyanine in **EA** buffer (500 mM, pH 8.7, 2% MeOH as a cosolvent), 140 µL min^{-1} flow rate (10 min residence time), irradiation with LEDs with emission maxima centered at 736 nm (Figure S63b). In experiments where it was necessary to regulate the temperature, it was achieved by the stream of warm or cold air and measured using a temperature probe placed close to the PFA tubing of the reactor (Figures S66b and S66c).

The HRMS experiments, including HRMS spectra, the collision-induced dissociation (CID) spectra of selected ions, the time traces of selected ions presented as ion count normalized to the total ion current (TIC), and mobilograms of selected ions are divided into following sections:

- HRMS of **Cy7** and derivatives in **EA** buffer
- Crossover experiment
- The kinetic isotope effect
- HRMS of **Cy7** and derivatives with addition of N-acetylcysteine
- HRMS of **Cy7** and derivatives irradiated in PBS buffer
- HRMS of **Cy7** and derivatives reaction with endoperoxide
- Crossover experiment with **Cy5**
- Mobilograms of selected ions
- HRMS detection of **10**

10.1. HRMS of Cy7 and derivatives in EA buffer

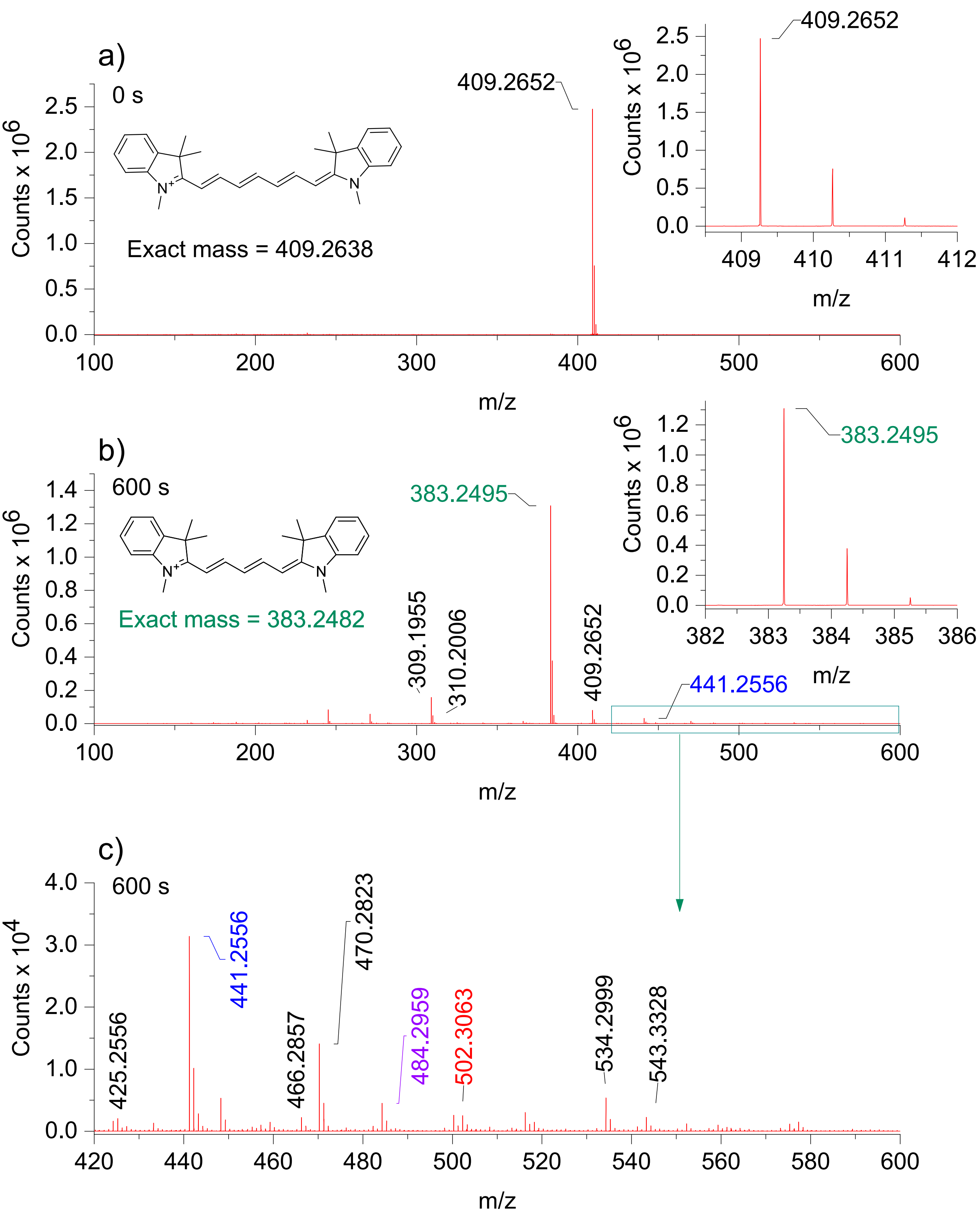

Figure S67. The HRMS spectra; a) before irradiation (pure **Cy7**; 409.26 *m/z*), b) after 600 s of irradiation (showing the conversion to **Cy5**, 383.24 *m/z*), and c) zoomed view in the 420–600 *m/z* region of the HRMS spectra presented in b). Reaction conditions: **Cy7** (50 µM), **EA** buffer (500 mM, pH = 8.7), irradiated in a flow photoreactor (Figure S62).

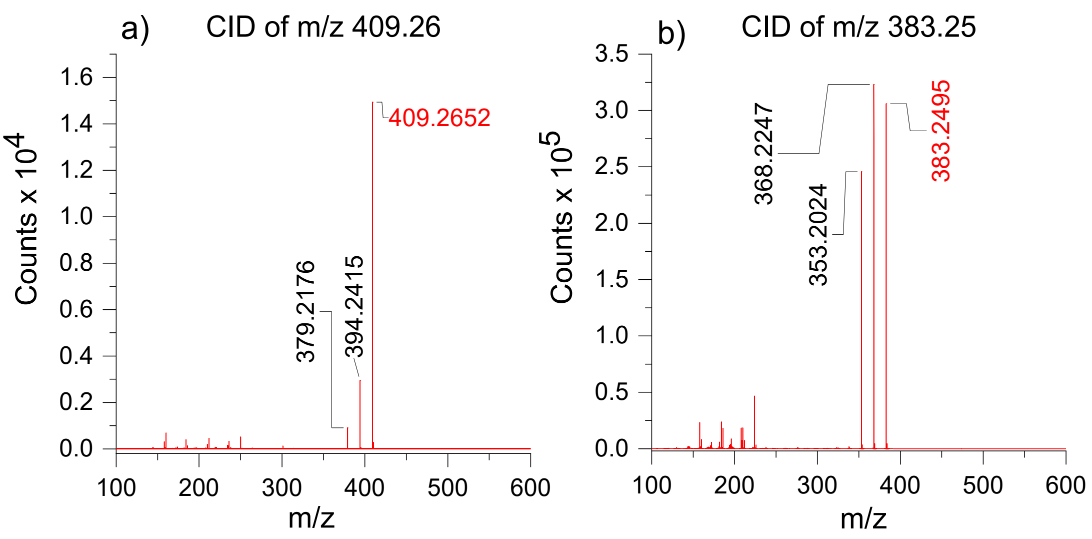

Figure S68. The CID spectra of a) 409.26 *m/z* (**Cy7**) and b) 383.24 *m/z* (**Cy5**). Both cyanines loose 15 *m/z* twice (CH_3 group).

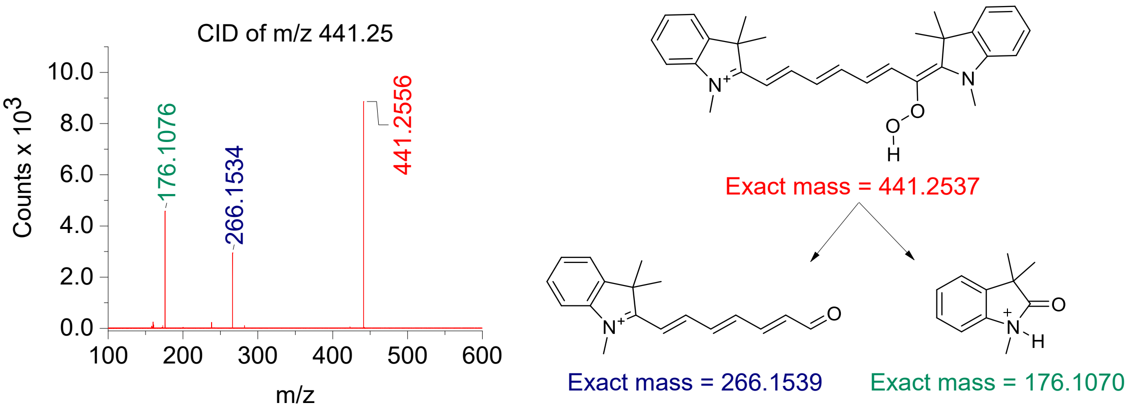

Figure S69. The CID spectra of a 441.25 *m/z* side-product (**3**).

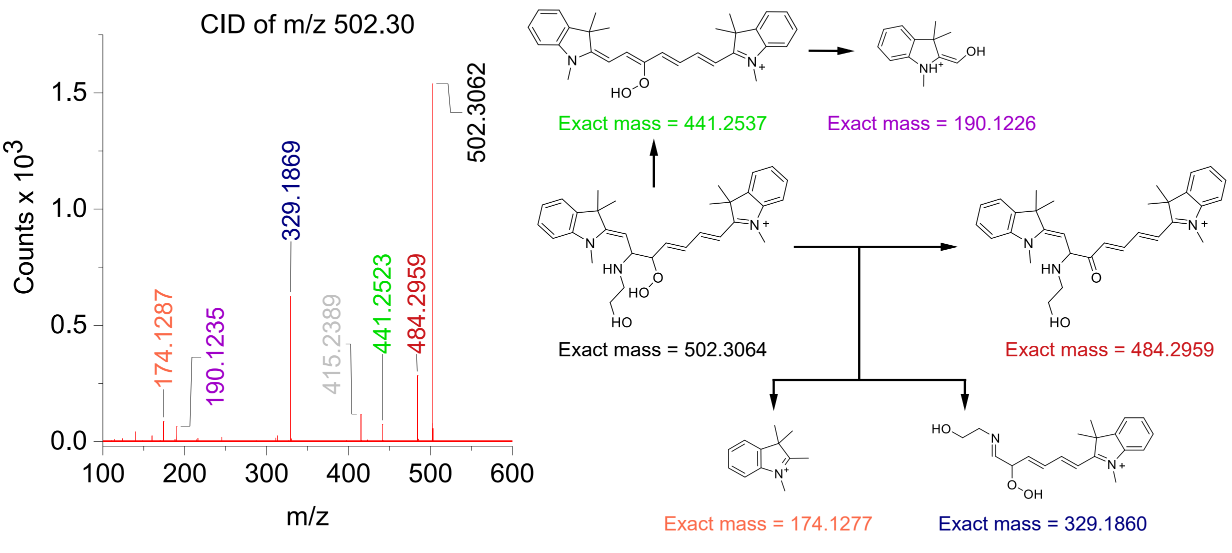

Figure S70. The CID spectrum of a 502.30 *m/z* intermediate (**4**).

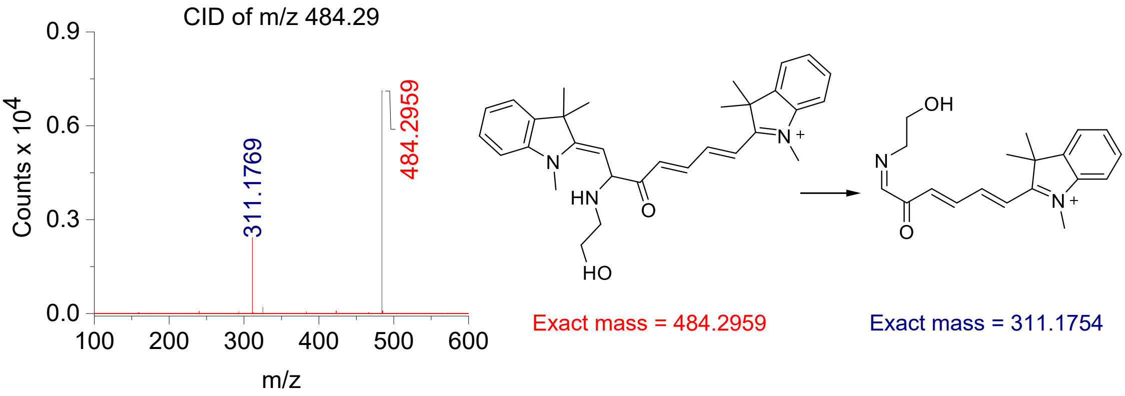

Figure S71. The CID spectra of 484.29 *m/z* intermediate (**5**).

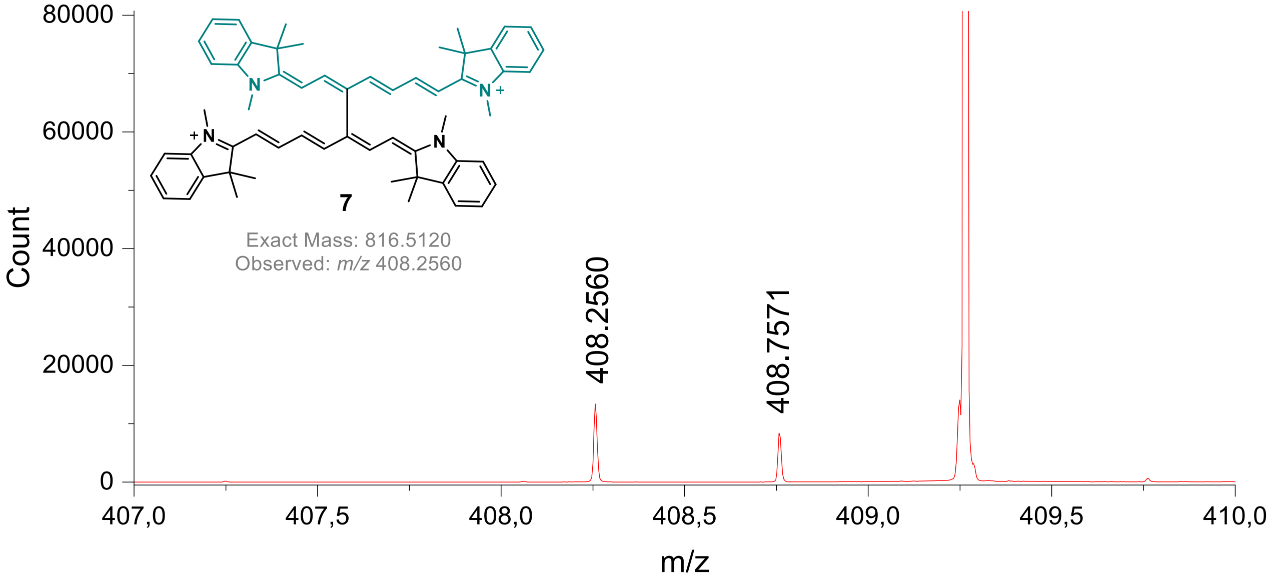

Figure S72. The HRMS spectrum of 408.25 *m/z* assigned to the **Cy7** dimer.

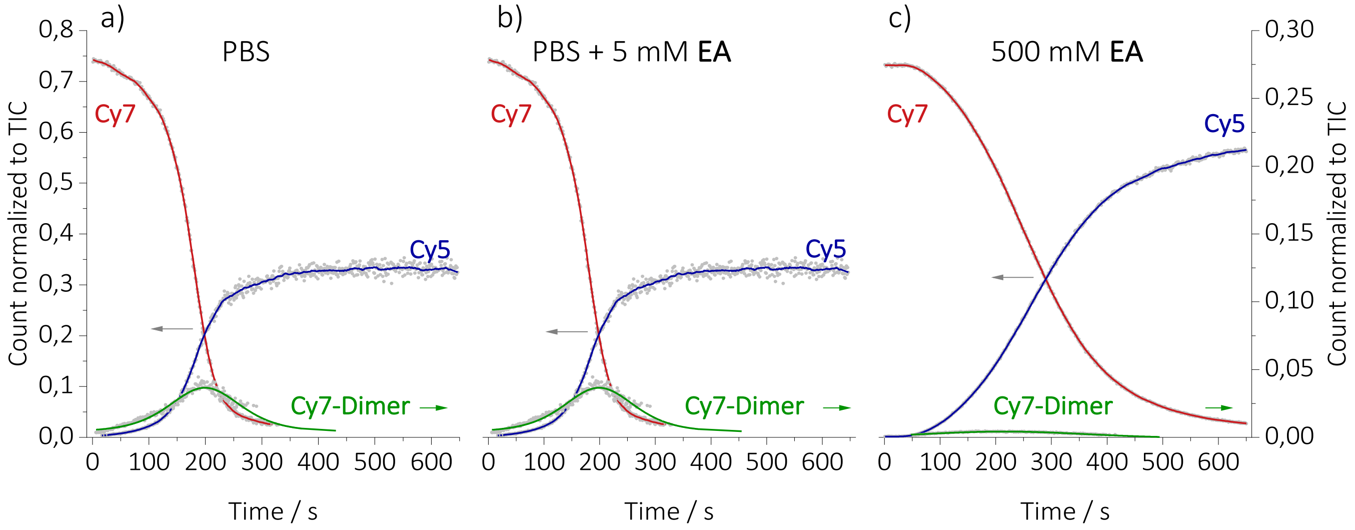

Figure S73. The time traces showing temporal evolution of **Cy7** (409.26 *m/z*), **Cy5** (383.24 *m/z*) and **Cy7**-dimer (408.25 *m/z*) when performing the reaction in a) PBS buffer (10 mM, pH = 7.4), b) PBS buffer with 5 mM **EA** (PBS; 10 mM, pH = 7.4), and c) 500 mM **EA** buffer (pH = 8.7).

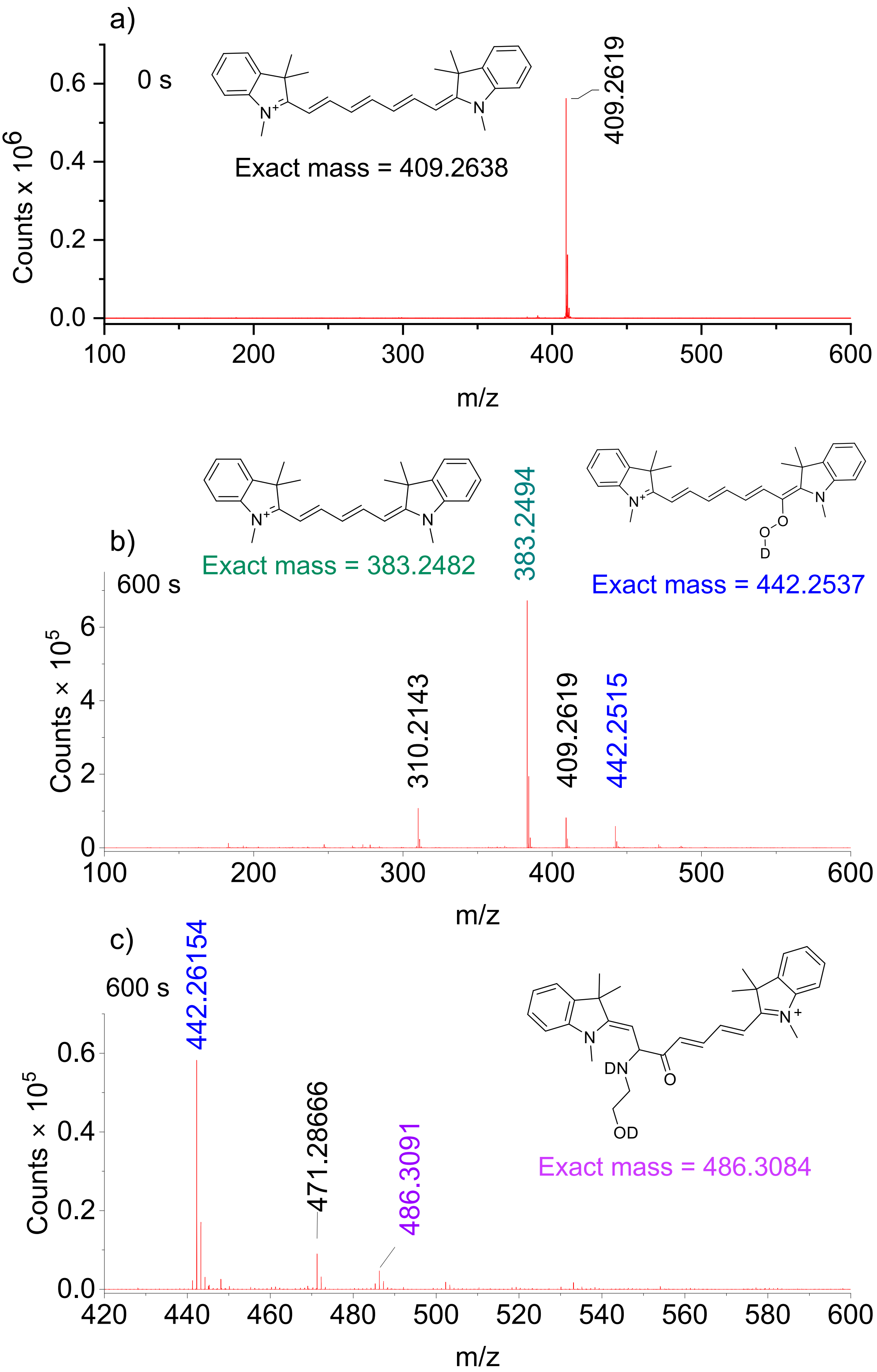

Figure S74. The HRMS spectra; a) before irradiation (pure **Cy7**; 409.26 *m/z*), b) after 600 s of irradiation (showing the conversion to **Cy5**, 383.24 *m/z*), and c) zoomed view in the 420 – 600 *m/z* region of the HRMS spectra presented in b). Reaction conditions: **Cy7** (50 µM), **EA** buffer (500 mM, pH = 8.7) prepared in D_2O and irradiated in a flow photoreactor (Figure S62).

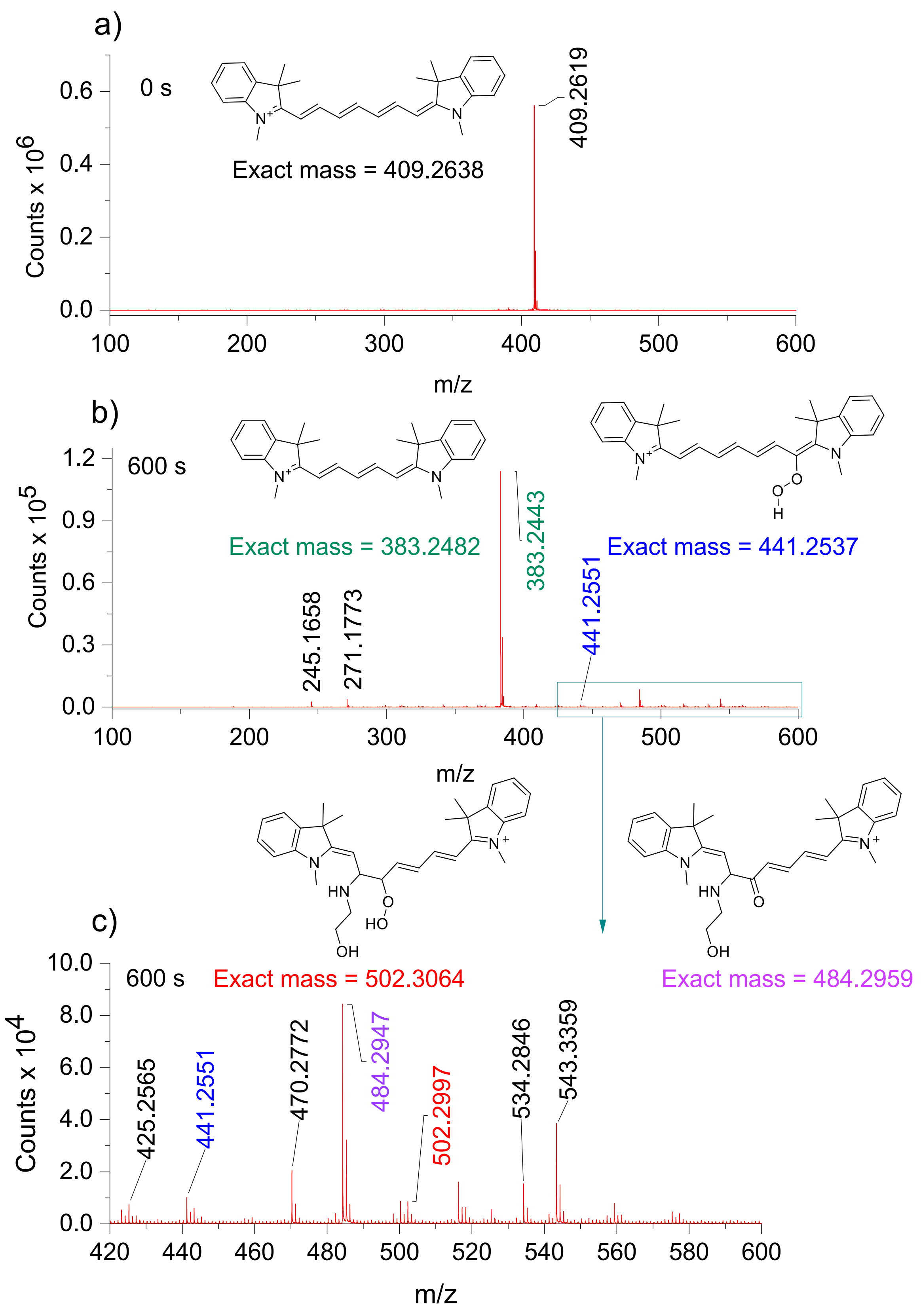

Figure S75. The HRMS spectra; a) before irradiation (pure **Cy7**; 409.26 *m/z*), b) after 600 s of irradiation (showing the conversion to **Cy5**, 383.24 *m/z*), and c) zoomed view in the 420 – 600 *m/z* region of the HRMS spectra presented in b). Reaction conditions: **Cy7** (50 µM), **EA** buffer (500 mM, pH = 8.7) prepared in $D_2^{18}O$ (75% ^{18}O, ≈ 75% D) and irradiated in a flow photoreactor (Figure S62).

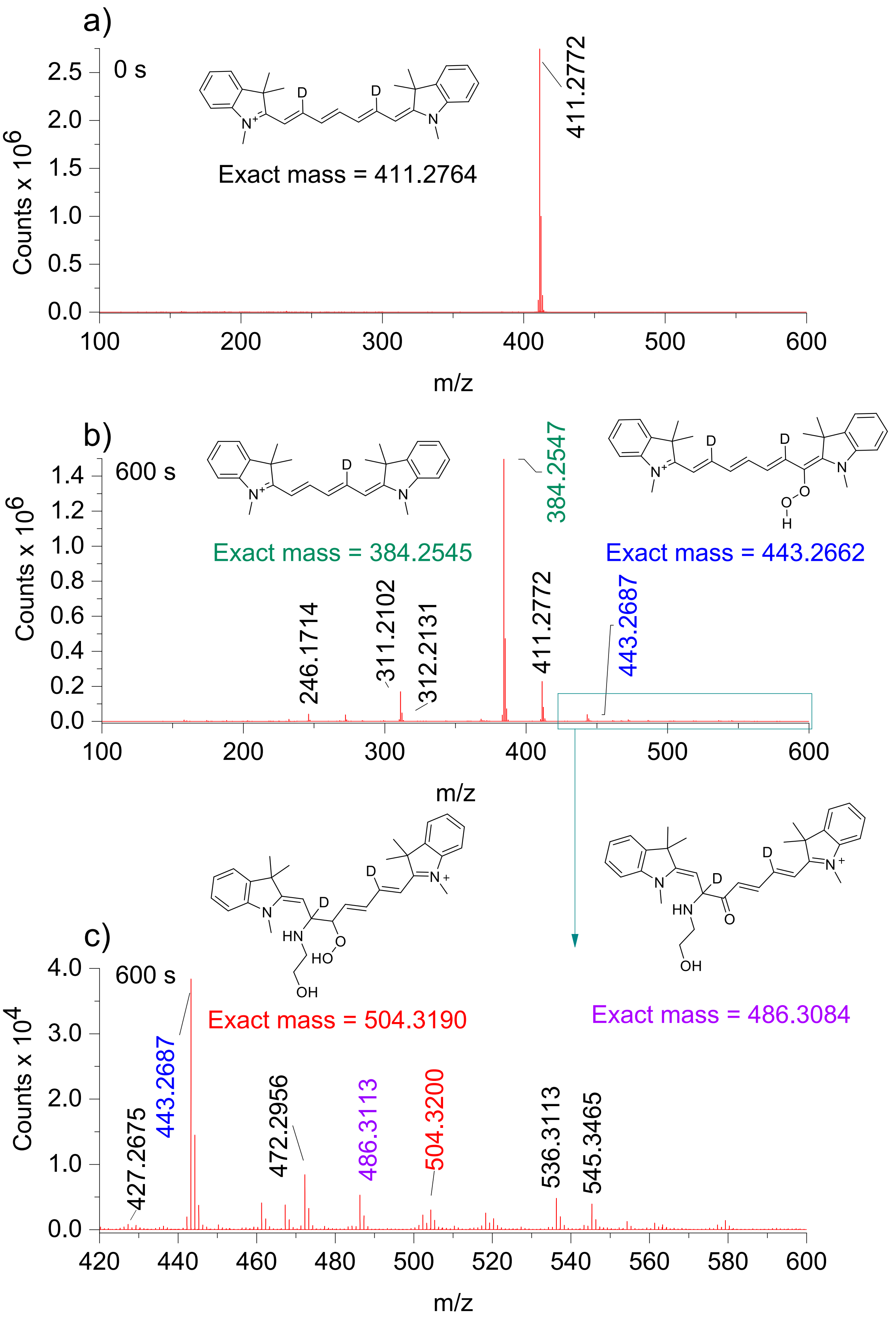

Figure S76. The HRMS spectra; a) before irradiation (**Cy7**-2´,6´-d_2; 411.27 *m/z*), b) after 600 s of irradiation (showing the conversion to **Cy5**-2´-*d*, 383.24 *m/z*), and c) zoomed view in the 420 – 600 *m/z* region of the HRMS spectra presented in b). Reaction conditions: **Cy7**-2´,6´-d_2 (50 µM), **EA** buffer (500 mM, pH = 8.7), irradiated in a flow photoreactor (Figure S62).

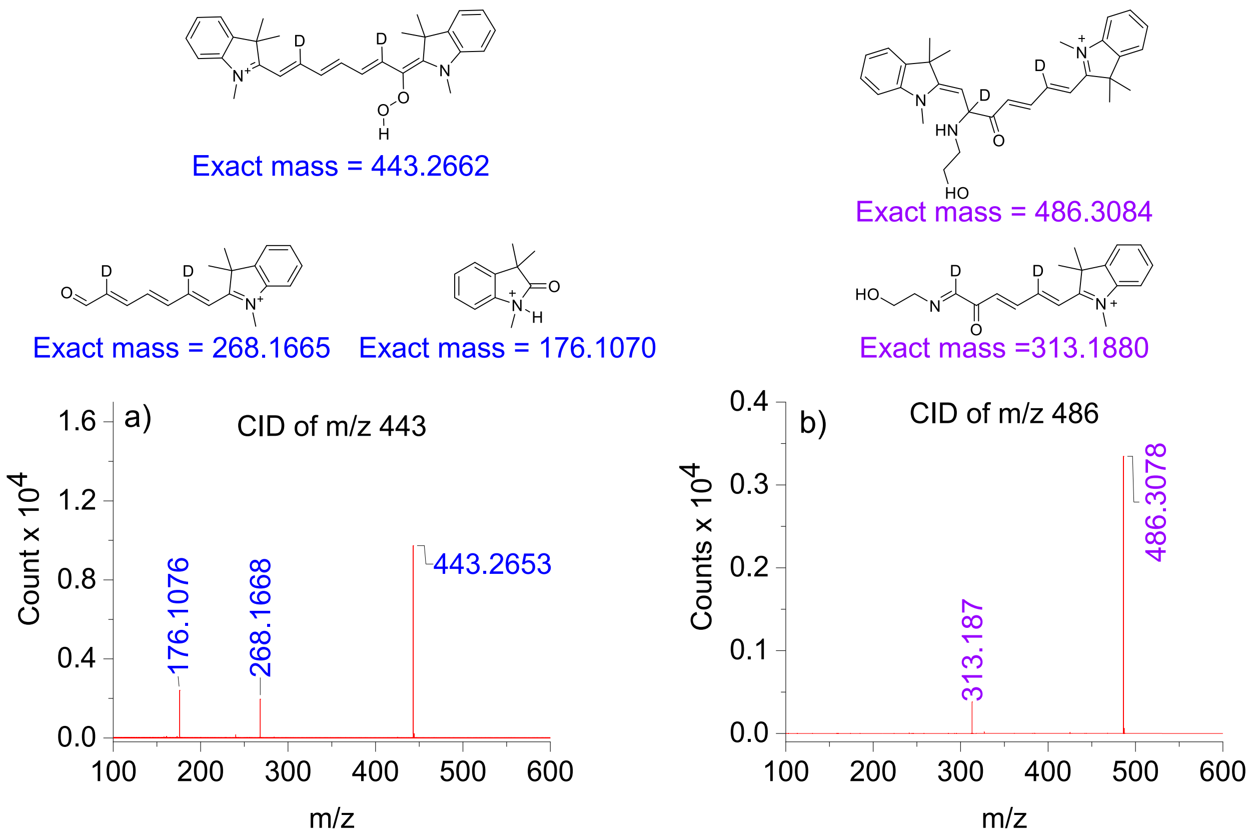

Figure S77. The CID spectra of a) 443.26 *m/z*, and b) 484.30 *m/z* ions, from the HRMS experiment in Figure S76

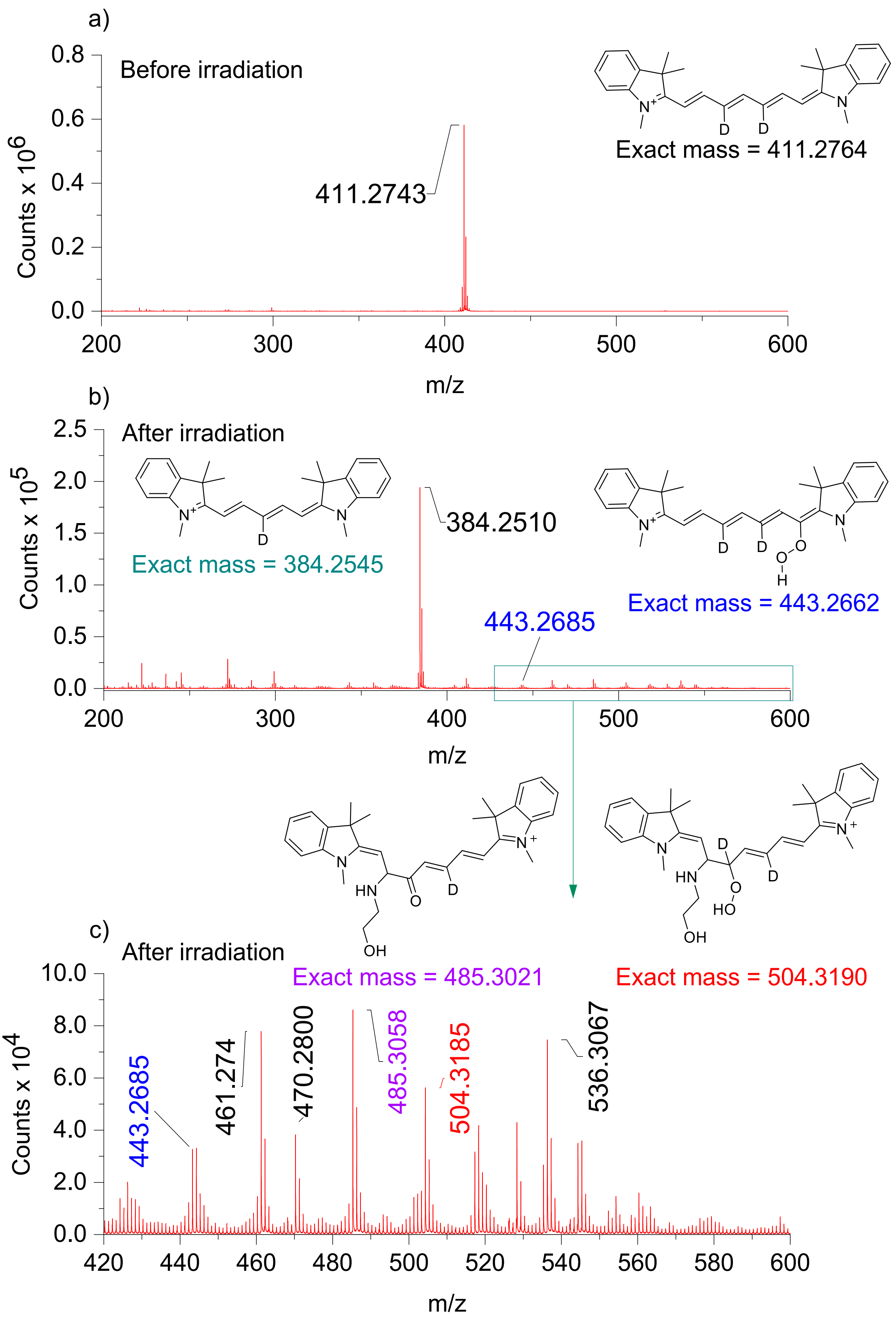

Figure S78. The HRMS spectra; a) before irradiation (**Cy7-3´,5´-*d*$_2$**; 411.27 *m/z*), b) after 600 s of irradiation (showing the conversion to **Cy5-2´-*d*,** 383.24 *m/z*), and c) zoomed view in the 420 – 600 *m/z* region of the HRMS spectra presented in b). Reaction conditions: **Cy7-2´,6´-*d*$_2$** (50 µM), **EA** buffer (500 mM, pH = 8.7), irradiated in a flow photoreactor (Figure S62).

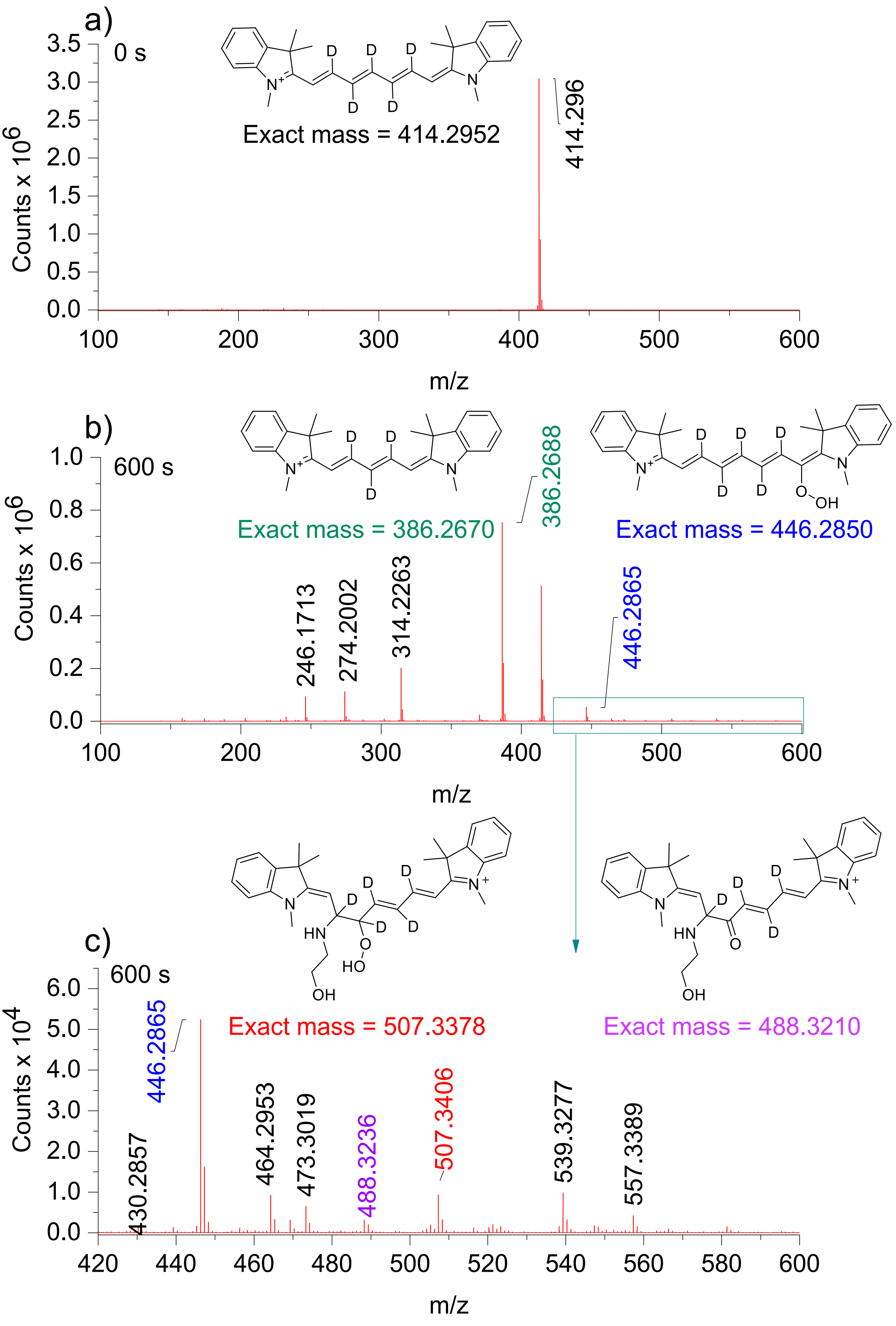

Figure S79. The HRMS spectra; a) before irradiation (**Cy7-2´,3´,4´,5´,6´-*d*$_5$**; 414.29 *m/z*), b) after 600 s of irradiation (showing the conversion to **Cy5-2´,3´,4´-*d*$_3$**, 386.26 *m/z*), and c) zoomed view in the 420 – 600 *m/z* region of the HRMS spectra presented in b). Reaction conditions: **Cy7** (50 μM), **EA** buffer (500 mM, pH = 8.7), irradiated in a flow photoreactor (Figure S62).

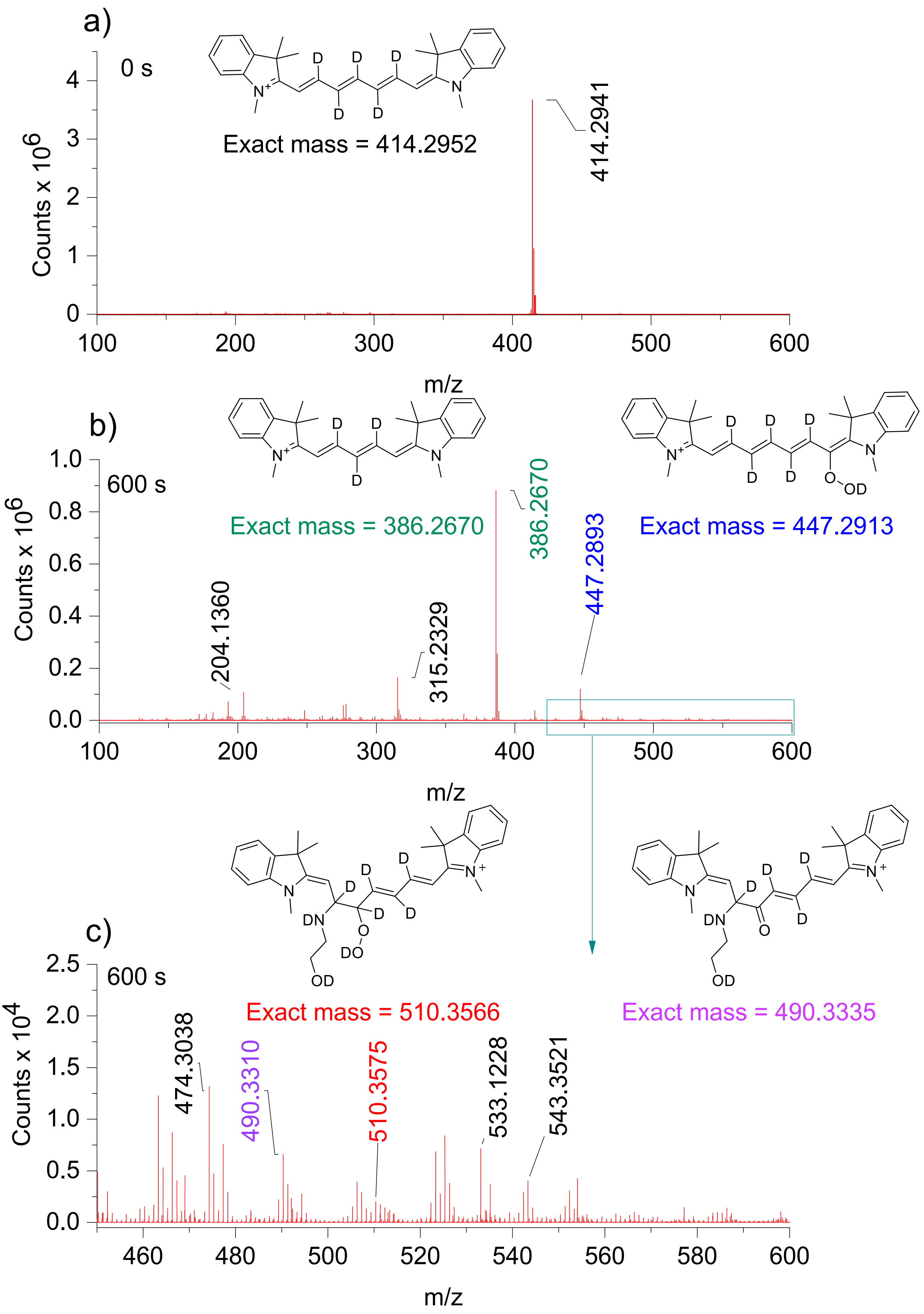

Figure S80. The HRMS spectra; a) before irradiation (**Cy7-2´,3´,4´,5´,6´,*d*$_5$**; 414.29 *m/z*), b) after 600 s of irradiation (showing the conversion to **Cy5-2´,3´,4´-*d*$_3$**, 386.26 *m/z*), and c) zoomed view in the 420 – 600 *m/z* region of the HRMS spectra presented in b). Reaction conditions: **Cy7** (50 µM), **EA** buffer (500 mM, pH = 8.7) prepared in D_2O and irradiated in a flow photoreactor (Figure S62).

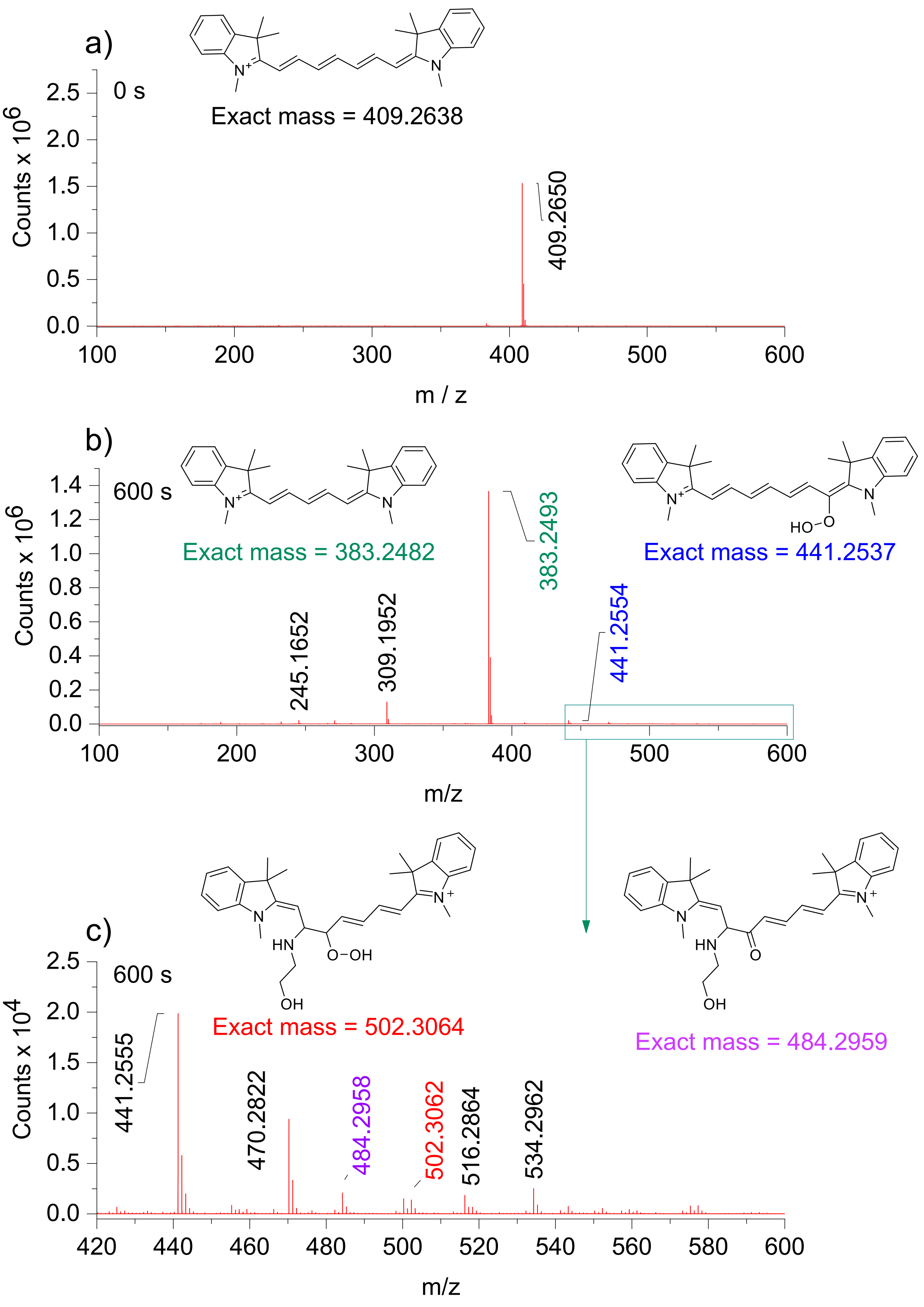

Figure S81. The HRMS spectra; a) before irradiation (**Cy7**; 409.26 *m/z*), b) after 600 s of irradiation (showing the conversion to **Cy5**; 383.24 *m/z*), and c) zoomed view in the 420 – 600 *m/z* region of the HRMS spectra presented in b). Reaction conditions: **Cy7** (50 µM), **EA** buffer (500 mM, pH = 8.7) with addition of **NBA** (2 mM), irradiated in a flow photoreactor (Figure S62).

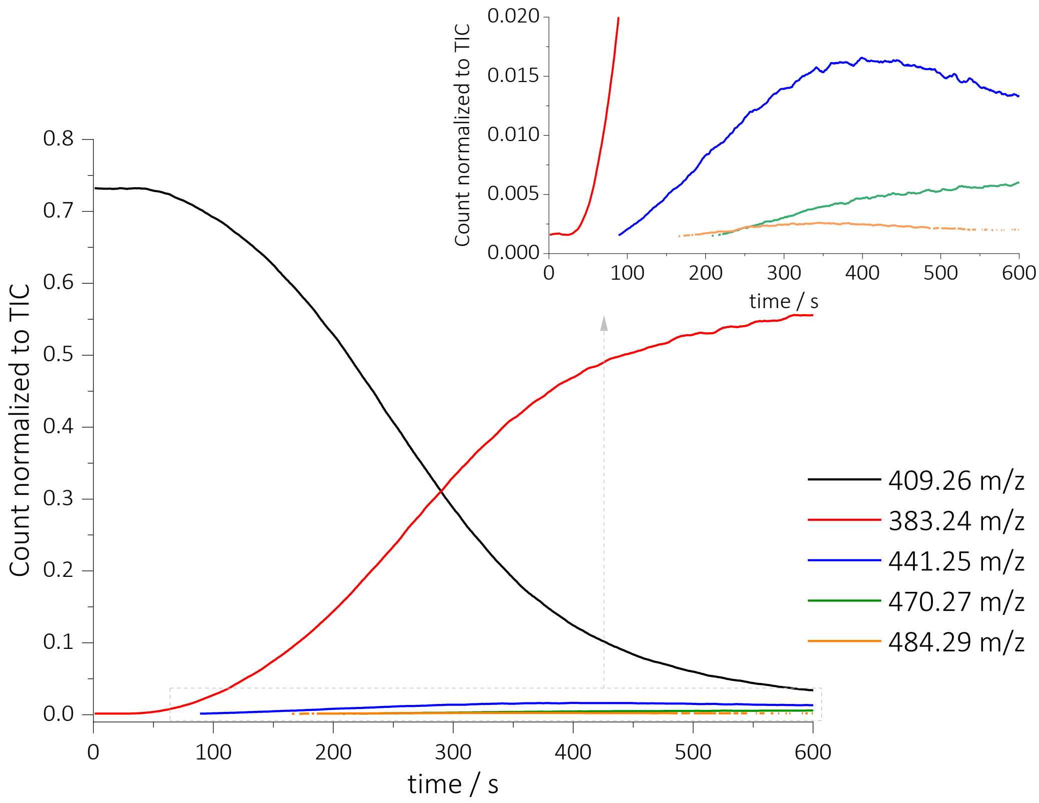

Figure S82. The time traces showing temporal evolution of 409.26 *m/z* (**Cy7**), 383.24 *m/z* (**Cy5**), 441.25 *m/z* (**Cy7-OOH**), 470.27 *m/z* and 484.29 *m/z* ions, from the HRMS experiment in Figure S67 (Irradiation of **Cy7** in **EA** buffer; 500 mM **EA**, pH = 8.7 prepared in H_2O).

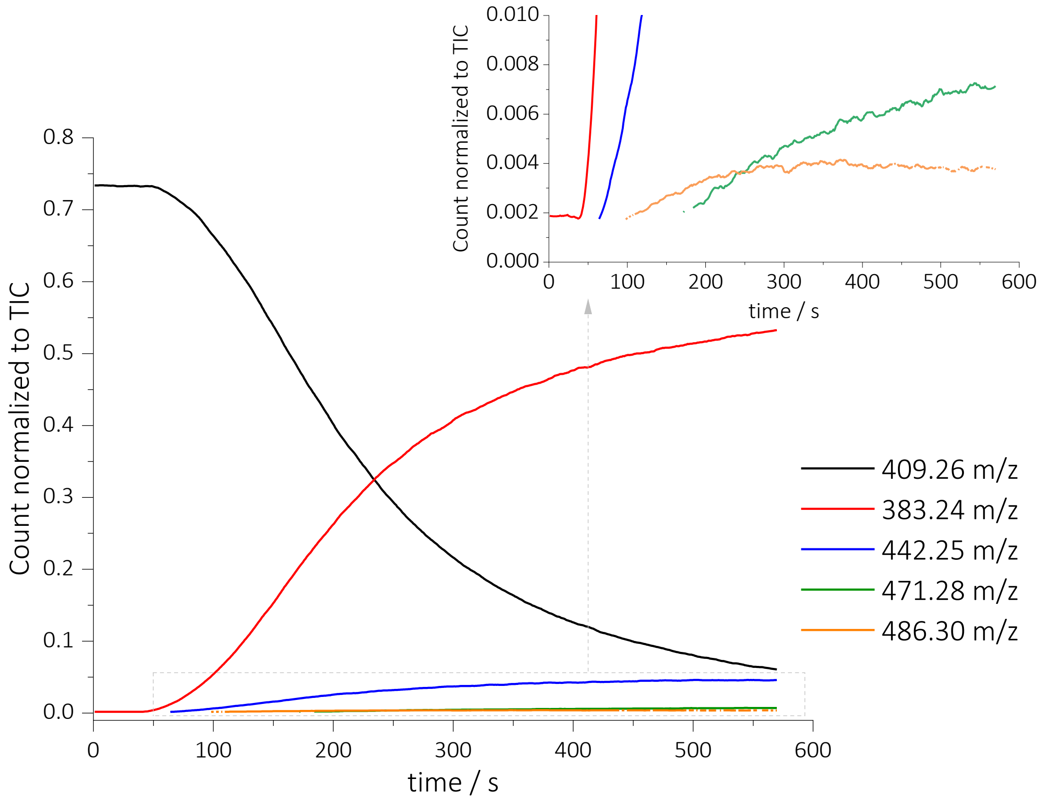

Figure S83. The time traces showing temporal evolution of 409.26 *m/z* (**Cy7**), 383.24 *m/z* (**Cy5**), 442.25 *m/z* (**Cy7-OOH**), 471.28 *m/z* and 486.30 *m/z* ions, from the HRMS experiment in Figure S74 (Irradiation of **Cy7** in **EA** buffer; 500 mM **EA**, pH = 8.7 prepared in D_2O).

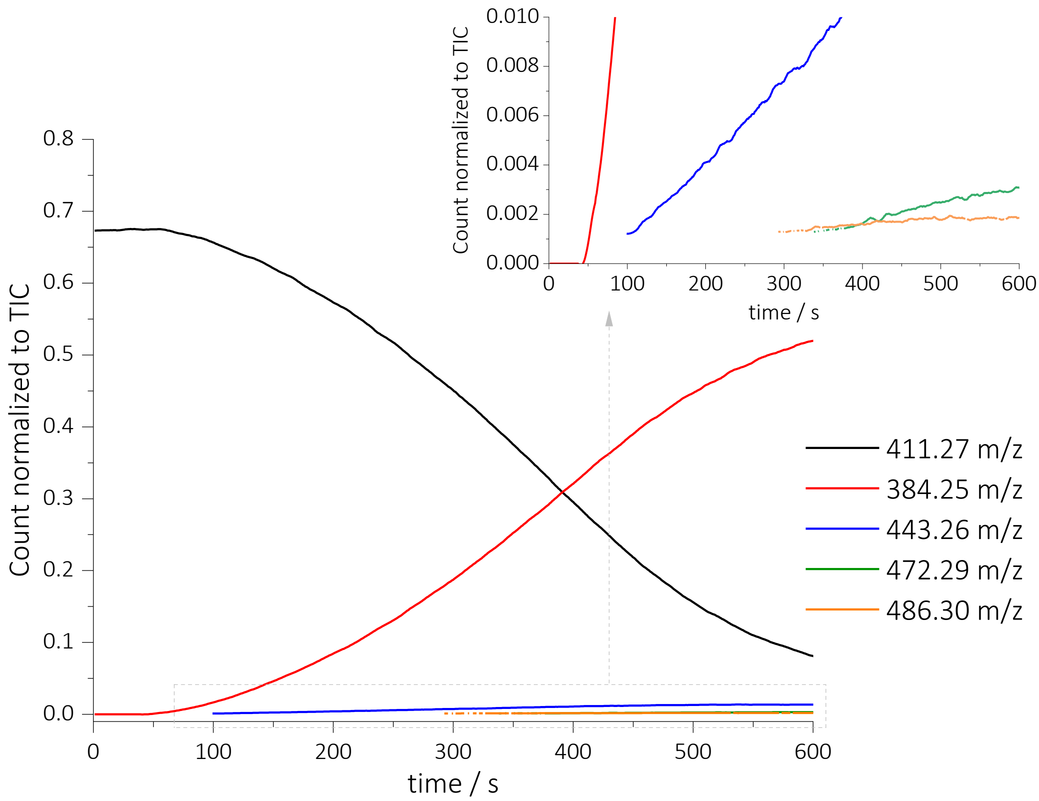

Figure S84. The time traces showing temporal evolution of 411.27 *m/z* (**Cy7-2',6'-*d*$_2$**), 384.25 *m/z* (**Cy5-2'-*d***), 443.26 *m/z* (**Cy7-2',6'-*d*$_2$-OOH**), 472.29 *m/z* and 486.30 *m/z* ions, from the HRMS experiment in Figure S76 (Irradiation of **Cy7** in **EA** buffer; 500 mM **EA**, pH = 8.7 prepared in H_2O).

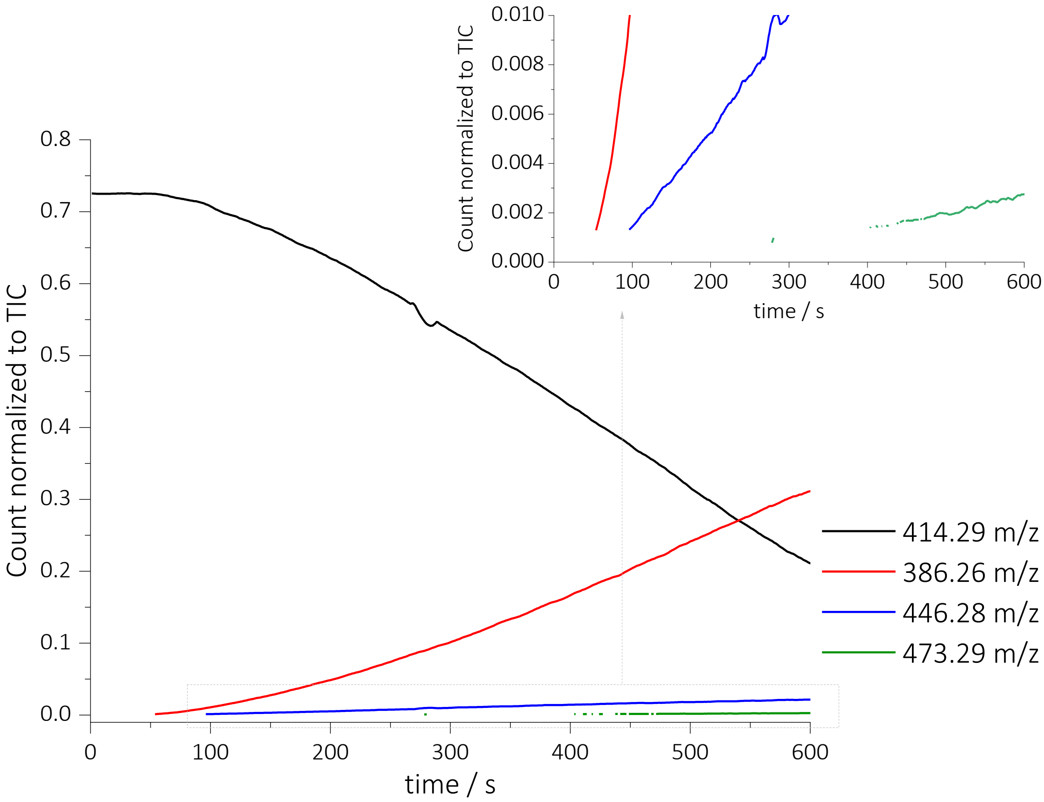

Figure S85. The time traces showing temporal evolution of 414.29 *m/z* (**Cy7-2'3'4'5'6'-*d*$_5$**), 386.26 *m/z* (**Cy5-2'3'4'-*d*$_3$**), 446.28 *m/z* (**Cy7-2'3'4'5'6'-*d*$_5$-OOH**), 473.29 *m/z* and 488.32 *m/z* ions, from the HRMS experiment in Figure S79 (Irradiation of **Cy7** in **EA** buffer; 500 mM **EA**, pH = 8.7 prepared in H_2O).

10.2. Crossover experiment

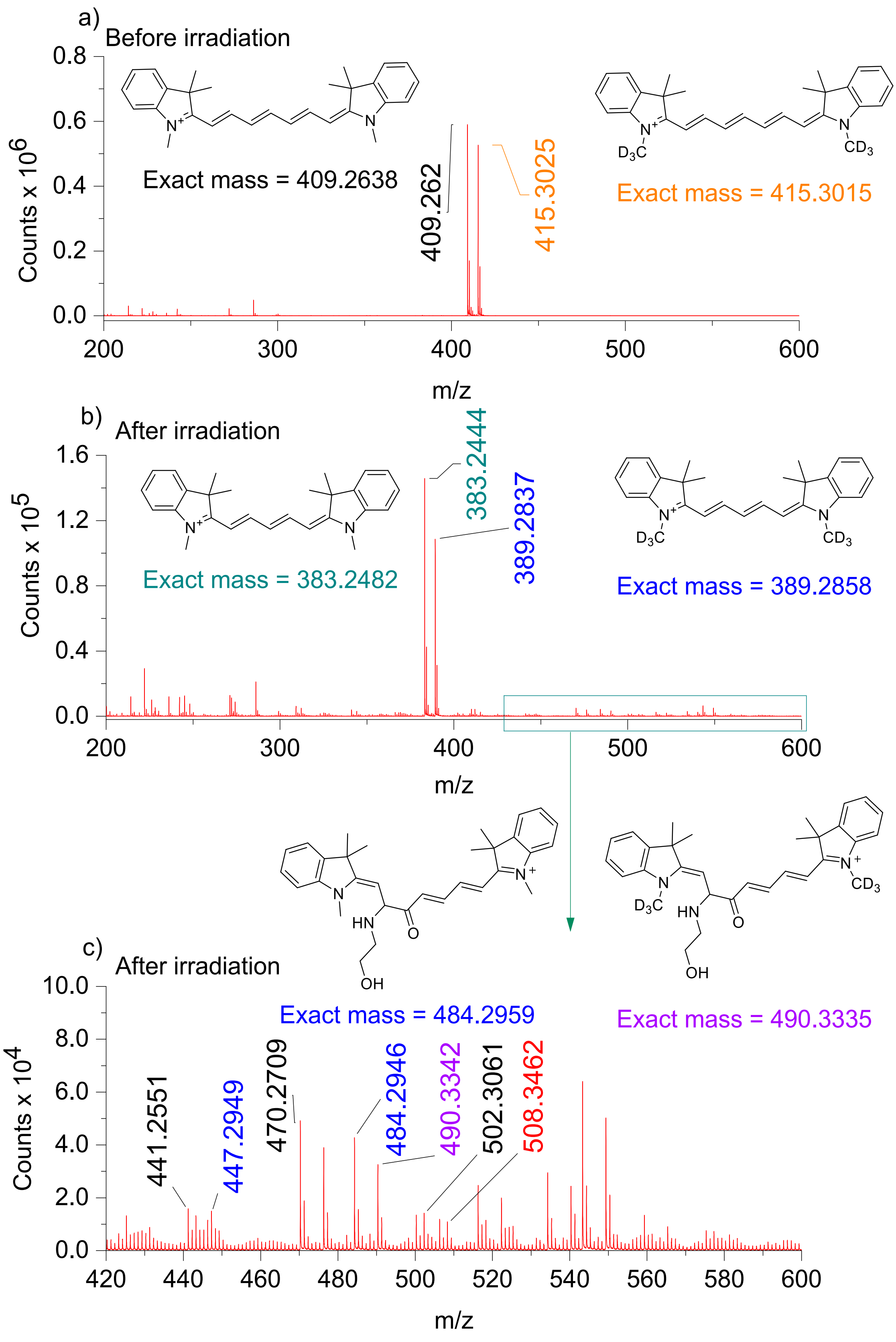

Figure S86. The HRMS spectra from the crossover experiment; a) before irradiation (**Cy7**; 409.26 *m/z* and **Cy7-*d*$_6$** (dimethyl-*d*$_3$) 415.30 *m/z*), b) after irradiation (showing the conversion to **Cy5**; 383.24 *m/z*, and **Cy5-*d*$_6$** (dimethyl-*d*$_3$) 389.28 *m/z*), and c) zoomed view in the 420 – 600 *m/z* region of the HRMS spectra presented in b). Reaction conditions: **Cy7** (50 µM) and 50 µM **Cy7-*d*$_6$** (dimethyl-*d*$_3$) in **EA** buffer (500 mM, pH = 8.7).

10.3. The kinetic isotope effect

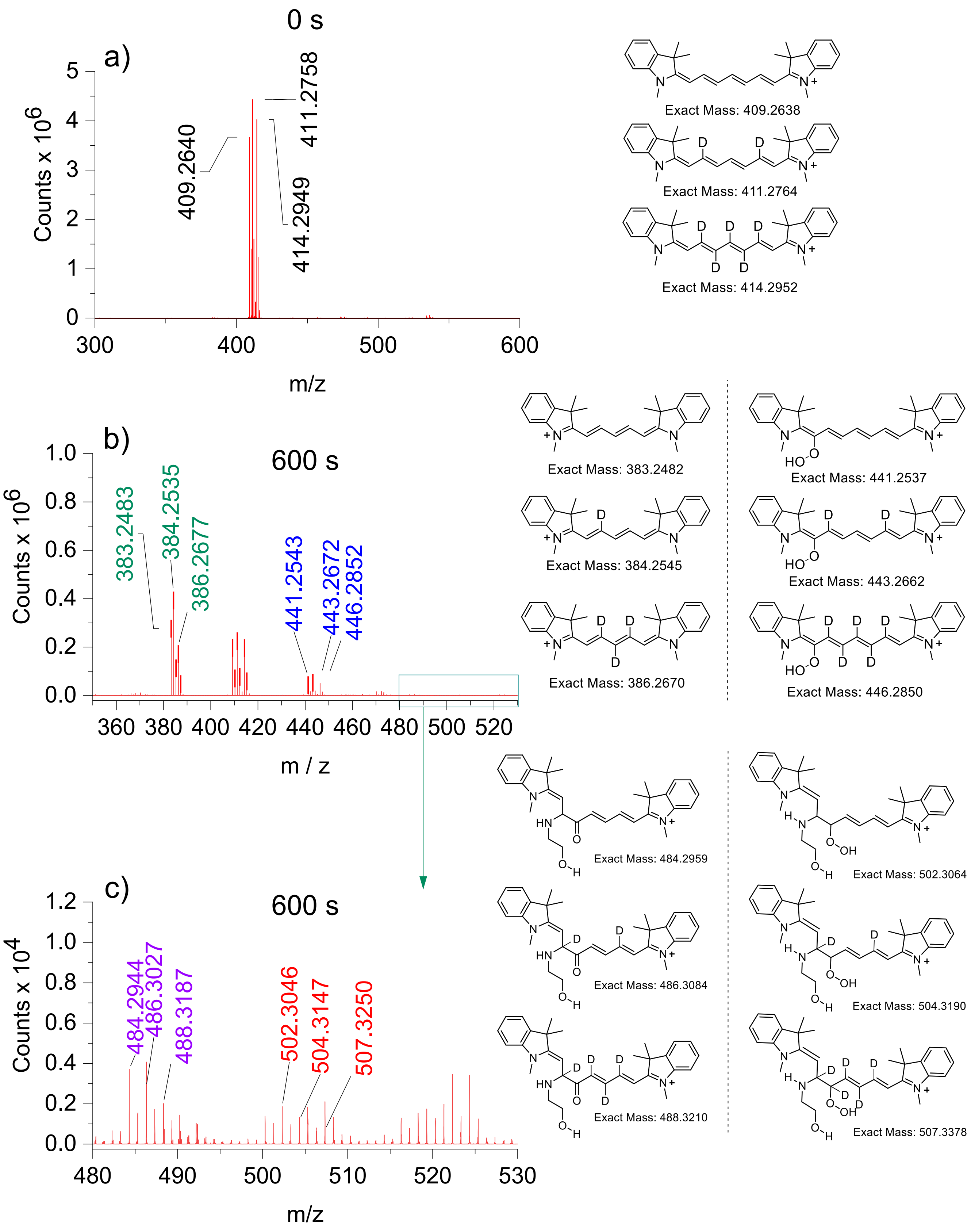

Figure S87. The HRMS experiment involving equimolar mixture of **Cy7**, **Cy7-2',6'-*d*$_2$**, and **Cy7-2'3'4'5'6'-*d*$_5$** (50 µM each) in **EA** buffer (500 mM, pH = 8.7): a) before irradiation, b) after 600 s irradiation, c) after 600 s irradiation, zoomed region 480 – 530 *m/z*.

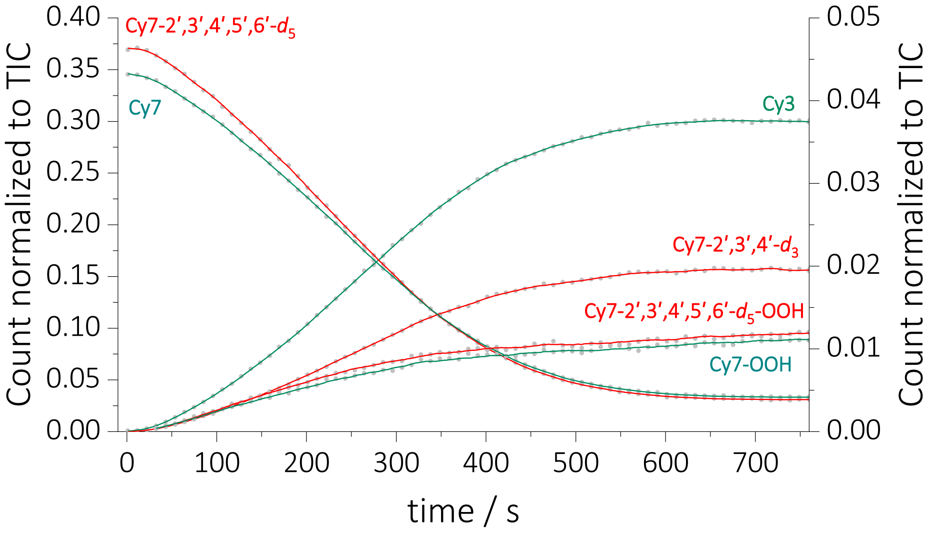

Figure S88. The time traces showing temporal evolution of **Cy7**, **Cy7-2’3’4’5’6’-*d*$_5$**, **Cy5**, **Cy5-2’3’4’-*d*$_3$**, **Cy7-OOH**, and **Cy7-2’3’4’5’6’-*d*$_5$**, from the HRMS experiment in Figure S87.

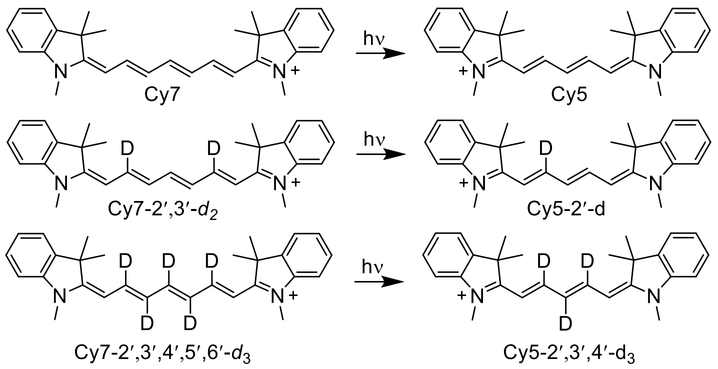

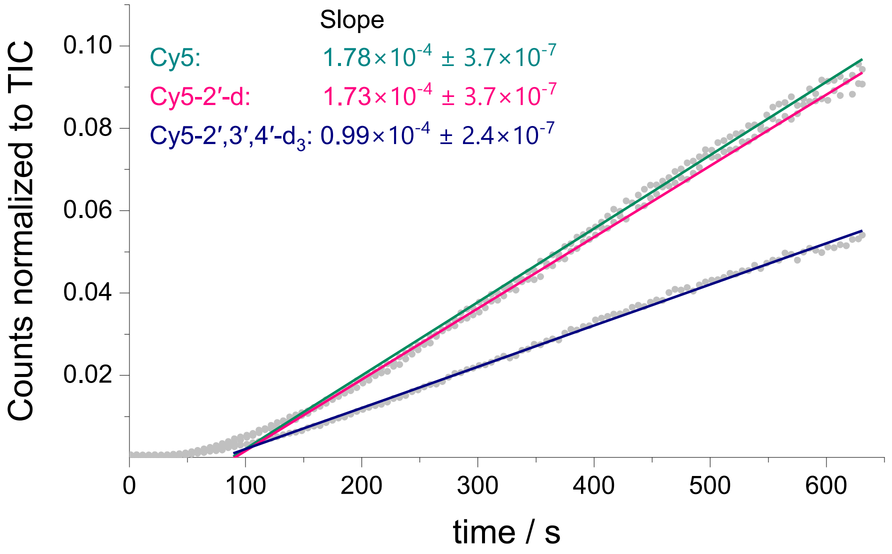

Figure S89. The time traces showing temporal evolution of **Cy5**, **Cy5-2’-*d***, and **Cy5-2’3’4’-*d*$_3$** from the HRMS experiments (Figure S87). Gray dots present experimental ion count normalized to the total ion count (TIC), colored full lines present a linear fit of the experimental data.

10.4. HRMS of Cy7 + N-acetylcysteine

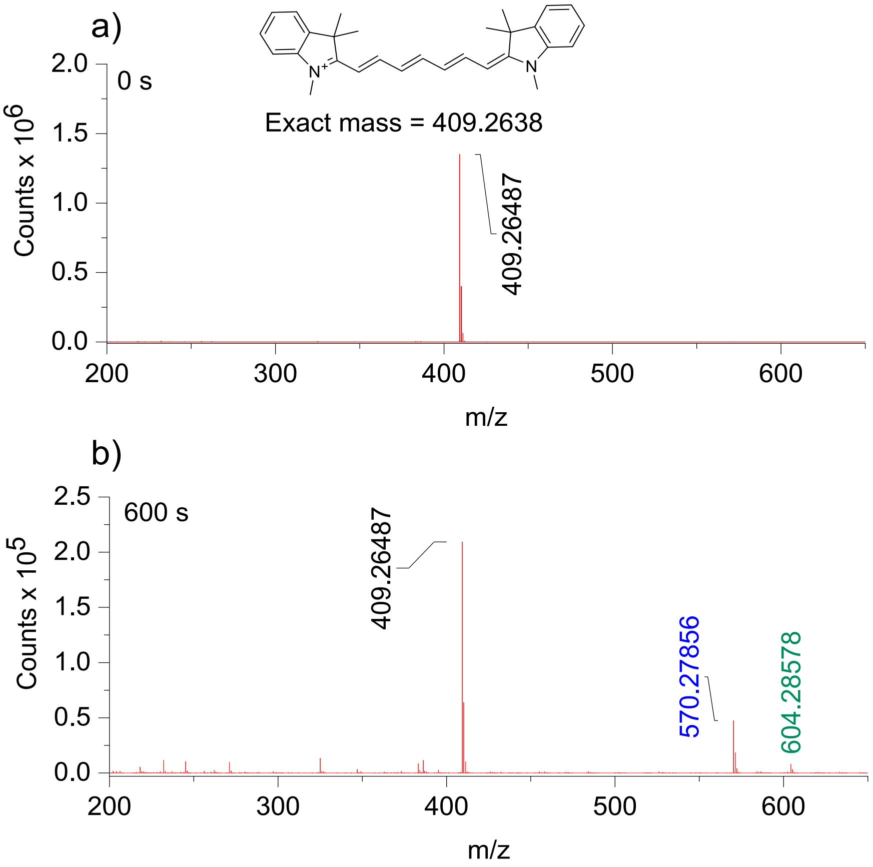

Figure S90. The HRMS spectra; a) before irradiation, and b) after 600 s of irradiation in **EA** buffer (500 mM, pH = 8.7) with addition of *N*-acetylcysteine (2 mM), irradiated in a flow photoreactor (Figure S62).

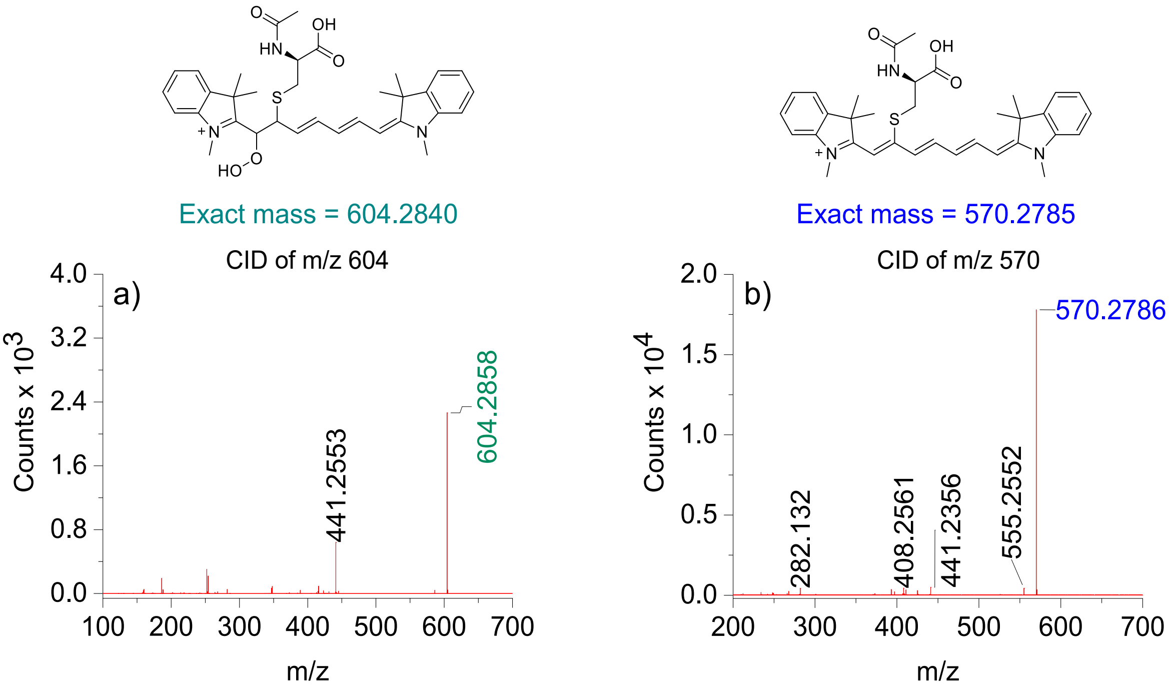

Figure S91. The CID spectra of a) 570.27 *m/z*, and b) 604.28 *m/z* ions, from the HRMS experiment in Figure S90.

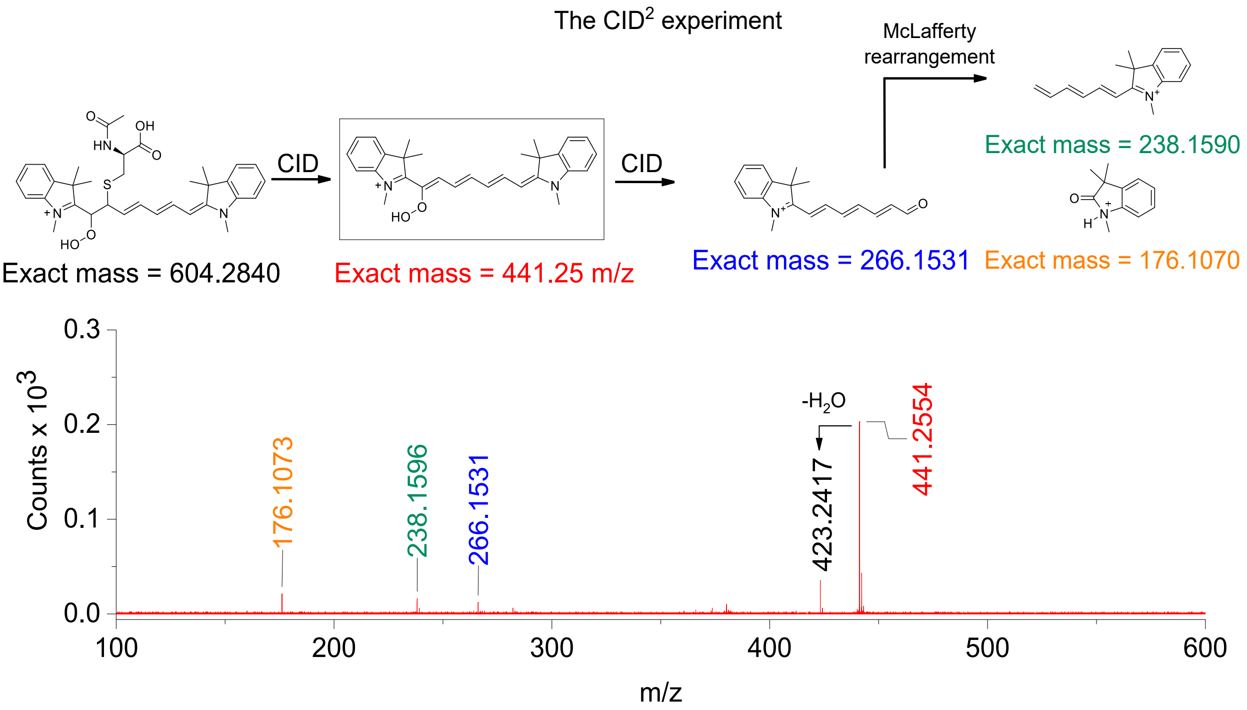

Figure S92. The CID^2 experiment. The CID of 441.25 *m/z* ion produced in the CID experiment of the 604.28 *m/z* ion (see Figure S90 for CID of 604.28 *m/z* ion)

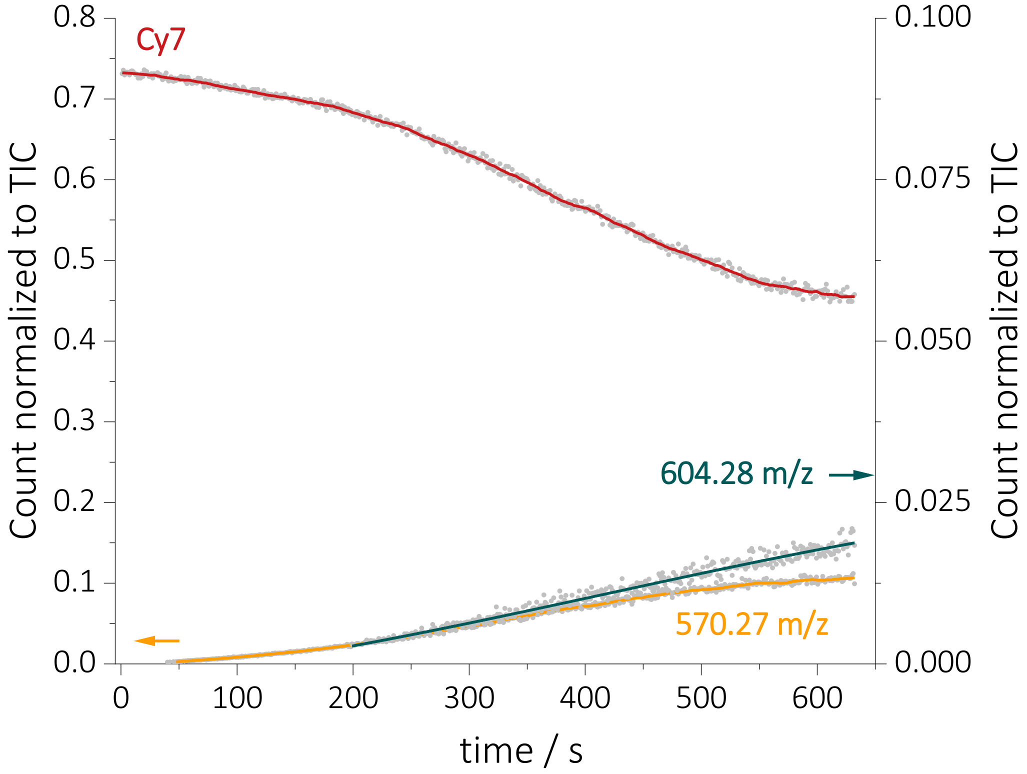

Figure S93. The time traces showing temporal evolution of **Cy7** (409.26 *m/z* (**Cy7**), 570.27 *m/z* and 604.28 *m/z* ions.

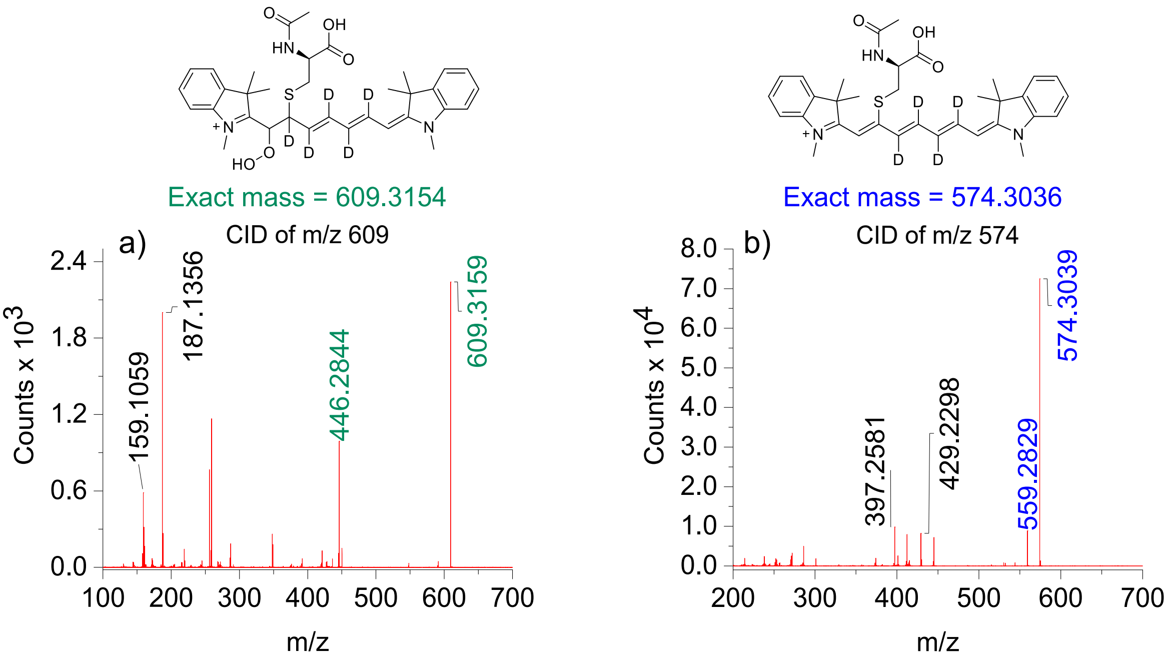

Figure S94. The CID spectra of a) 609.31 *m/z*, and b) 574.30 *m/z* ions, from the HRMS experiment with **Cy7-2′,3′,4′,5′,6′-*d*$_5$** in **EA** buffer (500 mM, pH = 8.7) with addition of *N*-acetylcysteine (2 mM).

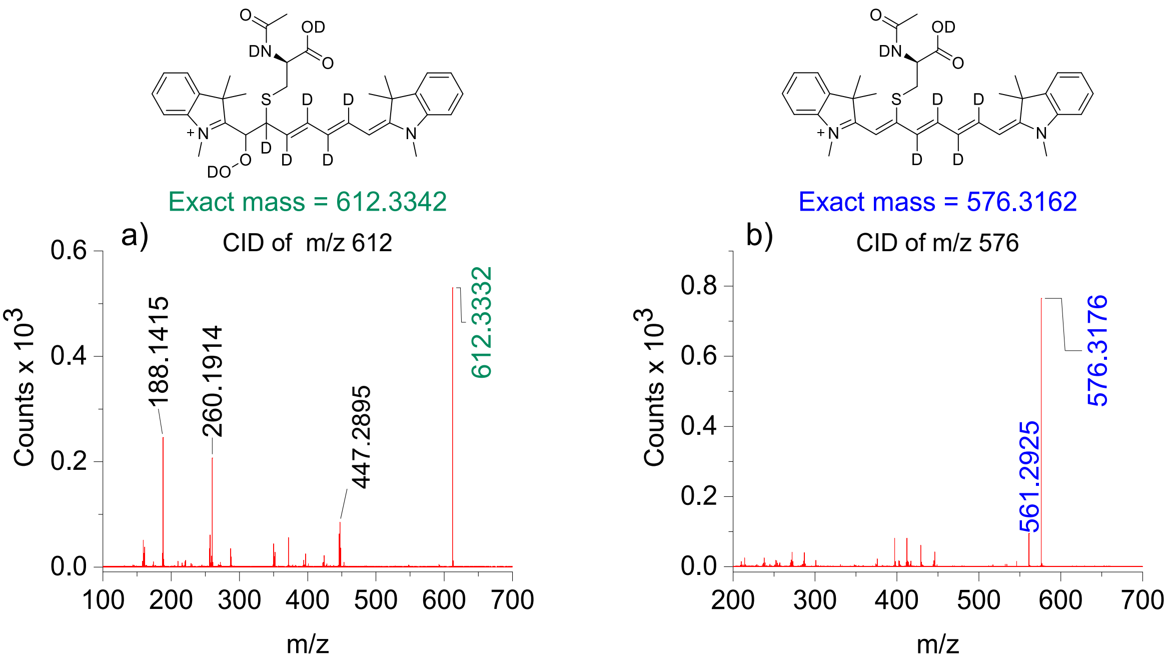

Figure S95. The CID spectra of a) 609.31 *m/z*, and b) 574.30 *m/z* ions, from the HRMS experiment with **Cy7-2′,3′,4′,5′,6′-*d*$_5$** in **EA** buffer (500 mM, pH = 8.7) prepared in D_2O with addition of *N*-acetylcysteine (2 mM).

10.5. HRMS of Cy7 irradiated in PBS buffer

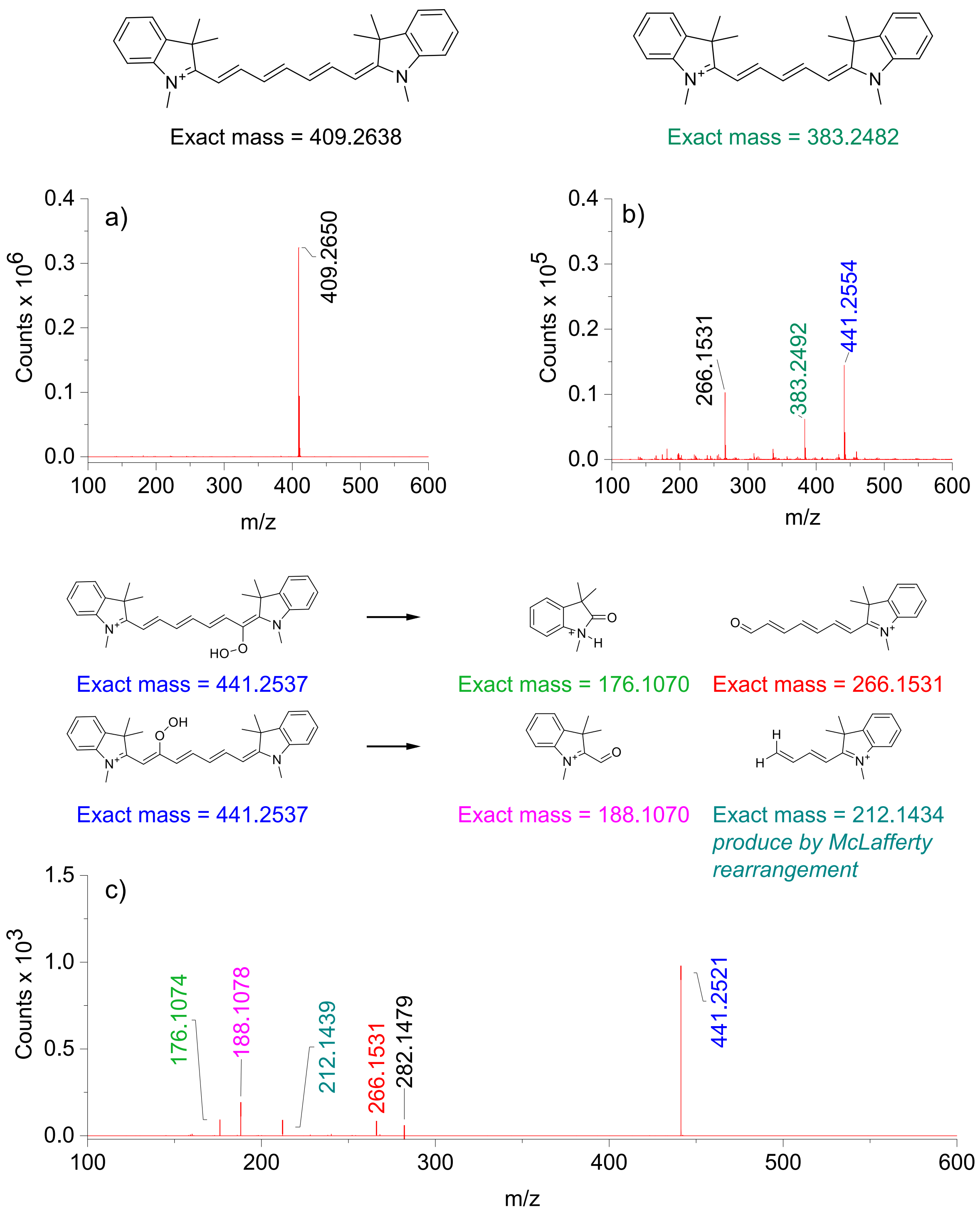

Figure S96. The HRMS analysis for irradiation of **Cy7** (50 µM) in PBS buffer (10 mM, pH = 7.4): a) before irradiation, b) after 600 s of irradiation. c) The CID spectra of a) 441.25 *m/z* ion.

10.6. HRMS of Cy7 and derivatives with endoperoxide

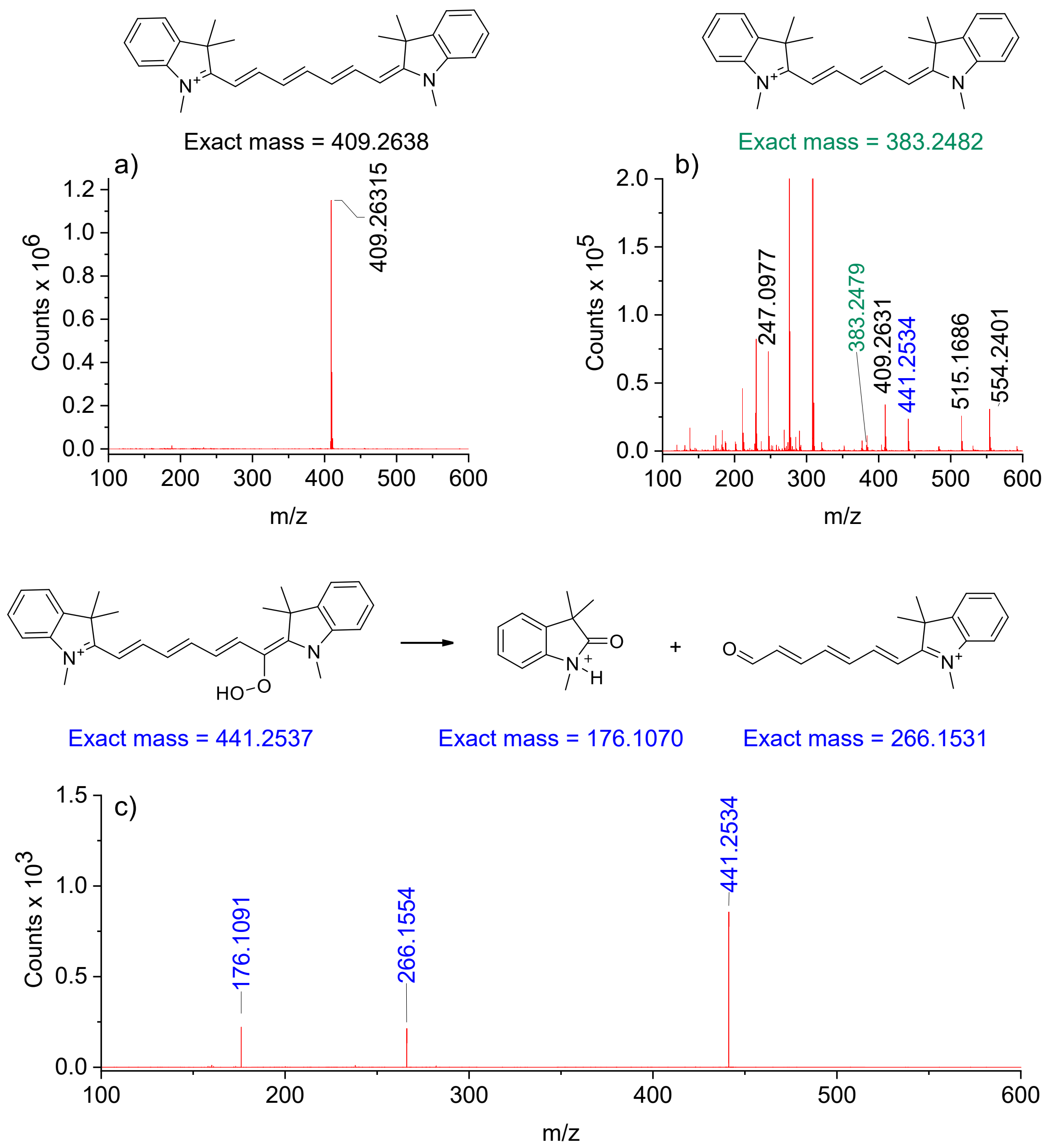

Figure S97. The HRMS analysis for reaction of **Cy7** (50 µM) with 1-methylnaphthalene-4-propionate endoperoxide (10 mM) in **EA** buffer (500 mM, pH = 8.7): a) before addition of endoperoxide, b) 15 min after the addition of 1-methylnaphthalene-4-propionate endoperoxide at 30 °C. c) The CID spectra of a) 441.25 *m/z* ion.

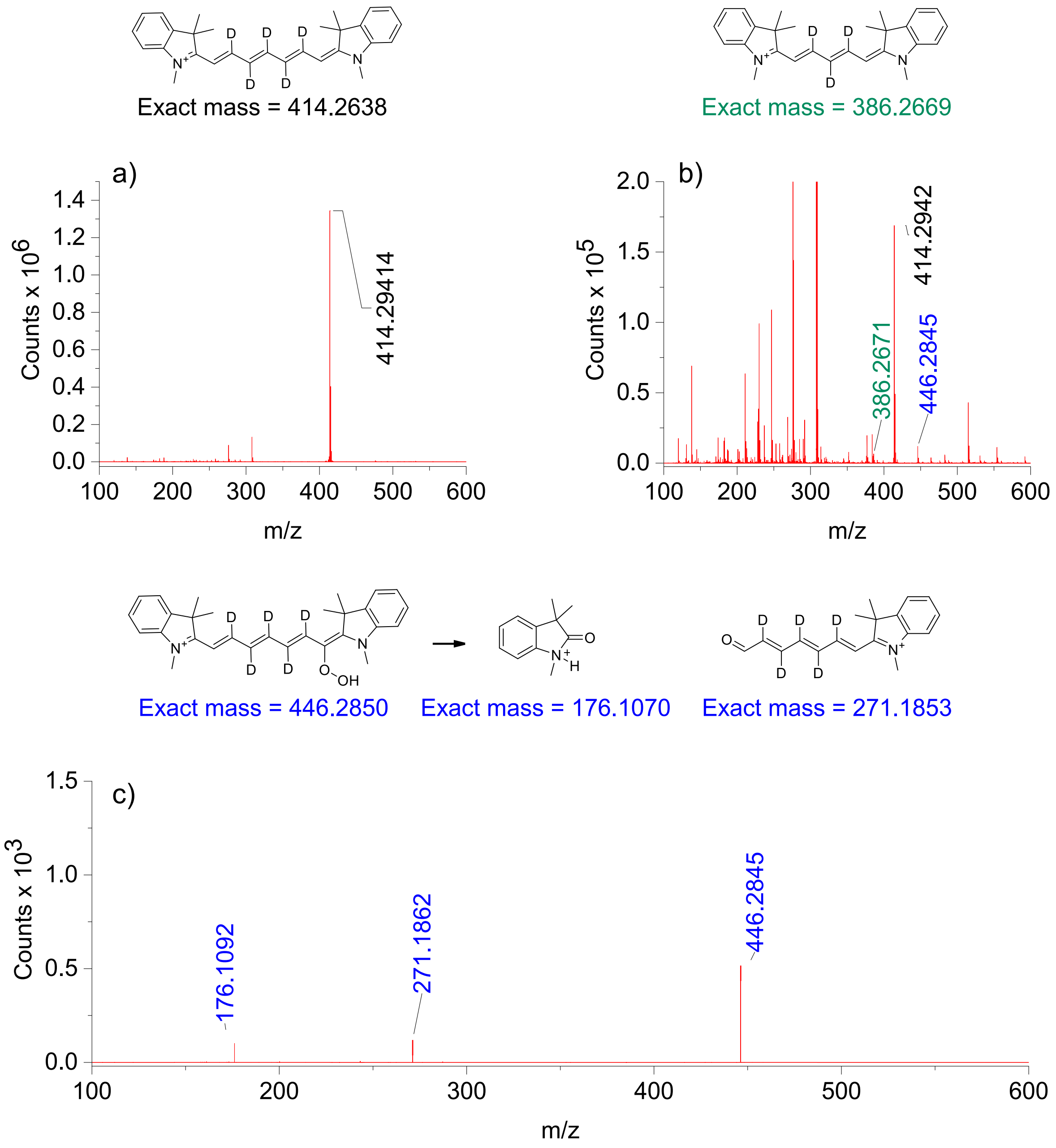

Figure S98. The HRMS analysis for reaction of **Cy7-2´,3´,4´,5´,6´-*d*$_5$** (50 µM) with 1-methylnaphthalene-4-propionate endoperoxide (10 mM) in **EA** buffer (500 mM, pH = 8.7): a) before addition of endoperoxide, b) 15 min after the addition of 1-methylnaphthalene-4-propionate endoperoxide at 30 °C. c) The CID spectra of a) 446.28 *m/z* ion.

10.7. Crossover experiment with Cy5

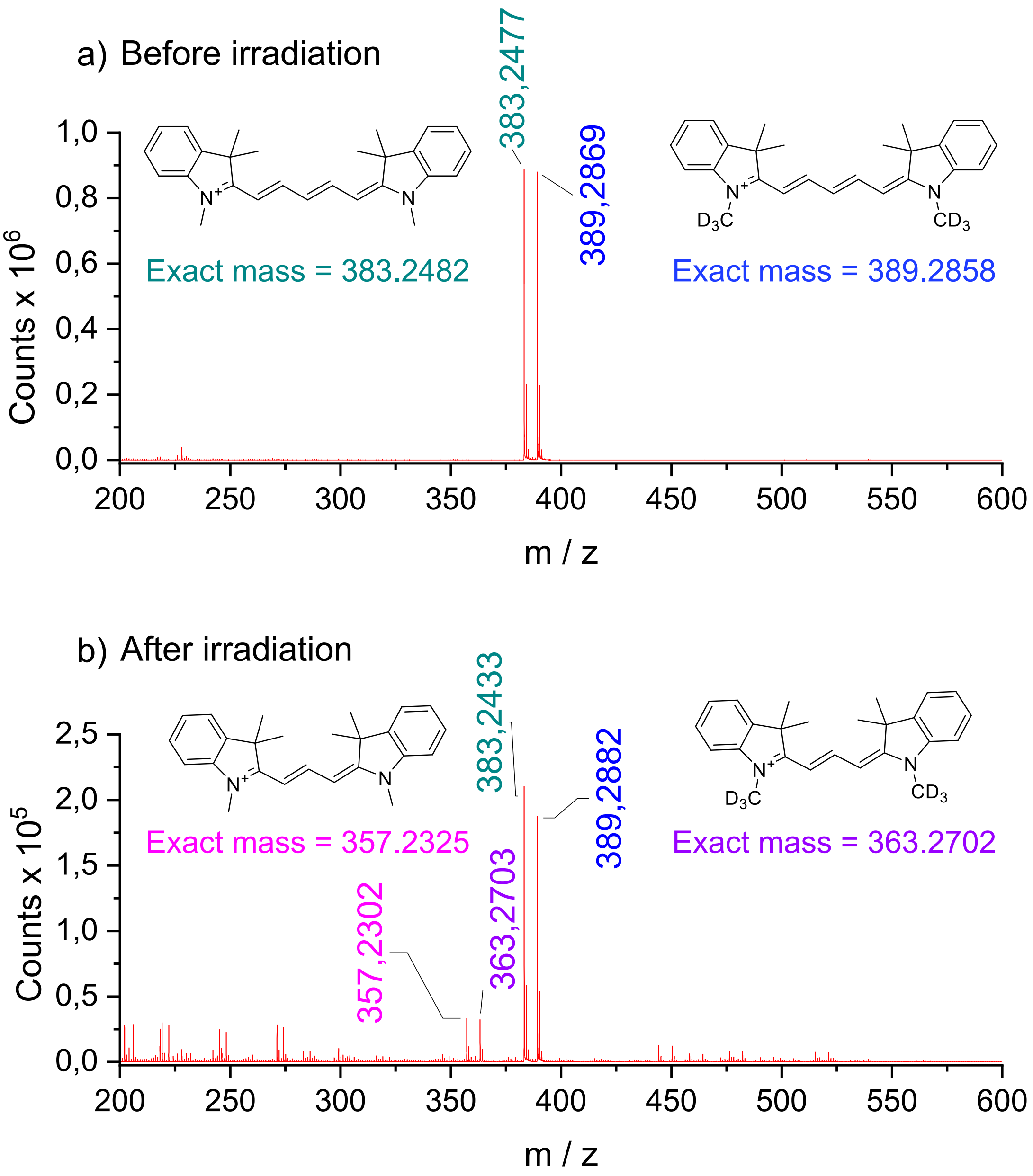

Figure S99 The HRMS experiment involving an equimolar mixture of **Cy5** and **Cy5-*d*₆** (dimethyl-d_6; 10 µM each) in **EA** buffer (500 mM, pH = 8.7): a) before irradiation, b) after irradiation with 625 nm LEDs.

10.8. Mobilograms of selected ions

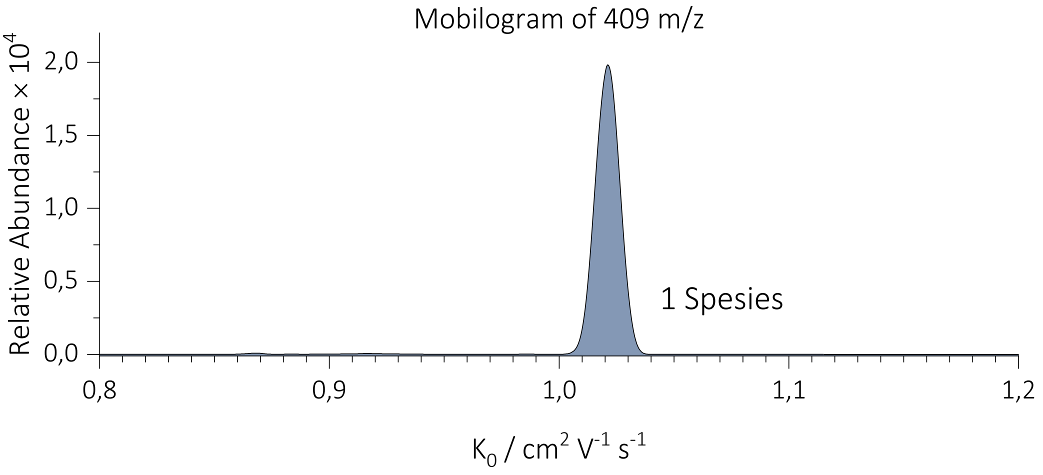

Figure S100. The deconvoluted mobilograms of 409.26 *m/z* ion, mobilogram was obtained from the HRMS experiment shown in Figure S67.

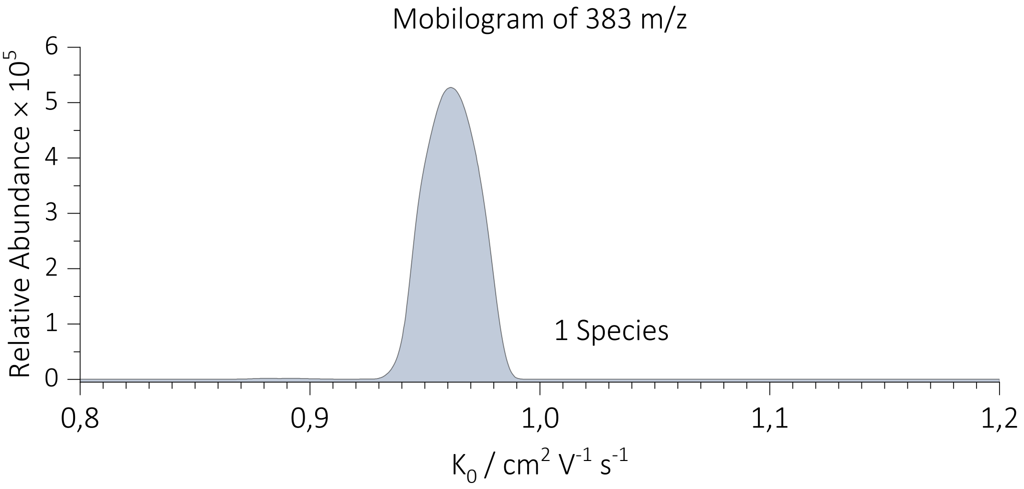

Figure S101. The deconvoluted mobilograms of 383.24 *m/z* ion, mobilogram was obtained from the HRMS experiment shown in Figure S67.

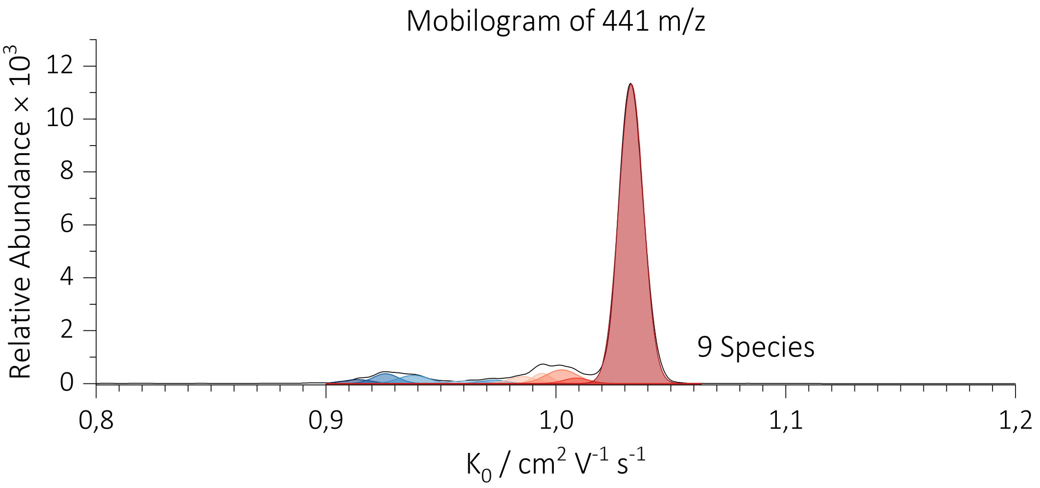

Figure S102. The deconvoluted mobilograms of 441.25 *m/z* ion, mobilogram was obtained from the HRMS experiment shown in Figure S67.

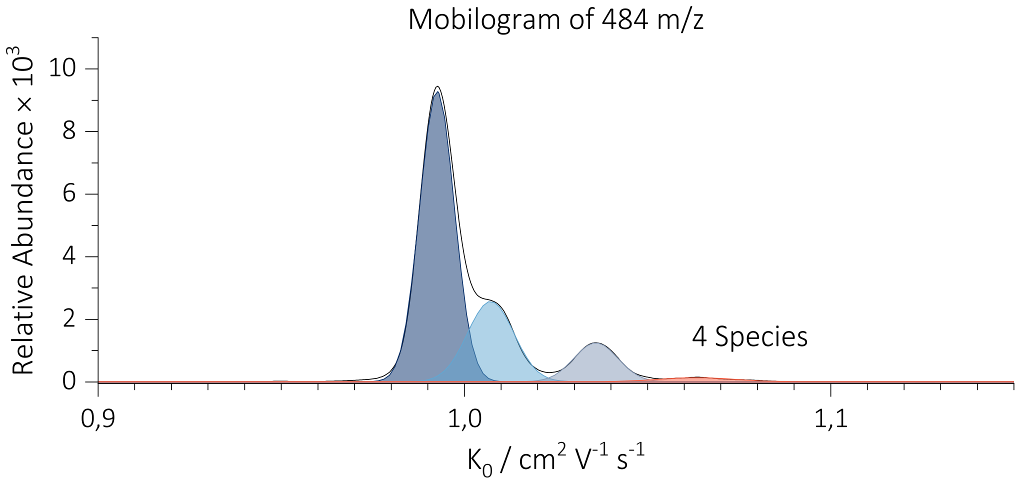

Figure S103. The deconvoluted mobilograms of 485.29 *m/z* ion, mobilogram was obtained from the HRMS experiment shown in Figure S67.

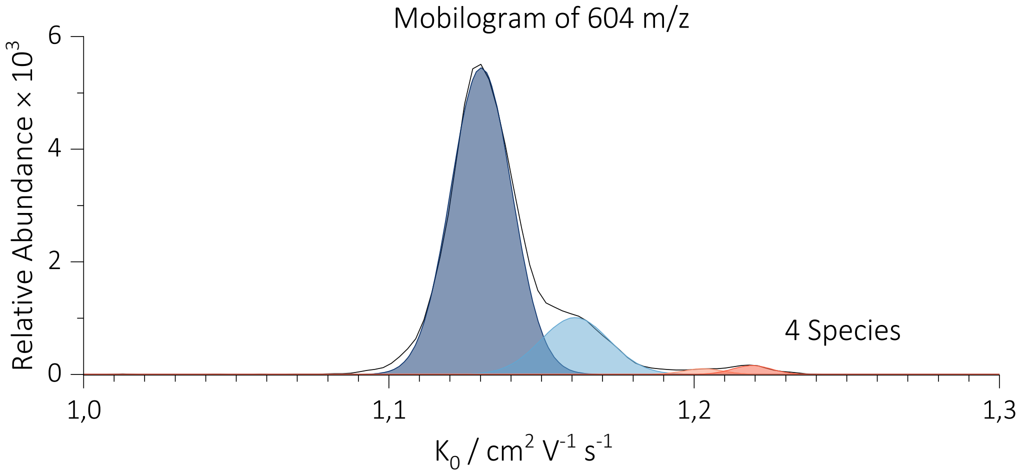

Figure S104. The deconvoluted mobilograms of 604.28 *m/z* ion, mobilogram was obtained from the HRMS experiment shown in Figure S90.

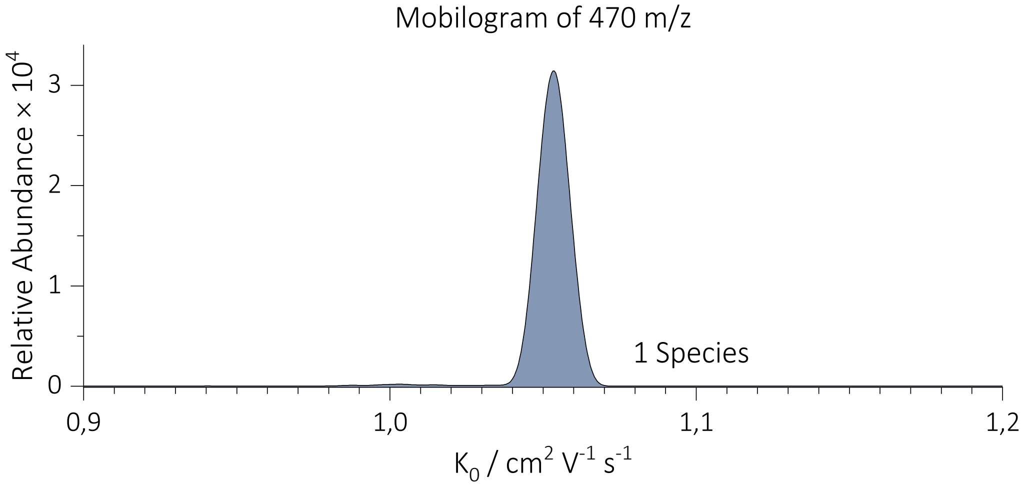

Figure S105. The deconvoluted mobilograms of 470.28 *m/z* ion, mobilogram was obtained from the HRMS experiment shown in Figure S67.

10.9. HRMS detection of 10a

Several spectroscopic methods were considered for detecting **10a**. However, it would be helpful to consider the maximum possible concentration of this species. Irradiating **Cy7** at a concentration of 10 µM results in a chemical yield of **Cy5** of ~30% under the best conditions. Assuming that **10a** is a stable molecule that accumulates in the solution, this would give a maximum obtainable concentration of **10a** of ~3 µM. Moreover, the concentration of **EA** is several orders of magnitude higher (500 mM), and due to a possible spectroscopically similar signature, strong interference was expected. Of all the methods, HRMS was considered the most suitable. We performed experiments with deuterium-labeled **Cy7** derivatives to detect the isotopomers of **10a** and search for follow-up products of **10a** with an appropriate isotopic pattern. To slow possible degradation upon formation, we tested the reaction at a low temperature (4 °C). We used sodium ions as tags for **10a** by adding 2 mM of $NaPF_6$ to the reaction mixture. This approach resulted in regularly spaced clusters with decreasing abundance, as observed by HRMS. Upon closer analysis, we concluded that the clusters resulted from **EA**-Na^+ adducts rather than the expected **10a**-Na^+ adducts. In CAPSO buffer and $NaPF_6$ (2 mM), only CAPSO-Na^+ adducts were observed. Attempting to decrease the concentration of the interfering compound (**EA**) resulted in much lower yields of **Cy5** (therefore, lowering the amount of **10a**). Lastly, we synthesized **10b**, the keto form of **10a**, as a reference compound to perform HRMS analysis with standard addition aiming to minimize the matrix effect. However, even that strategy did not provide conclusive proof of **10a**.

11. Helium tagging photodissociation spectroscopy

Helium tagging photodissociation spectroscopy was used to obtain visible photodissociation (VisPD) and infrared photodissociation (IRPD) spectra of selected ions with an Infrared Spectroscopy of Reaction Intermediates (ISORI) instrument equipped with an electrospray ionization (ESI) source. The ions of interest are mass-selected by a quadrupole mass filter and transferred to a cryogenic ion trap operated at 3 K. The thermalized ions attach helium atoms, and these helium-tagged ions are used for spectroscopic measurements. The ions were irradiated in alternative cycles using OPO/OPA pumped by a Nd:YAG laser (600–4700 cm^{-1} tuning range, FWHM ~1.5 cm^{-1}, 10 ns pulse length). After irradiation, the ions were extracted, mass analyzed by a quadrupole, and detected by a Daly type detector. The VisPD/IRPD spectra are constructed as $1–N_{(vi)}/N_0$, where $N_{(vi)}$ is the number of helium complexes after the irradiation, and N_0 is the number of helium complexes in alternative cycles without the irradiation.

11.1. Visible photodissociation spectroscopy (VisPD)

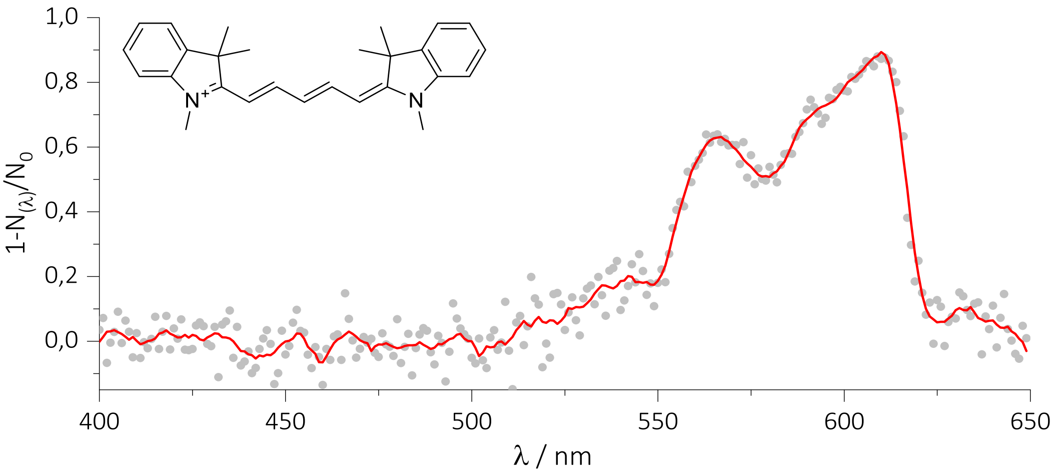

Figure S106. The experimental VisPD spectrum (gray dots) of ion with 383 *m/z* (**Cy5**); red line is the Savitzky-Golay average (points of window: 20, polynomial order: 2).

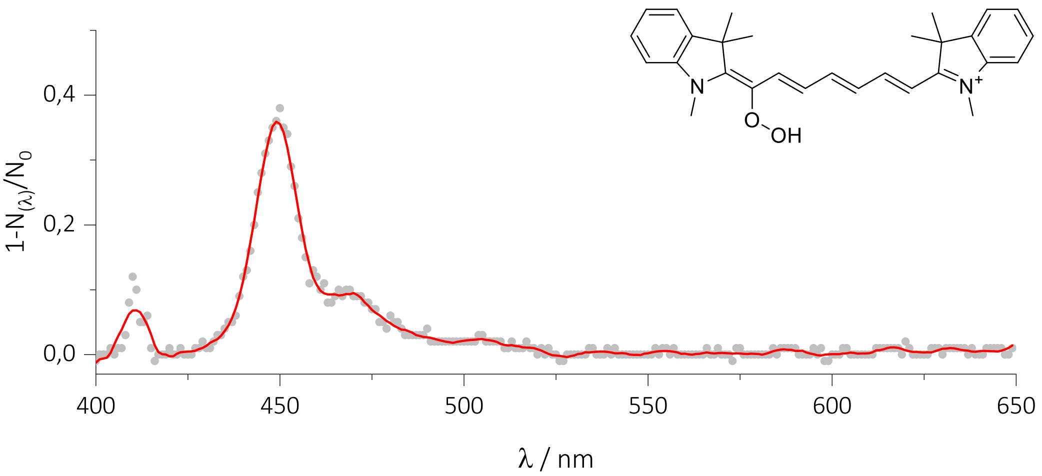

Figure S107. The experimental VisPD spectrum (gray dots) of ion with 441 *m/z* (note that there are 9 species with this *m/z*, see Figure S102), red line is the Savitzky-Golay average (points of window: 20, polynomial order: 2).

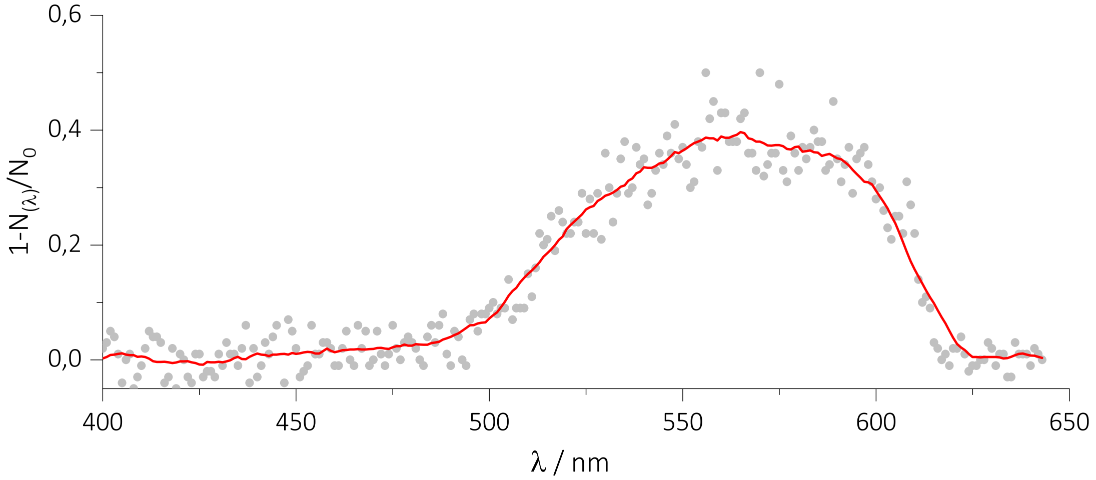

Figure S108. The experimental VisPD spectrum (gray dots) of ion with 470 *m/z*, red line is the Savitzky-Golay average (points of window: 20, polynomial order: 2).

11.2. Infrared Photodissociation Spectroscopy (IRPD)

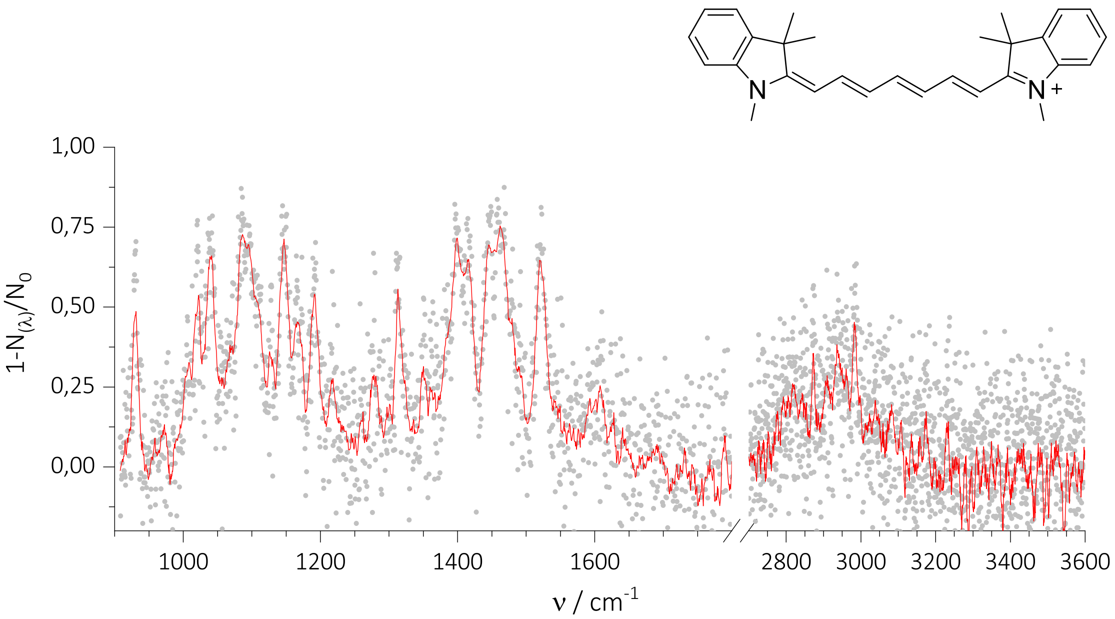

Figure S109. The experimental IRPD spectrum (gray dots) of ion with 409 *m/z* (**Cy7**); red line is the Savitzky-Golay average (points of window: 20, polynomial order: 2).

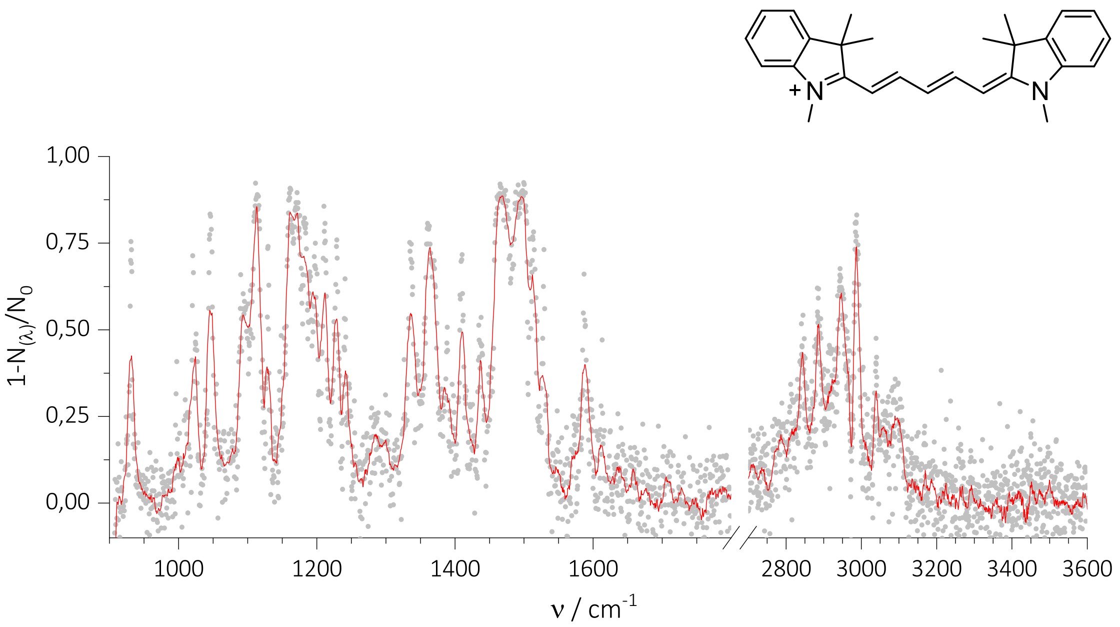

Figure S110. The experimental IRPD spectrum (gray dots) of ion with 383 *m/z* (**Cy5**); red line is the Savitzky-Golay average (points of window: 20, polynomial order: 2).

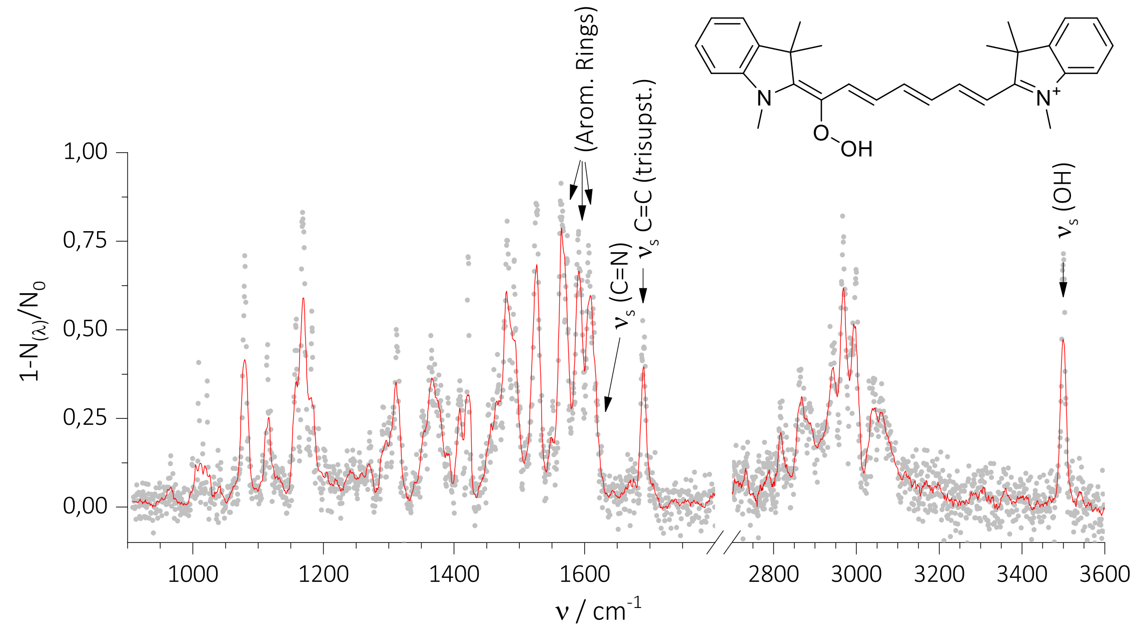

Figure S111. The experimental IRPD spectrum (gray dots) of ion with 441 *m/z* (note that there are 9 species, one of them with this *m/z*, see Figure S102); red line is the Savitzky-Golay average (points of window: 20, polynomial order: 2).

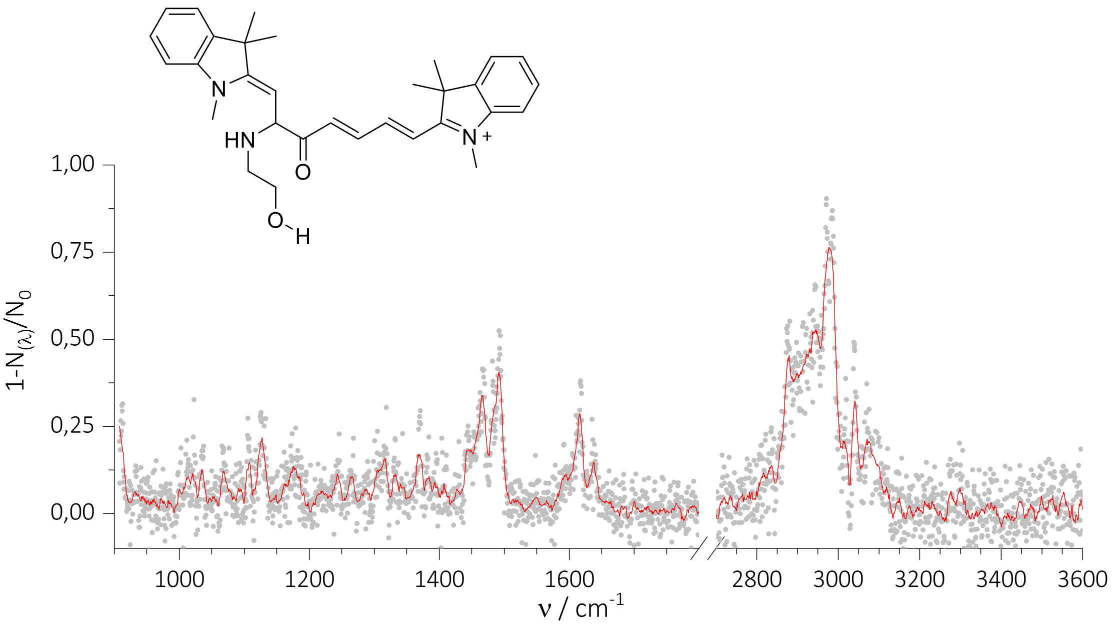

Figure S112. The experimental IRPD spectrum (gray dots) of ion with 484 *m/z* (note that there are 4 species with this *m/z*, see Figure S103); red line is the Savitzky-Golay average (points of window: 20, polynomial order: 2).

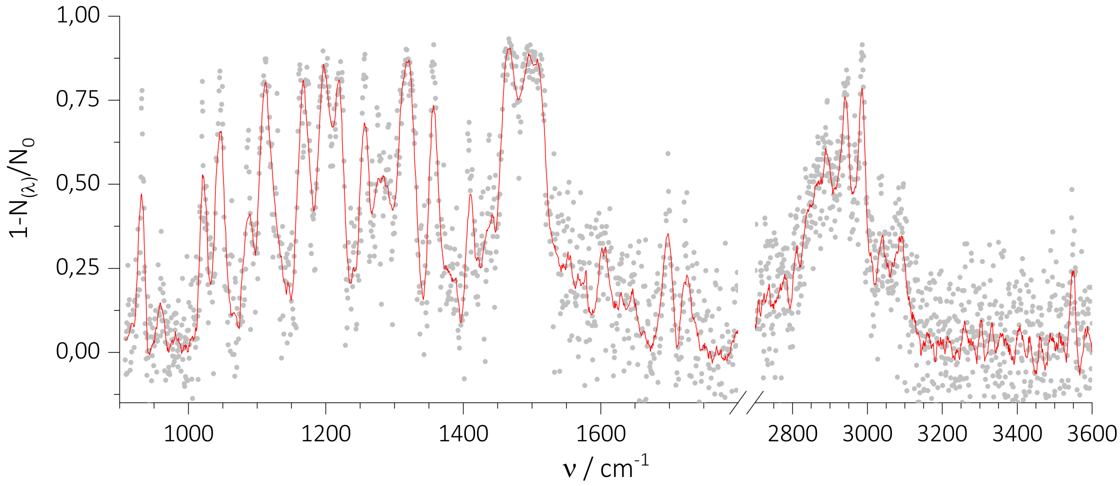

Figure S113. The experimental IRPD spectrum of ion with 470 *m/z* (note that there is only one species with this *m/z*, see Figure S105), red line is the Savitzky-Golay average (points of window: 20, polynomial order: 2).

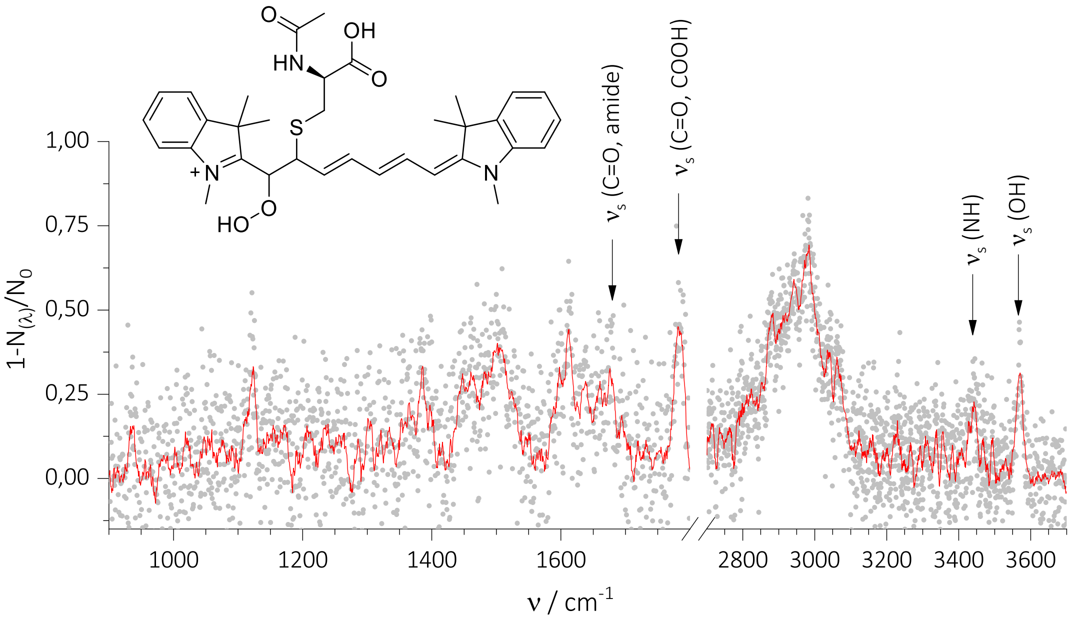

Figure S114. The experimental IRPD spectrum of ion with 604 *m/z* (note that there are 4 species with this *m/z*, see Figure S104); red line is the Savitzky-Golay average (points of window: 20, polynomial order: 2).

12. Synthesis of Cyanine derivatives

12.1. Synthesis of Cy7-3′,5′-d_2

The **Cy7-3′,5′-*d*$_2$** synthesis is shown in Scheme S2. Briefly, commercially available 3,5-dibromopyridine (**10**) was converted to pyridine-3,5-*d*$_2$ *via* lithium–halogen exchange followed by the reaction with D_2O, and the crude product was converted to the corresponding Zincke salt (**13**, Figure S115 and S116). The final **Cy7-3′,5′-*d*$_2$** was obtained by the reaction of **13** with 1,2,3,3-tetramethyl-3*H*-indolium iodide (**14**) according to previously published procedure;[1] the crude cyanine derivative was purified by flash column chromatography (Figures S117 and S118).

Scheme S2. The synthesis of **Cy7**-3′,5′-*d*$_2$.

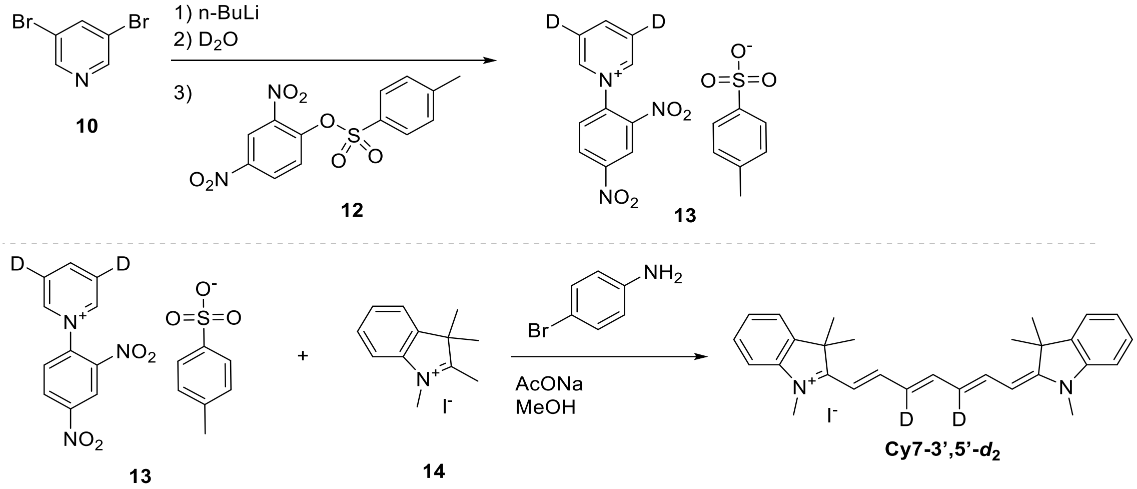

Preparation of 1-(2,4-Dinitrophenyl)pyridin-1-ium-3,5-d$_2$ (13).

Pyridine-3,5-d$_2$. 3,5-Dibromopyridine (**10**, 3.00 g, 12.66 mmol) in anhydrous Et_2O (40 mL) was added dropwise to a solution of *n*-BuLi (2.5 M in hexane, 13.5 mL, 1.2 mmol) in dry Et_2O (15 mL) under inert atmosphere at –78°C during 20 min. The resulting solution was stirred at –78°C for 1 h, after which D_2O (1.5 mL, 75.98 mmol) was added to the solution, and the reaction mixture was allowed to warm up to room temperature. The product (pyridine-3,5-*d*$_2$) was extracted with Et_2O (3 × 40 mL), the organic layers were combined and dried over Na_2SO_4, and the solvent was evaporated under reduced pressure. The crude product was used without purification in the next step.
Zincke salt (13); A mixture of 2,4-dinitrophenyl-*p*-toluenesulfonate (**2**, 229 mg, 0.67 mM) and pyridine-3,5-*d*$_2$ from the previous step (50 mg, 0.61 mM) were dissolved in toluene (8 mL). The reaction mixture was refluxed for 16 h, after which the precipitate was formed upon cooling to room temperature. The resulting precipitate was filtered, washed with toluene (2 × 5 mL), Et_2O (2×5 mL), and dried to give a white solid (**13**). Yield: 130 mg (32%). ^{1}H NMR (500 MHz, DMSO) δ 9.38 (d, *J* = 1.2 Hz, 2H), 9.14 (d, *J* = 2.5 Hz, 1H), 9.03 – 8.89 (m, 2H), 8.43 (dd, *J* = 12.9, 8.2 Hz, 1H), 7.47 (d, *J* = 8.0 Hz, 2H), 7.11 (d, *J* = 7.9 Hz, 2H), 2.29 (s, 3H). ^{13}C NMR (126 MHz, DMSO) δ 149.7, 149.1, 146.5, 143.6, 139.2, 137.9, 132.3, 130.7, 128.5, 125.9, 121.9, 21.2. HRMS (ESI$^+$): calcd. for $C_{11}H_6D_2N_3O_4^+$ [M–pTsOH$^-$] 248.0635, found 248.0634.

*Preparation of 1,3,3-trimethyl-2-((1E,3E,5E)-7-((E)-1,3,3-trimethylindolin-2-ylidene)hepta-1,3,5-trien-1-yl-3,5-d$_2$)-3H-indol-1-ium iodide (*Cy7*-3′,5′-d$_2$).*

4-Bromoaniline (43 mg 0.25 mM) and **13** (50 mg, 0.11 mM) were dissolved in methanol (7 mL), and the reaction mixture was stirred at room temperature for 30 min. Next, 1,2,3,3-tetramethyl-3*H*-indolium iodide (**14**, 75 mg , 0.25 mM) and sodium acetate (58 mg, 0.71 mM) were added, and the reaction mixture was stirred for another 16 h at room temperature. Afterward, Et_2O (20 mL) was added, and the mixture was placed in a freezer (−16 °C). The resulting precipitate was filtered, washed with water (2 × 5 mL) and Et_2O (2 × 5 mL) and dried on air. The crude product was purified by flash column chromatography (silica gel, 5% methanol/dichloromethane); the solvents were removed under reduced pressure to give **15** as a green solid. Yield: 35 mg (54%). ^{1}H NMR (500 MHz, DMSO) δ 7.92 (d, *J* = 13.5 Hz, 2H), 7.58 (s, 1H), 7.48 (t, *J* = 9.8 Hz, 2H), 7.40 (t, *J* = 7.7 Hz, 2H), 7.25 (dd, *J* = 12.9, 7.7 Hz, 4H), 6.26 (d, *J* = 13.7 Hz, 2H), 3.59 (s, 6H), 1.69 (s, 12H). ^{13}C NMR (126 MHz, DMSO) δ 173., 144.4, 142.3, 132.7, 129.7, 126, 123.2, 111.6, 104.7, 50.3, 31.4, 27.9. HRMS (ESI$^+$): calcd. for $C_{29}H_{31}N_2^+$ [M–I$^-$] 411.2764, found 411.2743.

12.2. Synthesis of Cy7-d_6 (dimethyl-d_6)

The synthesis of **Cy7-*d*$_6$** (dimethyl-d_6) is shown in Scheme S3. Briefly, commercially available 2,3,3-trimethylindolenine (**15**) was converted to 2,3,3-trimethyl-1-(methyl-d_3)-3*H*-indol-1-ium iodide (**16;** Figures S119 and S120) by reaction with CD_3I. The final **Cy7-*d*$_6$** (dimethyl-d_6) was obtained by the reaction of **16** with 2,4-dinitrophenyl-*p*-toluenesulfonate (**17**) according to previously published procedure,[1] and the crude cyanine derivative was purified by flash column chromatography (Figures S121 and S122).

Scheme S3. The synthesis of **Cy7**-d_6 (dimethyl-d_6; **9**).

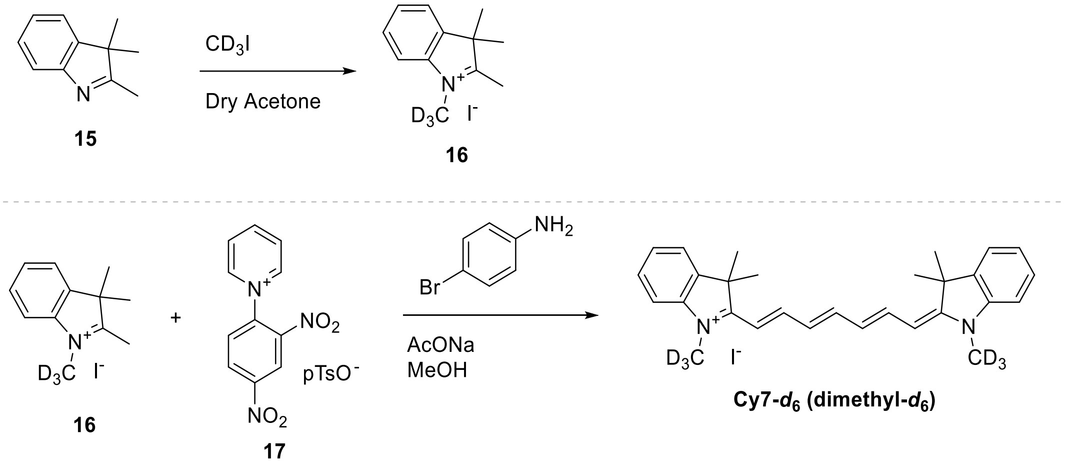

Preparation of 2,3,3-trimethyl-1-(methyl-d_3)-3H-indol-1-ium iodide (16)

A mixture of 2,3,3-trimethylindolenine (**15**, 350 mg, 2.20 mM) and CD_3I (956 mg, 6.60 mM) were dissolved in dry acetonitrile (10 mL). The reaction mixture was stirred at 80 °C overnight under inert atmosphere. The formed precipitate was filtered, washed with cold methanol, and recrystallized from methanol to give a pinkish solid (**16**). The Yield: 510 mg (76%). ^{1}H NMR (500 MHz, DMSO) δ 7.95 – 7.87 (m, 1H), 7.85 – 7.77 (m, 1H), 7.69 – 7.51 (m, 2H), 2.75 (s, 3H), 1.52 (s, 6H). ^{13}C NMR (126 MHz, DMSO) δ 196, 142, 142.1, 129.8, 129.3, 123.8, 115.6, 54.4, 22.2, 14.5. HRMS (ESI$^+$): calcd. for $C_{12}H_{13}D_3N^+$ [M–I$^-$] 177.1466, found 177.1481.

*Preparation of 2-((1E,3E,5E)-7-((E)-3,3-dimethyl-1-(methyl-d_3)indolin-2-ylidene)hepta-1,3,5-trien-1-yl)-3,3-dimethyl-1-(methyl-d_3)-3H-indol-1- ium iodide (*Cy7-d_6 (dimethyl-d_6)*).*

4-Bromoaniline (82 mg 0.47 mM) and **17** (100 mg, 0.23 mM) were dissolved in methanol (8 mL), and the reaction mixture was stirred at room temperature for 30 min. Next, 2,3,3-trimethyl-1-(methyl-d_3)-3H-indol-1-ium iodide (**16**, 153 mg , 0.50 mM) and sodium acetate (118 mg, 1.43 mM) were added, and the reaction mixture was stirred for another 16 h at room temperature. Afterward, Et_2O (20 mL) was added, and the mixture was placed in a freezer (−16 °C). The resulting precipitate was filtered, washed with water (2 × 5 mL) and Et_2O (2 × 5 mL), and dried on air. The crude product was purified by flash column chromatography (silica gel, 5% methanol/dichloromethane); the solvents were removed under reduced pressure to give **16** as a green solid. Yield: 90 mg (70%). ^{1}H NMR (500 MHz, MeOD) δ 7.95 (t, *J* = 12.9 Hz, 2H), 7.62 (d, *J* = 10.5 Hz, 1H), 7.48 (d, *J* = 7.3 Hz, 2H), 7.41 (t, *J* = 7.5 Hz, 2H), 7.34 – 7.22 (m, 4H), 6.57 (t, *J* = 12.0 Hz, 2H), 6.27 (d, *J* = 13.3 Hz, 2H), 1.71 (s, 12H). ^{13}C NMR (126 MHz, MeOD) δ 173.9, 152.9, 144.4, 142.4, 129.7, 126.9, 126.4, 123.2, 111.6, 104.7, 50.3, 27.9. HRMS (ESI$^+$): calcd. for $C_{29}H_{27}D_6N_2^+$ [M–I$^-$] 415.3015, found 415.3025.

12.3. NMR spectra

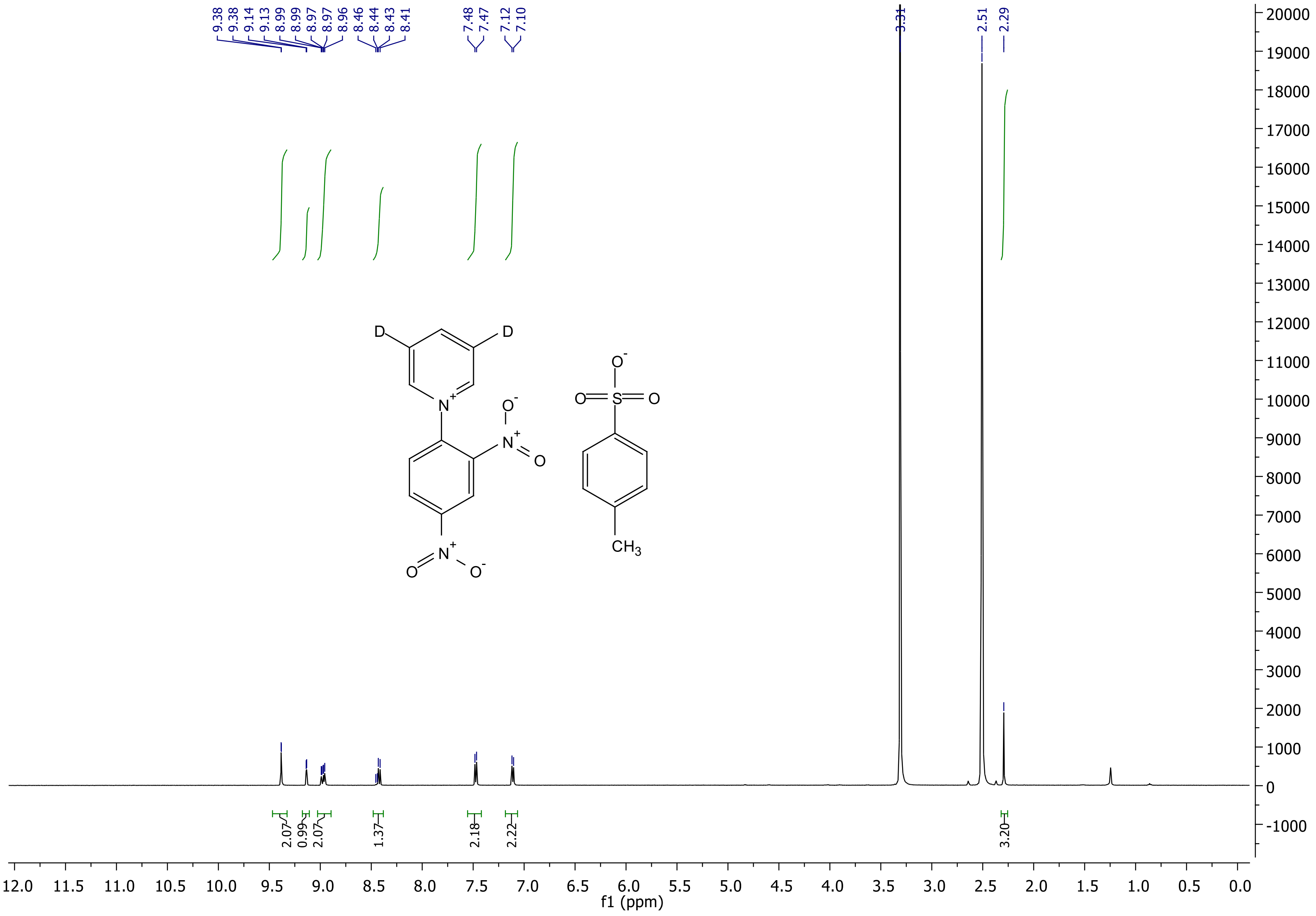

Figure S115. ^{1}H NMR (500 MHz, DMSO-d_6) of **13**.

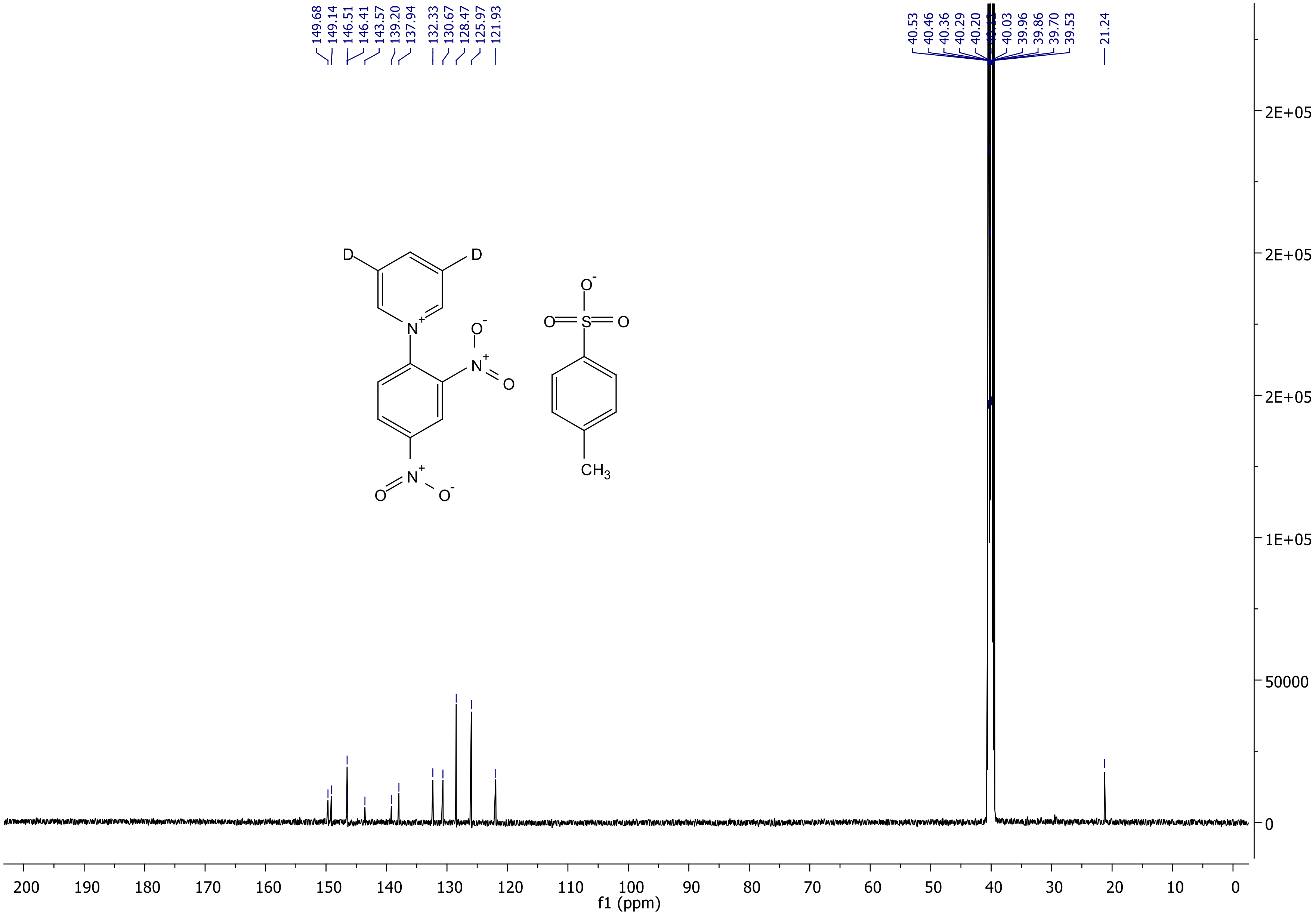

Figure S116. ^{13}C NMR (126 MHz, DMSO-d_6) of **13.**

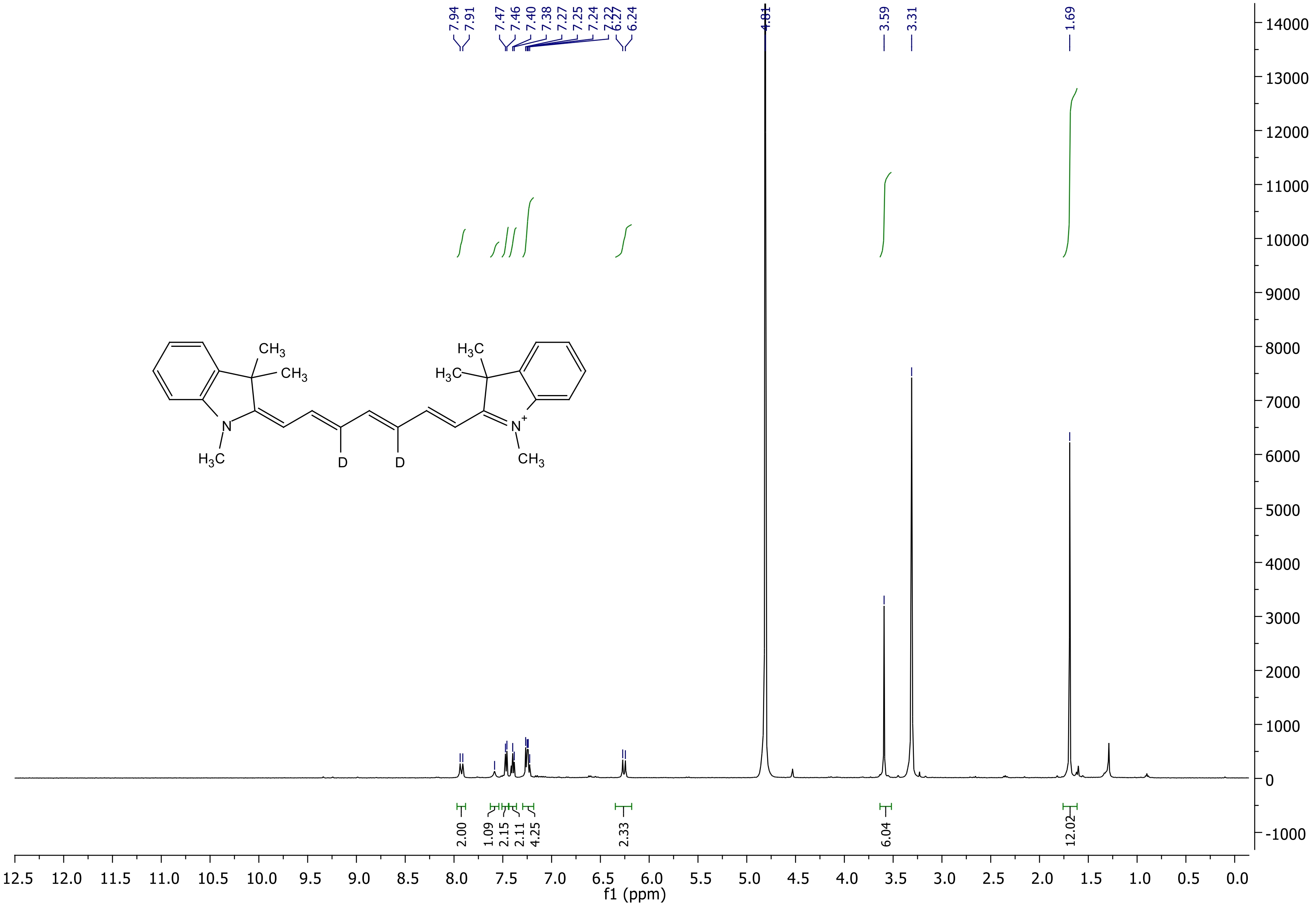

Figure S117. ^{1}H NMR (500 MHz, DMSO-d_6) of **Cy7-3',5'-d_2**.

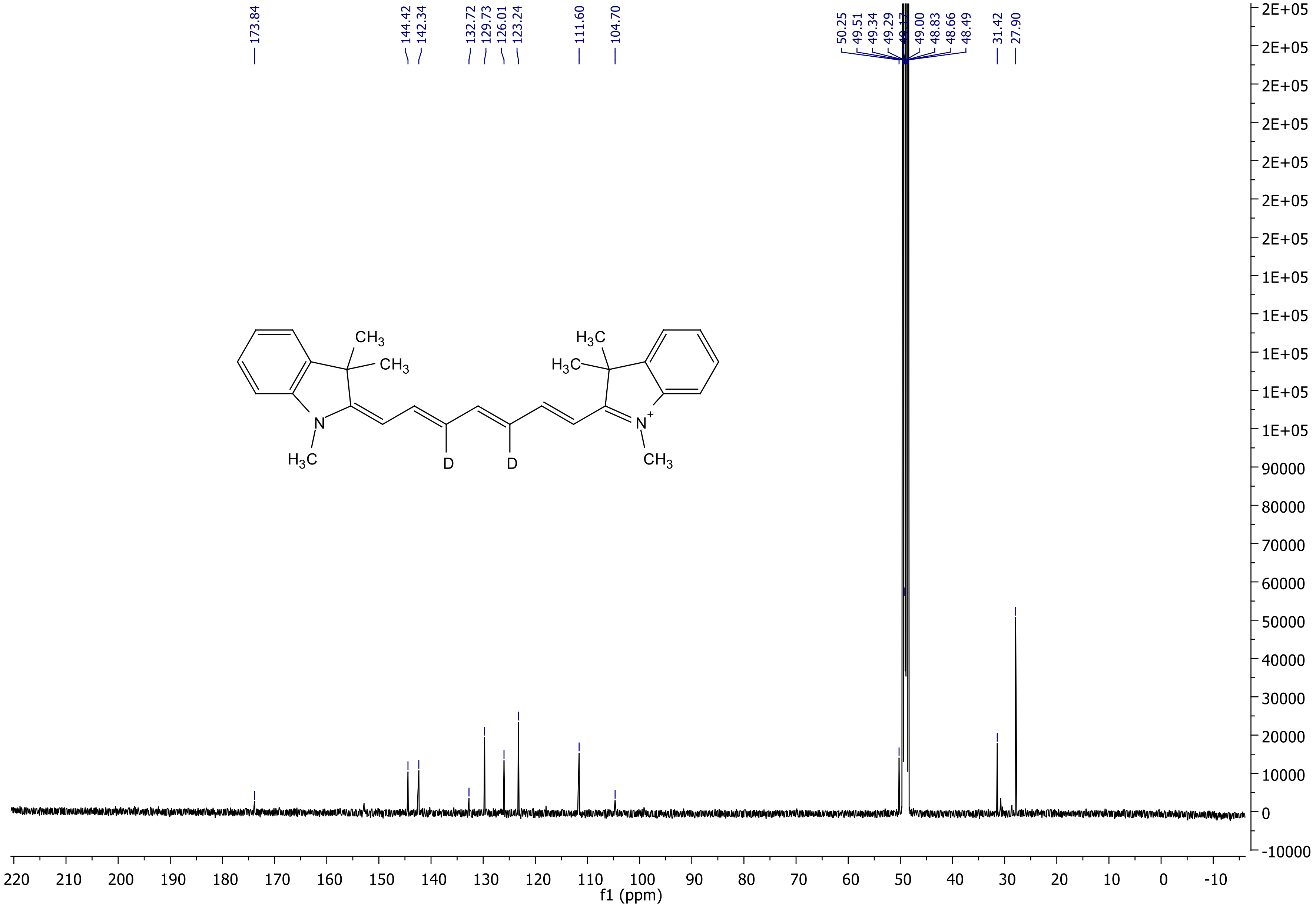

Figure S118. ^{13}C NMR (126 MHz, DMSO-d$_6$) of **Cy7-3',5'-*d*$_2$**.

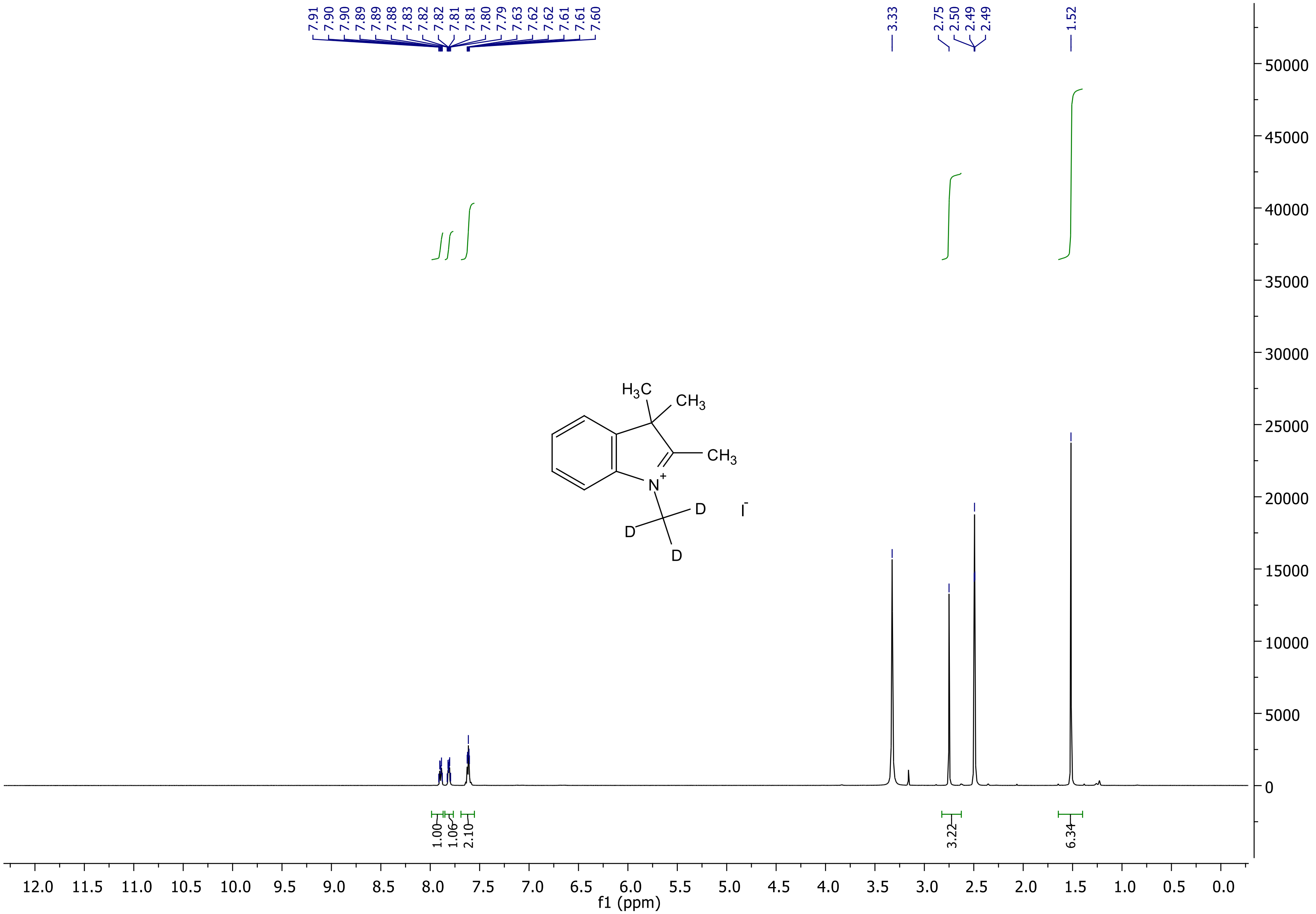

Figure S119. ^{1}H NMR (500 MHz, DMSO-d$_6$) of **16**.

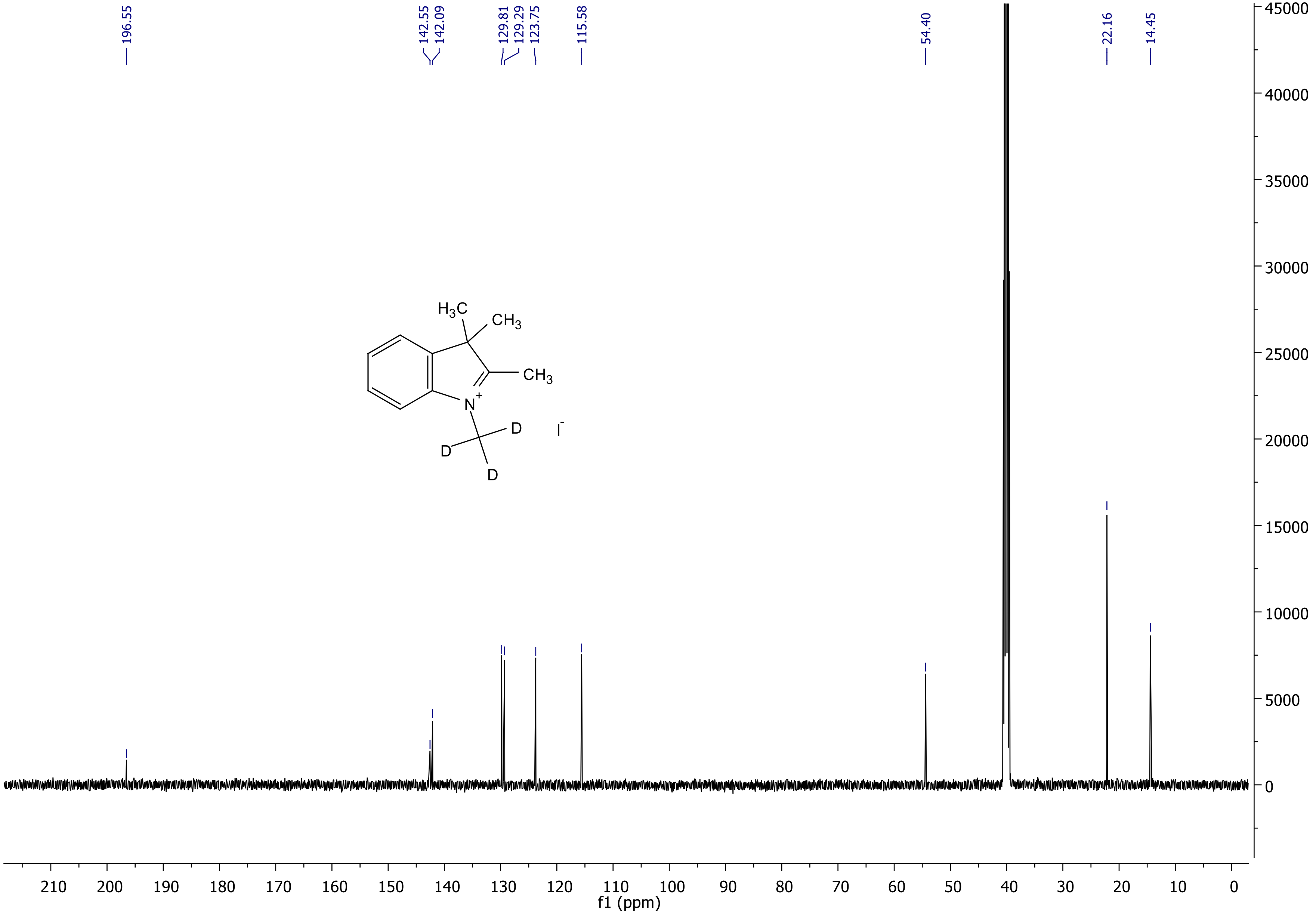

Figure S120. ^{13}C NMR (126 MHz, DMSO-d_6) of **16**.

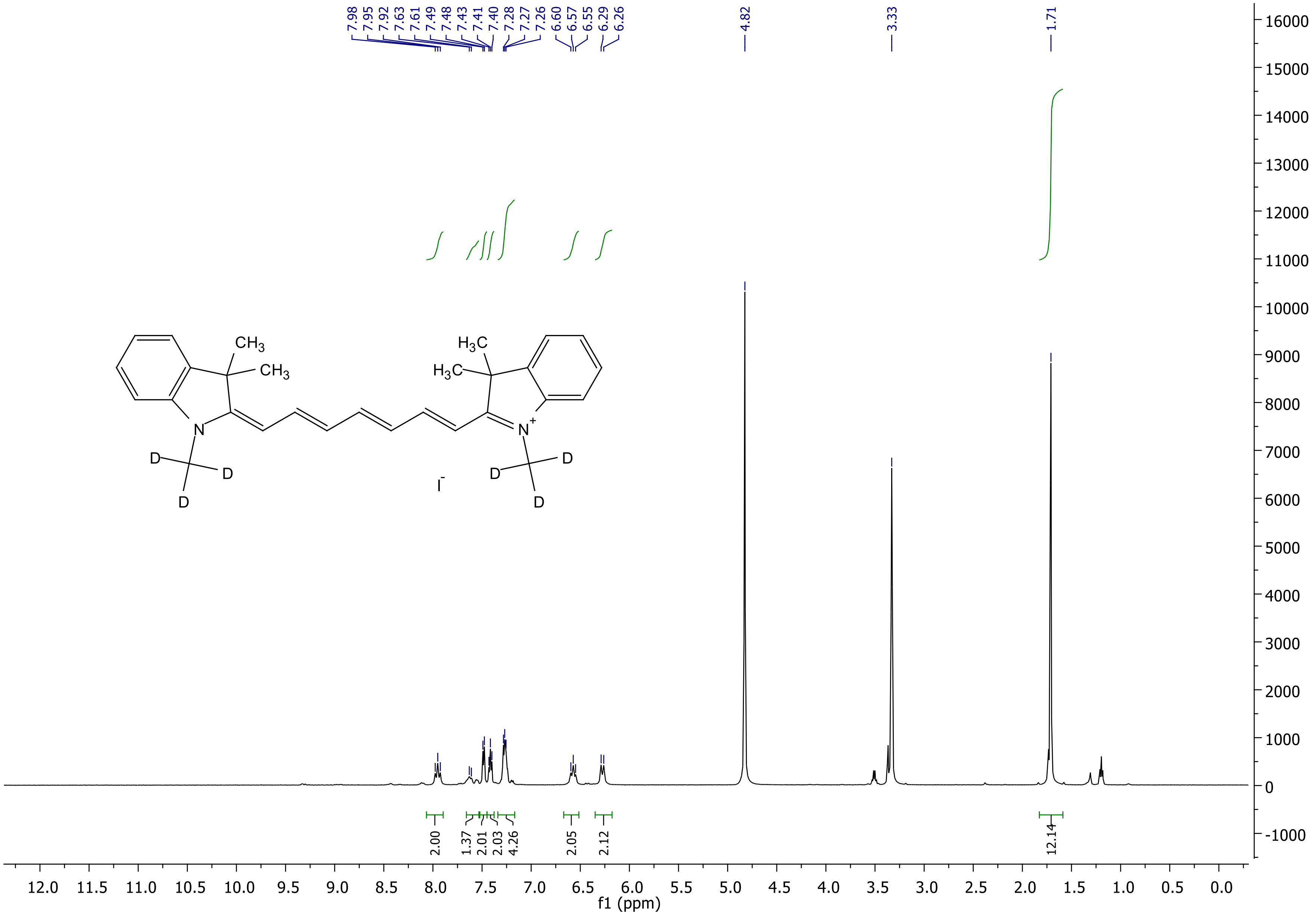

Figure S121. ^{1}H NMR (500 MHz, CD_3OD-d$_4$) of **Cy7-*d*$_6$** (dimethyl-*d*$_6$).

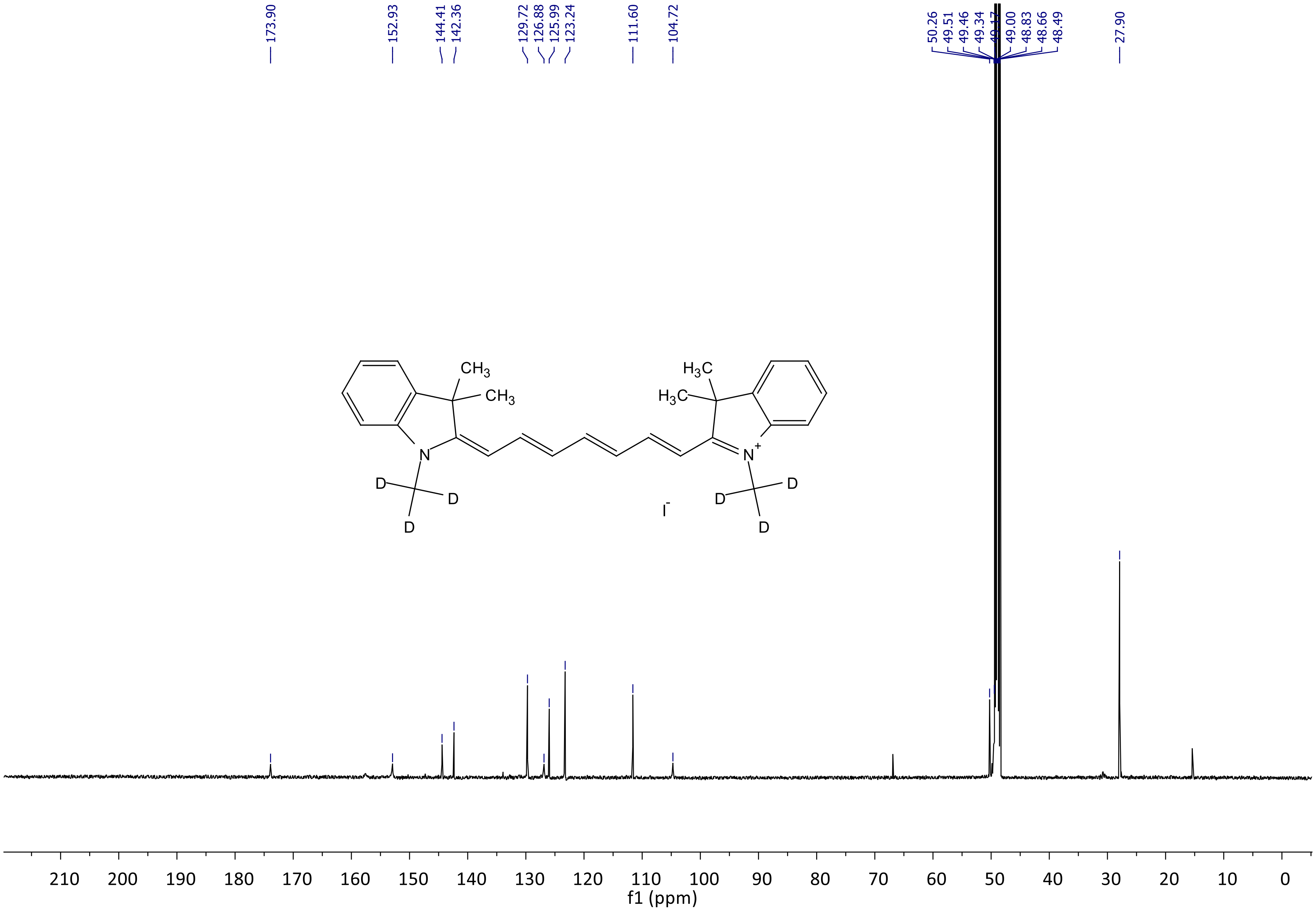

Figure S122. ^{13}C NMR (126 MHz, CD_3OD-d$_4$) of **Cy7-*d*$_6$** (dimethyl-*d*$_6$).

13. References

1. Stackova, L.; Stacko, P.; Klan, P., Approach to a Substituted Heptamethine Cyanine Chain by the Ring Opening of Zincke Salts. *J. Am. Chem. Soc.* **2019,** *141*, 7155-7162.
2. Lv, C. J.; Wan, C. W.; Liu, S.; Lan, Y.; Li, Y., Aryne trifunctionalization enabled by 3-silylaryne as a 1,2-benzdiyne equivalent. *Org. Lett.* **2018,** *20*, 1919-1923.
3. Glotz, G.; Polena, J.; Khan, N. M.; Mukherjee, A.; Kloz, M.; Slavíček, P.; Klán, P., The first microseconds of the life of excited heptamethine cyanine revealed by femtosecond stimulated Raman spectroscopy. *Commun. Chem.* **2025**, https://doi.org/10.1038/s42004-025-01850-2.
4. Janeková, H.; Friedman, H. C.; Russo, M.; Zyberaj, M.; Ahmed, T.; Hua, A. S.; Sica, A. V.; Caram, J. R.; Stacko, P., Deuteration of heptamethine cyanine dyes enhances their emission efficacy. *Chem. Commun.* **2024,** *60*, 1000-1003.
5. Pati, A. K.; El Bakouri, O.; Jockusch, S.; Zhou, Z.; Altman, R. B.; Fitzgerald, G. A.; Asher, W. B.; Terry, D. S.; Borgia, A.; Holsey, M. D.; Batchelder, J. E.; Abeywickrama, C.; Huddle, B.; Rufa, D.; Javitch, J. A.; Ottosson, H.; Blanchard, S. C., Tuning the Baird aromatic triplet-state energy of cyclooctatetraene to maximize the self-healing mechanism in organic fluorophores. *Proc. Nat. Acad. Sci. U.S.A.* **2020,** *117*, 24305-24315.
6. Madea, D.; Mujawar, T.; Dvorák, A.; Pospísilova, K.; Muchová, L.; Cubáková, P.; Kloz, M.; Svenda, J.; Vítek, L.; Klán, P., Photochemistry of (*Z*)-isovinylneoxanthobilirubic acid methyl Ester, a bilirubin dipyrrinone subunit: Femtosecond transient absorption and stimulated raman emission spectroscopy. *J. Org. Chem.* **2022,** *87*, 3089-3103.
7. Madea, D.; Mahvidi, S.; Chalupa, D.; Mujawar, T.; Dvorak, A.; Muchova, L.; Janos, J.; Slavicek, P.; Svenda, J.; Vitek, L.; Klan, P., Wavelength-dependent photochemistry and biological relevance of a bilirubin dipyrrinone subunit. *J. Org. Chem.* **2020,** *85*, 13015-13028.
8. Stackova, L.; Muchova, E.; Russo, M.; Slavicek, P.; Stacko, P.; Klan, P., Deciphering the structure-property relations in substituted heptamethine cyanines. *J. Org. Chem.* **2020,** *85*, 9776-9790.
9. Tutorials for Origin 9.0. https://www.originlab.com/pdfs/tutorials.pdf.
10. Feng, Y.; Vinogradov, I.; Ge, N. H., General noise suppression scheme with reference detection in heterodyne nonlinear spectroscopy. *Opt Express* **2017,** *25*, 26262-26279.
11. Janos, J.; Madea, D.; Mahvidi, S.; Mujawar, T.; Svenda, J.; Suchan, J.; Slavicek, P.; Klan, P., Conformational control of the photodynamics of a bilirubin dipyrrinone subunit: Femtosecond spectroscopy combined with nonadiabatic simulations. *J. Phys. Chem. A* **2020,** *124*, 10457-10471.
12. Mullen, K. M.; van Stokkum, I. H. M., The variable projection algorithm in time-resolved spectroscopy, microscopy and mass spectrometry applications. *Numer Algorithms* **2009,** *51*, 319-340.
13. Mullen, K. M.; van Stokkum, I. H. M., TIMP: An R package for modeling multi-way spectroscopic measurements. *J. Stat. Softw.* **2007,** *18*, 1-46.
14. Kovalenko, S. A.; Dobryakov, A. L.; Ruthmann, J.; Ernsting, N. P., Femtosecond spectroscopy of condensed phases with chirped supercontinuum probing. *Phys. Rev. A* **1999,** *59*, 2369-2384.
15. Slavov, C.; Hartmann, H.; Wachtveitl, J., Implementation and evaluation of data analysis strategies for time-resolved optical spectroscopy. *Anal. Chem.* **2015,** *87*, 2328-2336.
16. Liu, Y. L.; Chaudhari, A. S.; Chatterjee, A.; Andrikopoulos, P. C.; Picchiotti, A.; Rebarz, M.; Kloz, M.; Lorenz-Fonfria, V. A.; Schneider, B.; Fuertes, G., Sub-millisecond photoinduced dynamics of free and EL222-bound FMN by stimulated raman and visible absorption spectroscopies. *Biomolecules* **2023,** *13*, 161.
17. Kloz, M.; Weissenborn, J.; Polívka, T.; Frank, H. A.; Kennis, J. T. M., Spectral watermarking in femtosecond stimulated Raman spectroscopy: resolving the nature of the carotenoid S* state. *Phys. Chem. Chem. Phys.* **2016,** *18*, 14619-14628.
18. Kovalchuk, A. I.; Kobzar, Y. L.; Tkachenko, I. M.; Kurioz, Y. I.; Tereshchenko, O. G.; Shekera, O. V.; Nazarenko, V. G.; Shevchenko, V. V., Photoactive fluorinated poly(azomethine)s with azo groups in the main chain for optical storage applications and controlling liquid crystal orientation. *ACS Appl. Polym. Mater.* **2020,** *2*, 455-463.
19. Sellet, N.; Clement-Comoy, L.; Elhabiri, M.; Cormier, M.; Goddard, J. P., Second generation of near-infrared cyanine-based photocatalysts for faster organic transformations. *Chem. Eur. J.* **2023,** *29*, e202302353.
20. Ole Hammerich, B. S., *Organic electrochemistry*. 5th ed.; CRC Press: 2015.
21. Mohammad, M.; Khan, A. Y.; Subhani, M. S.; Bibi, N.; Ahmad, S.; Saleemi, S., Kinetics and electrochemical studies on superoxide. *Res. Chem. Intermediates* **2001,** *27*, 259-267.
22. Romero, N. A.; Nicewicz, D. A., Organic photoredox catalysis. *Chem. Rev.* **2016,** *116*, 10075-10166.

23. Zón, M.; Fernández, H.; Montoya, M. R.; Mellado, J. R., Contribution to the elucidation of the redox behaviour of camphorquinone I. Mechanism of the reduction at mercury and platinum electrodes in aqueous and acetonitrile solutions. *Electrochim. Acta* **1993,** *38*, 2209-2216.
24. Morlet-Savary, F.; Klee, J. E.; Pfefferkorn, F.; Fouassier, J. P.; Lalevée, J., The camphorquinone/amine and camphorquinone/amine/phosphine oxide derivative photoinitiating systems: Overview, mechanistic approach, and role of the excitation light source. *Macromol. Chem. Phys.* **2015,** *216*, 2161-2170.
25. AlNashef, I. M.; Leonard, M. L.; Kittle, M. C.; Matthews, M. A.; Weidner, J. W., Electrochemical generation of superoxide in room-temperature ionic liquids. *Electrochem. Solid-State Lett.* **2001,** *4*, D16-D18.
26. Adams, G. E.; Clarke, E. D.; Flockhart, I. R.; Jacobs, R. S.; Sehmi, D. S.; Stratford, I. J.; Wardman, P.; Watts, M. E.; Parrick, J.; Wallace, R. G.; Smithen, C. E., Structure-activity-relationships in the development of hypoxic cell radiosensitizers. 1. Sensitization efficiency. *Int. J. Radiat. Biol.* **1979,** *35*, 133-150.
27. Wardman, P., Reduction potentials of one-electron couples involving free-radicals in aqueous-solution. *J. Phys. Chem. Ref. Data* **1989,** *18*, 1637-1755.
28. Bockman, T. M.; Kochi, J. K., Isolation and oxidation reduction of methylviologen cation radicals - novel disproportionation in charge-transfer salts by X-Ray crystallography. *J. Org. Chem.* **1990,** *55*, 4127-4135.
29. Jasik, J.; Zabka, J.; Roithova, J.; Gerlich, D., Infrared spectroscopy of trapped molecular dications below 4 K. *Int. J. Mass Spectrom.* **2013,** *354*, 204-210.
